# Supplementary material for: Bimodal centromeres in pentaploid dogroses shed light on their unique meiosis
Source: Nature. 2025 Jun 18;643(8070):148–57. doi: 10.1038/s41586-025-09171-z (PMC12222009; doi:10.1038/s41586-025-09171-z)
Supplement: Supplementary file 1 — Supplementary Tables 1–4, Supplementary Figs 1–20 and a guide for Supplementary Data 1–17. [file 41586_2025_9171_MOESM1_ESM.pdf]

nature portfolio

https://doi.org/10.1038/s41586-025-09171-z

Supplementary information

Bimodal centromeres in pentaploid dogroses shed light on their unique meiosis

In the format provided by the
authors and unedited

Supplementary Information

Bimodal centromeres in pentaploid dogroses shed light on their unique meiosis

Herklotz, V.[1*], Zhang, M.[2*], Nascimento, T.[2*], Kalfusová, R.[3], Lunerová, J.[3], Fuchs, J.[4], Harpke, D.[4], Huettel, B.[5], Pfordt, U.[2], Wissemann, V.[6], Kovařík[3#], A., Marques, A.[2,7,10,#], Ritz, C.M.[1,8,9,#]

[1]*Senckenberg Museum for Natural History Görlitz, Senckenberg – Leibniz Institution for Biodiversity and Earth System Research, Am Museum 1, D-02826 Görlitz, Germany*

[2]*Department of Chromosome Biology, Max Planck Institute for Plant Breeding Research, Carl-von-Linné-Weg 10, D-50829 Cologne, Germany*

[3]*Department of Molecular Epigenetics, Institute of Biophysics, Czech Academy of Sciences, v.v.i., Královopolská 135, CZ-612 65 Brno, Czech Republic*

[4]*Leibniz Institute of Plant Genetics and Crop Plant Research (IPK) Gatersleben, Corrensstraße 3, D-06466 Seeland, Germany*

[5]*Max Planck Genome Centre Cologne, Max Planck Institute for Plant Breeding Research, Carl-von-Linné-Weg 10, D-50829 Cologne, Germany*

[6]*Institute of Botany, Systematic Botany Group, Justus-Liebig-University, Heinrich-Buff-Ring 38, D-35392 Gießen, Germany,*

[7]*Cluster of Excellence on Plant Sciences (CEPLAS), Heinrich-Heine University, Düsseldorf, Germany*

[8]*International Institute (IHI) Zittau, Dresden University of Technology, Markt 23, D-02763 Zittau, Germany*

[9]*German Centre for Integrative Biodiversity Research (iDiv) Halle-Jena-Leipzig, Puschstraße 4, D-04103 Leipzig, Germany*

[10]Lead contact

*These authors contributed equally.

#Correspondence: Aleš Kovařík (kovarik@ibp.cz), André Marques (amarques@mpipz.mpg.de), Christiane Ritz (christiane.ritz@senckenberg.de)

Table of contents

Supplementary Tables

Supplementary Table 1. Contig and scaffold assembly statistics of *R. canina* (S27). 4

Supplementary Table 2. Contig and scaffold assembly statistics of *R. canina* and *R. agrestis* both from the Darwin Tree of Life (DToL). 5

Supplementary Table 3. *Rosa canina* (S27) centromere composition. 6

Supplementary Table 4. *Rosa canina* (S27) gene and transcripts counts in each subgenome compared to expectations. 7

Supplementary Figures

Supplementary Figure 1. All-to-all dotplot comparisons of the assembled *R. canina* (S27) chromosomes. 8

Supplementary Figure 2. Orthologous cluster analysis of *R. canina* (S27) subgenomes. 9

Supplementary Figure 3. Self-synteny analysis of the *R. canina* (S27) genome. 10

Supplementary Figure 4. Synteny analysis between the diploid roses *R. chinensis* (**a**) and *R. rugosa* (**b**) with the *R. canina* (S27) genome. 11

Supplementary Figure 5. Analysis of gene fractionation bias in the *R. canina* (S27) genome. 12

Supplementary Figure 6. Evolutionary divergence and relative divergence time estimates among *R. canina* (S27) subgenomes and related species. 13

Supplementary Figure 7. Subgenome phasing and characterisation of the DToL genome assemblies for the pentaploid *R. canina* and *R. agrestis*. 14

Supplementary Figure 8. Comparative subgenome phasing between *R. canina* (S27) and *R. agrestis* DToL. 15

Supplementary Figure 9. Diagrammatical representation of the overal repeat composition in the *R. canina* (S27) genome determined by the RepeatExplorer2 clustering analysis. 16

Supplementary Figure 10. The organisation and structure of *R. canina* (S27) centromeres. 17

Supplementary Figure 11. Comparative dot plot structural analysis of centromeres between the genomes of the assembled *R. canina* (S27) and *R. canina* DToL using 85% similarity threshold. 25

Supplementary Figure 12. Comparative dot plot structural analysis of centromeres between the genomes of the assembled *R. canina* (S27) and *R. agrestis* DToL using 85% similarity threshold. 26

Supplementary Figure 13. Characterisation of four centromeric LTRs (*cenLTR1–4*) found in the centromere of Rca4_*R4* of *R. canina* (S27) genome. 27

Supplementary Figure 14. Metaplot of CENH3 enrichment, DNA methylation, and centromeric elements—*ATHILA* and *CANR4* density—on each chromosome. 28

Supplementary Figure 15. Total centromeric abundance of *CANR4* and *ATHILA* repeats on each subgenome across *R. canina* (S27), *R. canina* DToL and *R. agrestis* DToL. 30

Supplementary Figure 16. Pollen viability screening in *R. canina* (S27). 31

Supplementary Figure 17. Dogrose female meiosis illustrations from Täckholm 1922. 32

Supplementary Figure 18. Gene maps of the chloroplast genome of *R. canina* (S27) differing in the orientation of their single copy units. 33

Supplementary Figure 19. Gating strategy to isolate generative nuclei of *R. canina* as presented in **Fig. 2a**. 34

Supplementary Figure 20. Gene number comparison among the subgenomes of *R. canina* (S27) and clustering of top 100 highly expressed genes. 35

Supplementary Videos (provided as separated files)

Supplementary Video 1. STED-imaged mitotic metaphase. *Rosa canina* (S27) mitotic metaphase immunolabeled with antibody against CENH3 protein. Please note the size difference of CENH3 signals among different chromosomes (Figure 4a).

Supplementary Video 2. STED-imaged early prophase I. CENH3 immunostaining during early prophase I of *R. canina* (S27) (Figure 4b).

Supplementary Video 3. STED-imaged early prophase I. CENH3 immunostaining during early prophase I of *R. canina* (S27) (Figure 4c).

Supplementary Video 4. STED-imaged diakinesis. CENH3 immunostaining in diakinesis of *R. canina* (S27) (Figure 4d).

Supplementary Video 5. STED-imaged pro-metaphase I. CENH3 immunostaining in pro-metaphase I of *R. canina* (S27) (Figure 4e).

Supplementary Video 6. STED-imaged metaphase I. CENH3 and alpha-Tubulin immunostaining during metaphase I of *R. canina* (S27) (Figure 4f).

Supplementary Video 7. STED-imaged early anaphase I. CENH3 and alpha-Tubulin immunostaining during anaphase I of *R. canina* (S27) (Figure 4g).
Supplementary Video 8. STED-imaged late anaphase I. CENH3 and alpha-Tubulin immunostaining during anaphase I of *R. canina* (S27) (Figure 4h).
Supplementary Video 9. STED-imaged metaphase II. CENH3 and alpha-Tubulin immunostaining during metaphase II of *R. canina* (S27) (Figure 4i).
Supplementary Video 10. STED-imaged anaphase II. CENH3 and alpha-Tubulin immunostaining during anaphase II of *R. canina* (S27) (Figure 4j).
Supplementary Video 11. STED-imaged polyad. CENH3 immunostaining in a polyad of *R. canina* (S27) showing different nuclei with varying number of centromeric foci.

Supplementary Datasets (provided as separated files)

Supplementary Dataset 1. Pairwise comparisons of *R. canina* (S27), *R. chinensis* and *R. rugosa* chromosomes.

Supplementary Dataset 2. Summary of SCO pollen mapping for chromosome hit pair counts.

Supplementary Dataset 3. Maximum Likelihood SCO phylogenies per synteny group.

Supplementary Dataset 4. Repeat annotation and characterization of the *R. canina* (S27) genome with DANTE, DANTE-LTR and TideCluster.

Supplementary Dataset 5. Centromere length and sequence composition in *R. canina* (S27).

Supplementary Dataset 6. Centromere length and sequence composition in *R. canina* DToL.

Supplementary Dataset 7. Centromere length and sequence composition in *R. agrestis* DToL.

Supplementary Dataset 8. Structural analysis of whole chromosome and centromeres of bivalent- and univalent-forming chromosomes of *R. agrestis* DToL.
Supplementary Dataset 9. Structural analysis of whole chromosome and centromeres of bivalent- and univalent-forming chromosomes of *R. chinensis*.

Supplementary Dataset 10. Centromere length and sequence composition in *R. chinensis*.

Supplementary Dataset 11. Structural analysis of whole chromosome and centromeres of bivalent- and univalent-forming chromosomes of *R. rugosa* DToL.
Supplementary Dataset 12. The target sequences of single copy orthologue (SCO) designed based on Debray et al. (2019) and *Rosa chinensis* haploid line genome v.1.0.
Supplementary Dataset 13. Glmm statistics of centromere composition in *R. canina* (S27), *R. canina* DToL and *R. agrestis* DToL.
Supplementary Dataset 14. Consensus sequence of four centromeric LTRs forming tandem arrays in *R. canina* (S27) genome.
Supplementary Dataset 15. CENH3 abundance and amount of *CANR4* sequences across *R. canina* (S27) centromeres.
Supplementary Dataset 16. Centromere sequence composition and length across different dogrose's subgenomes.

Supplementary Dataset 17. Overview about sampled material.

Supplementary Table 1. Contig assembly statistics of *R. canina* (S27).

	Contig hap1 (2x)	Contig hap2 (3x)	S1_h1 scaffolds	S1_h2 scaffolds	S2 scaffolds	R3 scaffolds	R4 scaffolds	Unplaced contigs
Total length (bp)	1003260183	1477873413	517908549	516677462	496701192	449860202	432931235	84057232
Count	3423	1216	7	7	7	7	7	4522
Largest sequence (bp)	68054406	69739602	90969763	89045000	87692573	79498036	77221208	1937460
N50 (bp)	24826522	43421611	72060088	72013834	69904013	62316241	67565529	63534
N90 (bp)	4899689	10288194	53247335	53695739	50635429	49798260	44791695	34132
L50	14	14	4	4	4	4	3	251
L90	41	38	7	7	7	7	7	1049
GC (%)	39.29	39.12	38.98	38.98	38.86	39.1	39.27	43.67
Gaps	-	-	12	13	21	14	24	-

N.B.: Since ploidy level was set as 2, the contig assembly was output as hap1 and hap2 with HiFiasm. After subgenome assignment for the final genome assembly, it was shown that hap1 contigs finally comprise subgenome *S1*_h2, *R3*; hap2 contigs comprise subgenome *S1*_h1, *S2*, *R4*. Note that the unplaced contigs shown here include all contigs from hap1 and hap2 that were not scaffolded into pseudochromosomes. Hence, the unplaced contig sequences are potential sequences from 5 haplotypes. Many of them are homologous sequences or repetitive sequences.

Supplementary Table 2. Contig assembly and scaffolding statistics of *R. canina* and *R. agrestis* both from the Darwin Tree of Life (DToL).

	Rosa canina DToL			*Rosa agrestis* DToL		
	Contigs hap1 (2n)	Contigs hap2 (3n)	Scaffolding (5n)	Contigs hap1 (2n)	Contigs hap2 (3n)	Scaffolding (5n)
Total length (bp)	1,026,227,128	1,462,284,126	2,400,414,864	1,021,286,406	1,414,578,319	2,363,849,846
Number	1,208	286	35	1,125	341	35
Largest contig (bp)	74,947,926	85,059,557	92,373,536	72,185,930	70,918,500	92,789,151
N50 (bp)	56,674,350	44,041,928	71,138,518	29,293,717	34,136,793	71,924,096
N90 (bp)	11,396,600	11,992,407	51,931,363	9,544,858	11,916,992	55,628,520
L50	8	13	16	11	14	15
L90	23	36	31	32	38	31
Gaps	0	0	123	0	0	117
Unscaffolded (bp)	0	0	88,034,890	0	0	72,073,379

Supplementary Table 3. ***Rosa canina*** **(S27) centromere composition.** The abundance of centromeric and pericentromeric repeats were compared in qualitative manner for each chromosome and synteny group. Detailed quantitative quantifications are provided in Supplementary Dataset 5.

synteny group	chromosome	Meiotic behaviour	LTR T3/Gypsy/ ATHILA	CANR4 satellite	others	Protein coding genes embedded in CANR4 arrays
1	S1_h1	Bivalent	++++	+/-		
1	S1_h2	Bivalent	++++	+/-		
1	S2	Univalent	+	++++		
1	R4	Univalent	+++		++	
1	R3	Univalent	+	++++		+
2	S1_h1	Bivalent	++	+++		
2	S1_h2	Bivalent	++	+++		
2	S2	Univalent	+	++++		
2	R4	Univalent	+	+++		+
2	R3	Univalent	+	++++		
3	S1_h1	Bivalent	+++++			
3	S1_h2	Bivalent	+++++			
3	S2	Univalent	+	++++		
3	R4	Univalent	+++++			
3	R3	Univalent	+/-	++++		+
4	S1_h1	Bivalent	++	+++		
4	S1_h2	Bivalent	++	+++		
4	S2	Univalent	+/-	++++		
4	R4	Univalent	+++++			
4	R3	Univalent	+/-	++++		+
5	S1_h1	Bivalent	++	+++		
5	S1_h2	Bivalent	++	+++		
5	S2	Univalent	++++	+++++		
5	R4	Univalent	++++	+/-		
5	R3	Univalent	+/-	+++++		
6	S1_h1	Bivalent	+++++			
6	S1_h2	Bivalent	+++++			
6	S2	Univalent	+/-	+++++		+
6	R4	Univalent	+/-	++++		+
6	R3	Univalent		+++++		
7	S1_h1	Bivalent	+++		++	
7	S1_h2	Bivalent	+++		++	
7	S2	Univalent	+	++++		
7	R4	Univalent	+++++			
7	R3	Univalent	+/-	++++		

Supplementary Table 4. ***Rosa canina*** **(S27) gene and transcripts counts in each subgenome compared to expectations.**

Subgenome	Gene number	Transcript number	Transcript /Expectation
S1	36,783	20,842,646	1.00
S2	35,080	18,015,332	0.91
R3	36,123	18,513,914	0.90
R4	35,915	20,728,100	1.02

N.B.: The expectation rate here is defined as expected count of transcripts if we assume their number are proportional to gene number. Then we take the transcript number/gene number of subgenome *S1* as reference and other subgenome's transcript/expectation rate was normalized by *S1*. That is why the rate of *S1* subgenome is 1.00. So *S1* and *R4* has higher transcription activities in general comparing to *S2* and *R3* subgenome.

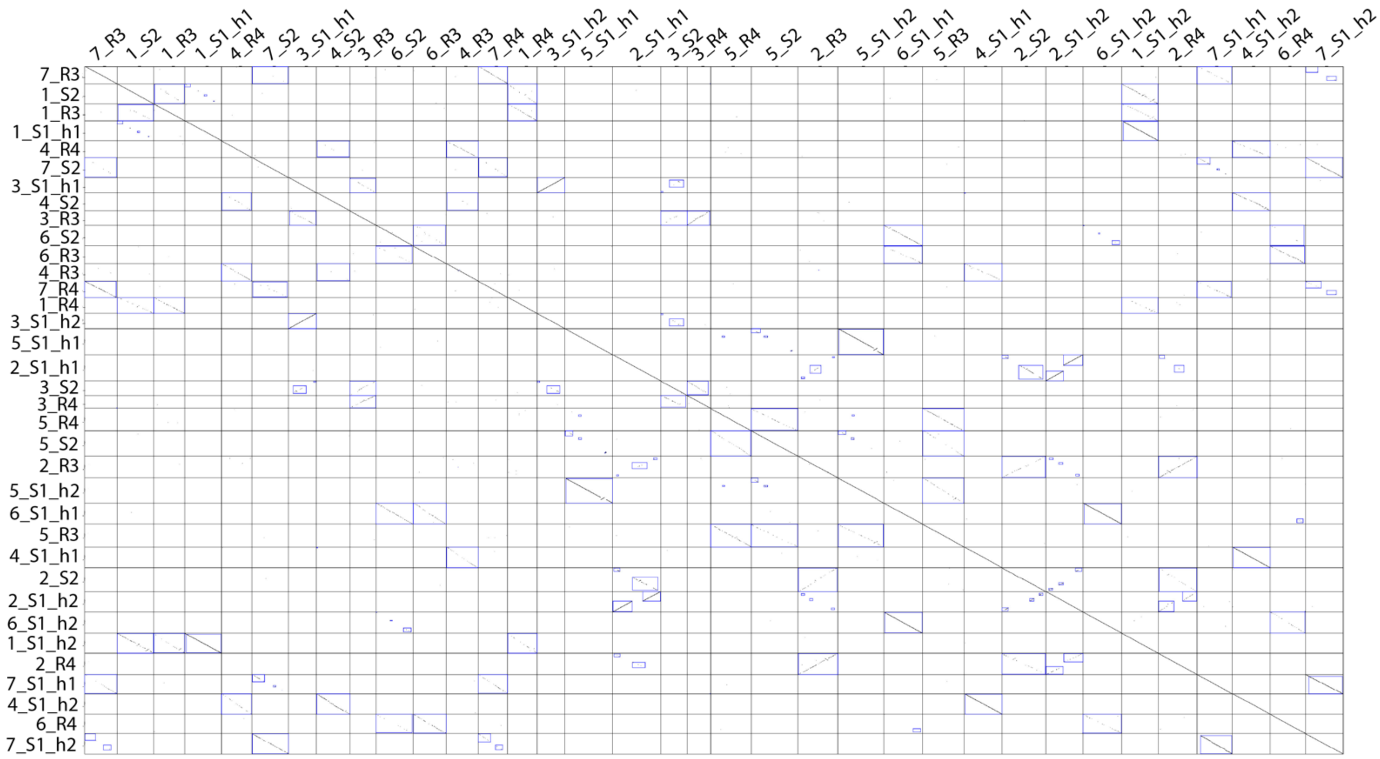

Supplementary Figure 1. All-to-all dotplot comparisons of the assembled *R. canina* (S27) chromosomes. The plot indicates the chromosomes showing self-synteny at a sequence similarity threshold of 98% across all the 35 scaffolded pseudochromosomes. Please note the high similarity between *S1* haplotypes and between *S1* and *S2* subgenomes, as well as between *R3* and *R4* subgenomes. In contrast, sequence similarity between *S* and *R* subgenomes is remarkably lower. Blue rectangles depict the syntenic regions identified that share >98% sequence similarity. Empty boxes show no similarities.

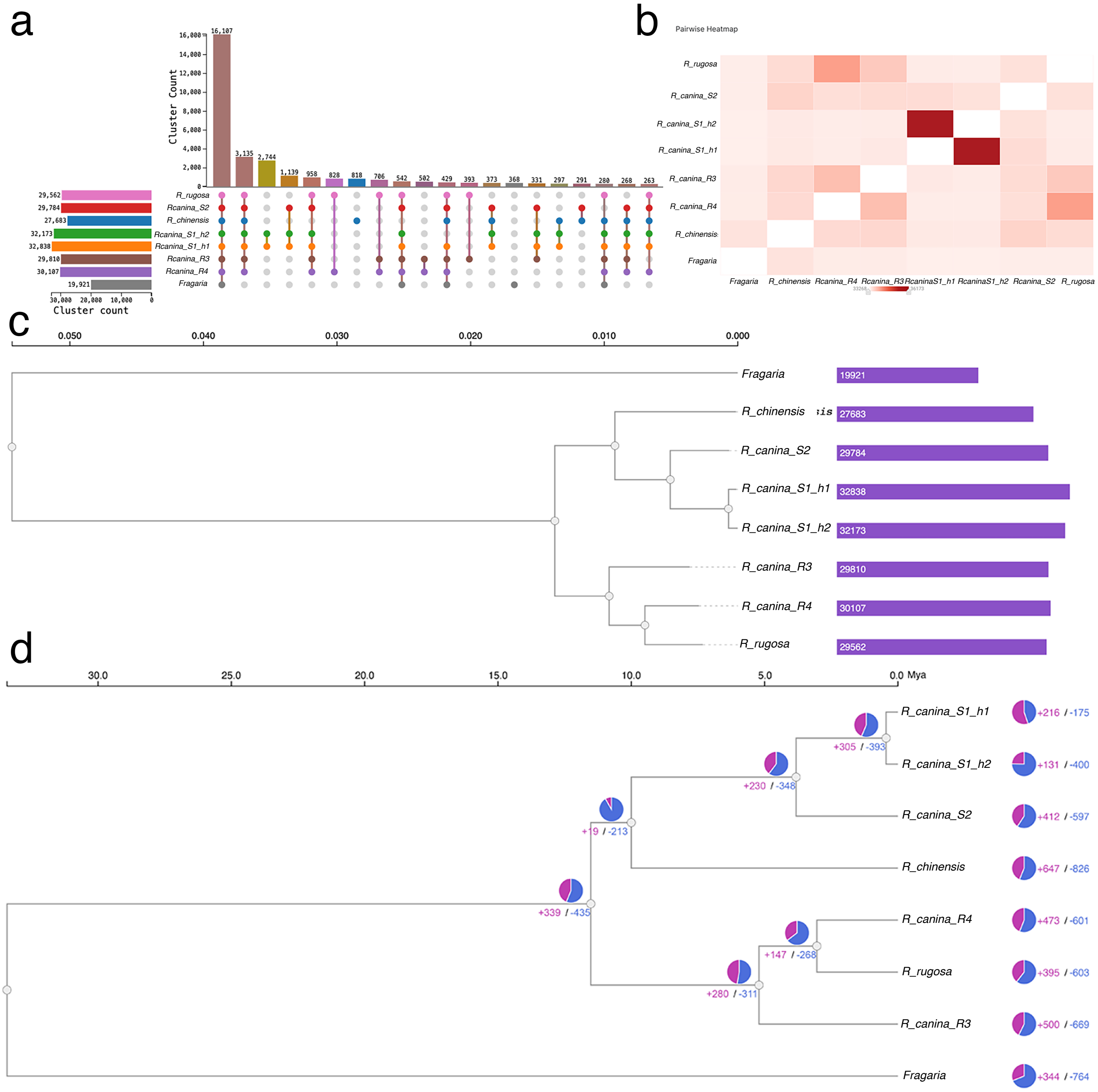

Supplementary Figure 2. Orthologous cluster analysis of *R. canina* (S27) subgenomes. (**a**) An UpSet table displays unique and shared orthologous clusters among the subgenomes and species. The left horizontal bar chart shows the number of orthologous clusters per sample, while the right vertical bar chart shows the number of orthologous clusters shared among the samples. The lines represent intersecting sets. (**b**) The heatmap shows the number of overlapping clusters between each pair of samples. (**c**) A phylogenetic tree based on single-copy genes illustrates the evolutionary relationships and distances among the species and subgenomes. (**d**) A pie chart shows the number of gene families that have expanded (purple) or contracted (blue) during evolution, while the phylogenetic tree shows the evolutionary timeline of the species.

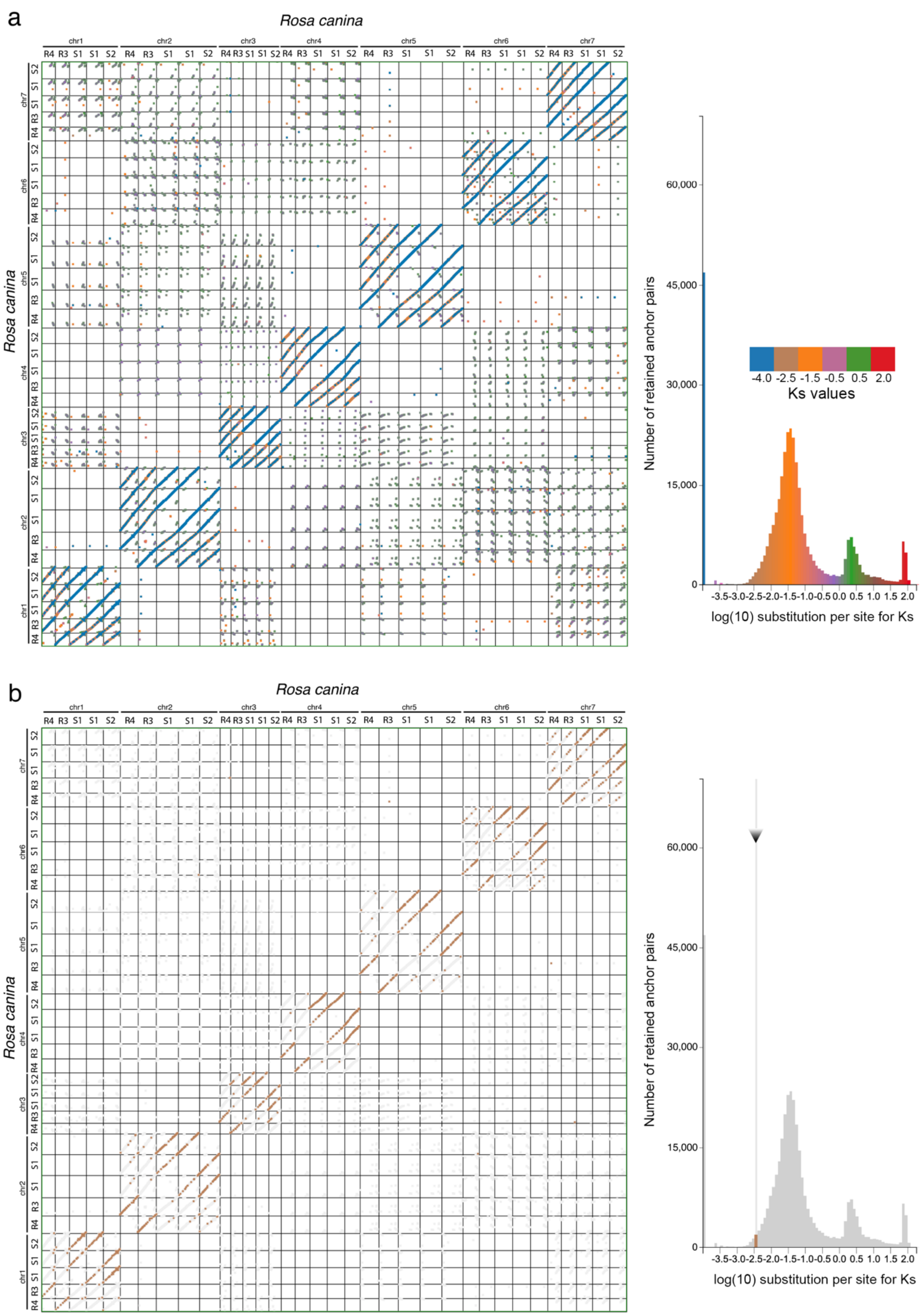

Supplementary Figure 3. Self-synteny analysis of the *R. canina* (S27) genome. (**a**) SynMap2 self-synteny dot plot coloured based on the distribution of synonymous (K_s) substitutions. K_s values on a decimal log scale are shown to the right of the dot plot. Note the large orange peak that correlates with the recent polyploidisation events in *R. canina* and a second small peak (green) most likely representing an ancient WGD. (**b**) Same plot as (**a**) but selecting only the sequences with the lowest number of synonymous substitutions, allowing the identification of the close relationships between *R3/R4* and *S1/S2* subgenomes, respectively. The small coloured block within the vertical grey bar represents the sequences with the lowest number of synonymous substitutions used in the dot plot to the left. K_s values are indicated by the colour scale in (**a**).

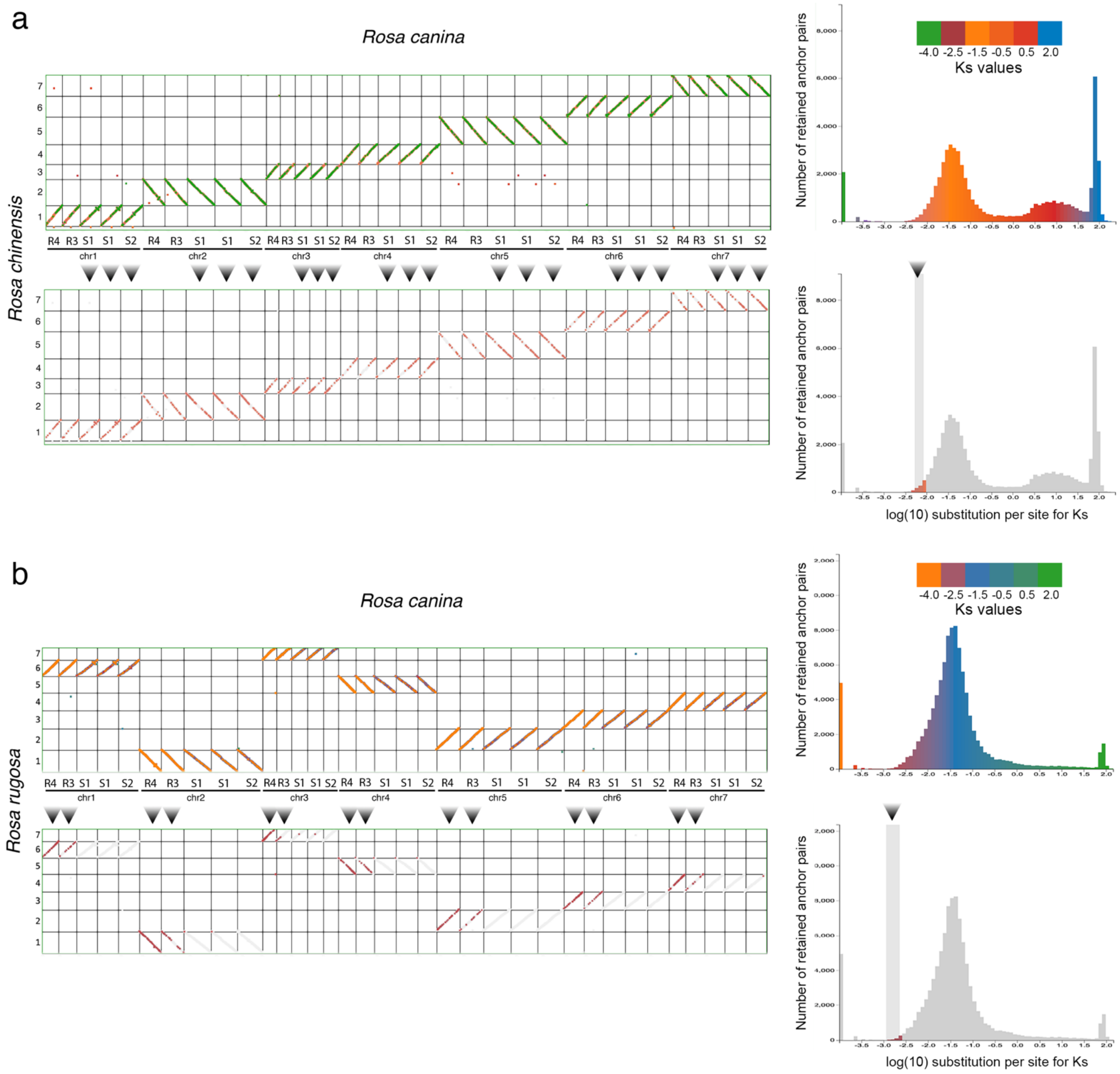

Supplementary Figure 4. Synteny analysis between the diploid roses *R. chinensis* (a) and *R. rugosa* (b) with the *R. canina* (S27) genome. (**upper plot**) SynMap2 synteny dot plot coloured based on K_s values. K_s values on a log scale are shown to the right of the dot plot. (**bottom plot**) Same plot as the upper plot but selecting only the sequences with the lowest number of synonymous substitutions allowing the identification of the close relationships between *S1/S2* and *R3/R4* subgenomes with *R. chinensis* (**a**) and *R. rugosa* (**b**), respectively. The small coloured block within the vertical grey bar represents the sequences with the lowest number of synonymous substitutions used in the dot plot to the left. K_s values are indicated by the colour scale in the upper plots. Shaded black triangles in **a** and **b** indicate low K_s values of *S1/S2* and *R3/R4* subgenomes when aligned to *R. chinensis* (**a**) and *R. rugosa* (**b**), respectively. Please note the higher amount of lower K_s values in all *R4* chromosomes compared to *R3* ones in **b**, corroborating the later introgression of the *R4* subgenome into modern *R. canina*.

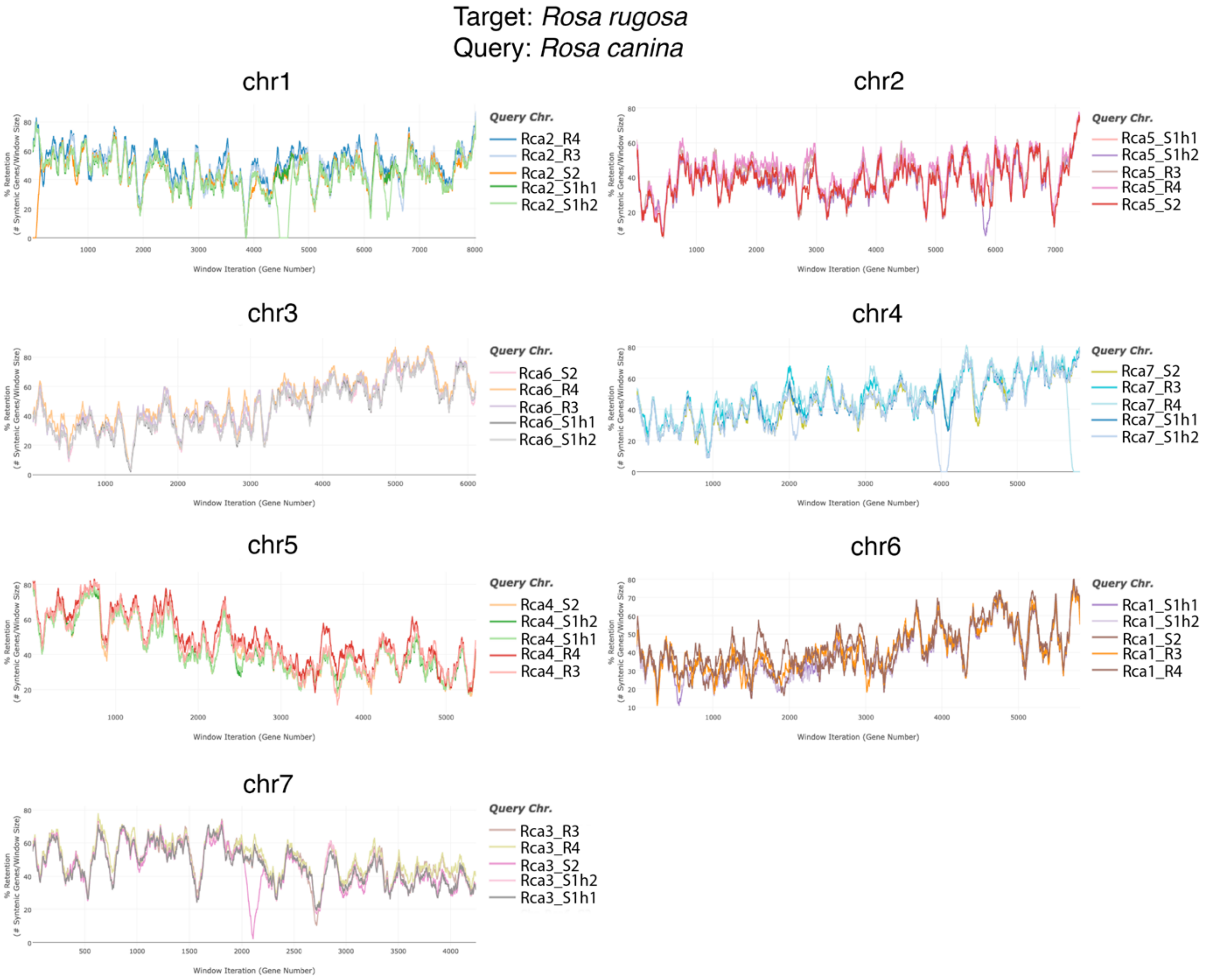

Supplementary Figure 5. Analysis of fractionation bias in the *R. canina* (S27) genome. The rate of syntenic gene retention was evaluated by aligning the *R. canina* genome (5*x*) against the *R. rugosa* genome (1*x*) Gene retention rates in synteny blocks were plotted along all *R. canina* chromosomes against *R. rugosa* chromosomes. Note the similar levels of gene retention across all *R. canina* subgenomes.

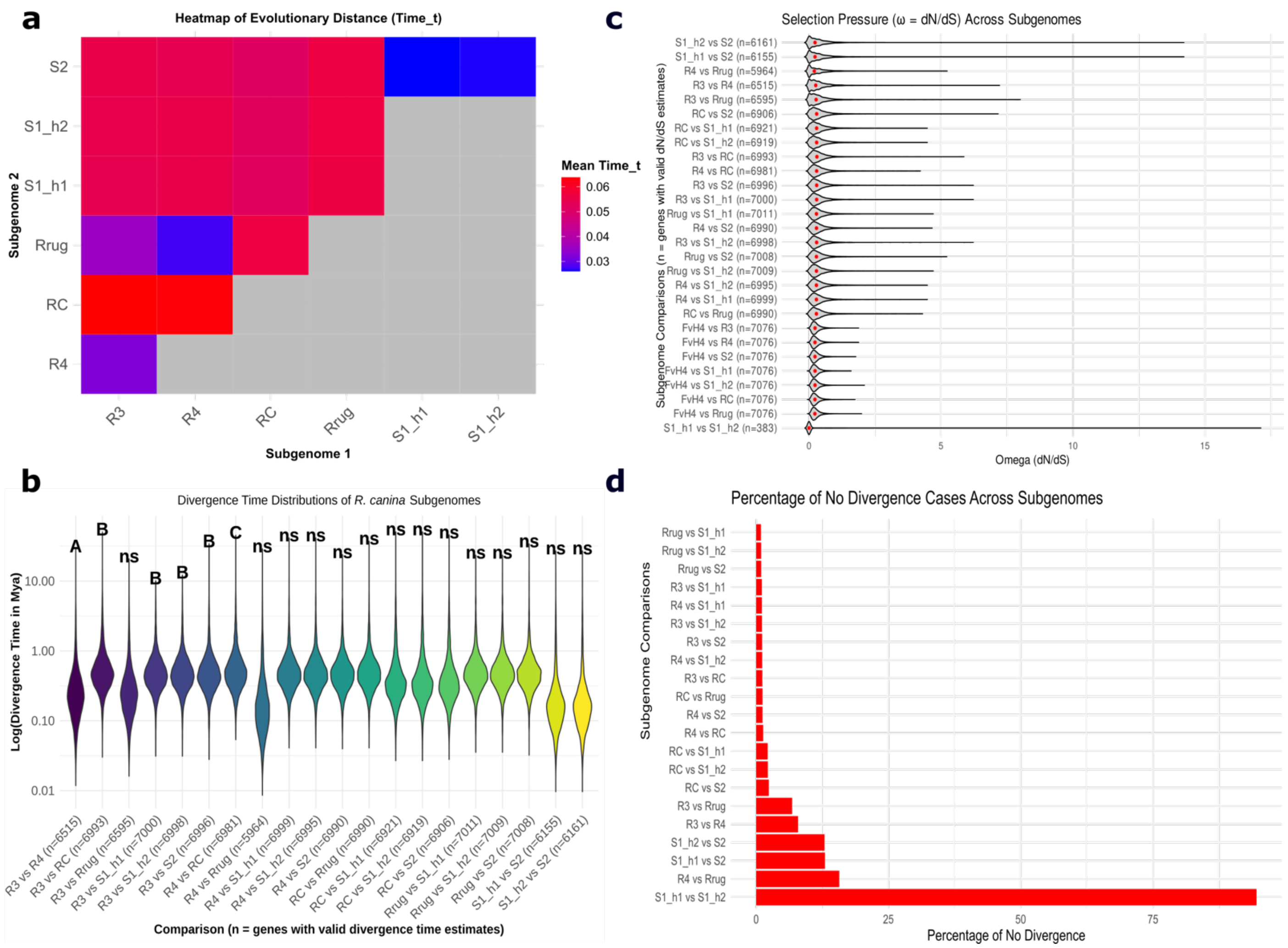

Supplementary Figure 6. Evolutionary divergence and relative divergence time estimates among *R. canina* (S27) subgenomes and related species. Orthologues were identified using OrthoFinder after extracting proteomes from *Fragaria vesca*, *Rosa rugosa*, and *Rosa chinensis* from available datasets (see Online Methods) resulting in 7076 single-copy orthologues. Codon-based alignments of CDS sequences were used for pairwise comparisons between the rate of non-synonymou and synonymous substitutions (dN/dS = ω) and evolutionary divergence estimation via PAML's yn00 function. (**a**) Heatmap displaying the mean evolutionary distance. *Fragaria vesca* and *S1*_h1 vs. *S1*_h2 comparisons were subsequently excluded due to excessive divergence (outgroup) and negligible divergence (haplotypes of the same S1 subgenome), respectively. (**b**) Violin plot of relative divergence time estimates (log10 scale), calibrated using the *Fragaria* stem fossil dated to 2.96 MYA (Matthews and Ovenden 1990; Matthews et al. 2003). *Fragaria vesca* and *S1*_h1 vs. *S1*_h2 were excluded for similar reasons as in (**a**). Only genes with valid estimates of divergence time were included (n per comparison shown in axis labels). Data distributions were non-normal (Shapiro-Wilk test: $W = 0.442$, $p < 2.2\times10^{-16}$), so group differences were tested using a Kruskal-Wallis rank-sum test ($\chi^2 = 32710$, $df = 19$, $p < 2.2\times10^{-16}$), followed by Dunn's post hoc test with Benjamini-Hochberg correction. Significance groupings (A, B, C) are shown; "ns" indicates non-significant differences (adjusted $p \geq 0.05$). (**c**) Selection pressure (dN/dS = ω) across *R. canina* subgenomes and related species. Boxplots display the distribution of ω (dN/dS) values. Boxes represent the interquartile range (IQR), whiskers extend to 1.5×IQR, and red dots indicate the median ω value per comparison. Individual gene-level ω estimates are overlaid as dots using a quasirandom layout to show data density. Only genes with valid ω estimates (excluding placeholder values) are counted, and sample sizes (n) refer to the number of biologically independent gene alignments per comparison, shown in the axis labels. Due to very low divergence between *S1_h1* and *S1_h2*, a large proportion of ω estimates were originally missing; these were assigned a placeholder value of 0.001 to retain them in the plot, but were excluded from sample size reporting (n). Comparisons are ordered by interquartile range to emphasize differences in variability across subgenome pairs. (**d**) Percentage of genes showing no divergence across *R. canina* subgenomes and related species. This plot illustrates the proportion of single-copy orthologues that exhibit no detectable divergence across different pairwise comparisons. For each comparison, the total number of genes, the count of genes with no detectable divergence, and their percentage were calculated. Comparisons involving *S1*_h1 vs. *S1*_h2 exhibited the highest proportion of no divergence, reflecting their status as haplotypes of the same subgenome. Abbreviations: Rrug = *Rosa rugosa*, RC = *Rosa chinensis*, FvH4 = *Fragaria vesca*.

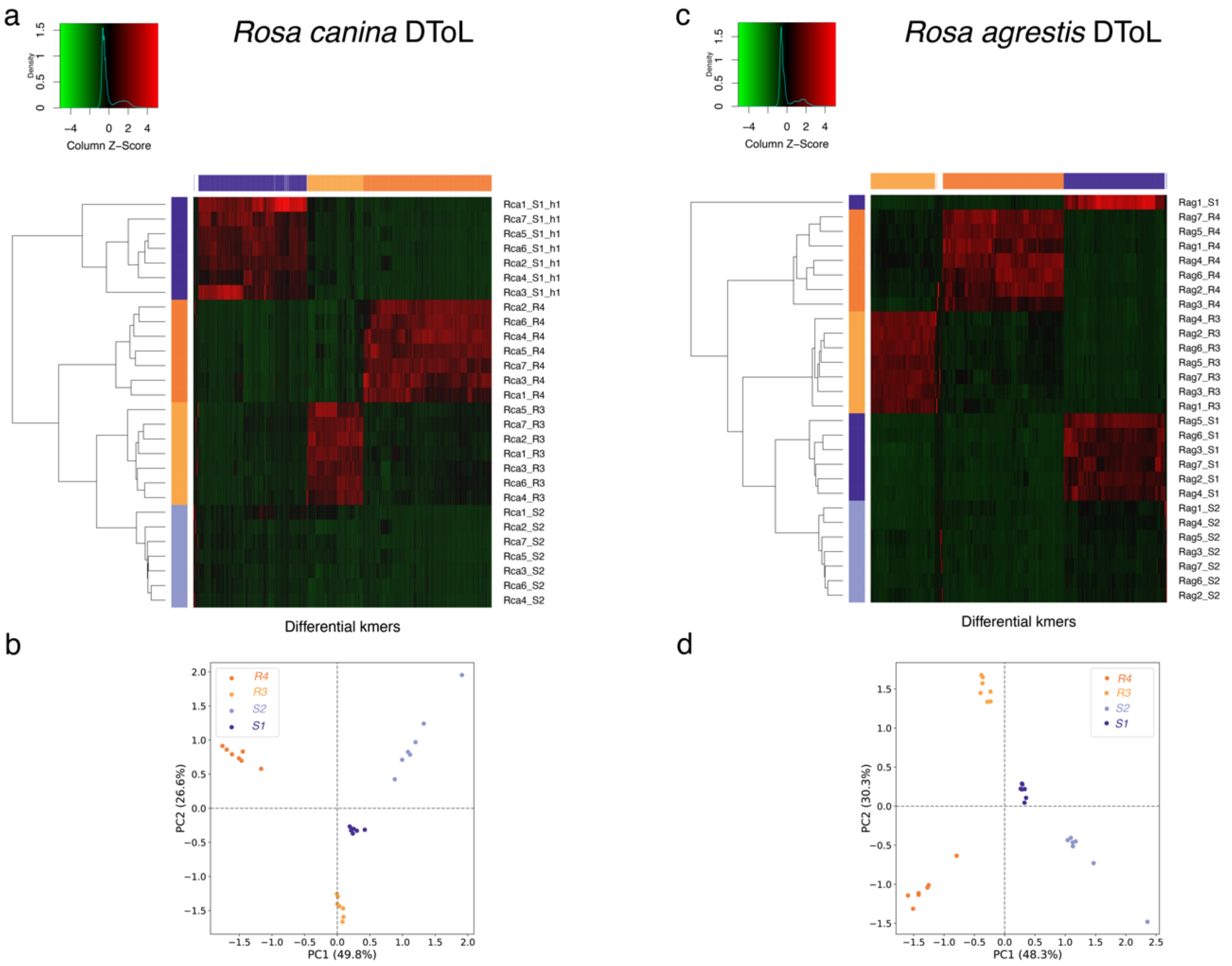

Supplementary Figure 7. Subgenome phasing and characterisation of the DToL genome assemblies for the pentaploid *R. canina* and *R. agrestis*. (**a** and **c**) Unsupervised hierarchical clustering (the horizontal colour bar at the top of the axis indicates to which subgenome the *k*-mer is specific; the vertical colour bar on the left of the axis indicates the subgenome to which the chromosome is assigned). The heatmap indicates the *Z*-scale relative abundance of *k*-mers. The larger the *Z* score is, the greater the relative abundance of a *k*-mer). (**b** and **d**) Principal Component Analysis (PCA) of differential 15-mers confirmed that the genome was successfully phased into four subgenomes based on clearly distinct patterns of both differential *k*-mers and homoeologous chromosomes.

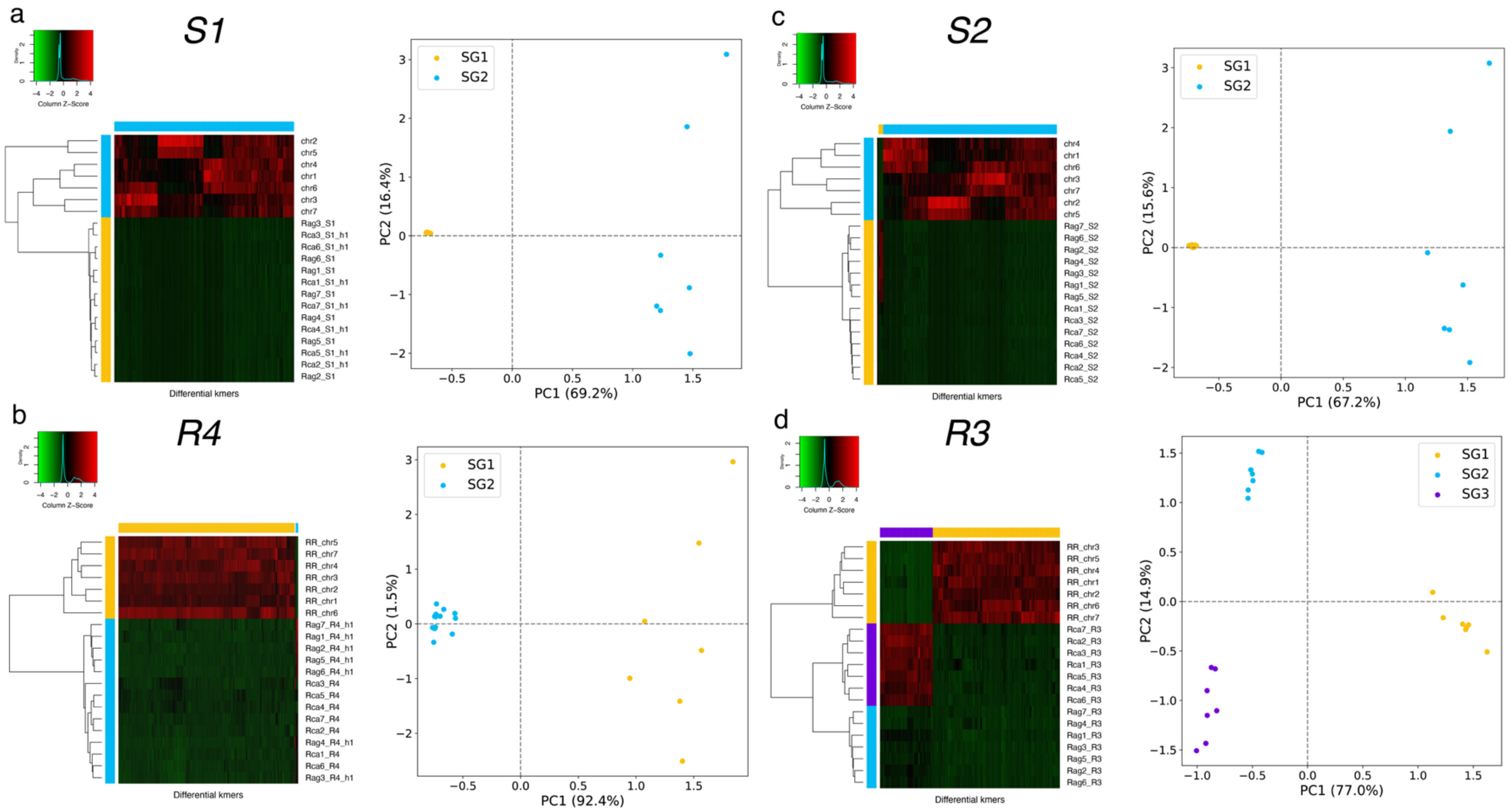

Supplementary Figure 8. Comparative subgenome phasing between *R. canina* (S27) and *R. agrestis* DToL. (**a–d**) Unsupervised hierarchical clustering (the horizontal colour bar at the top of the axis indicates to which subgenome the *k*-mer is specific; the vertical colour bar on the left of the axis indicates the subgenome to which the chromosome is assigned). The heatmap indicates the *Z*-scale relative abundance of *k*-mers. The larger the *Z* score is, the greater the relative abundance of a *k*-mer) (left panels). Principal Component Analysis (PCA) of differential 15-mers confirmed that the genome was successfully phased into four subgenomes based on clearly distinct patterns of both differential *k*-mers and homoeologous chromosomes (right panels). In **a** SG2 = *R. chinensis*, SG1 = *R. canina* S27 + *R. agrestis* DToL. In **b** SG1 = *R. rugosa*, SG2 = *R. canina* S27 + *R. agrestis* DToL. In **c** SG2 = *R. chinensis*, SG1 = *R. canina* S27 + *R. agrestis* DToL. In **d** SG1 = *R. rugosa*, SG2 = *R. agrestis* DToL, SG3 = *R. canina* S27. Note the clear separation of *R3* subgenomes (**d**) between the two species. *R. chinensis* chromosomes were used as outgroup for the *S1* and *S2* (**a**, **c**) subgenomes comparision, while *R. rugosa* chromosomes were used for *R3* and *R4* (**b**, **d**)

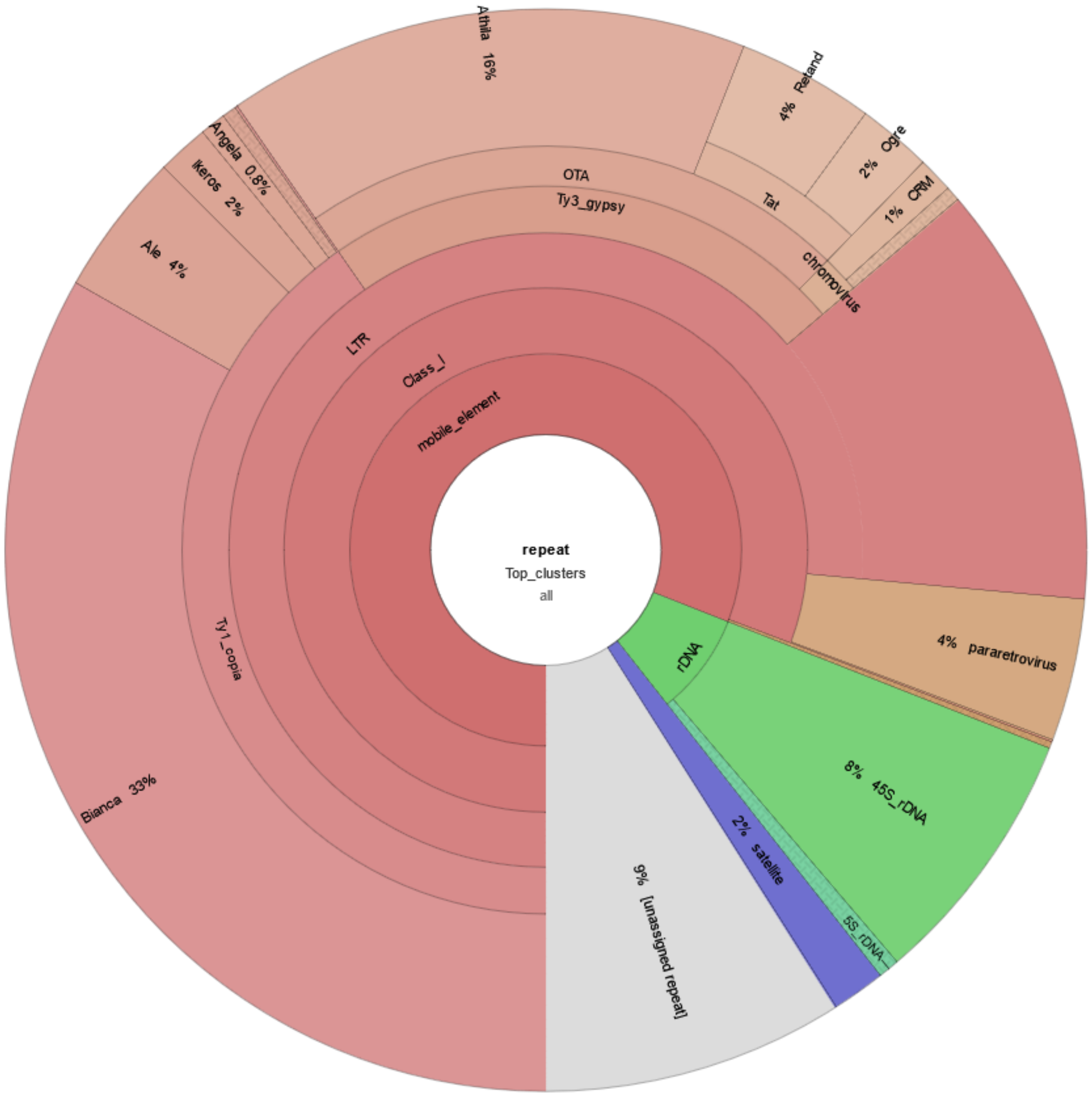

Supplementary Figure 9. Diagrammatical representation of the overal repeat composition in the *R. canina* (S27) genome determined by the RepeatExplorer2 clustering analysis.

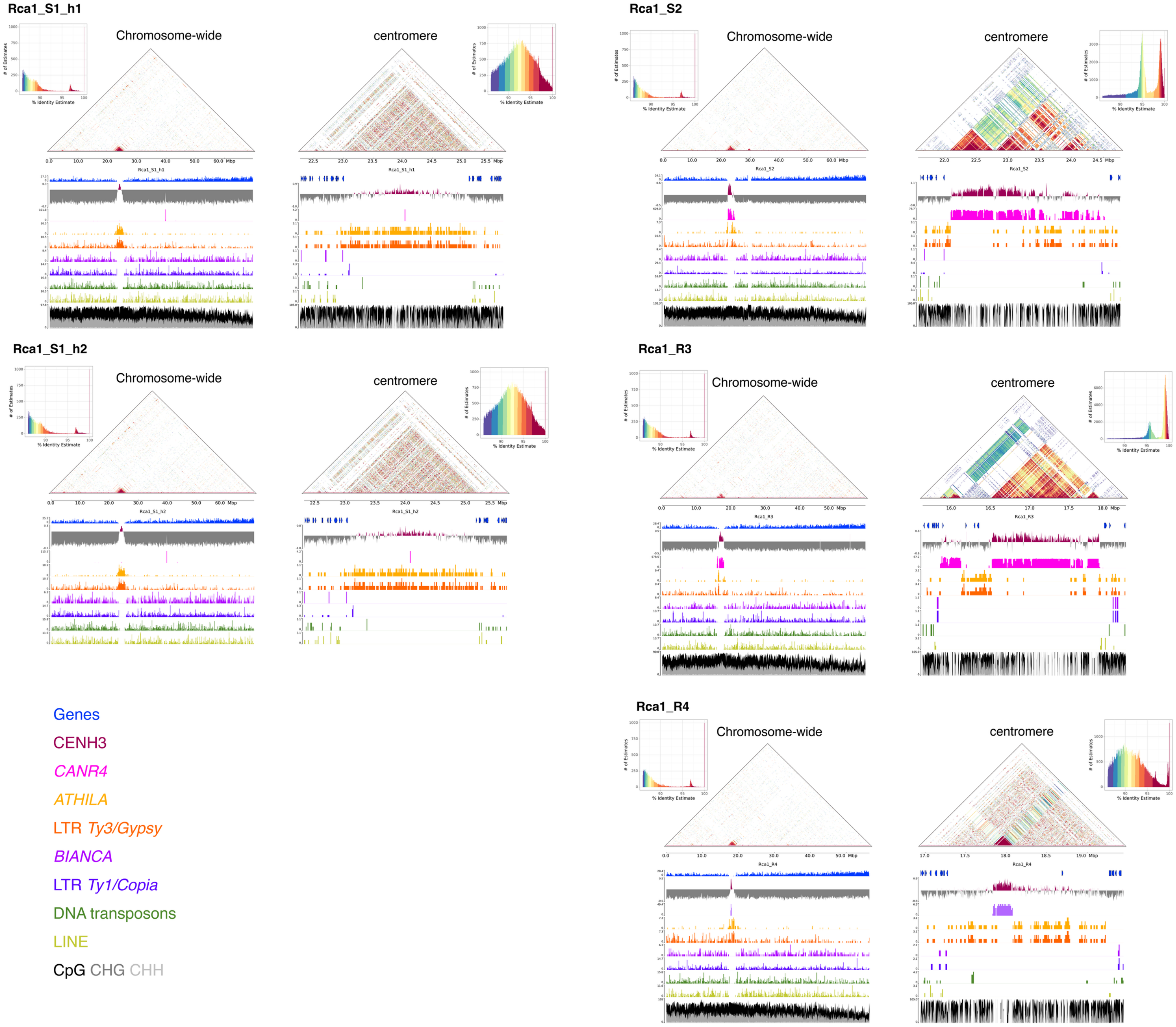
Rca1_S1_h1
Chromosome-wide
centromere
Rca1_S2
Chromosome-wide
centromere
Rca1_S1_h2
Chromosome-wide
centromere
Rca1_R3
Chromosome-wide
centromere
Rca1_R4
Chromosome-wide
centromere
Genes
CENH3
CANR4
ATHILA
LTR Ty3/Gypsy
BIANCA
LTR Ty1/Copia
DNA transposons
LINE
CpG CHG CHH

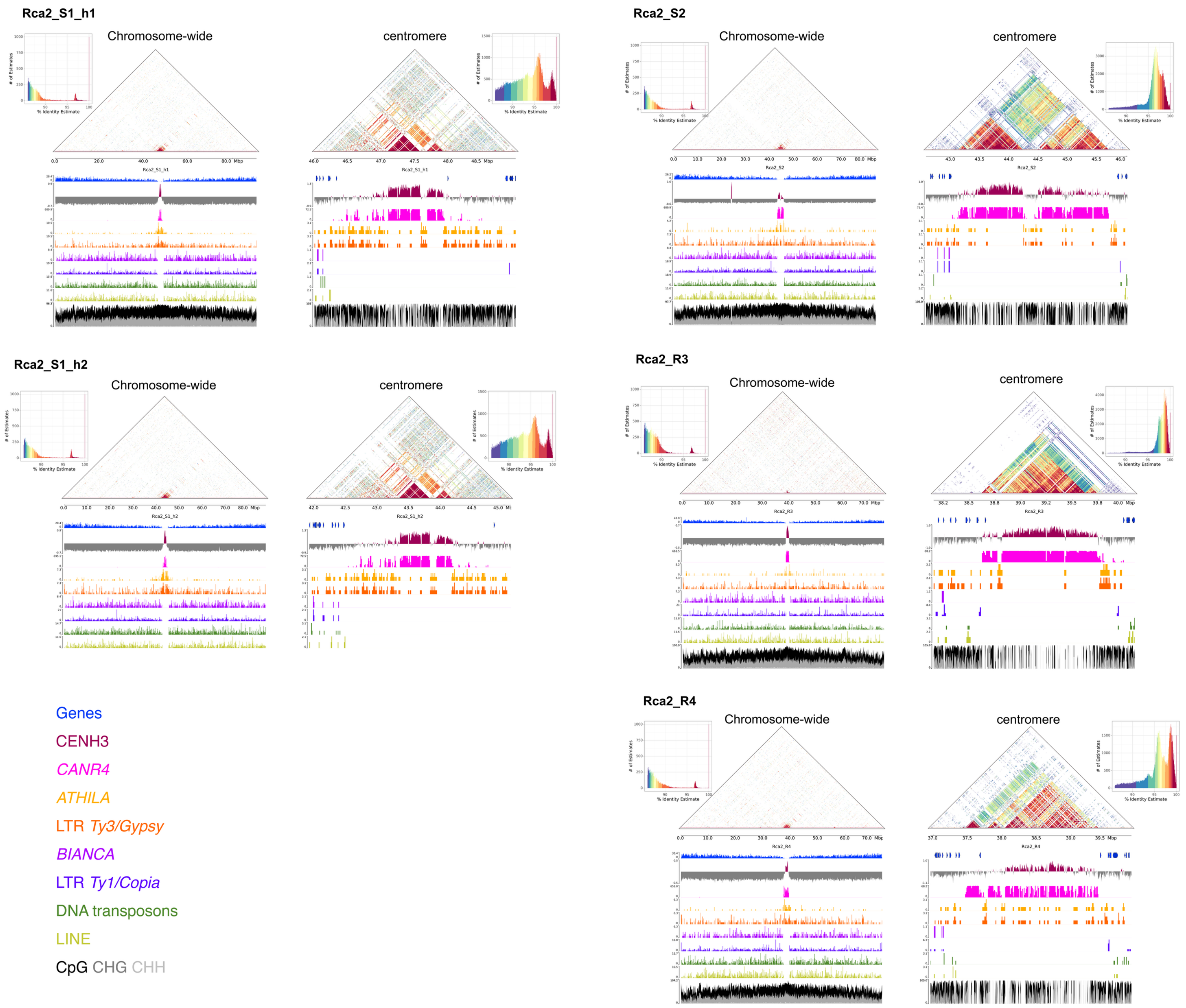
Rca2_S1_h1
Chromosome-wide
centromere
Rca2_S2
Chromosome-wide
centromere
Rca2_S1_h2
Chromosome-wide
centromere
Rca2_R3
Chromosome-wide
centromere
Rca2_R4
Chromosome-wide
centromere
# of Estimates
% Identity Estimate
Genes
CENH3
CANR4
ATHILA
LTR Ty3/Gypsy
BIANCA
LTR Ty1/Copia
DNA transposons
LINE
CpG CHG CHH

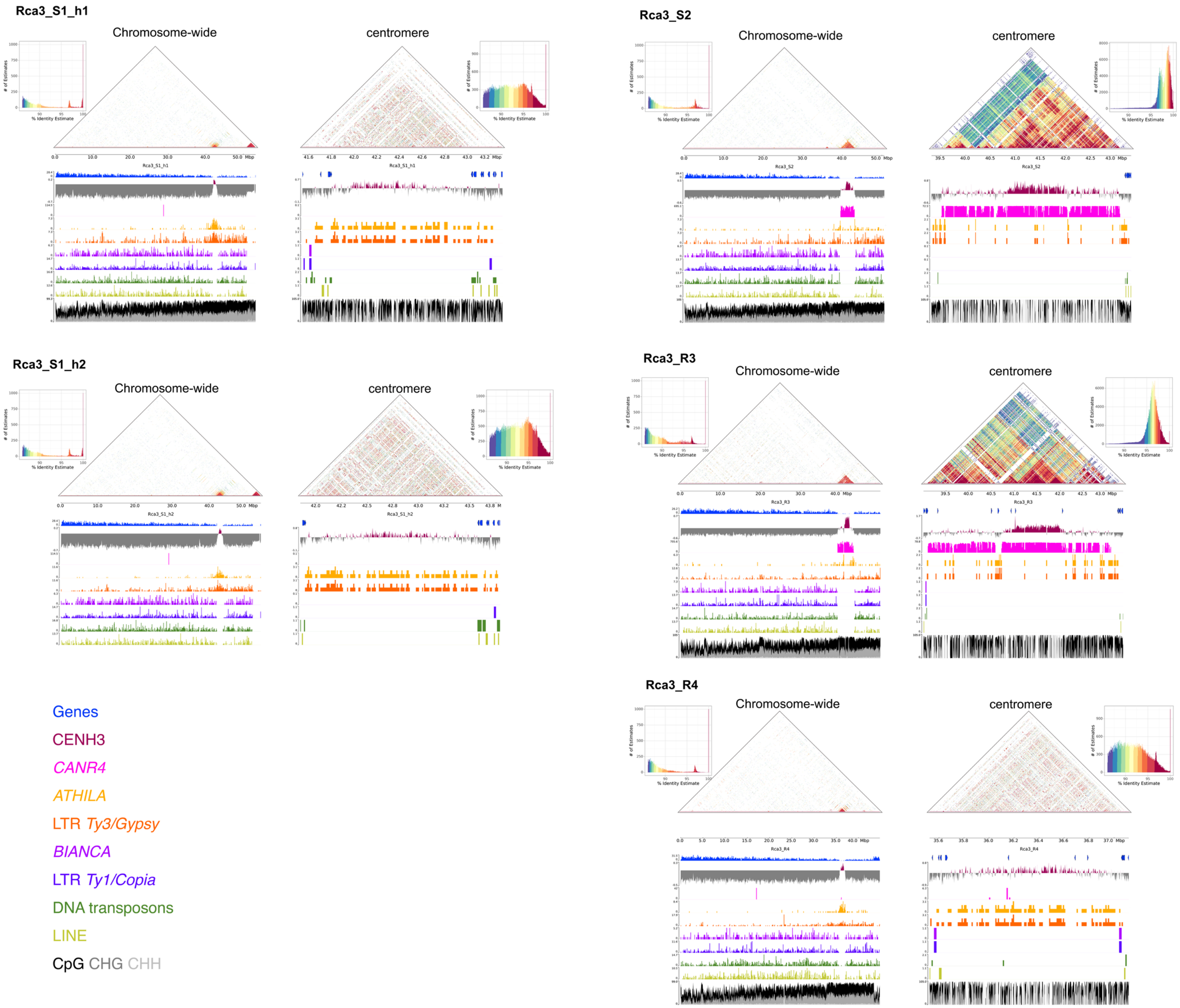
Rca3_S1_h1
Chromosome-wide
centromere
Rca3_S2
Chromosome-wide
centromere
Rca3_S1_h2
Chromosome-wide
centromere
Rca3_R3
Chromosome-wide
centromere
Rca3_R4
Chromosome-wide
centromere
% Identity Estimate
# of Estimates
Genes
CENH3
CANR4
ATHILA
LTR Ty3/Gypsy
BIANCA
LTR Ty1/Copia
DNA transposons
LINE
CpG CHG CHH

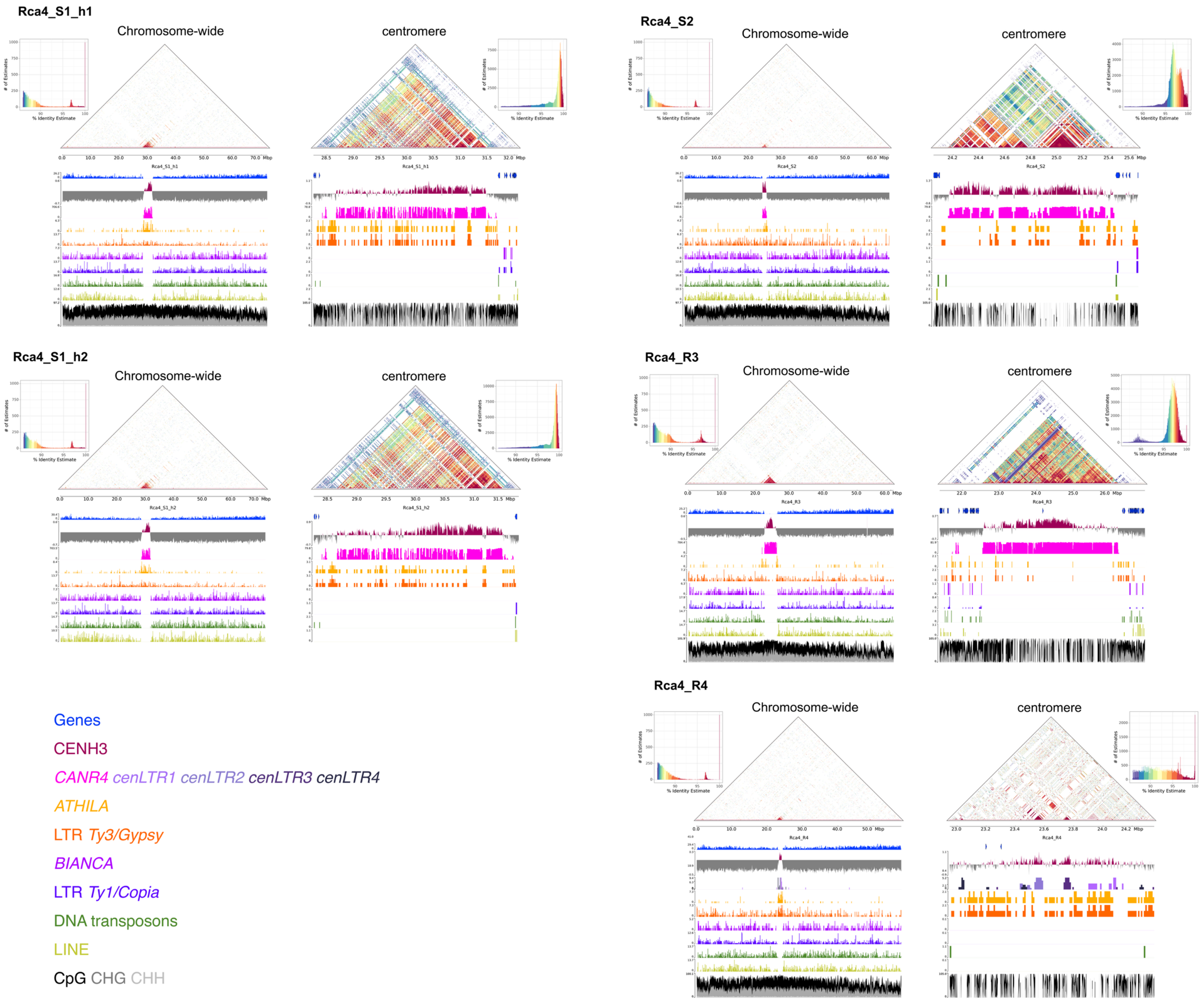
Rca4_S1_h1
Chromosome-wide
centromere
Rca4_S2
Chromosome-wide
centromere
Rca4_S1_h2
Chromosome-wide
centromere
Rca4_R3
Chromosome-wide
centromere
Rca4_R4
Chromosome-wide
centromere
Genes
CENH3
CANR4 cenLTR1 cenLTR2 cenLTR3 cenLTR4
ATHILA
LTR Ty3/Gypsy
BIANCA
LTR Ty1/Copia
DNA transposons
LINE
CpG CHG CHH

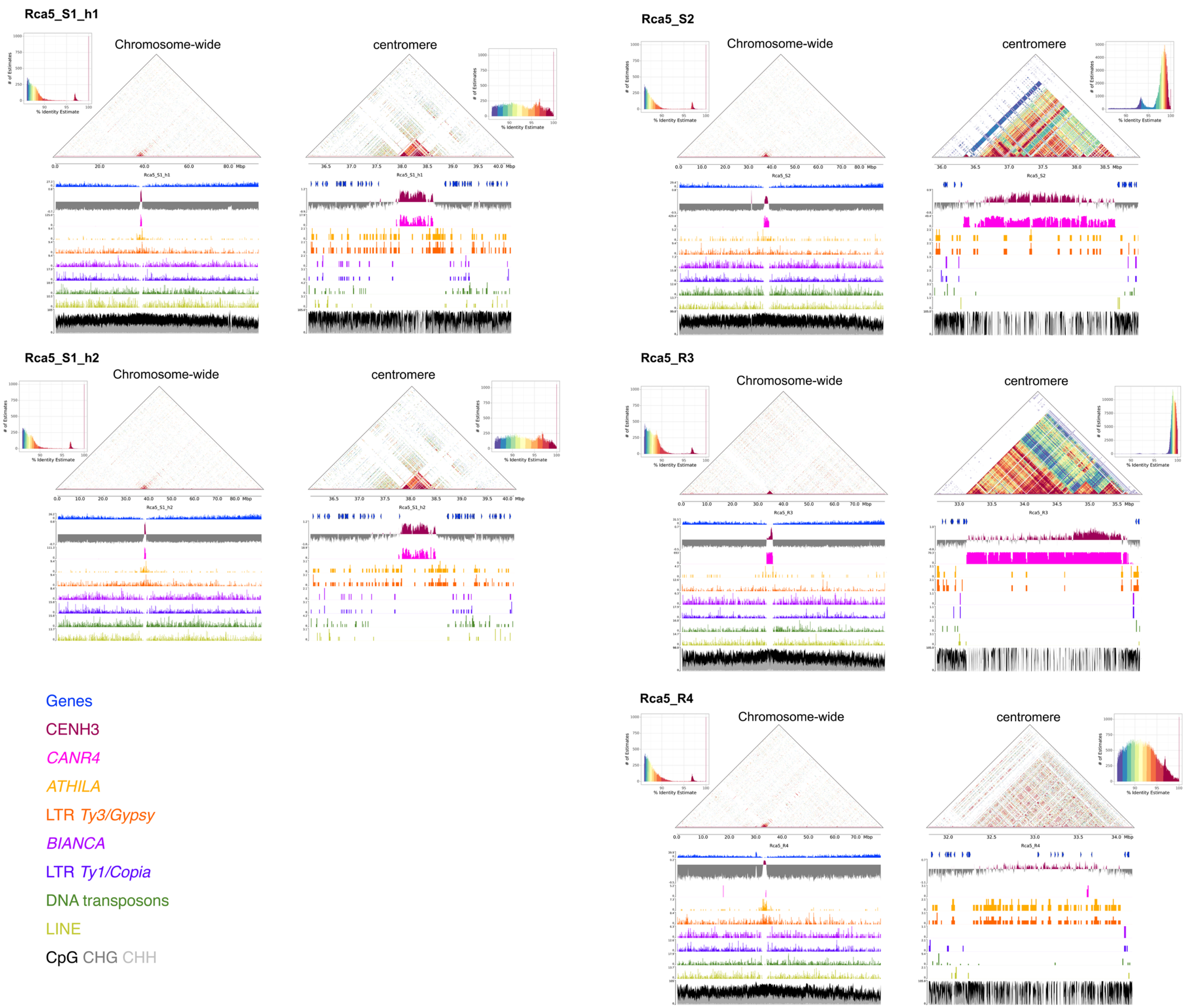
Rca5_S1_h1
Chromosome-wide
centromere
Rca5_S2
Chromosome-wide
centromere
Rca5_S1_h2
Chromosome-wide
centromere
Rca5_R3
Chromosome-wide
centromere
Rca5_R4
Chromosome-wide
centromere
Genes
CENH3
CANR4
ATHILA
LTR Ty3/Gypsy
BIANCA
LTR Ty1/Copia
DNA transposons
LINE
CpG CHG CHH

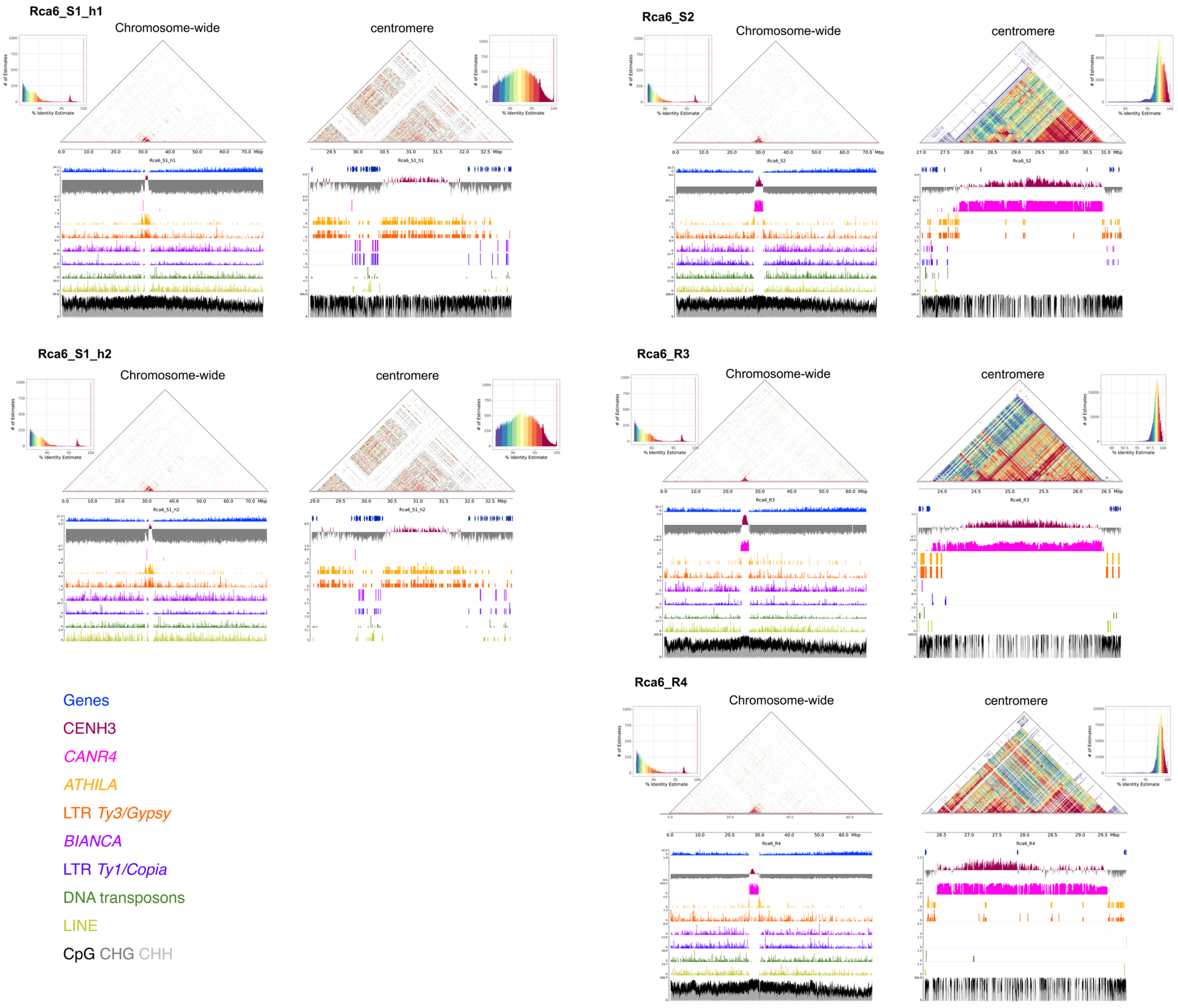

Rca6_S1_h1
Chromosome-wide
centromere
Rca6_S2
Chromosome-wide
centromere
Rca6_S1_h2
Chromosome-wide
centromere
Rca6_R3
Chromosome-wide
centromere
Rca6_R4
Chromosome-wide
centromere
Genes
CENH3
CANR4
ATHILA
LTR *Ty3/Gypsy*
BIANCA
LTR *Ty1/Copia*
DNA transposons
LINE
CpG CHG CHH

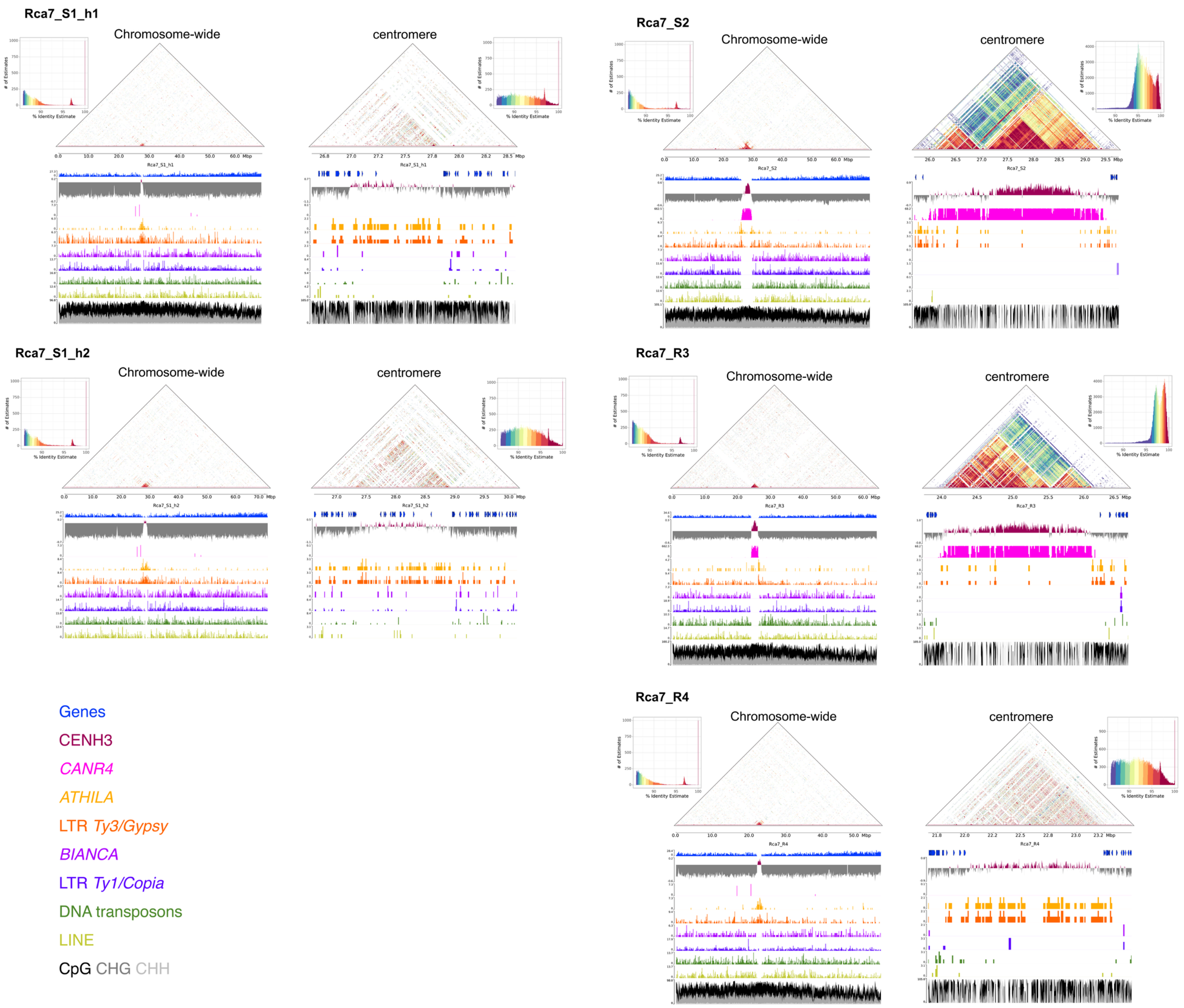
Rca7_S1_h1
Chromosome-wide
centromere
Rca7_S2
Chromosome-wide
centromere
Rca7_S1_h2
Chromosome-wide
centromere
Rca7_R3
Chromosome-wide
centromere
Rca7_R4
Chromosome-wide
centromere
Genes
CENH3
CANR4
ATHILA
LTR Ty3/Gypsy
BIANCA
LTR Ty1/Copia
DNA transposons
LINE
CpG CHG CHH

Supplementary Figure 10. The organisation and structure of *R. canina* (S27) centromeres. The sequence structure of bivalent and univalent centromeres is visualised with ModDotPlot (upper triangles). The colour intensity histograms (top right) show the number of alignments versus pairwise sequence similarity. Repeat profile densities are plotted below ModDotPlots showing the main classes of repeats identified and the respective association with CENH3. The window size used was 100 Kbp for chromosome-wide and 10 Kbp for centromre close-up plots. Please note the higher enrichment of CENH3 in *CANR4*-based centromeres compared to *ATHILA*-based ones.

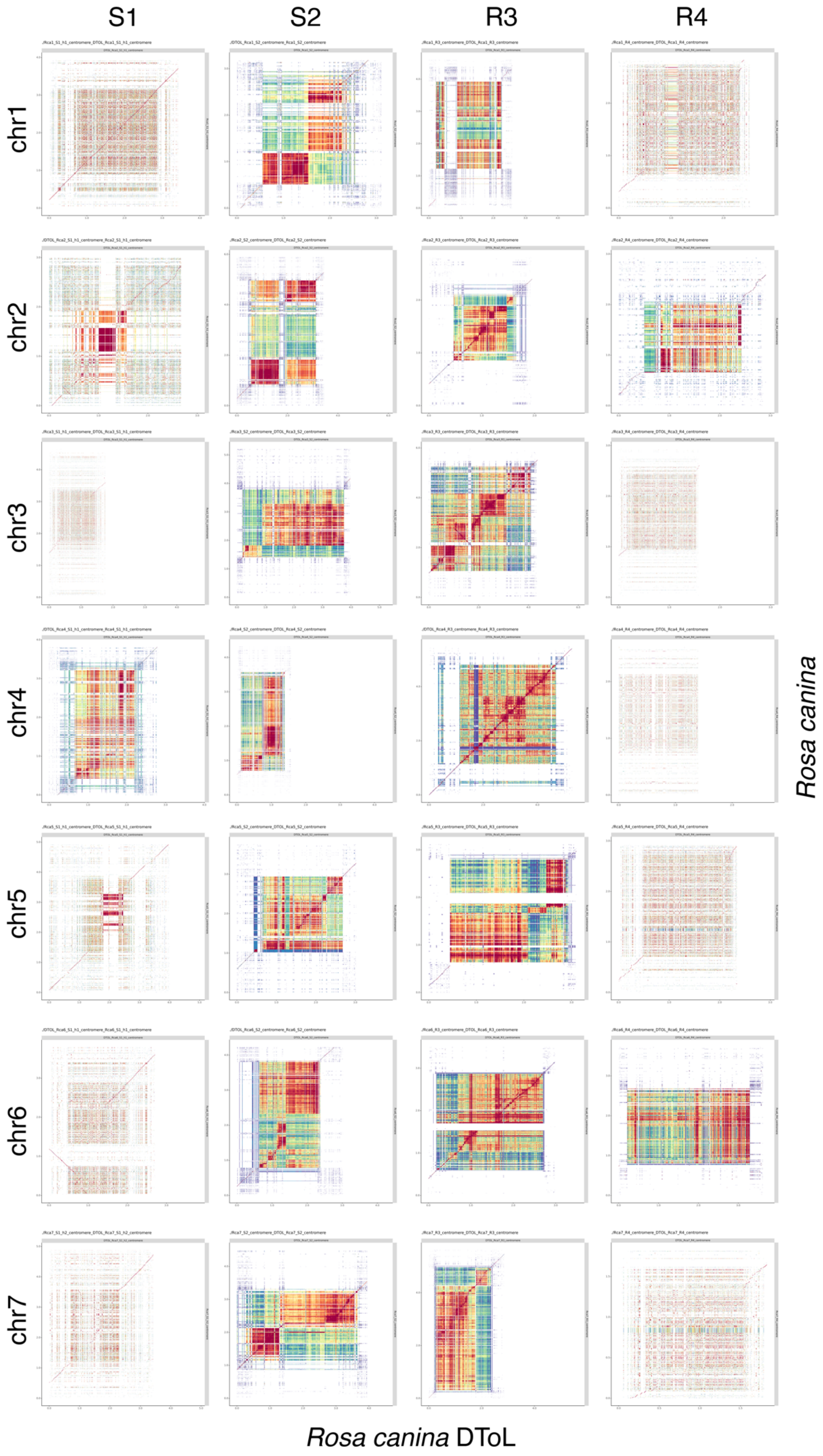

Supplementary Figure 11. Comparative dot plot structural analysis of centromeres between the genomes of the assembled *R. canina* (S27) and *R. canina* DToL using 85% similarity threshold. Faint, high-identity diagonals are visible in the comparative plot, indicating the orthologous sequences between the variable arrays.

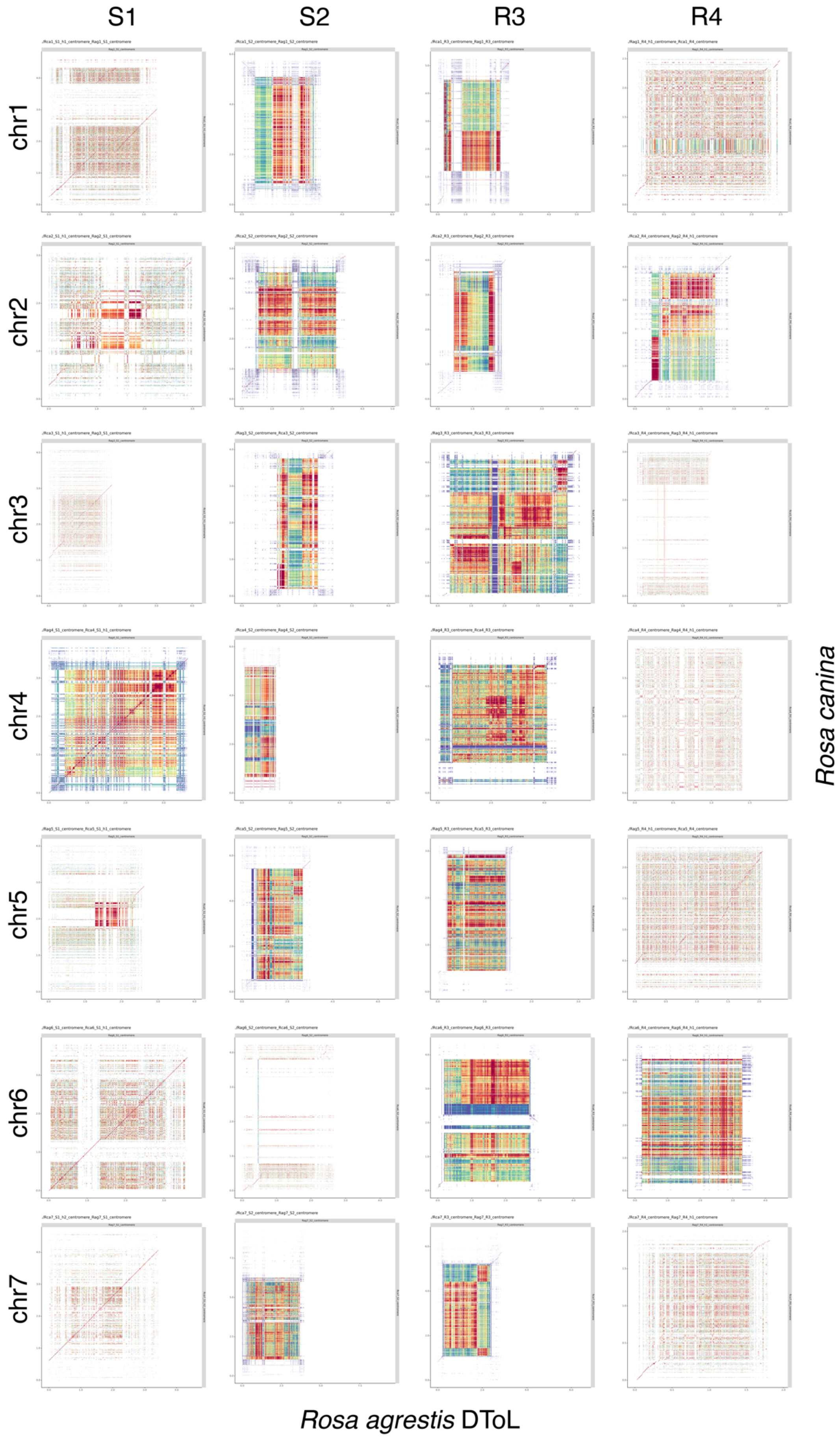

Supplementary Figure 12. Comparative dot plot structural analysis of centromeres between the genomes of the assembled *R. canina* (S27) and *R. agrestis* DToL using 85% similarity threshold. Faint, high-identity diagonals are visible in the comparative plot, indicating the orthologous sequences between the variable arrays.

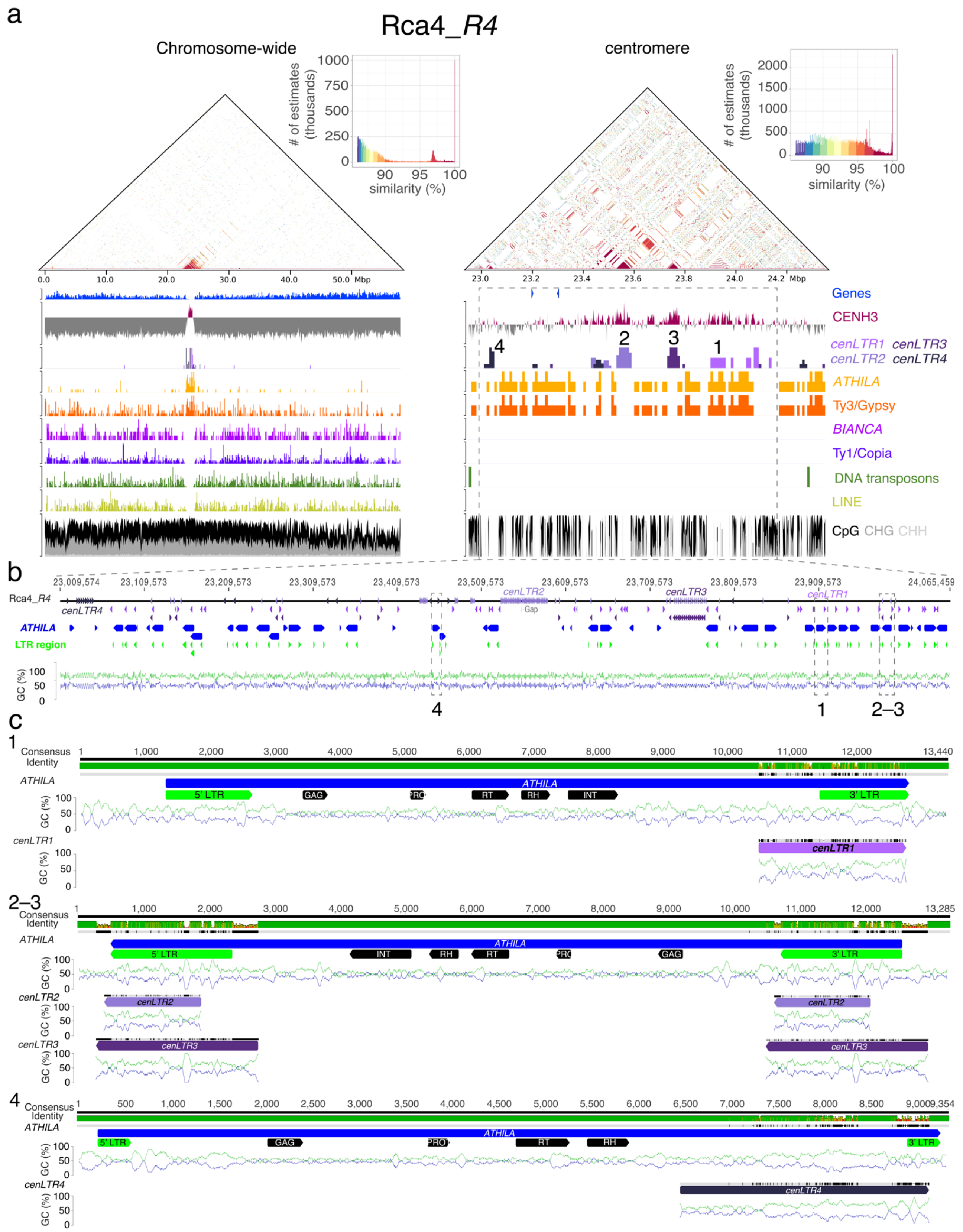

Supplementary Figure 13. Characterisation of four centromeric LTRs (*cenLTR1–4*) found in the centromere of Rca4_*R4* of *R. canina* (S27) genome. (**a**) Structural visualisation of the chromosome-wide (left) and centromere (right) close up. Please note the higher enrichment of the CENH3 ChIP-seq signal on the *cenLTR1* and *cenLTR2* tandem arrays. (**b**) Close up visualisation of the *cenLTR* arrays within the centromere of Rca4_*R4*. (**c**) Alignment of each *cenLTR1–4* consensus sequence with the LTR sequence from different *ATHILA* elements nearby showing over 75% sequence similarity. Please note that *cenLTR2* and *cenLTR3* share very high similarity between each other.

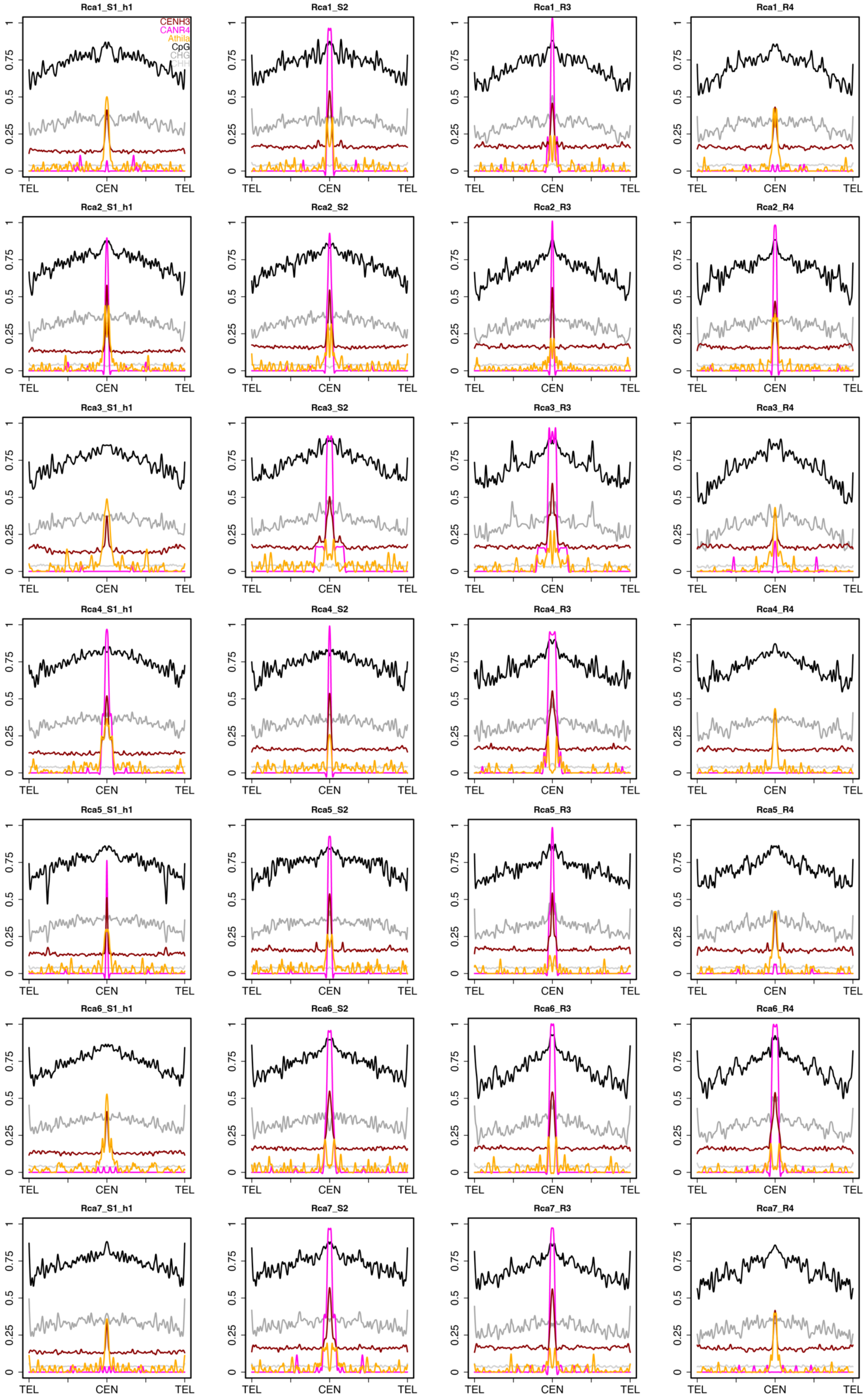
Rca1_S1_h1
Rca1_S2
Rca1_R3
Rca1_R4
Rca2_S1_h1
Rca2_S2
Rca2_R3
Rca2_R4
Rca3_S1_h1
Rca3_S2
Rca3_R3
Rca3_R4
Rca4_S1_h1
Rca4_S2
Rca4_R3
Rca4_R4
Rca5_S1_h1
Rca5_S2
Rca5_R3
Rca5_R4
Rca6_S1_h1
Rca6_S2
Rca6_R3
Rca6_R4
Rca7_S1_h1
Rca7_S2
Rca7_R3
Rca7_R4
CENH3
CANR4
Athila
CpG
CHG
CHH
0
0.25
0.5
0.75
1
TEL
CEN
TEL

Supplementary Figure 14. Metaplot of CENH3 enrichment, DNA methylation, and centromeric elements—*ATHILA* and *CANR4* density—on each chromosome. CENH3 enrichment marked in dark red was calculated by CENH3 ChIP-seq (log_2CENH3/H3) signal normalised by coverage. DNA methylations have three contexts with CpG marked in black, CHG maked in dark grey, and CHH marked in light grey. *ATHILA* (yolk yellow) and *CANR4* (magenta) were presented by their density. All signals were calculated in 50 kb adjacent windows. All chromosome coordinates were scaled based on their distance to centromere against the distance of centromere (CEN) to telomere (TEL). Centromere position (CEN) was defined by where maximum CENH3 enrichment was located. All signal values (y-axis) were scaled from 0 to 1 by global minimum to global maximum except for DNA methylations, which retained the original percentage values. The p- and q-arm values were averaged and mirrored.

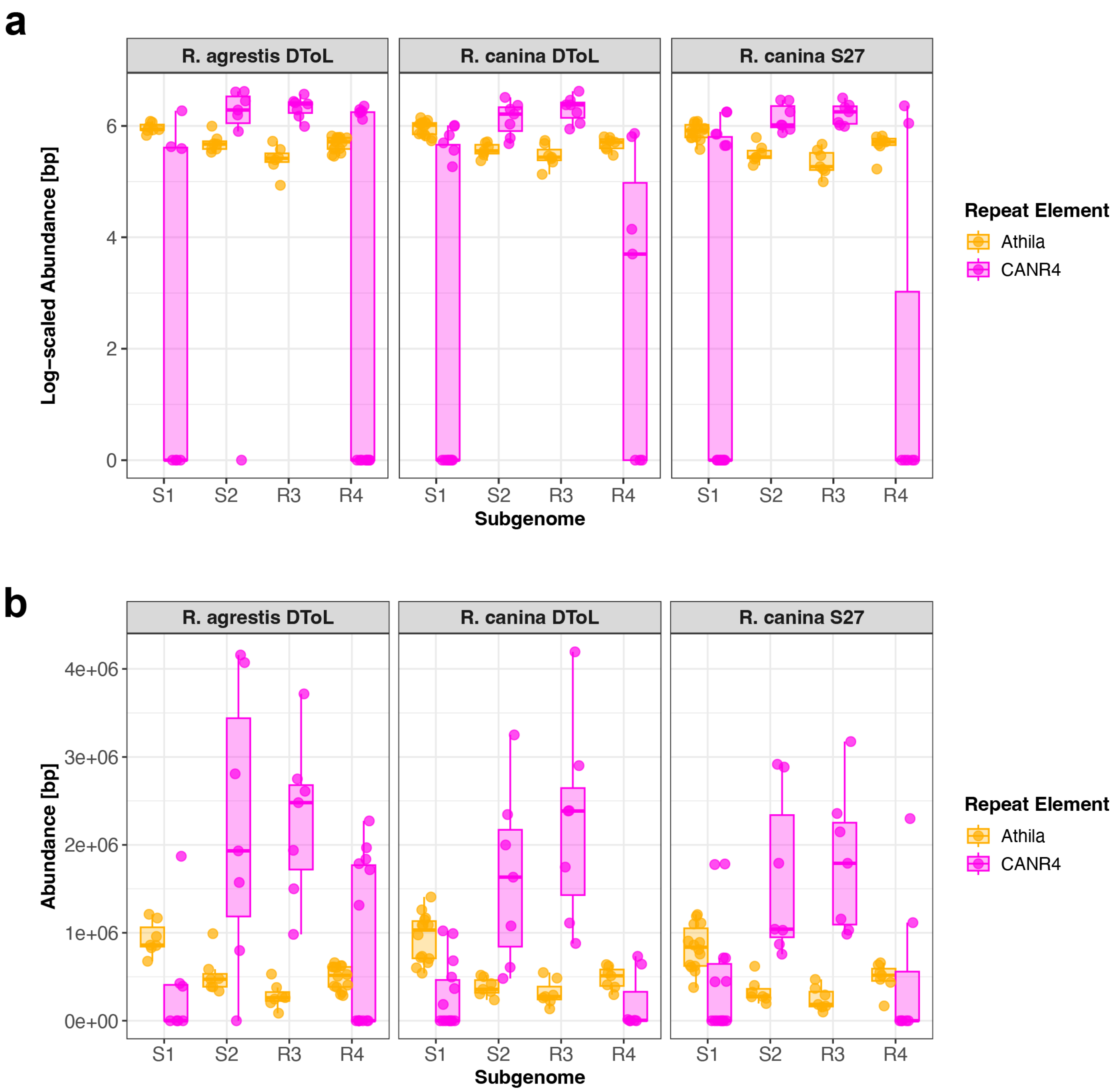

Supplementary Figure 15. Total centromeric abundance of *CANR4* and *ATHILA* repeats on each subgenome across *R. canina* (S27), *R. canina* DToL and *R. agrestis* DToL. (**a**) log scale and (**b**) without log scale abundance. Centromere length was defined by the total interval of CENH3 peaks called within centromeric regions in *R. canina* 27 and respective regions from DToL samples *R. canina* and *R. agrestis*. Each dot represents one biologically independent chromosome (n = 7 per subgenome per genome), plotted per subgenome (S1, S2, R3, R4). Colors indicate the repeat element type. The MCMCglmm statistics is available in Supplementary Dataset 13, source data in Supplementary Dataset 16.

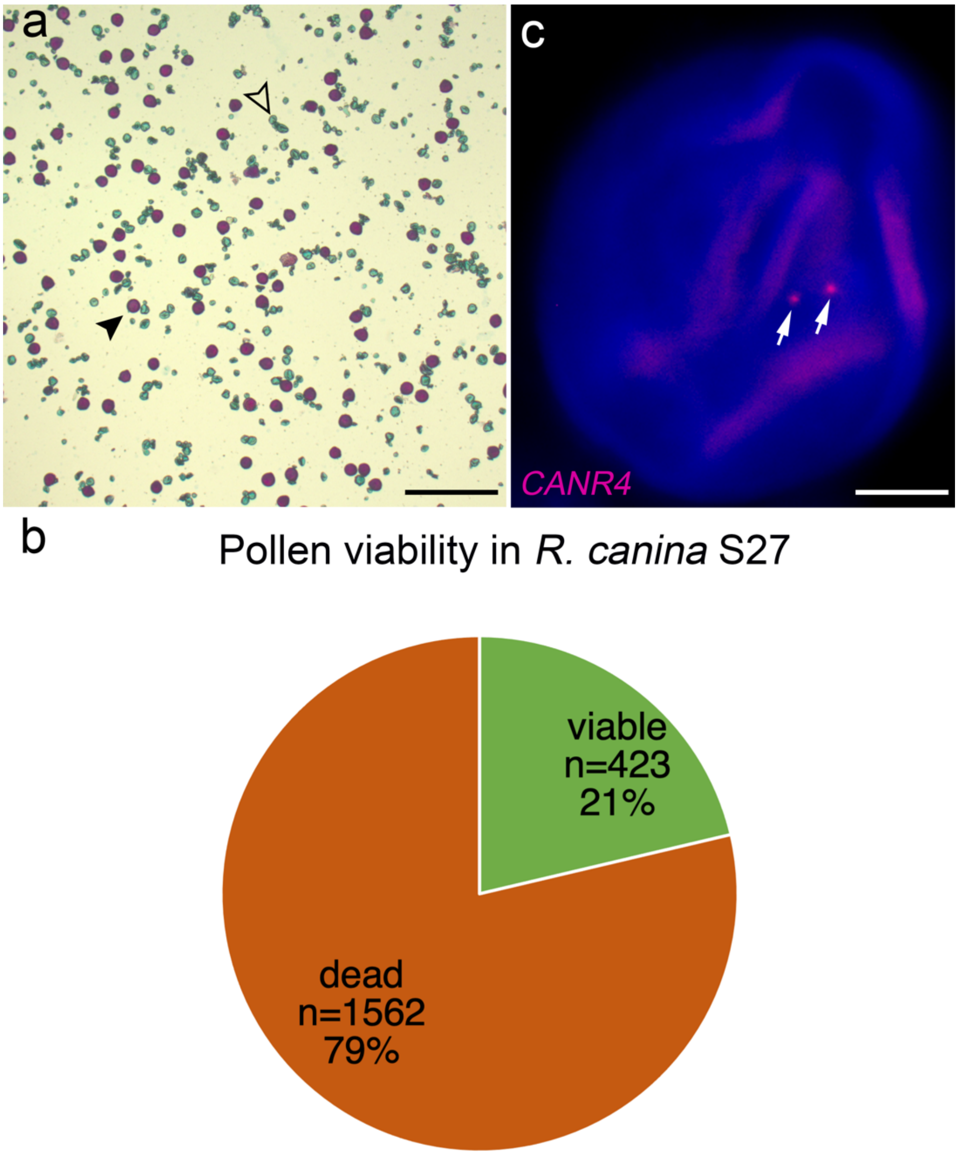

Supplementary Figure 16. Pollen viability screening in *R. canina* (S27). (**a**) Viability of pollen grains estimated by Alexander staining. Viable pollen grains are dark purple coloured (filled arrowhead), while sterile and deformed pollen grains are seen as mostly translucent (outlined arrowhead). Scale bar = 100 µm. (**b**) Pollen viability counts based on the Alexander staining assay. Scale bar = 10 µm. (**c**) Young pollen grain showing only two signals of the *in situ* hybridised *CANR4* probe, agreeing with the presence of only *S1* chromosomes in viable pollen.

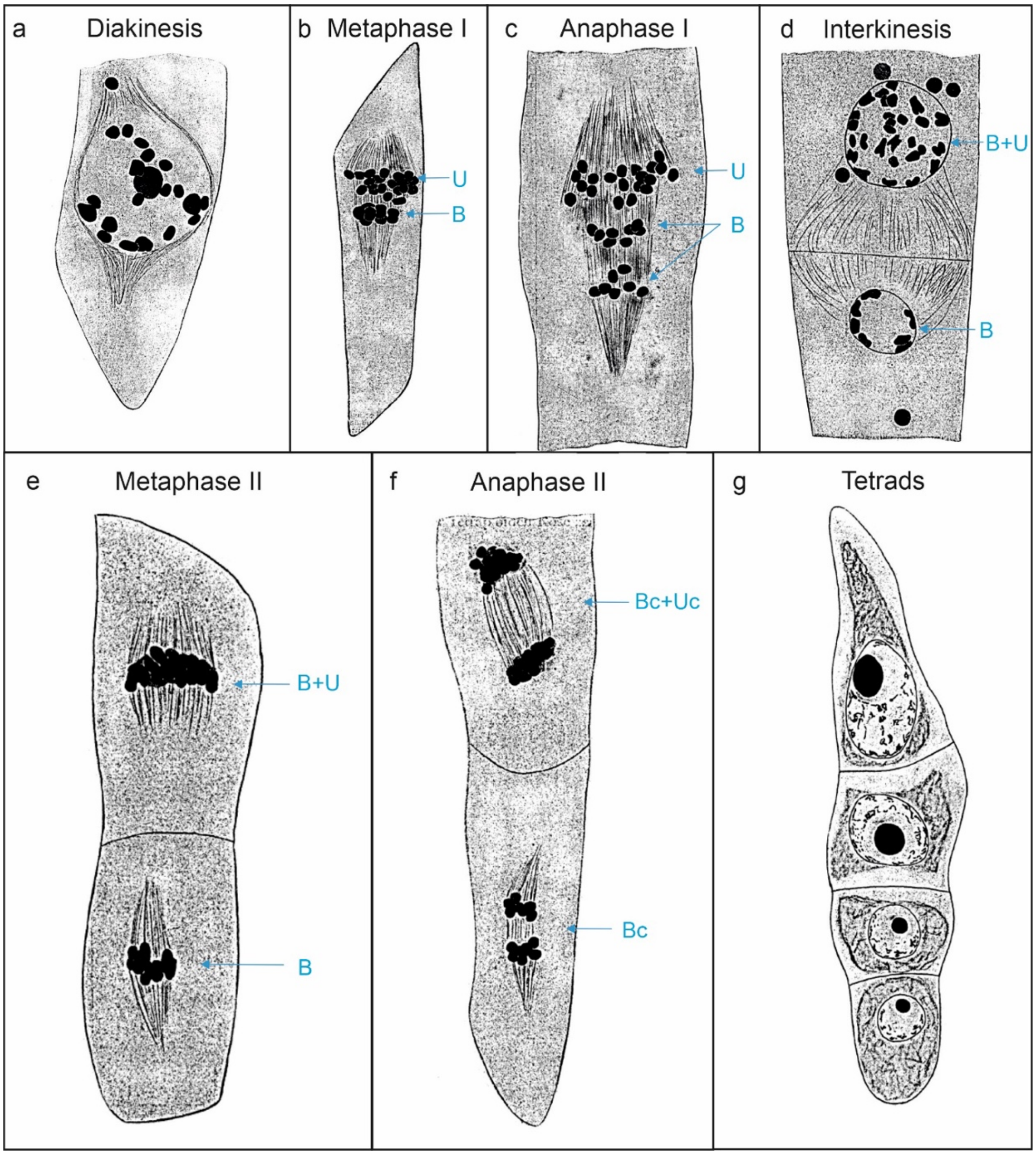

Supplementary Figure 17. **Course of female meiosis of pentaploid dogroses compiled from drawings of Täckholm (1922).** Täckholm's original descriptions are shown in quotation marks and were translated from German to English, the original numbering of figures is given in brackets, current taxonomy of species is given in rectangular brackets. Bivalent and univalent are marked by us in blue. B = bivalent-forming chromosomes, U = univalent-forming chromosomes, Bc = chromatids of bivalent-forming chromosomes, Uc = chromatids of univalent-forming chromosomes. Note, that drawings did not contain scale bars. (**a**) (Fig. 39b; p. 213) *R. rubiginosa,* transitional stage between diakinesis and metaphase; the two largest bodies represent nucleoli; 6 bivalent and 23 univalent chromosomes. (**b**) (Fig. 40a; p. 214) [*R. caesia*], "Heterotypic metaphase; the 7 bivalents form a small plate and all 21 single chromosomes are located on the micropylar side of the same plate". (**c**) (Fig. 40b; p. 214) [hybrid with *R. glauca*], "Heterotypic anaphase; the bivalents are separated; 21 univelants are located above the bialents". (**d**) (Fig. 42a; p. 216) "Interkinesis. [*R. balsamica* × *R. balsamica*]. In the micropylar nucleus 28, in the chalazal nucleus 7 chromosomes; several nucleoli in the plasma". (**e**) (Fig. 43b; p. 217) "Homotypic metaphase, [*R.* cf. *caesia*] 7 chromosomes are visible in the chalazal spindle". (**f**) (Fig. 44a; p. 218) [*R.* cf. *caesia*] "Homotypic anaphase; two groups of each 7 bivalent-forming chromosomes in the chalazal dyad". (**g**) (Fig. 44; p. 218*)* [*R. caesia*]*,* "Tetrad, consisting of two large micropylar and two small chalazal macrospores". Note, that Täckholm added to the text (p. 219): "As far as my observations go, the embryo sacs originate from the larger micropylar tetrad cells".

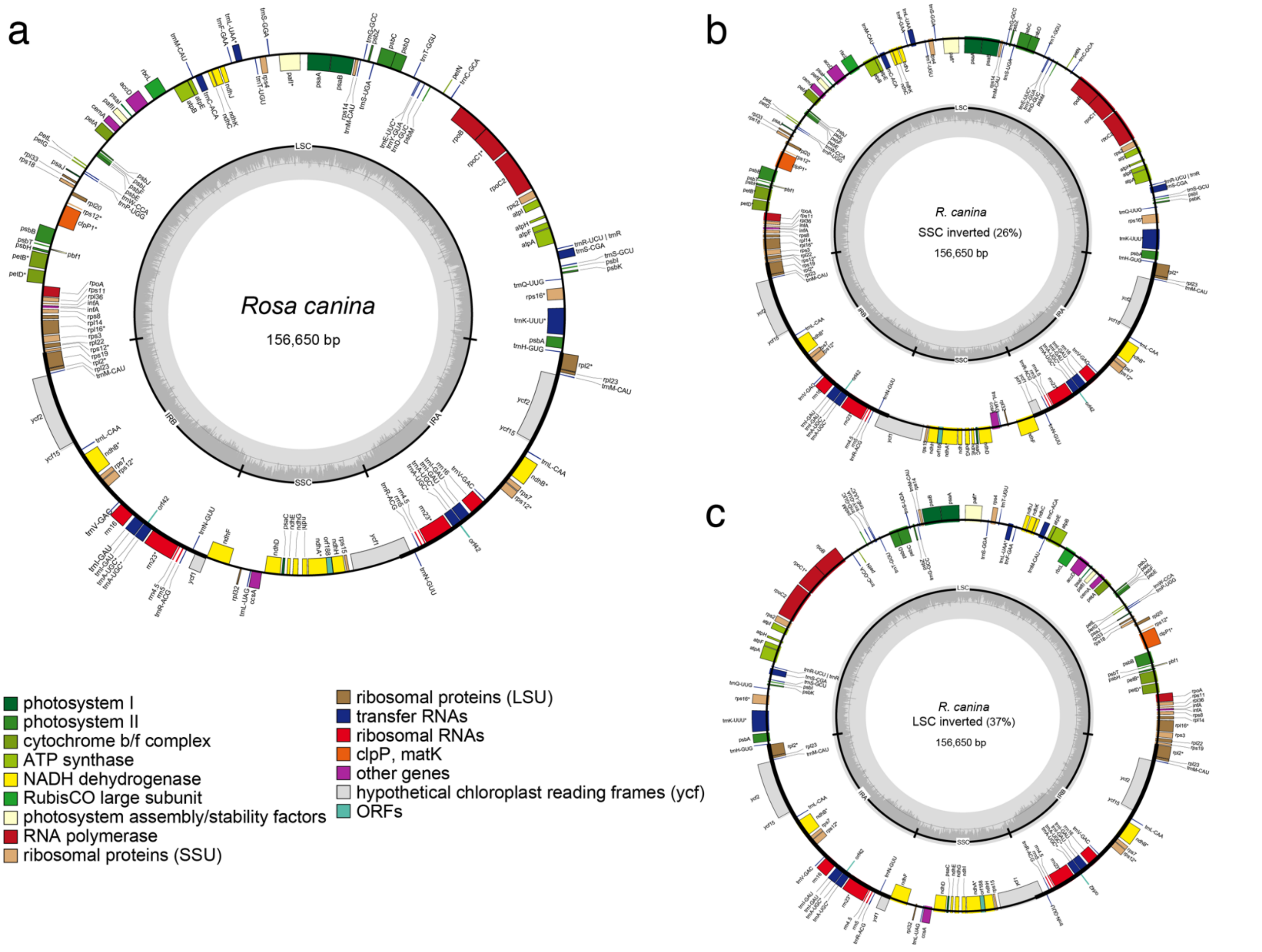

Supplementary Figure 18. Gene maps of the chloroplast genome of *R. canina* (S27) differing in the orientation of their single copy units. (**a**) The most abundant isomer detected with ca. 63% of all reads having LSC in this orientation. About 74% of all reads have the SSC and IR in shown direction. (**b**) An inverted SSc was detected in 26% of the reads. (**c**) The LSC was found to be inverted in 37% of the reads.

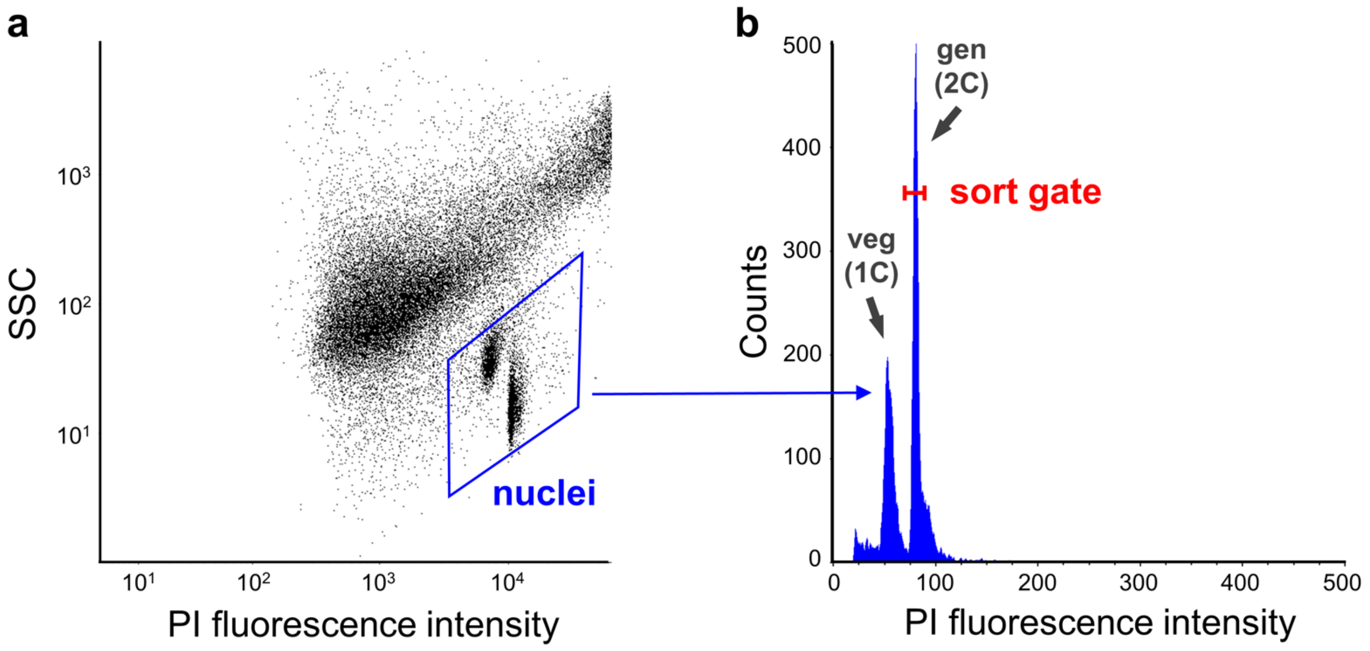

Supplementary Figure 19. Gating strategy to isolate generative nuclei of *R. canina* as presented in **Fig. 2a**. (a) After identifying the nuclear populations in a dotplot displaying the PI fluorescence signal (log-scale) versus side scatter signal (SSC, log-scale), (b) a sort gate was defined in the corresponding fluorescence intensity (lin-scale) histogram. A similar strategy as shown in (a) was applied to identify the nuclei in isolates of nutlets from dogroses as shown in **Fig. 2d**.

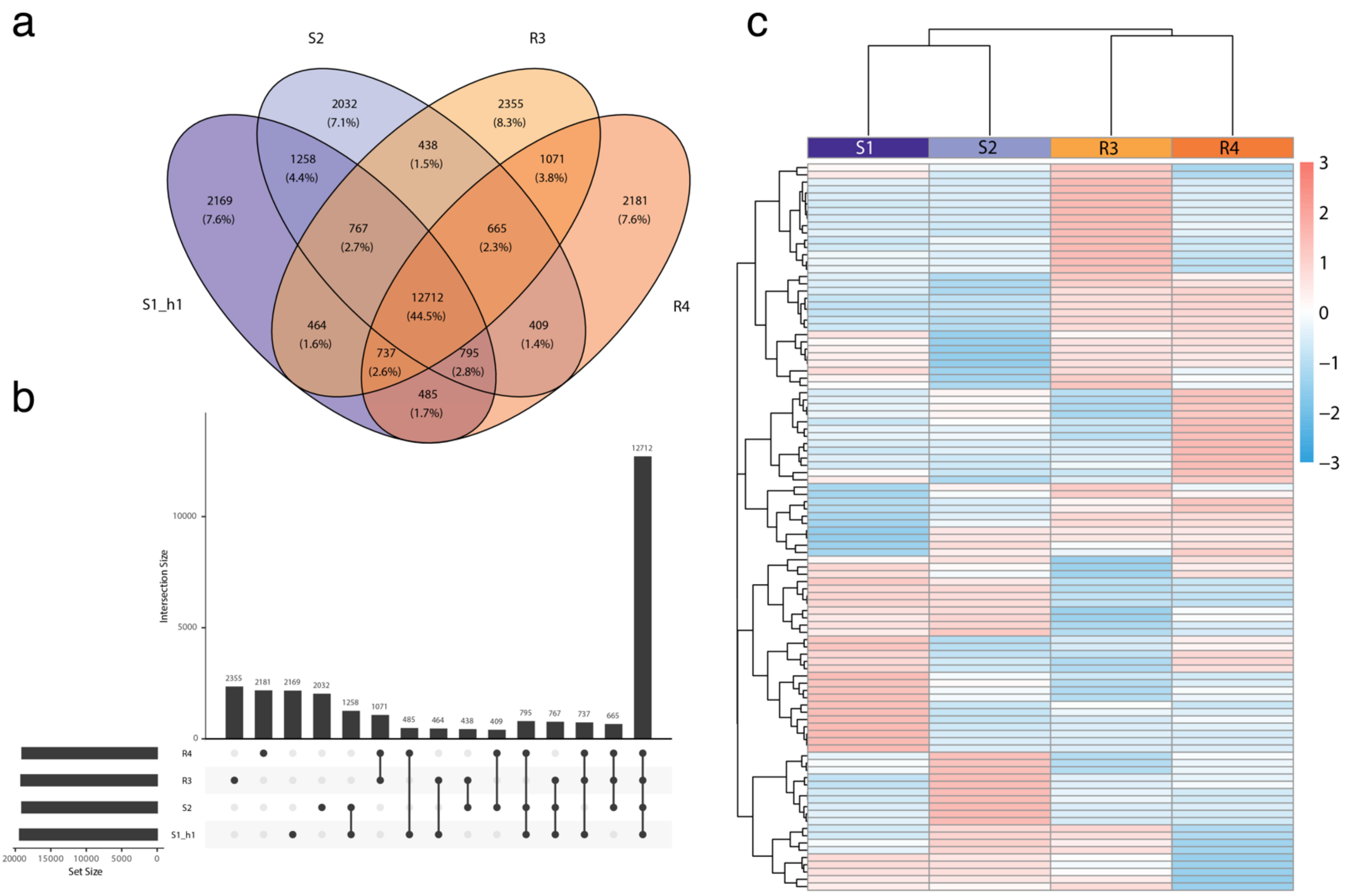

Supplementary Figure 20. Gene number comparison among the subgenomes of *R. canina* (S27) and clustering of top 100 highly expressed genes. (**a**) Venn plot of function-annotated genes. (**b**) Upset plot of function-annotated genes showing the intersected gene number of different subgenome combinations. (**c**) Heatmap and clustering of top 100 expressed genes in four subgenomes. The color indicates the fold change of up- and down-regulation of genes.
