# Supplementary material for: Bimodal centromeres in pentaploid dogroses shed light on their unique meiosis
Source: Nature. 2025 Jun 18;643(8070):148–57. doi: 10.1038/s41586-025-09171-z (PMC12222009; doi:10.1038/s41586-025-09171-z)
Supplement: Supplementary file 2 — Reporting Summary [file 41586_2025_9171_MOESM2_ESM.pdf]

nature portfolio

Corresponding author(s): André Marques
Last updated by author(s): May 5, 2025

Reporting Summary

Nature Portfolio wishes to improve the reproducibility of the work that we publish. This form provides structure for consistency and transparency in reporting. For further information on Nature Portfolio policies, see our Editorial Policies and the Editorial Policy Checklist.

Statistics

For all statistical analyses, confirm that the following items are present in the figure legend, table legend, main text, or Methods section.

n/a	Confirmed	
☐	☒	The exact sample size (*n*) for each experimental group/condition, given as a discrete number and unit of measurement
☐	☒	A statement on whether measurements were taken from distinct samples or whether the same sample was measured repeatedly
☐	☒	The statistical test(s) used AND whether they are one- or two-sided<br>*Only common tests should be described solely by name; describe more complex techniques in the Methods section.*
☒	☐	A description of all covariates tested
☐	☒	A description of any assumptions or corrections, such as tests of normality and adjustment for multiple comparisons
☐	☒	A full description of the statistical parameters including central tendency (e.g. means) or other basic estimates (e.g. regression coefficient) AND variation (e.g. standard deviation) or associated estimates of uncertainty (e.g. confidence intervals)
☒	☐	For null hypothesis testing, the test statistic (e.g. *F*, *t*, *r*) with confidence intervals, effect sizes, degrees of freedom and *P* value noted<br>*Give P values as exact values whenever suitable.*
☒	☐	For Bayesian analysis, information on the choice of priors and Markov chain Monte Carlo settings
☒	☐	For hierarchical and complex designs, identification of the appropriate level for tests and full reporting of outcomes
☒	☐	Estimates of effect sizes (e.g. Cohen's *d*, Pearson's *r*), indicating how they were calculated

Our web collection on statistics for biologists contains articles on many of the points above.

Software and code

Policy information about availability of computer code

Data collection

HiFi and Omni-C reads were obtained through own sequencing at the Max Planck Institute for Plant Breeding Research, Cologne, Germany. Images were analyzed using the ZEN software (Carl Zeiss GmbH) and the ZENBlack software (Carl Zeiss GmbH).

Data analysis

Available open source tools used in this study were:

Bedtools (v2.29.0)
bcftools 1.15.1
Bismark (v0.23.0)
BLAST 2.13.0+
Bowtie2 (2.5.4)
BUSCO (v5.1.2)
BWA (0.7.17)
CoGe (v7)
Cutadapt (v4.7)
DANTE_LTR (v0.3.5.2)
Deeptools (v3.5.1)
DESeq2 (1.46.0)
Dotter (v0.13.1)
EMBOSS (v2024.0419.155605)
findGSE_v1.94.R
GATK v4.1.9.0

GenomeScope2
Geneious (v2023.0.1)
GENESPACE (v1.3.1)
Helixer (0.3.4)
Hisat (2.2.1)
Hifiasm (0.19.8-r603)
htseq-count (v2.0.1)
IQ-TREE (2.4.0)
Jellyfish (v2.3.1)
Juicer (v1.6)
Kaks Calculator (v3)
MACS3 (3.0.1)
MAFFT (7.526)
minimap2 (v2.26)
ModDotPlot (v0.8.2)
PAML v4.10.6
plotsr (v0.5.3)
pyGenomeTracks (v3.8)
QUAST (v5.2.0)
RepeatExplorer2 (v2.3.7)
REXdb (v1.0)
SALSA2 (v2.3)
Samtools (v1.9)
StainedGlass (v0.6)
syri (v1.5.3)
VCFtools (0.1.16)
ZEN blue (3.1)

For manuscripts utilizing custom algorithms or software that are central to the research but not yet described in published literature, software must be made available to editors and reviewers. We strongly encourage code deposition in a community repository (e.g. GitHub). See the Nature Portfolio guidelines for submitting code & software for further information.

Data

Policy information about availability of data

All manuscripts must include a data availability statement. This statement should provide the following information, where applicable:

- Accession codes, unique identifiers, or web links for publicly available datasets
- A description of any restrictions on data availability
- For clinical datasets or third party data, please ensure that the statement adheres to our policy

All raw sequencing data (HiFi, Hi-C, RNA, CENH3-ChIP, DNA methylation, SCO of pollens) and genome assembly of Rosa canina S27 isolate are available under NCBI BioProject: PRJNA1111045. The chloroplast genome of Rosa canina S27 isolate available under GeneBank accession number PV550499. Raw sequencing data of Rosa canina DToL and Rosa agrestis DToL are available from Darwin Tree of Life (DToL) data portal. The corresponding NCBI BioProject accession numbers are PRJEB79802 and PRJEB79880, respectively. Genome assemblies, the sample-specific SCO reference sequences, variant calling format files, annotations and alignments presented in this work are also made available for download at DRYAD: https://doi.org/10.5061/dryad.cc2fqz6fh. The REXdb database Viridiplantae v.3.0 [http://repeatexplorer.org/?page_id=918] is publicly available. All other data needed to evaluate the conclusions in the paper are provided in the paper and/or the supplemental information.

Research involving human participants, their data, or biological material

Policy information about studies with human participants or human data. See also policy information about sex, gender (identity/presentation), and sexual orientation and race, ethnicity and racism.

Reporting on sex and gender	n/a
Reporting on race, ethnicity, or other socially relevant groupings	n/a
Population characteristics	n/a
Recruitment	n/a
Ethics oversight	n/a

Note that full information on the approval of the study protocol must also be provided in the manuscript.

Field-specific reporting

Please select the one below that is the best fit for your research. If you are not sure, read the appropriate sections before making your selection.

☒ Life sciences ☐ Behavioural & social sciences ☐ Ecological, evolutionary & environmental sciences

For a reference copy of the document with all sections, see nature.com/documents/nr-reporting-summary-flat.pdf

Life sciences study design

All studies must disclose on these points even when the disclosure is negative.

Sample size	Sample-size calculation was performed based on assessment of the literature in the field, our own experience from previous studies and requirement for corresponding protocols. For Immunocytochemistry and in situ hybridisation analyes sample size was based on the number of cells obtained. The size of the sample used was performed according to the requirements for each protocol. For cytological analysis, different roots and anthers were collected and analysed to confirm the reproducibility of the results. For sequencing, sufficient coverage (>20x) was used to assemble and scaffold the R. canina genome. The sample size used for all experiments provided sufficient resolving power.
Data exclusions	No data was excluded form the analysis.
Replication	Cytogenetic analyses were performed on several cells, using the best superposition for the final figure. Experiments were independently repeated at least ten times with similar results, in order to track all meiotic stages.
Randomization	A randomization is not relevant for this study because no genotype or treatment were compared with each other. However, the tissues for cytogenetic and ChIPseq experiments were randomly collected from different plant individuals grown under the same condition in a greenhouse.
Blinding	The experiments were performed without knowing the final results.

Reporting for specific materials, systems and methods

We require information from authors about some types of materials, experimental systems and methods used in many studies. Here, indicate whether each material, system or method listed is relevant to your study. If you are not sure if a list item applies to your research, read the appropriate section before selecting a response.

Materials & experimental systems

n/a	Involved in the study	
☐	☒	Antibodies
☒	☐	Eukaryotic cell lines
☒	☐	Palaeontology and archaeology
☒	☐	Animals and other organisms
☒	☐	Clinical data
☒	☐	Dual use research of concern
☐	☒	Plants

Methods

n/a	Involved in the study	
☐	☒	ChIP-seq
☐	☒	Flow cytometry
☒	☐	MRI-based neuroimaging

Antibodies

Antibodies used	Customized Rosa canina-specific antibodies generated in this study:<br>rabbit anti-CENH3 (AB016310, LifeTein, generated in this study; dilution 1:500)<br>Commercially available antibodies:<br>mouse anti-alpha Tubulin (Sigma-Aldrich, St. Louis, MO; catalogue number T6199, Clone: MABI 0301, dilution 1:200)<br>rabbit anti-Histone H3 (Active Motif, cat. No. 39064, ChIP only, 1µg used)<br>Previously designed antibodies:<br>rabbit anti-KNL1 (AB015677-3; GenScript, Piscataway, NJ, USA; Oliveira et al. 2024, dilution 1:500)
Validation	Newly validated antibodies:<br>anti-Rosa canina CENH3 antibody was generated by the company LifeTein and validated by peptide ELISA tests and ChIP experiments. ELISA test information is available upon request. Furthermore, the observed indirect immuno-signals of anti-CENH3 on R. canina cells are compatible with centromere data previously reported in the published literature for other species.<br><br>Previously validated antibodies:<br>KNL1 was previously validated by Oliviera et al. (2024) by Immunostaining and Western blot.<br><br>Validation by commercial providers:<br>mouse anti-alpha Tubulin (validation; https://www.sigmaaldrich.com/DE/de/product/sigma/t6199?srsltid=AfmBOopZVI8rakd6EyMi2t1B9KcuqVPwcg6UFkhi3RDxHotJnOjCzZQW)

rabbit anti-Histone H3 (validation: https://www.activemotif.com/documents/tds/39763.pdf; https://www.activemotif.com/catalog/details/39763)

Plants

Seed stocks

Rosa canina plants were cultivated under controlled greenhouse conditions (16h daylight, 26 °C, >70% humidity).

Novel plant genotypes

n/a

Authentication

n/a

ChIP-seq

Data deposition

☒ Confirm that both raw and final processed data have been deposited in a public database such as GEO.

☒ Confirm that you have deposited or provided access to graph files (e.g. BED files) for the called peaks.

Data access links
May remain private before publication.

https://www.ncbi.nlm.nih.gov/sra/?term=SRR32424402

Files in database submission

6919_A_run867_AGGTTCCT_S30_L001_R1_001.fastq.gz
6919_A_run867_AGGTTCCT_S30_L001_R2_001.fastq.gz
6919_A_run867_AGGTTCCT_S30_L002_R1_001.fastq.gz
6919_A_run867_AGGTTCCT_S30_L002_R2_001.fastq.gz
6919_B_run867_GAACCTTC_S31_L001_R1_001.fastq.gz
6919_B_run867_GAACCTTC_S31_L001_R2_001.fastq.gz
6919_B_run867_GAACCTTC_S31_L002_R1_001.fastq.gz
6919_B_run867_GAACCTTC_S31_L002_R2_001.fastq.gz
6919_C_run867_AAGTCCTC_S32_L001_R1_001.fastq.gz
6919_C_run867_AAGTCCTC_S32_L001_R2_001.fastq.gz
6919_C_run867_AAGTCCTC_S32_L002_R1_001.fastq.gz
6919_C_run867_AAGTCCTC_S32_L002_R2_001.fastq.gz
6919_D_run867_CCACAACA_S33_L001_R1_001.fastq.gz
6919_D_run867_CCACAACA_S33_L001_R2_001.fastq.gz
6919_D_run867_CCACAACA_S33_L002_R1_001.fastq.gz
6919_D_run867_CCACAACA_S33_L002_R2_001.fastq.gz
6919_E_run867_ATAACGCC_S34_L001_R1_001.fastq.gz
6919_E_run867_ATAACGCC_S34_L001_R2_001.fastq.gz
6919_E_run867_ATAACGCC_S34_L002_R1_001.fastq.gz
6919_E_run867_ATAACGCC_S34_L002_R2_001.fastq.gz
6919_F_run867_CCGGAATA_S35_L001_R1_001.fastq.gz
6919_F_run867_CCGGAATA_S35_L001_R2_001.fastq.gz
6919_F_run867_CCGGAATA_S35_L002_R1_001.fastq.gz
6919_F_run867_CCGGAATA_S35_L002_R2_001.fastq.gz

Genome browser session
(e.g. UCSC)

no longer applicable

Methodology

Replicates

Two biological replicates of CENH3 sequenced as 6919_A and 6919_B; Two biological replicates of H3 sequenced as 6919_C and 6919_D; Two biological replicates of input control sequenced as 6919_E and 6919_F.

Sequencing depth

6919_A: 21,902,101 pairs of reads (150bpx2); 30,512,245 reads uniquely mapped
6919_B: 21,890,693 pairs of reads (150bpx2); 31,961,379 reads uniquely mapped
6919_C: 21,798,217 pairs of reads (150bpx2); 34,691,504 reads uniquely mapped
6919_D: 21,798,366 pairs of reads (150bpx2); 34,835,465 reads uniquely mapped
6919_E: 21,304,165 pairs of reads (150bpx2); 33,647,990 reads uniquely mapped
6919_F: 21,682,527 pairs of reads (150bpx2); 34,367,915 reads uniquely mapped

Antibodies

The CENH3 gene of Rosa canina was identified using BLASTP with the parameter "-evalue 1e-5 -qcov_hsp_perc 50" and the A. thaliana CENH3 protein HTR12 (AT1G01370) was used as the reference. A specific polyclonal antibody against its CENH3 protein (ARVKHTAARKDRIKTARRQP-C / AB016310) was designed and synthetised by LifeTein with immunisation in rabbits.

Peak calling parameters

Genome indexing and read mapping were both done by bowtie2 (v2.5.4) with `--sensitive-local` flag activated for all experiments.

Peak calling parameters	After comparing the CENH3 domains with H3 or input samples woth bamCompare, peak calling was performed using the MACS3 pipeline with the inclusion of parameters --broad -g 1.9e+9.
Data quality	Mapped reads were not filtered by mapping quality considering the high similarity of four subgenomes. 251 out of 6105 broad peaks were 5 fold enriched when setting FDR value as 0.05.
Software	The raw 150bp pair-end ChIP -seq reads were checked by FastQC and then mapped to the R. canina haplotype phased reference genome using bowtie2 (as described in "Peak calling parameters"). The BAM file was converted to bigwig using the bamCompare tool from deeptools2, and then normalized to RPKM (reads per kilobase of transcript per million reads mapped). After this a peak calling, comparing the CENH3 domains with H3 or input samples, was performed using the MACS3 pipeline with the inclusion of parameters --broad -g 1.9e+9.

Flow Cytometry

Plots

Confirm that:

☒ The axis labels state the marker and fluorochrome used (e.g. CD4-FITC).

☒ The axis scales are clearly visible. Include numbers along axes only for bottom left plot of group (a 'group' is an analysis of identical markers).

☒ All plots are contour plots with outliers or pseudocolor plots.

☒ A numerical value for number of cells or percentage (with statistics) is provided.

Methodology

Sample preparation	Nuclei of mature pollen grains were isolated by applying the filter bursting method63 using the nuclei isolation buffer according to Galbraith et al. (1983)64. Pollen grains were burst on the surface of a 20 µm disposable CellTrics filter (Sysmex-Partec). The resulting nuclei suspension was stained with propidium iodide (50 µg/ml, PI) and run on a BD Influx cell sorter (BD Biosciences). After identifying the nuclear populations in a dotplot displaying the PI fluorescence signal (log-scale) versus side scatter signal (SSC, log-scale) a sort gate was defined in the corresponding fluorescence intensity (lin-scale) histogram. Per individual 200,000 generative nuclei (volume ca. 400 µL) were collected into a 1.5 ml reaction tube using the '1.0 Drop Pure' sort mode of the BD FACS Software (BD Biosciences). After adding 50 µL 1× TE and 50 µL NaN3 nuclei were sedimented by centrifugation (1000 × g for 10 min at 4°C). Afterwards 300 µL of the supernatant was removed and the nuclei with the remaining liquid stored at -20°C
Instrument	BD Influx cell sorter
Software	BD FACSDiva™ Software (v9.0)
Cell population abundance	Per individual 200,000 generative nuclei (volume ca. 400 µL) were collected into a 1.5 ml reaction tube using the '1.0 Drop Pure' sort mode of the BD FACS Software (BD Biosciences).
Gating strategy	After identifying the nuclear populations in a dotplot displaying the PI fluorescence signal (log-scale) versus side scatter signal (SSC, log-scale) a sort gate was defined in the corresponding fluorescence intensity (lin-scale) histogram.

☒ Tick this box to confirm that a figure exemplifying the gating strategy is provided in the Supplementary Information.
