# Supplementary material for: Bimodal centromeres in pentaploid dogroses shed light on their unique meiosis
Source: Nature. 2025 Jun 18;643(8070):148–57. doi: 10.1038/s41586-025-09171-z (PMC12222009; doi:10.1038/s41586-025-09171-z)
Supplement: Supplementary file 3 — Supplementary Data 1–17. [file 41586_2025_9171_MOESM3_ESM.zip › Suppl_Dataset_10_rosChi_ModDotPlot.pdf]

Rosa chinensis

GCA_041222415

ModDotPlot

Supplementary Dataset 9. Structural analysis of whole chromosome and centromeres of the diploid rose *R. chinensis* chromosomes. Window-size of 100 kbp and 10 kbp are shown for chromosome-wise and centromere plots, respectively.

chr1

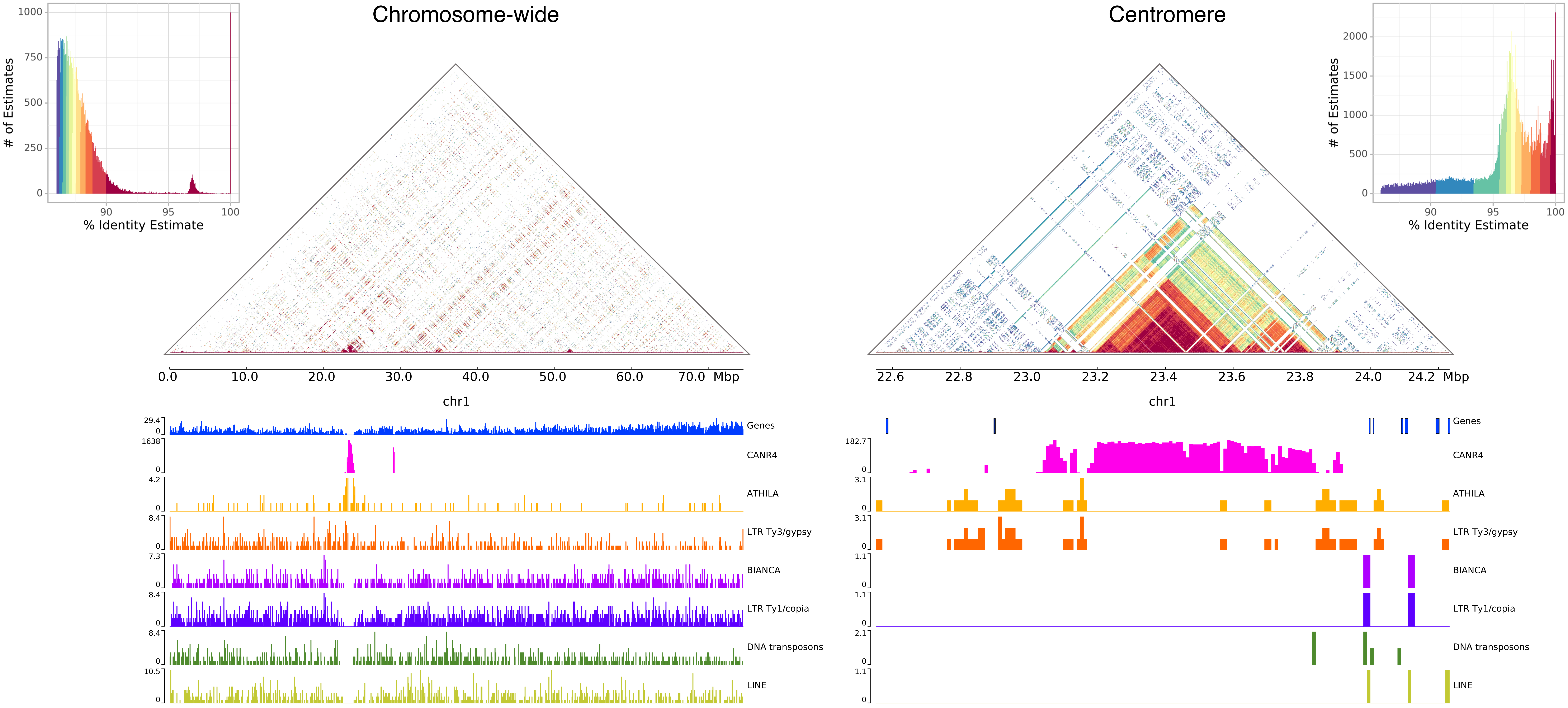

Chromosome-wide
Centromere
# of Estimates
% Identity Estimate
1000
750
500
250
0
90
95
100
2000
1500
1000
500
0
0.0
10.0
20.0
30.0
40.0
50.0
60.0
70.0
Mbp
22.6
22.8
23.0
23.2
23.4
23.6
23.8
24.0
24.2
chr1
Genes
CANR4
ATHILA
LTR Ty3/gypsy
BIANCA
LTR Ty1/copia
DNA transposons
LINE
29.4
1638
4.2
8.4
7.3
10.5
182.7
3.1
1.1
2.1

chr2

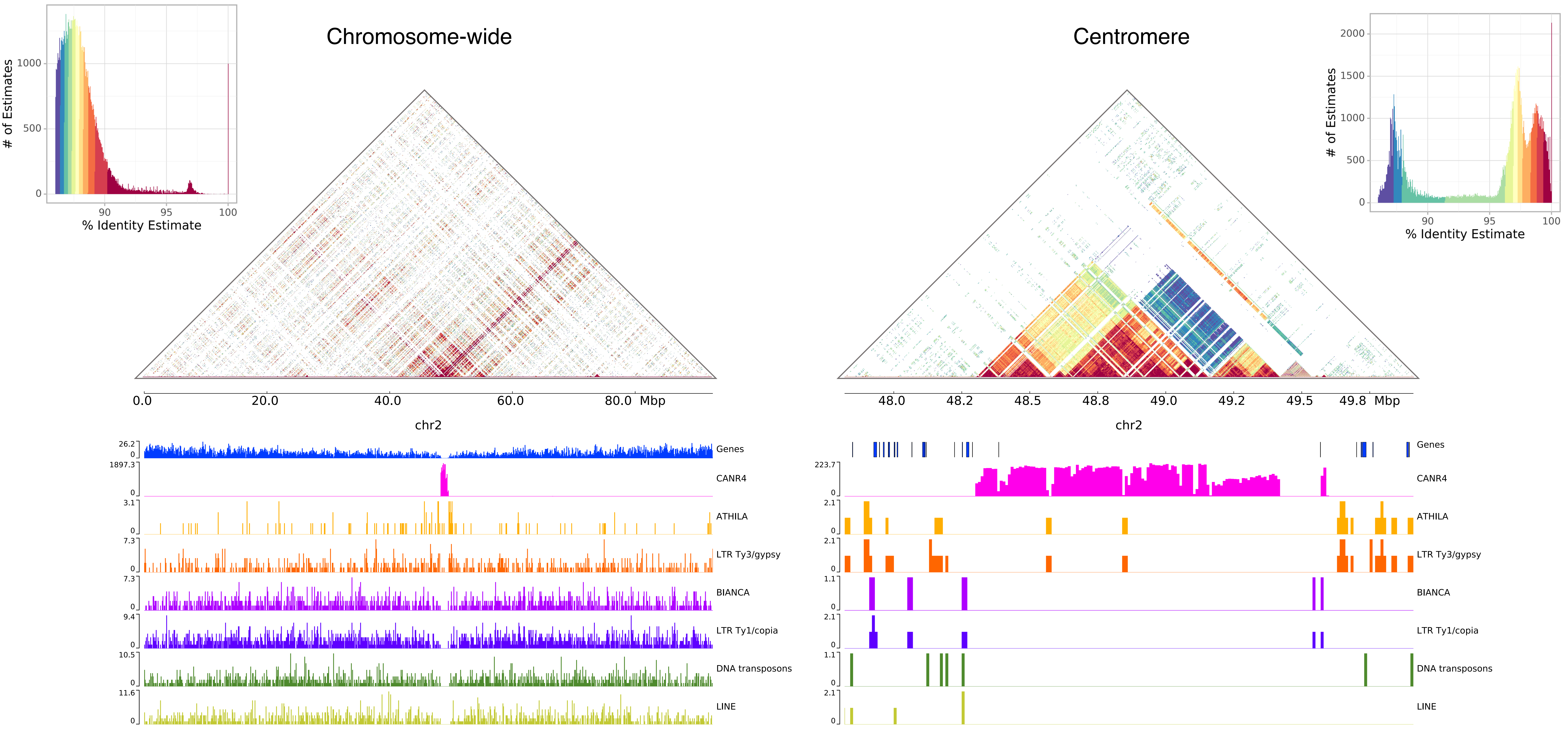

Chromosome-wide
Centromere
# of Estimates
% Identity Estimate
1000
500
0
90
95
100
2000
1500
1000
500
0
90
95
100
0.0
20.0
40.0
60.0
80.0 Mbp
chr2
48.0
48.2
48.5
48.8
49.0
49.2
49.5
49.8 Mbp
chr2
Genes
CANR4
ATHILA
LTR Ty3/gypsy
BIANCA
LTR Ty1/copia
DNA transposons
LINE
26.2
1897.3
3.1
7.3
7.3
9.4
10.5
11.6
223.7
2.1
2.1
1.1
2.1
1.1
2.1

chr3

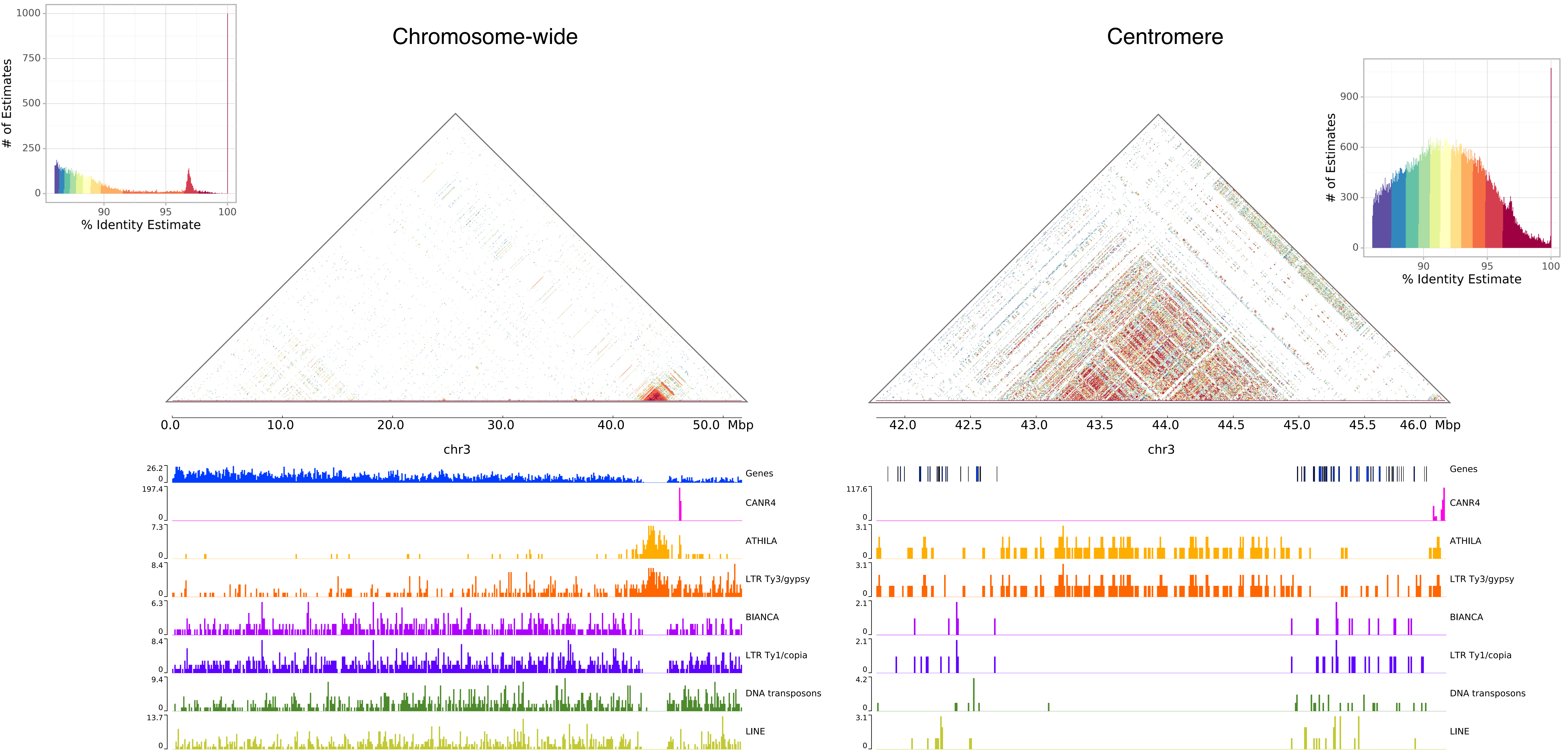

Chromosome-wide
Centromere
1000
750
500
250
0
90
95
100
# of Estimates
% Identity Estimate
900
600
300
0
0.0
10.0
20.0
30.0
40.0
50.0
Mbp
chr3
42.0
42.5
43.0
43.5
44.0
44.5
45.0
45.5
46.0
26.2
197.4
117.6
7.3
3.1
8.4
6.3
2.1
9.4
4.2
13.7
Genes
CANR4
ATHILA
LTR Ty3/gypsy
BIANCA
LTR Ty1/copia
DNA transposons
LINE

chr4

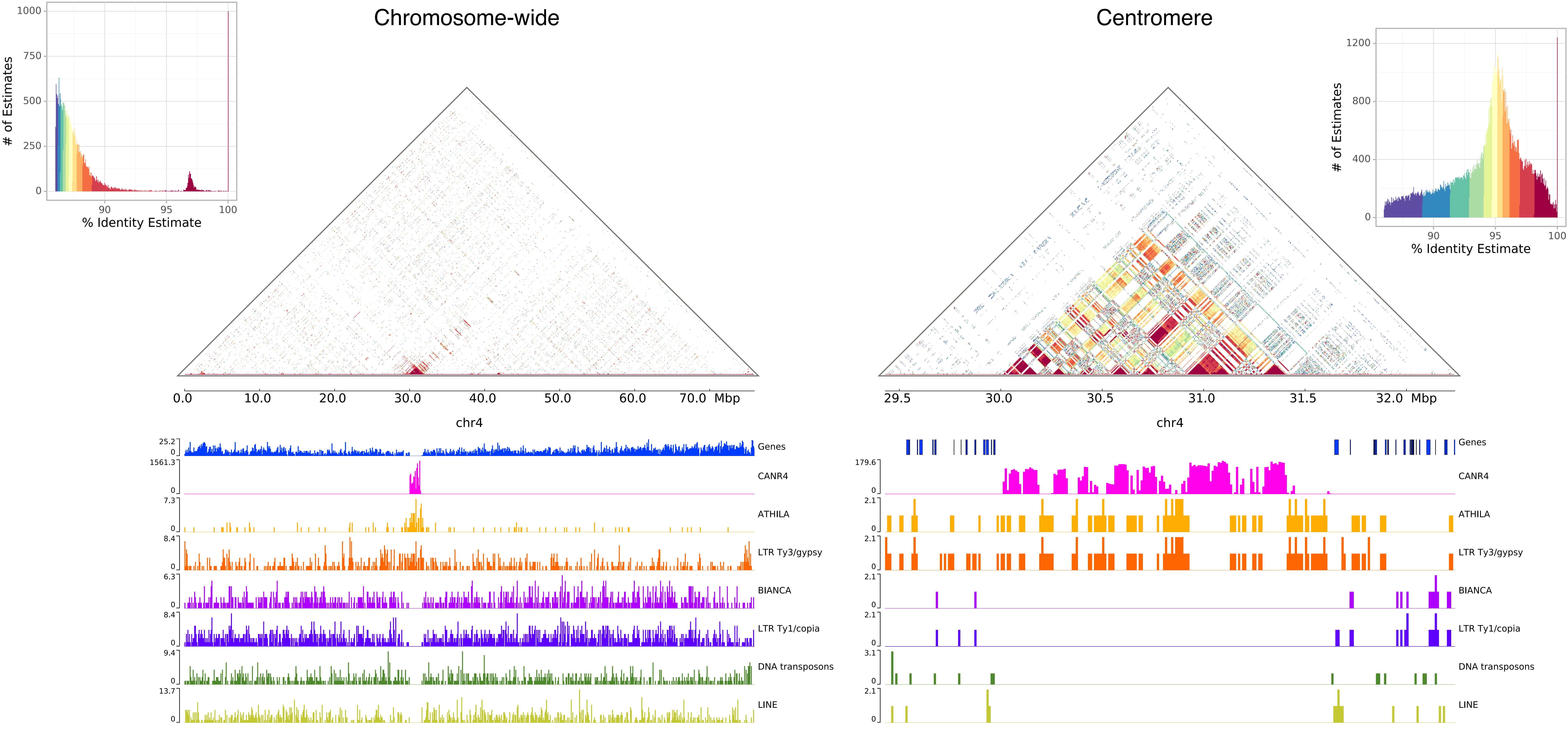

Chromosome-wide
Centromere
# of Estimates
% Identity Estimate
1000
750
500
250
0
90
95
100
1200
800
400
0.0
10.0
20.0
30.0
40.0
50.0
60.0
70.0
Mbp
29.5
30.0
30.5
31.0
31.5
32.0
chr4
Genes
CANR4
ATHILA
LTR Ty3/gypsy
BIANCA
LTR Ty1/copia
DNA transposons
LINE
25.2
1561.3
7.3
8.4
6.3
9.4
13.7
179.6
2.1
3.1

chr5

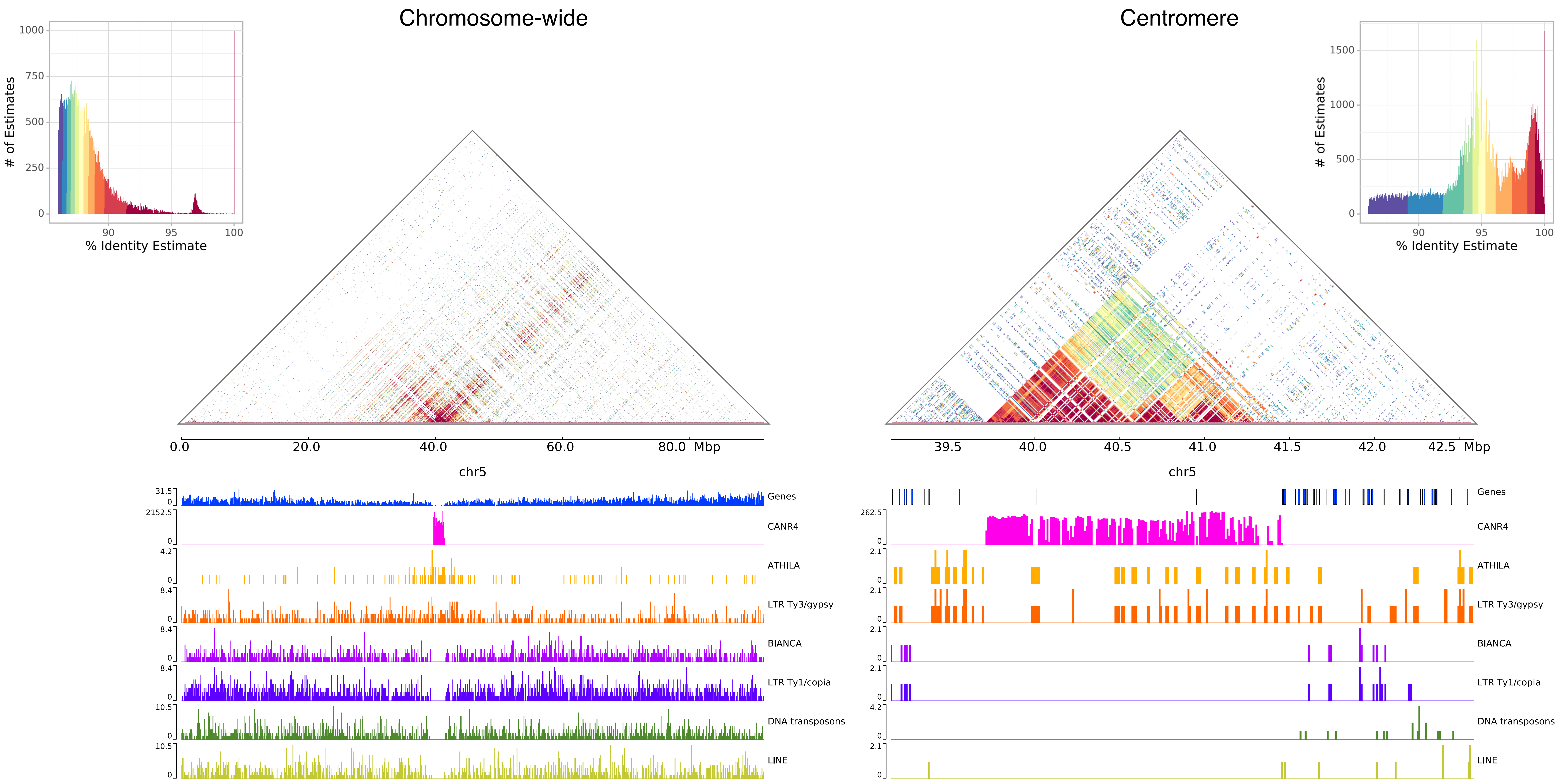
Chromosome-wide
Centromere
# of Estimates
% Identity Estimate
1000
750
500
250
0
90
95
100
1500
1000
500
0
0.0
20.0
40.0
60.0
80.0 Mbp
39.5
40.0
40.5
41.0
41.5
42.0
42.5 Mbp
chr5
Genes
CANR4
ATHILA
LTR Ty3/gypsy
BIANCA
LTR Ty1/copia
DNA transposons
LINE
31.5
2152.5
4.2
8.4
10.5
262.5
2.1

chr6

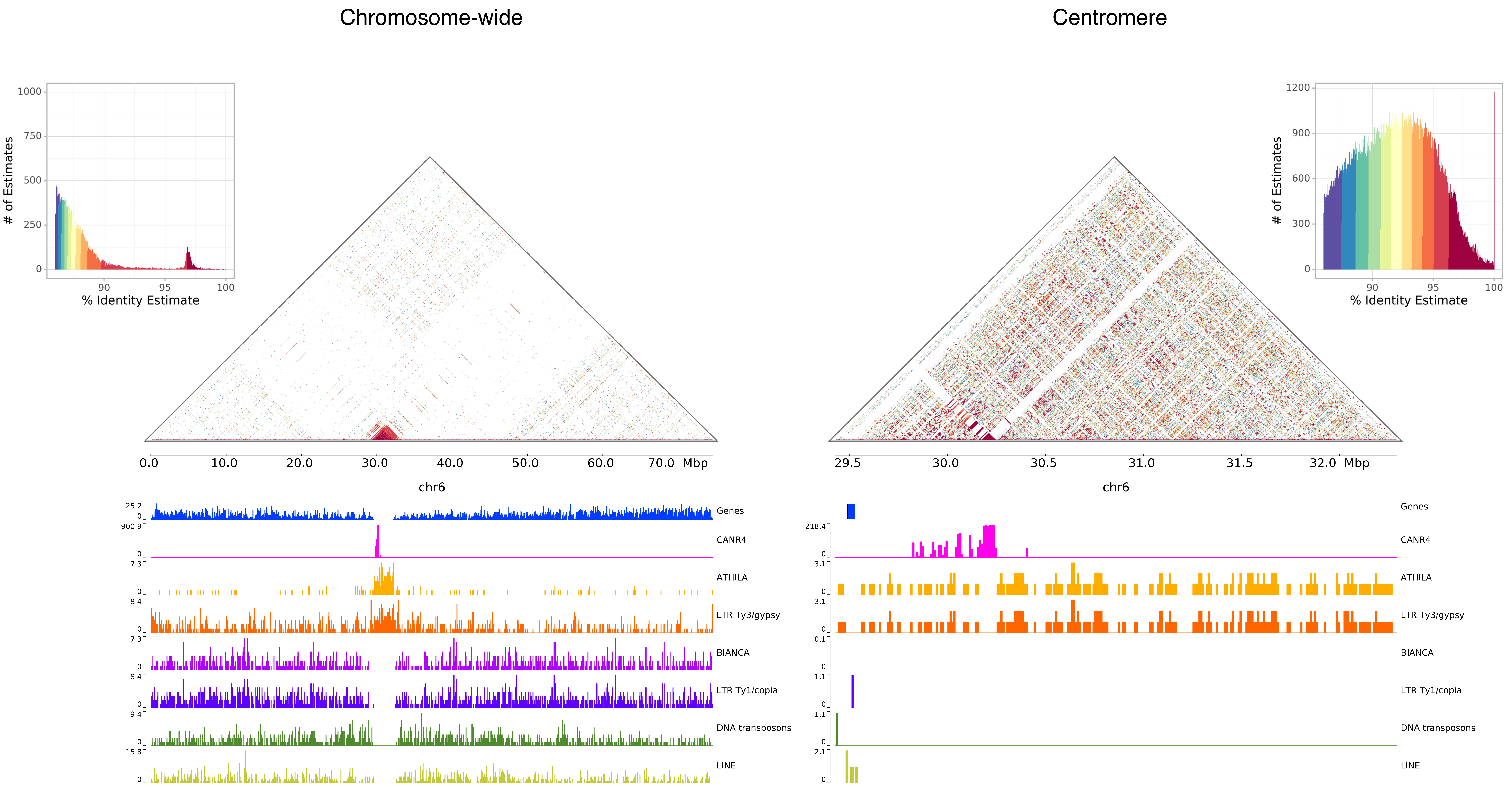
Chromosome-wide
Centromere
# of Estimates
% Identity Estimate
1000
750
500
250
0
90
95
100
1200
900
600
300
0.0
10.0
20.0
30.0
40.0
50.0
60.0
70.0 Mbp
chr6
29.5
30.0
30.5
31.0
31.5
32.0 Mbp
chr6
Genes
CANR4
ATHILA
LTR Ty3/gypsy
BIANCA
LTR Ty1/copia
DNA transposons
LINE
25.2
900.9
7.3
8.4
7.3
8.4
9.4
15.8
218.4
3.1
3.1
0.1
1.1
1.1
2.1

chr7

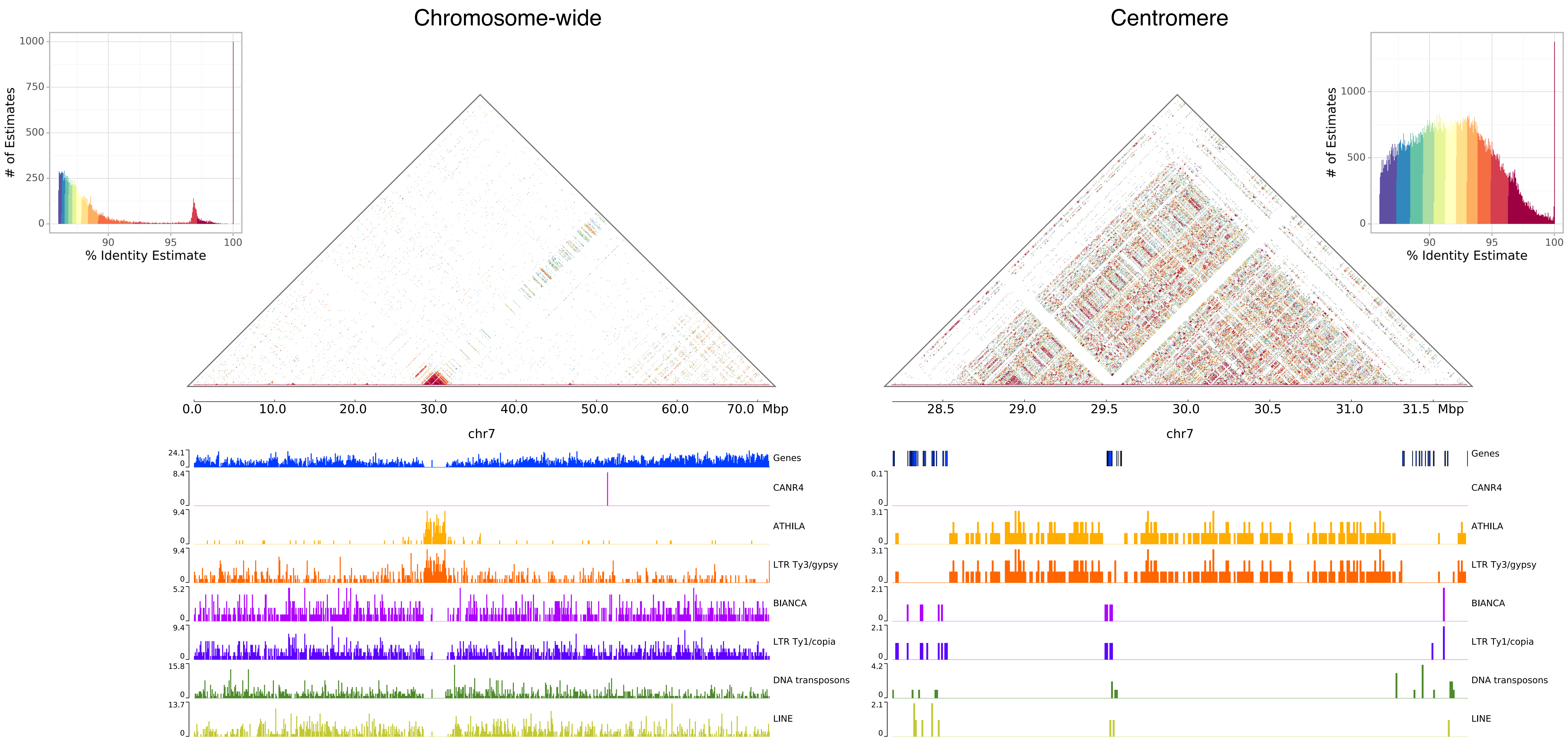
Chromosome-wide
Centromere
# of Estimates
% Identity Estimate
1000
750
500
250
0
90
95
100
0.0
10.0
20.0
30.0
40.0
50.0
60.0
70.0
Mbp
chr7
28.5
29.0
29.5
30.0
30.5
31.0
31.5
Genes
CANR4
ATHILA
LTR Ty3/gypsy
BIANCA
LTR Ty1/copia
DNA transposons
LINE
24.1
8.4
9.4
5.2
15.8
13.7
0.1
3.1
2.1
4.2
