# Supplementary material for: Bimodal centromeres in pentaploid dogroses shed light on their unique meiosis
Source: Nature. 2025 Jun 18;643(8070):148–57. doi: 10.1038/s41586-025-09171-z (PMC12222009; doi:10.1038/s41586-025-09171-z)
Supplement: Supplementary file 3 — Supplementary Data 1–17. [file 41586_2025_9171_MOESM3_ESM.zip › Suppl_Dataset_11_rosRug_ModDotPlot.pdf]

Rosa rugosa

GCA_958449725.1

ModDotPlot

Supplementary Dataset 10. Structural analysis of whole chromosome and centromeres of the diploid rose *R. rugosa* chromosomes. Window-size of 100 kbp and 10 kbp are shown for chromosome-wise and centromere plots, respectively.

chr1

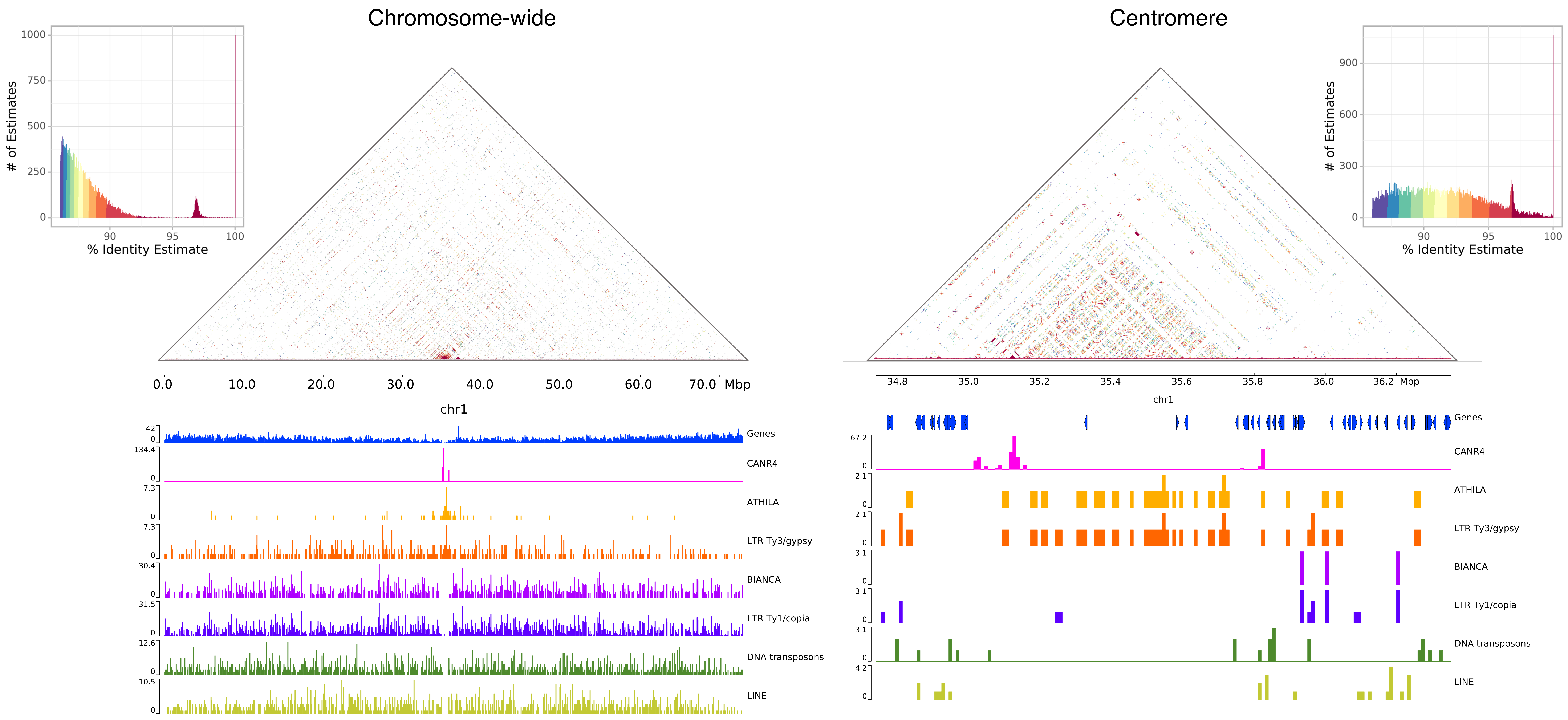

Chromosome-wide
Centromere
# of Estimates
% Identity Estimate
1000
750
500
250
0
90
95
100
900
600
300
0.0
10.0
20.0
30.0
40.0
50.0
60.0
70.0 Mbp
chr1
34.8
35.0
35.2
35.4
35.6
35.8
36.0
36.2 Mbp
Genes
CANR4
ATHILA
LTR Ty3/gypsy
BIANCA
LTR Ty1/copia
DNA transposons
LINE
42
134.4
7.3
30.4
31.5
12.6
10.5
67.2
2.1
3.1
4.2

chr2

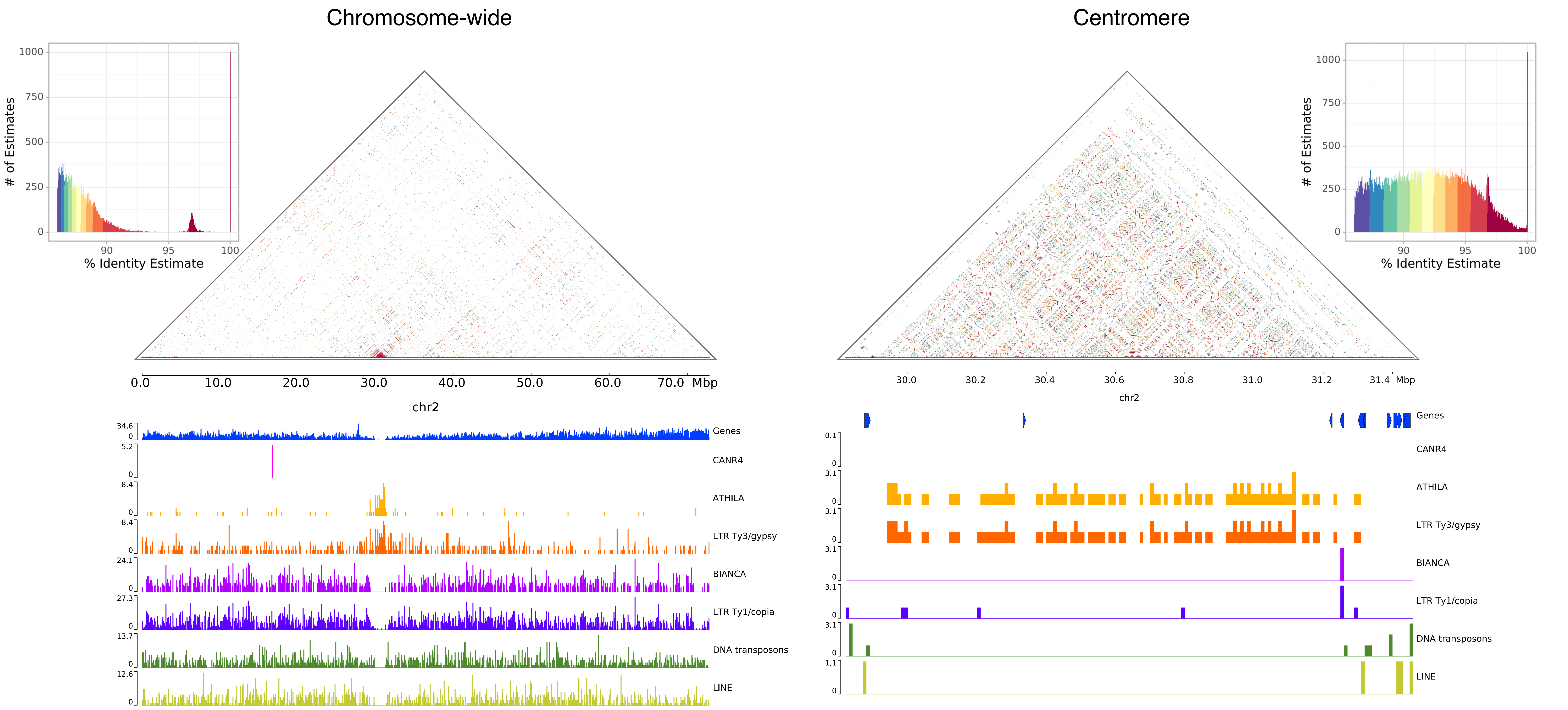

Chromosome-wide
Centromere
# of Estimates
% Identity Estimate
1000
750
500
250
0
90
95
100
0.0
10.0
20.0
30.0
40.0
50.0
60.0
70.0
Mbp
chr2
30.0
30.2
30.4
30.6
30.8
31.0
31.2
31.4
Genes
CANR4
ATHILA
LTR Ty3/gypsy
BIANCA
LTR Ty1/copia
DNA transposons
LINE
34.6
5.2
8.4
24.1
27.3
13.7
12.6
0.1
3.1
1.1

chr3

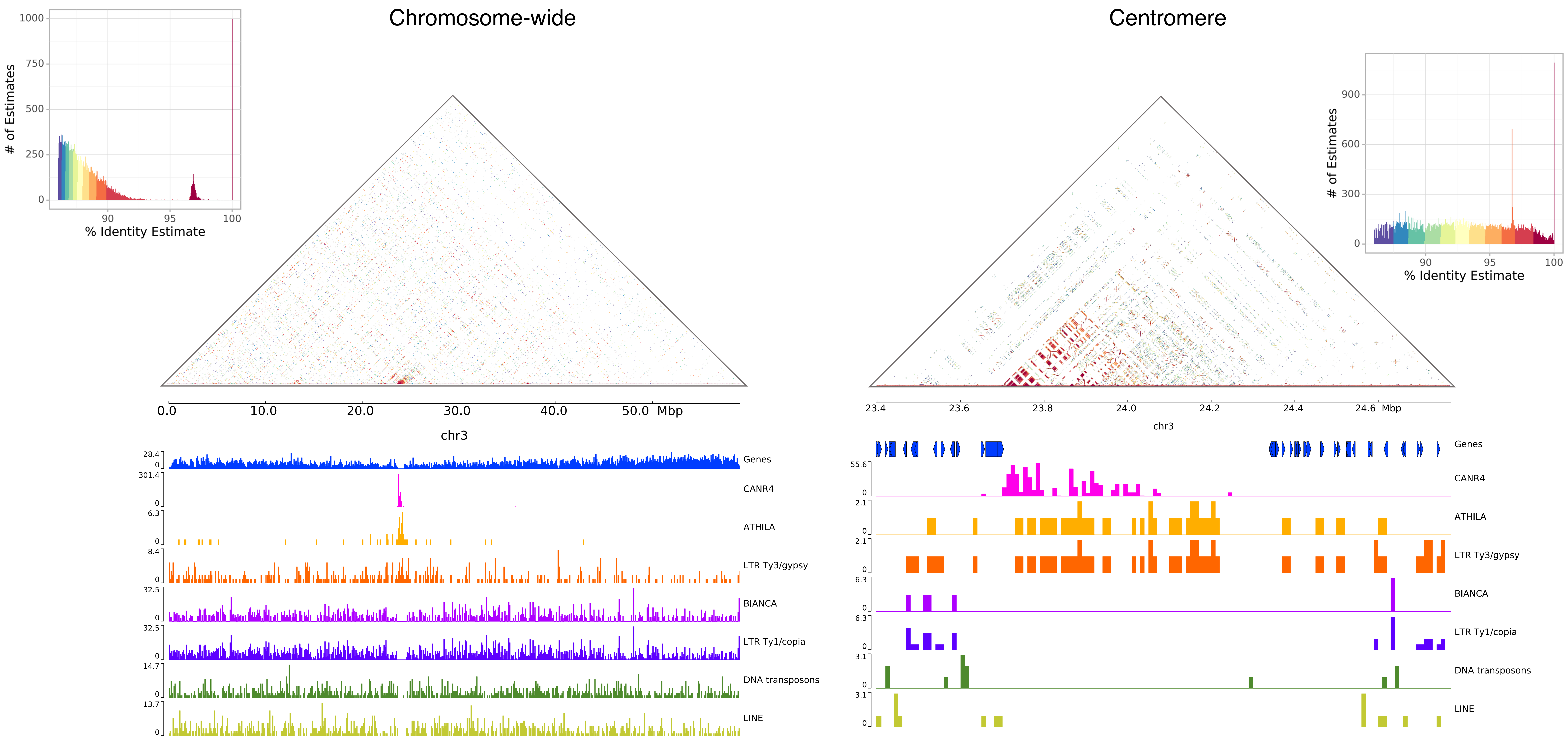
Chromosome-wide
Centromere
# of Estimates
% Identity Estimate
1000
750
500
250
0
90
95
100
900
600
300
0.0
10.0
20.0
30.0
40.0
50.0
Mbp
chr3
23.4
23.6
23.8
24.0
24.2
24.4
24.6
Genes
CANR4
ATHILA
LTR Ty3/gypsy
BIANCA
LTR Ty1/copia
DNA transposons
LINE
28.4
301.4
6.3
8.4
32.5
14.7
13.7
55.6
2.1
3.1

chr4

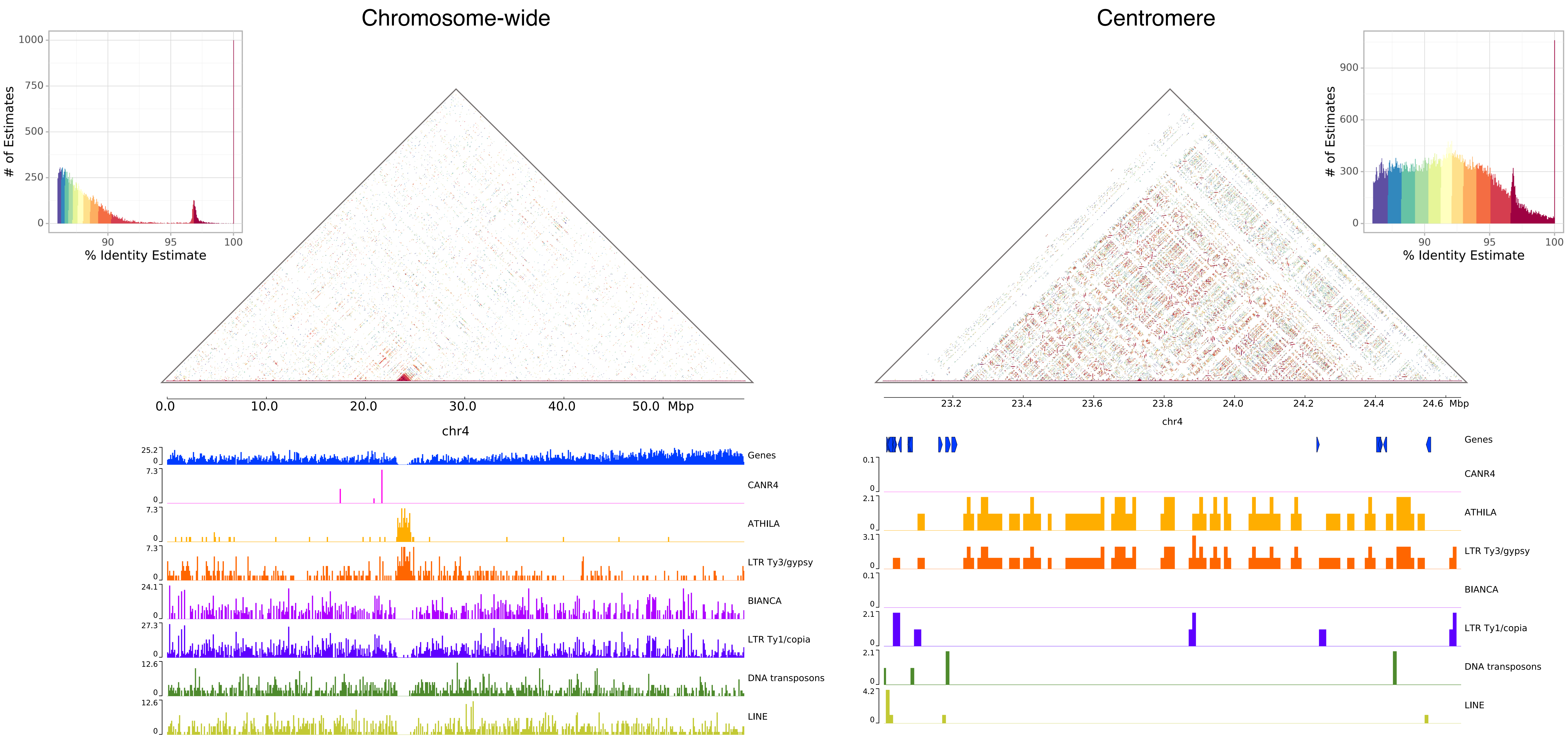

Chromosome-wide
Centromere
# of Estimates
% Identity Estimate
1000
750
500
250
0
90
95
100
900
600
300
0.0
10.0
20.0
30.0
40.0
50.0 Mbp
chr4
23.2
23.4
23.6
23.8
24.0
24.2
24.4
24.6 Mbp
Genes
CANR4
ATHILA
LTR Ty3/gypsy
BIANCA
LTR Ty1/copia
DNA transposons
LINE
25.2
7.3
24.1
27.3
12.6
0.1
2.1
3.1
4.2

chr5

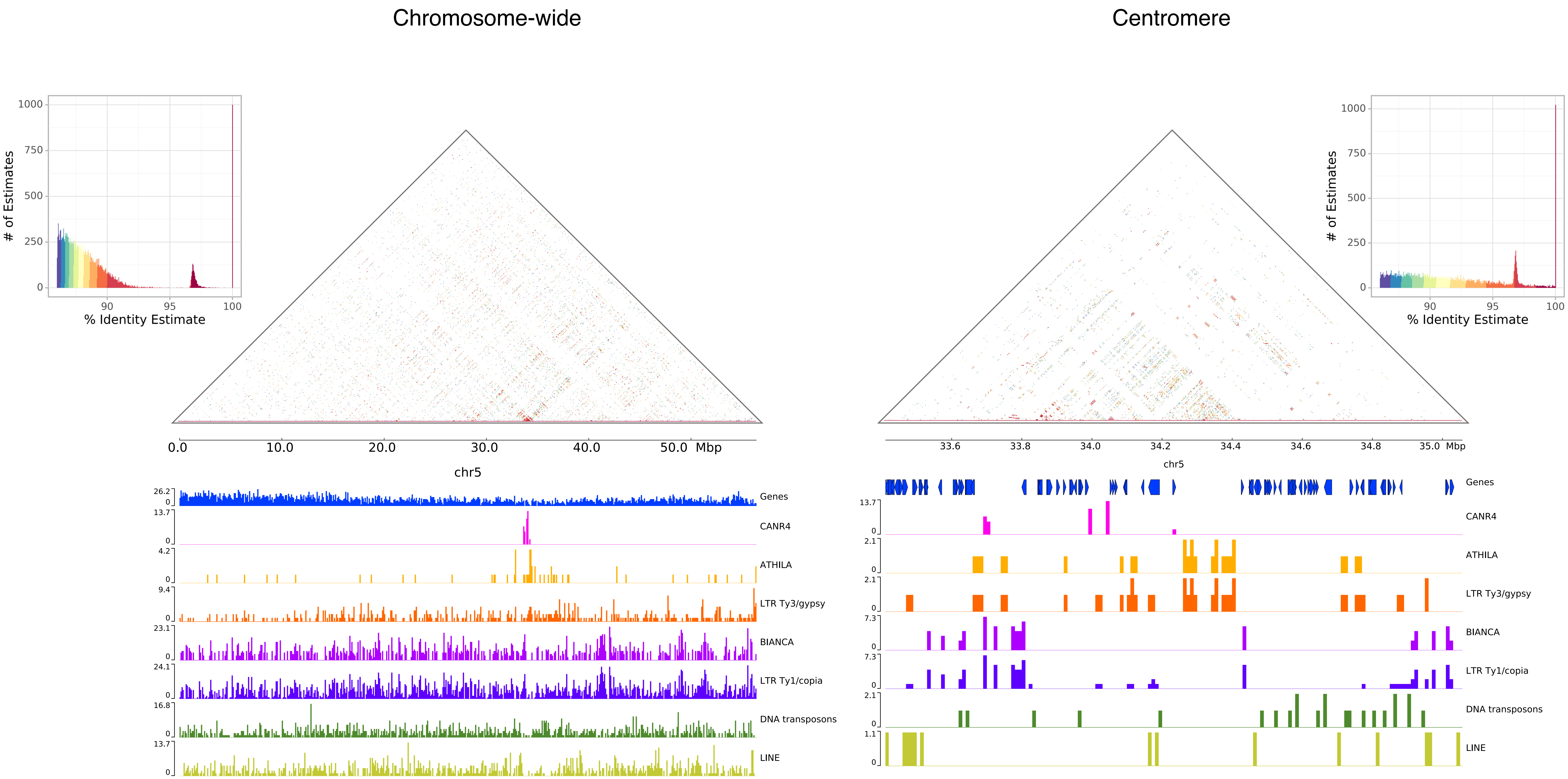

Chromosome-wide
Centromere
# of Estimates
% Identity Estimate
1000
750
500
250
0
90
95
100
0.0
10.0
20.0
30.0
40.0
50.0 Mbp
chr5
33.6
33.8
34.0
34.2
34.4
34.6
34.8
35.0 Mbp
Genes
CANR4
ATHILA
LTR Ty3/gypsy
BIANCA
LTR Ty1/copia
DNA transposons
LINE
26.2
13.7
4.2
9.4
23.1
24.1
16.8
2.1
7.3
1.1

chr6

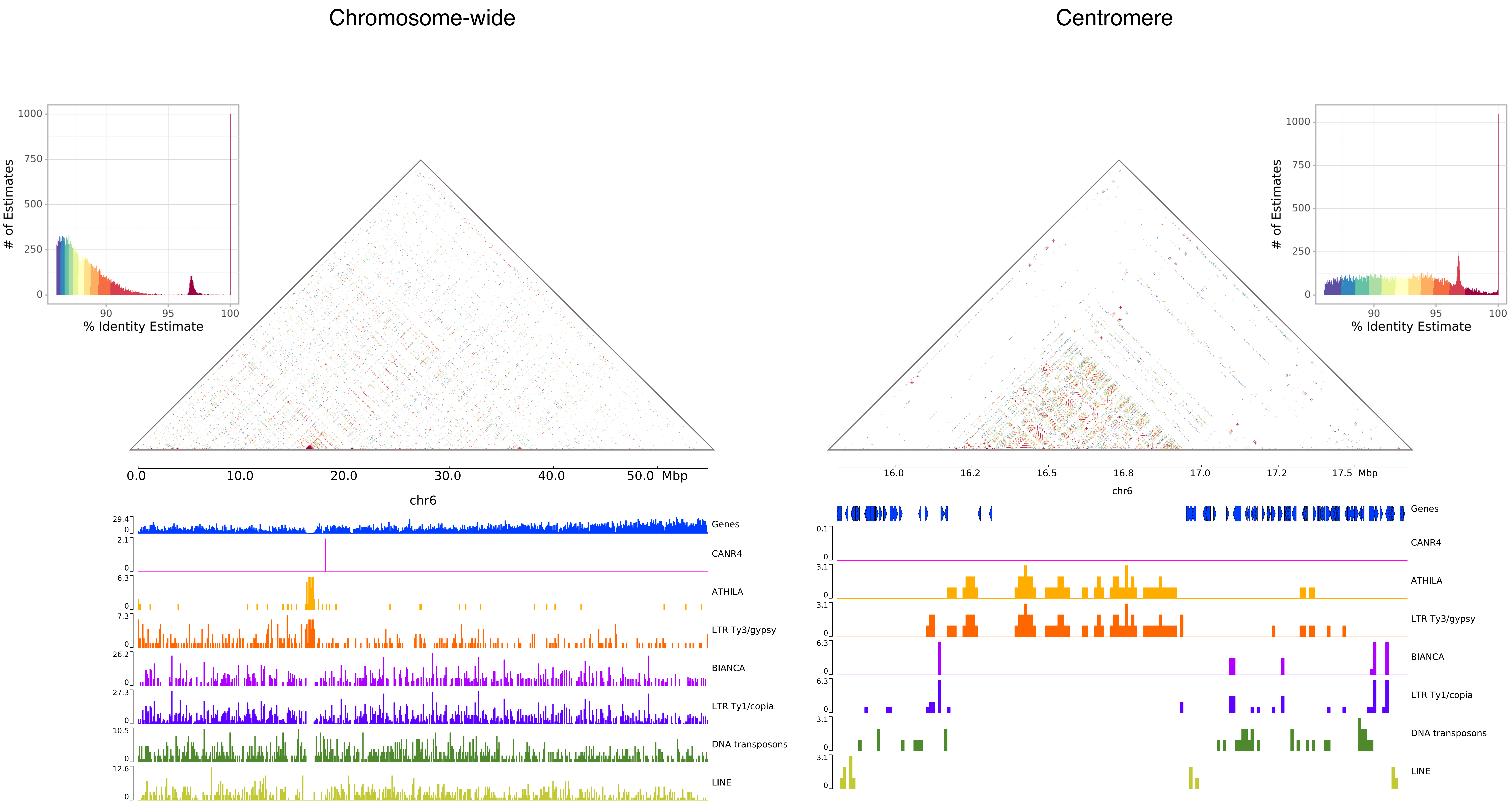

Chromosome-wide
Centromere
# of Estimates
% Identity Estimate
1000
750
500
250
0
90
95
100
0.0
10.0
20.0
30.0
40.0
50.0 Mbp
chr6
16.0
16.2
16.5
16.8
17.0
17.2
17.5 Mbp
Genes
CANR4
ATHILA
LTR Ty3/gypsy
BIANCA
LTR Ty1/copia
DNA transposons
LINE
29.4
2.1
6.3
7.3
26.2
27.3
10.5
12.6
0.1
3.1

chr7

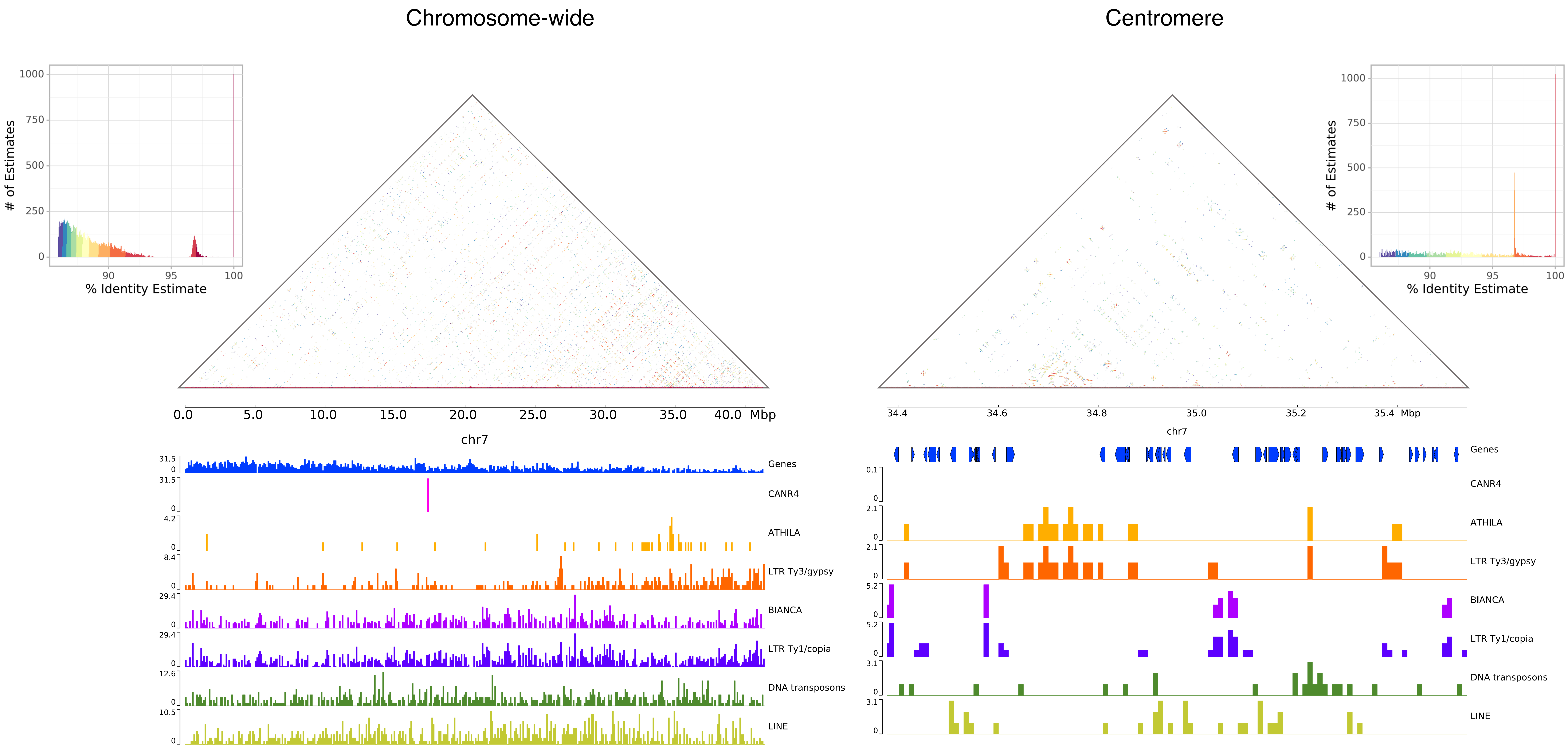
Chromosome-wide
Centromere
# of Estimates
% Identity Estimate
1000
750
500
250
0
90
95
100
0.0
5.0
10.0
15.0
20.0
25.0
30.0
35.0
40.0
Mbp
chr7
34.4
34.6
34.8
35.0
35.2
35.4
Genes
CANR4
ATHILA
LTR Ty3/gypsy
BIANCA
LTR Ty1/copia
DNA transposons
LINE
31.5
4.2
8.4
29.4
12.6
10.5
0.1
2.1
5.2
3.1
