# Supplementary material for: Bimodal centromeres in pentaploid dogroses shed light on their unique meiosis
Source: Nature. 2025 Jun 18;643(8070):148–57. doi: 10.1038/s41586-025-09171-z (PMC12222009; doi:10.1038/s41586-025-09171-z)
Supplement: Supplementary file 3 — Supplementary Data 1–17. [file 41586_2025_9171_MOESM3_ESM.zip › Suppl_Dataset_13_glmm_analysis_ESM.pdf]

Supplementary Dataset 13: MCMCglmm Analysis of Centromeric Athila/CANR4 Abundance _LOG

Mai 03, 2025

Description

A bivariate Bayesian Generalized Linear Mixed Model (GLMM) was used to analyse the repeat abundance of CANR4 satellite repeats and Ty3/Gypsy ATHILA retroelements in a pre-defined centromeric region.

Data structure

Athila_log: Log scaled centromeric abundance of Ty3/Gypsy ATHILA retroelements per chromosome in base pairs [bp]

CANR4_log : Log scaled centromeric abundance of CANR4 satellite repeats per chromosome in base pairs [bp] subgenome: Category of subgenome (S1, S2, R3, R4)

linkage: Category of chromosome linkage group (L1, L2, L3, L4, L5, L6, L7)

genome: Category of genome (R. canina S27, R. canina DToL, R. agrestis DToL)

pairing: Category of pairing mode per chromosome (B = bivalent, U = univalent, Ub = univalent, but in other species bivalent)

pairer: Category of general pairing behavior per chromosome (pairer = S1 and R4 chromosomes, not-pairer = S2 and R3 chromosomes) ### not included in the model anymore ###

```
# Load necessary libraries
#install.packages("lme4")
#install.packages("glmmTMB")
#install.packages("nortest")
library(lme4)
```

```
## Lade nötiges Paket: Matrix
```

```
library(glmmTMB)
```

```
## Warning in check_dep_version(dep_pkg = "TMB"): package version mismatch:
## glmmTMB was built with TMB package version 1.9.15
## Current TMB package version is 1.9.17
## Please re-install glmmTMB from source or restore original 'TMB' package (see '?reinstalling' for more
```

```
library(nortest)
library(ggplot2)
library(knitr)
library(MCMCglmm)
```

```
## Lade nötiges Paket: coda
```

```
## Lade nötiges Paket: ape
```

```
library(dplyr)
```

```
##
## Attache Paket: 'dplyr'

## Das folgende Objekt ist maskiert 'package:ape':
##
##     where

## Die folgenden Objekte sind maskiert von 'package:stats':
##
##     filter, lag

## Die folgenden Objekte sind maskiert von 'package:base':
##
##     intersect, setdiff, setequal, union

library(tidyr)

##
## Attache Paket: 'tidyr'

## Die folgenden Objekte sind maskiert von 'package:Matrix':
##
##     expand, pack, unpack

library(patchwork)
library(forcats)

setwd("/home/veit/Senckenberg Dropbox/veit herklotz/DFG_II/Rosa_canina_genome/CENH3/centromer_AM/")

# Read the data
df <- read.table("glmm_in_Ub.txt", header = TRUE, sep = "\t")

# Factor levels and clean-up
df$subgenome <- factor(df$subgenome, levels = c("S1", "S2", "R3", "R4"))
df$genome <- as.factor(df$genome)
df$pairing <- as.factor(df$pairing)
df$linkage <- as.factor(df$linkage)

levels(df$pairing)

## [1] "B"  "U"  "Ub"

df$pairing <- relevel(df$pairing, ref = "U") ### to have univalent (U) as baseline
levels(df$pairing)

## [1] "U"  "B"  "Ub"

#### showing the head of the data
head(df)

##    Athila   CANR4         chr pairing linkage subgenome     pairer  genome
## 1 1206896       0 Rca1_S1_h2       B      L1        S1     pairer     S27
## 2 1189004       0 Rca1_S1_h1       B      L1        S1     pairer     S27
## 3  658930       0    Rca1_R4      Ub      L1        R4     pairer     S27
## 4  619749 1029096    Rca1_S2       U      L1        S2 not-pairer     S27
## 5  367603 1156515    Rca1_R3       U      L1        R3 not-pairer     S27
## 6 1167458       0 Rca1_S1_h1       B      L1        S1     pairer canDTOL

#### Log transformation of the data
df$Athila_log <- log(df$Athila+1,10)
```

```r
df$CANR4_log <- log(df$CANR4+1,10)
```

Normality Test

```r
# Test if the ratio is normally distributed (Shapiro-Wilk test)
shapiro_testAthila <- shapiro.test(df$Athila_log)
print(shapiro_testAthila)
```

```
##
##  Shapiro-Wilk normality test
##
## data:  df$Athila_log
## W = 0.97632, p-value = 0.05682
```

```r
shapiro_testCANR4 <- shapiro.test(df$CANR4_log)
print(shapiro_testCANR4)
```

```
##
##  Shapiro-Wilk normality test
##
## data:  df$CANR4_log
## W = 0.67707, p-value = 7.142e-14
```

MCMCglmm for a True Multivariate Model on total base pairs

```r
#install.packages("MCMCglmm")
library(MCMCglmm)
# Define priors
prior_updated <- list(
  R = list(V = diag(2) * 0.5, nu = 5),
  G = list(
    G1 = list(V = diag(2) * 0.5, nu = 5),   # Random effect for 'linkage'
    G2 = list(V = diag(2) * 0.5, nu = 5),   # Random effect for 'subgenome'
    G3 = list(V = diag(2) * 0.5, nu = 5)    # Random effect for 'genome'
  )
)

# Run MCMCglmm
model_bivariate_full <- MCMCglmm(
  cbind(Athila_log, CANR4_log) ~ trait - 1 + trait:pairing,  # Pairing as fixed effect
  random = ~ us(trait):linkage + us(trait):subgenome + us(trait):genome,  # random
  rcov = ~ us(trait):units,
  family = c("gaussian", "gaussian"), ### CANR4 is not normal distributed, but because of zero fit
  data = df,
  prior = prior_updated,
  nitt = 100000,
  burnin = 50000,
  thin = 50
)

summary(model_bivariate_full)
```

```r
# Summarize the model results
summary(model_bivariate_full)
```

```
##
```

```
##  Iterations = 50001:99951
##  Thinning interval  = 50
##  Sample size  = 1000
##
##  DIC: 402.4371
##
##  G-structure:  ~us(trait):linkage
##
##                                           post.mean l-95% CI u-95% CI eff.samp
## traitAthila_log:traitAthila_log.linkage    0.31970  0.09359   0.6741     1000
## traitCANR4_log:traitAthila_log.linkage    -0.02438 -0.49163   0.4962     1000
## traitAthila_log:traitCANR4_log.linkage    -0.02438 -0.49163   0.4962     1000
## traitCANR4_log:traitCANR4_log.linkage      1.15054  0.16916   2.6170     1089
##
##                ~us(trait):subgenome
##
##                                             post.mean l-95% CI u-95% CI eff.samp
## traitAthila_log:traitAthila_log.subgenome    0.63955   0.1457   1.6002     1000
## traitCANR4_log:traitAthila_log.subgenome    -0.03661  -0.7880   0.6034     1000
## traitAthila_log:traitCANR4_log.subgenome    -0.03661  -0.7880   0.6034     1000
## traitCANR4_log:traitCANR4_log.subgenome      0.69970   0.1010   1.7302     1000
##
##                ~us(trait):genome
##
##                                          post.mean l-95% CI u-95% CI eff.samp
## traitAthila_log:traitAthila_log.genome    0.60647   0.1251   1.5117     1000
## traitCANR4_log:traitAthila_log.genome     0.01129  -0.6421   0.6532     1000
## traitAthila_log:traitCANR4_log.genome     0.01129  -0.6421   0.6532     1000
## traitCANR4_log:traitCANR4_log.genome      0.66965   0.1403   1.6902     1000
##
##  R-structure:  ~us(trait):units
##
##                                         post.mean l-95% CI u-95% CI eff.samp
## traitAthila_log:traitAthila_log.units    0.04786  0.03405  0.06033     1000
## traitCANR4_log:traitAthila_log.units    -0.11374 -0.20417 -0.01963     1000
## traitAthila_log:traitCANR4_log.units    -0.11374 -0.20417 -0.01963     1000
## traitCANR4_log:traitCANR4_log.units      4.52410  3.32017  5.80431     1000
##
##  Location effects: cbind(Athila_log, CANR4_log) ~ trait - 1 + trait:pairing
##
##                          post.mean l-95% CI u-95% CI eff.samp  pMCMC
## traitAthila_log             5.4779   4.1062   7.0259     1000 <0.001 ***
## traitCANR4_log              6.1538   4.1291   7.6071     1000 <0.001 ***
## traitAthila_log:pairingB    0.3621  -1.2615   1.7626     1000  0.600
## traitCANR4_log:pairingB    -3.5523  -5.4302  -1.6879     1000  0.002 **
## traitAthila_log:pairingUb   0.3758  -1.3092   1.7257     1000  0.594
## traitCANR4_log:pairingUb   -3.7626  -5.6847  -1.7571     1000 <0.001 ***
## ---
## Signif. codes:  0 '***' 0.001 '**' 0.01 '*' 0.05 '.' 0.1 ' ' 1
```

```
# Extract Fixed Effects
fixed_effects <- as.data.frame(summary(model_bivariate_full)$solutions)

# Extract Random Effects (G-structure)
```

```
random_effects <- as.data.frame(summary(model_bivariate_full)$Gcovariances)

# Extract Residual Variance (R-structure)
residual_variance <- as.data.frame(summary(model_bivariate_full)$Rcovariances)

# Save as CSV
write.csv(fixed_effects, "fixed_effects.csv", row.names = TRUE)
write.csv(random_effects, "random_effects.csv", row.names = TRUE)
write.csv(residual_variance, "residual_variance.csv", row.names = TRUE)
```

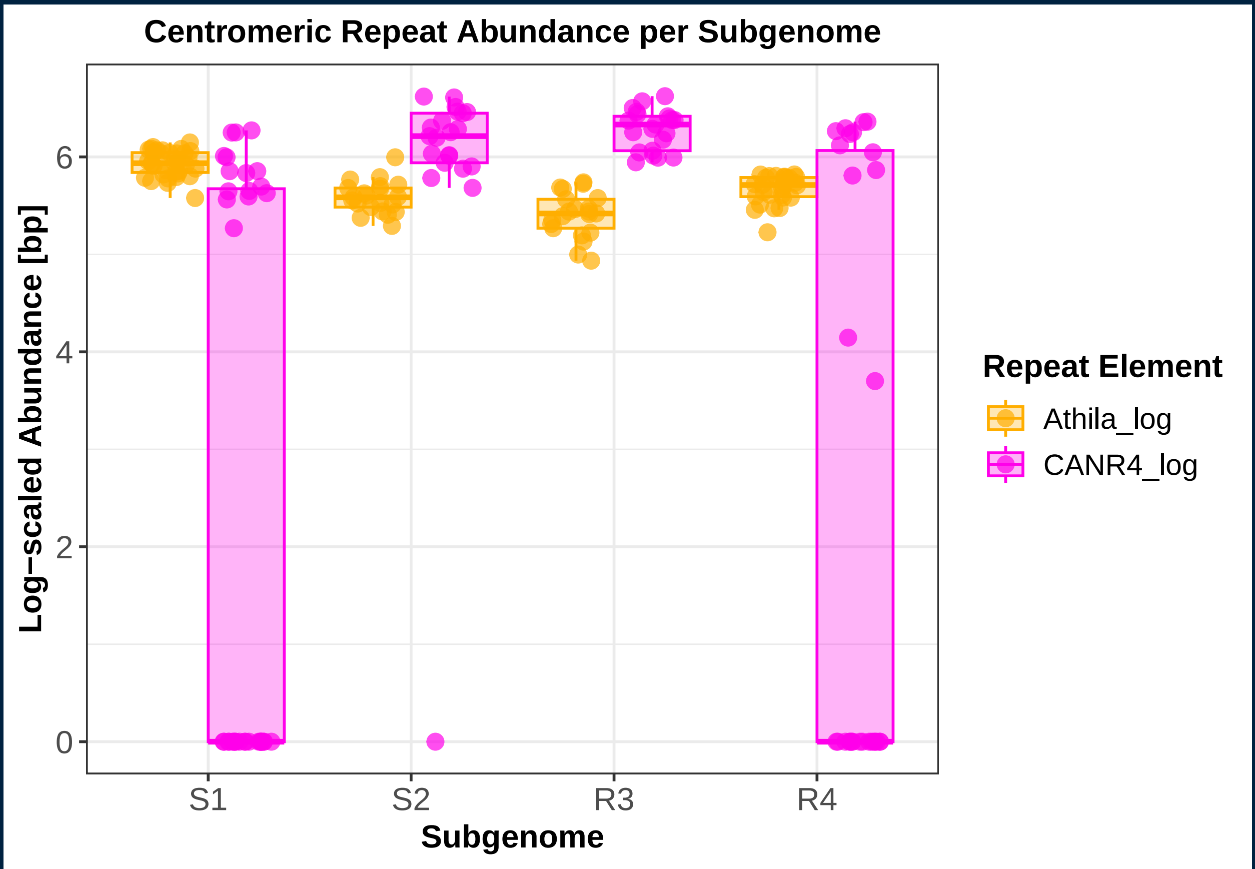

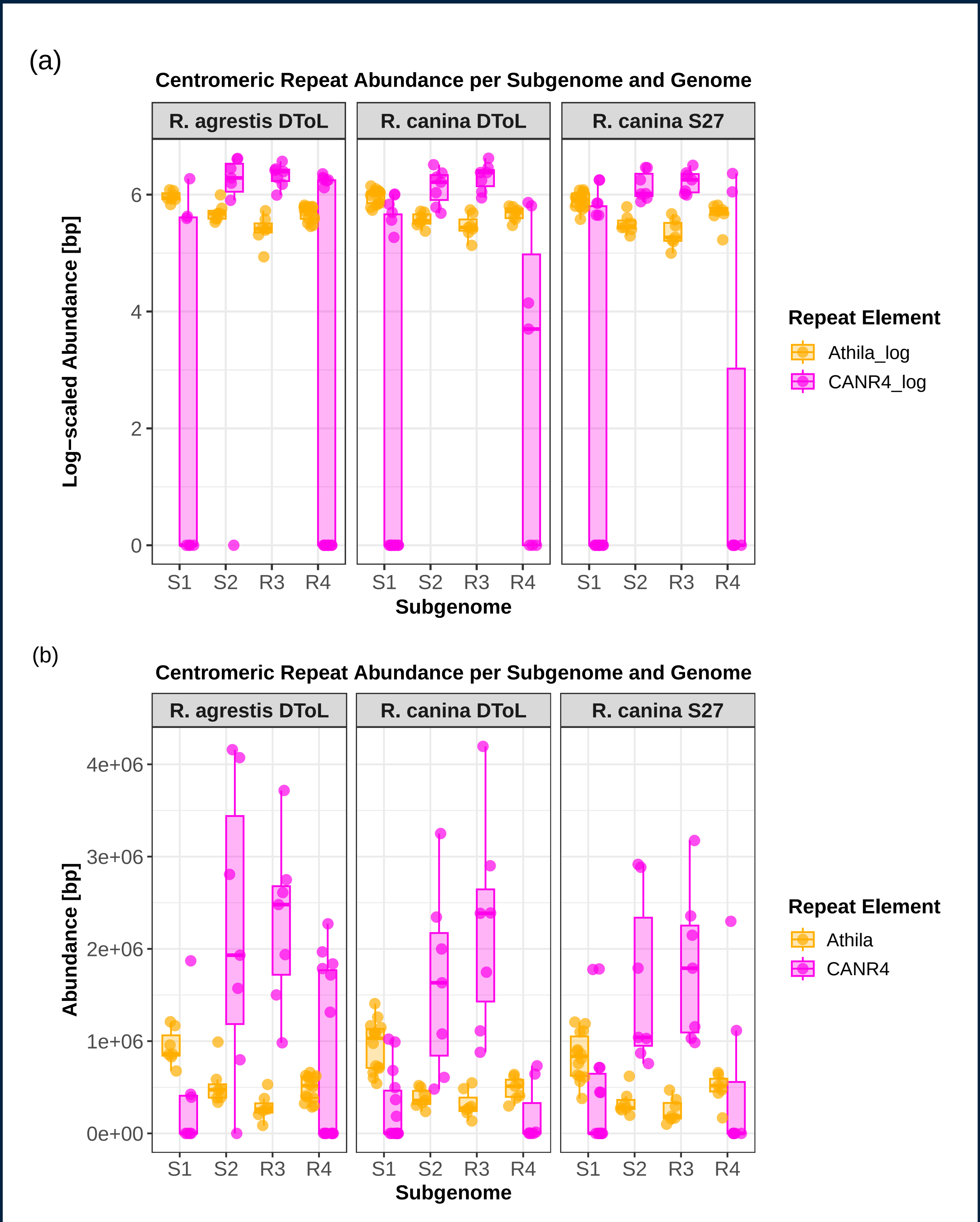

Note: Thanks to ChatGPT 4o with canvas for great help.
