# Supplementary material for: Bimodal centromeres in pentaploid dogroses shed light on their unique meiosis
Source: Nature. 2025 Jun 18;643(8070):148–57. doi: 10.1038/s41586-025-09171-z (PMC12222009; doi:10.1038/s41586-025-09171-z)
Supplement: Supplementary file 3 — Supplementary Data 1–17. [file 41586_2025_9171_MOESM3_ESM.zip › Suppl_Dataset_14_FINAL_cenLTRs.pdf]

cenLTR sequence consensus

>cenLTR1
TACTAATGACATTTCAGGGTTAAATTATGCGCTTGCTTAAAGCGTATCAATTTCCTTATTAAAACCCTGTTTAGG
AAAGTTTCCATTCTTTGATTGGGAAAGTTCCTATTTGTAGAAAGTTTCTATTTTTGTAGTTTCTATTTATCATTT
TTAGTAAGTTTCCATTTTCGGTAGTTTCTATTTTTCATTTTTAGAAAGTTGCCATTTTTCATTTTTAGGAAAGTT
TCTATTTTTCTTACTAGTTTCTATTTTCAGGACCTCCGAGCTAAAAAGTGGAAATGAACTCACAAATGGAAAGAG
GAGCTTGCGAACACCAAGGGGAGCAAAAATGAGGAACAATGAGCCACAGAAATGAGCAGAAATGAAGAAAAGAAG
TTTTCCTGCTTCAACCAGGAAACCCTGCGAAAAGGAGGAAAGCTTACCAATGAAGTTTCCAACGTTACTATGAAA
AAGATATGGAAAAATAAAGTGTTATCAACGTTGGAAGGTGACAAGGCTTGAATGAGAAGCAACAAGGCCCAAGAA
CTCTCAAGACTTATTGGATTTTCGGCAAATTGGATAAAAGCCCATAAAGGCCCATGCAATTAGGGTTTGTGACGC
AAGGTAAACCCTAGAGATGTTGTTGCCGTGAAATTTCAAGAGATAAACAAGGAGTTTGCCGTGGAAAATCAGAAA
TATAAAAGAAGATTTCGGTGGAGATTTTCTAGGGAGATTTTCTTGGAATGAAATATTCTTGGAGAATTATTTTGT
GTTGTTGCCGTGAAGAATTAAGAGAGAAAAGAGGAGGATTAACGTGAAACCCTAGAAGATGACGCCAAAGCAAGA
GAGTTTTAAGGAAGGAAGAAGTGTGGACAACAAGAAAAGCTCAAAACAAGACTAGATACATTCCTAAAATCCCTA
AAGGTAGTCTGGCCGAAATAAAAGAGAGAAAAATCAGAATATTTGCAAGGGAATTTCGGCAAGATTAATATTGGA
ATTTTGTGATTGGTTGGACCACCTCATAGGGCTTATGTGGCAAAATACTATTGGAGGAAATTATGTGGAGTTATC
ACATTGTTGCCGTGAGAATCCTTGGAGAAGTGCACGGCTTGGAGGCCAAGCATTGCCGTTCTCTATATAATCCCT
TCATTCTCAACGTCAAAGGGTTCCTCTCCCTTACATTTACACTTGGAGAAAAGTTCAGAAAATACCCTAGCATAG
CCGTGAGCCTCTCCATCCTCTAGTCATCTCCACGAATTCAAGCAAGGCCAAGGGAGAAGAAGAGAAGCCGTGAGC
ACCACCATCCGTATCCATTCTTGAAGCTTGCTTTCGAGTTTCAAGAAACAACAACAACGTTCACCCATCTCATCT
TCATCCACGGTGTAATCCGATTCTCCTTTGTAACCTTTGCTTTGAATTTCGTTGGTTATGAACTAGTTGACATAT
ATGTATGTTTGAACAAGATTTTAATTTCGAATTCTATGGTTGAATAAATTTCAGATTCTATTATTGCGATTCATT
GTTGCTTTTGTGTGAGTGTTTGATTAAATTTGCATGATAGATATCTTTTGTATGTTAATTCTAAATGGTTCGAAA
CTTTTAGGGTTTTTATATGATTGGTGCTACGAATTTAAGAACATGAATCAACTTTTTGGTTTTGTGTTCTTAAAT
CGATAAGTAGTAAAGGTTTTGTGCAAAAATCGGGTTTAATCAAAGAGAATTGCAATTAGGTGGACTTTTTCATAC
TAAGTTGCACACTTGCGTTGATAGCCTTTCTAGGTGCTTATTGCGTTAACCATGTATGATTGACTAGCTTTCTAG
GGCTTGAATGCATGTTTGATAGGATTAGTCTTTGTGCTTTCACTTAGATTAATTTAGCATTGAAAAGTAAAATAT
GGGAATTGTTTGCTTTCTAACAATTTACATGATCAACTCCTCTTGCATGACTTTGATGAACAATATAGAACTTGA
ATCGATTTTAATCATAGTTTTGGTTTTAATCTTCGTTTTCTCATTCCATTCGTATTTTTATGTTTTTGCATTTTA
ATTTATTTTCTTAACTTAGTTTATTTTTCGAAAACCAAAATCAATCAAAAATATCCCCCCTTTTTCGTAAATAGT
GTATATATGTGTGAATATTATACTTTGTTTTAATTTTAATTGTTTAATAGTTTGAAAATGACAGGTGTACCCTCA
ATCCCCGGAATAGAACGATCCCTATTTACTTATAC
>cenLTR2
TTAAAAGACCCCATTATGAGAGGAATTAAGGCCATAAAGGAGACGTTTTCAGTTGGAAAATTAAAGGAATTATGA
TGCAATCCCATCCGTCCAATGATGAAGGGTATGATCGACAAAAGCCAACCAATGCTCTCCTATAAATAGGCAACG
TCTAGAACAGAATTTCCATCACTTCCTAGCCAAGATCACTTCCCAGCCTATCACTTCCTAGCCAAGATCACTTCC
CAGCCTATCACTTCCCGGCCTATCACTTCCTAGCCTATCACTTCCCGGCCTATCACTTCCTGGCCAAAAGCTATT
CCAAGACTCTGCCCAATTCACTTCTCAAACCTCCAACACCTACAAGACCGTGACACCATCCATCCATCAATCTAC
GAAGTTCTTAGGCGCTGAGTCAAGGACGCTCCACCACCATAGCAGAGACGAGTTCATCACCTTGTTGCTAAGCCG
CTGAGGAAAGCTTCAAAGTGTAACTATGACTCTACTTACAATTTTTATTTCGGTTTTGTGTGTTTGGATTTGCGA
GTTGTGTAATTGGAGACATGAGATTTTCAGAATATTTTTATTAATATTTTTGAGATTTTCAGTTTATTCAATTAA
TTTCGAGAATTCTTTTATGATGCATGTTACAATCTTGTGCCCTTTTATGTGTTTAGGTAATTTTCAGAGTTAGGT
TCATAAGTTTGCATGCTAGAATAATGGTGAGAGTTCTTGTGTGTTTGCTTAATTTGCCAAGAGTAATTATTATTT
GTTAAGGCGCTGAGTTAAACAAGTAGTAATTAGTTCTAATGGGTGGTAAAAATCATGTCTTAATGATTAAATGAT
TCTGGAAATTACGTGTTAAATTCTATGTGTAATGGTTAATTTGCACGTGTGAGTTGATTCGAGGGTTAGATAATA
CTACTACTTAAGAGAACTACGCTGAGTGTTTTCGAAAATTAGTAGTATTAGGCTTGGTAAGGACTTTTCCGATCC
AAGCCTACATTAGAACGAATCAGATAAATGGATTGATCCGCTGAGGCTTTTCATTTGGGCTCTTATCTATGCATT
CAAGATGGGCACAGTTTTGTAGCATGTTGAAATGAATTTCTGAGTGGTATTGGTCTAGGTTAGGGAAGTCGATCA
TTGTATATAGTTTTATTTGTTTTGTTTTTATTTTAAGTGGATTAGGAACCAAATCTCAAAACCCCCATTTTATTC
TTTTATTTGTTAATTGACCTTTTTGTGTAGGTGTACCCTACAATCCCCGGACTGAACGATCCCTGCTTATCCTAT
ACTGACAACTACATTTTGTAGGGTTAAATTGTGAGGCTATTTTAGCCGCATAATAAATAAAGGAGAAAGACGTTT
CAGTTGGAAAATAAATAAGAGAAAGATGTTTTCAGTTGAGGAATAAAAGAGAGAGACGTTTCAGCTAATTGAAAA
>cenLTR3
TTTTTTTTTTTTTTTTTTAACATGTTGTAAATTGTATGTGTTTCACTTAGATTAAGCATGTTGTAAATTGTATGTG
TTTCAAGTAGATTAATCTACTTAGGTAGTCATGAATTTAGTTAAATACTTAATTGTTGTCAGTGTGTAAAATCGT
ATGAGTAGACAAGGGCCCTTTTGTTAATATTGGCCTAAACCCTTCATTAGTACCTTGCGAAGGTCTCTTGAGGTT
GAGGGCGGCCTTTATTCGCACTTGGAGAACGGTCTAACACATAAGTTAATTTCCCAGCTGAGTAAGGGGCACATA
CGGATGGTGTCTTAGATTGGGACCCTGGGTGATGGGTTCAATTGGATCTCAGAGATACTATAGAATGAGCGTAAA
CCACTATGTGGGAGTAGTTGATAGGAGCATAAAGTGCGACGATATTATTGAGTATATGCCCCATTTTAGCCTTGT
TTCTCCCTTAAGTTTCGTGTTTTGAGTCATCGAGTCATTTTAAGAGTATTGTAGAGTGTTTGGCGTGAAATAAGC

GGAAAAAGATAAATTCATGAAAAATCCTAGCTGGATCAGGATTCCTCACTGTCGACTGTTTTTGCGGTCTTTACA
TTTTTATTCCCTTTATTTTCTCTAGAAAATTCTGATATTCTCCTGCTTGTTGTAGGAAACCTTGGCTGGTGGAAC
TCTTGGAAACAACAACGATGGAGTCATAAAATAAAGCATTTAAGACATTTGACCTAGCAATGAAGAAGGGAAATA
AAGGAGAAAGACGTTTTCAGTTGGGGAATAAAGGAGAAAGACGTTTTTCAGTTGGGGAATAAAGGAAAAAGATGT
TTCAACTGGAAAATAAATAAAGGACAAAGACGTTTCAGCTTGTTAAAAGACCCCATTATGAGAGGAATTAAGGCC
ATAAAGGAGATGTTTCAGTTGGGAAATTAAAGGAATTATGATGCAATCCCATCCGTCCAATAATGAAGGGTATGA
TCGACAAAACCCAACCAATGCTGCCCTATAAATAGGCAACGTCCAGAATAGAATTGGTATCACTTCCCGGCCTAT
CACTTCCTGGCCTATCACTTCCTGGCCAAAAACTATTCCAAGACTCTGCCCAATTCACTCCTTAAACTTCCATCA
CTCACAAGACCGTGACCACCATCCATCCATCATTCTACGAAGTTCTTAGGCGCTGAGTCAAGGACGCTCCACCAC
CATAGCAGAGACGAGTTCATCACCTTGTTGCCAAGCCGCTGAGGAAAGCTTCAAAGTGTAACTATGACTCTACTT
ACAATTTTTATTTCGGTTTTGTGTGTTTGGATTTGTGAGTTGTGTAATTGGAGACATGAAATTTTCAGAATATTT
TTATTAATATTTTTGAGATTTTCAGTTTATTATTGAGTTAATTTCGAGAATTCTTTTATGATGCATGTTACAATC
TTGTGCCCTTTTACGTGTTTAGGTAATTTTCAGAGTTAGGTTAATAAGTTTACATGCTAGAATCACGCTGAGTGT
TCTTGTGTGTTTGCTTAATTTGCCAAGAGTAATTATTATTTGTTAAGGCGCTGAGTTAAACAAGTAGCAATTAGT
TCTAAAAAGTGGTAAAAATCATGCTTTAATGATTAAACGATTCTGGAAATTACGTGTTAAATTCTATGTGTAATG
GTTAATTTGCACGTGTGAGTTGATTCGAGGGTTAGATAATACTACTAGTTAAGAGAATTACGCTGAGTGTTTTCG
AAAATTAGTAGTATTAGGCTTGGTAAGGACTTTTCCGATCCAAGCCTACATTAGAACGAATCAGATAAATGGATT
GATCCGCTGAGGCTTTTCATTTGAGCTCTTATCTATGCATTCAAGATGGGCACAGTTTTGTAGCATGTTGAAATG
GATTTTCTGTTTTTACACTTAGTAATTTCCGAGTGGTATTGGTCTAGGTTAGGGAAGTCGATCATTGTATATAGG
TTTATTTGTTTCGTTTTTTTATTTTAAGTAGATTAGGAACCAAATCTCAAAACCCCCATTTTATTCTTTTATTTG
TTAATTGACCTTTTTGTGTAGGTGTACCCTACAATCCCCGGACTGAACGATCCCTGCTTATCCTATACTGACAAC
TACATTTTGCAGGGTTAAATTGTGAGGCTATTTCAGCCGCATCAATTTTTGGCGCCGTTGCCGGGGATTGTTAAA
ATCCCTAGCGCTTAAAGTGTCATCGATTTTGTTGTTTATATAGAATGCATGCTCAATTTTTGTACTATACTTGTG
GAATGGTGACAAATTGCGATTTATGTTAGGCCAAGCTATAATTGTGATTTAGTTTAAGTTTTGTGAGTGTTACAA
AATTAAGCATGTATTTTATAATTGTTGTACAATTTGTAGAAG

>cenLTR4

GGACAAGGAATCCCTGTGGGATAAGGATTACTCAAGAAGGTTCCGGAAGAGTGTAGAAGCATCTCGGAAAAGGAA
GCAGTATTTGACTAGGAAACCTACTTGGATATGGAGTTCTACTCATACTAGGAGAGCTATGGAACGTTCATGAAG
CATCTAGAAGTCTCAAGAAGGTCATGTGCCGTGCACATGGAAGAAGAGTACACTTTGGATTCGGTTTTGAGAGAA
ACAAGGAGTTGGAATTCGAAGTGGAAAAGGATTGAGGAATTTCCGTGAGATTCTTGAAGGTTCTTGAAGGTTCTT
GGCGTTTCATTCTTCAATAAGGCTTGGAAGGCATGTGCGAGGCATGTGATGTGAAGGAAAACCTAATTGGACACA
ACTTGAGCTTTGAAAGTCAAATCCATTTCAGATTCGGATTACTGCCTTTGCAGATCAGTCTACTTCAACGGAGCA
TAGAAAATCACTCAGAGCTCAGAAAATTATGATCTTTATATGGTTGGAAAGCTACGGATGTCTATTTTCCAGAGC
TTTTTACAGCTTGTCAATAGCTATTTTCTAGAGGAAGTTATGGCTGTTTTAGTGCACTGAGGTCAAGACTGCCGG
AAATCTGCTTTGTGCCAAAGAAGTCCTAAATCAACACAACTTTGGAGAAACCAAGTAGAAACGAATTGGGTGAGC
CTTGGGACGTCTTCCTATATATACCTTGTGTATTAGACGTTTCAAGGTTACCATCTTTTCTCCACAATTTCGTAT
TCTCACTTGTGTGCTCTCAAGTTTTCAGGTTTTCACGATTCTTCAAGGAGCAAGCCGTGCCATAACCCTCCACTT
CCGTGTTCATCACTTGTGCACCATCTTGGATCTGCGTTGGACCTTGGGACGTGACTAAGGGTTGTCCATAAGCCT
TATCATACATGTACTTCACGGTTTTGTGTAATTGTTGAATAGATTTTCTGCTTTGGATTATCTATGTGTAATTTG
AATCTTTTGAGTATGCAAAACTGTTTTCGAAGTATAATTTTGATGTCTTTTCATGTTTGATTTGTCTTGAGTGTT
CTTTGTCTTGATTGATTGCCGAATTGATTGAATGATATTCTGATTTTGTGCTTCATTACATGTTAATGATTGACG
TGGTTCGAAACATAGATGACTTGCATGTAACTTGCTAGTAATTAGGTTTATGCTTGGTAAACTCTAATTCGATTG
AGTAGTAAAGGCTTGCTTTGAGTCGGAAATCAGATTATGCATGTGATGATGAGTAGTGACGCCCTTGGATTTGCA
TGTTCATCAATTCTTTCTTGAACTTAATGATTTGAAAACGAATTTTCAACGTATGTGTGTATTCGGGTTTTCAAA
ATGTATTGACTTAGTGCTTGCTATTTCGATTAACTTAAGAAAGAAAGTTAAAAGGGACATTGGTTGCTTTGATCT
TTGTGTCTTGGTTGATGGCGTTCTTGGTAATCAAAAGAAAGATTAAGGGATGCATGAAACATTGATTCTTGATGA
GTTCTTGATAAATTGTTTTCGAAAATTTTTCTTTTCCCACCTATGTAAATAAAATGTTTTTAATCTTTTATTTCT
TTTCTGAAAATTTATAGTTTAAATCTTTAAAACCCCCCCTATCTTTATGTTTTCTTGTTTGTGTAAATATTTAAA
ATCGGAATTAGGTGTATAGGATTGCATGATCACGTTAATTAGTAAGTTAATAATCTGAAAATACTTAGTTAAATC
GTAAATAATTAAAATGTAAAGTGTTTTTGCCGAAATATACCTTAGGTGTTTCTTAGGAATTGTTTTGGTGGTTTT
TAGGGATTCTTTTGGTGTTTTTTAGGGATTGCTAAAAAATAGGGATTTCTTTTGGTGTTTTTTAGGAAAAGTAGT
TAGAGTTAGTTTTAAACATGGAATCCTTGTTTGACTTGGTCAATAATTTGTTTCCTTGTTGTATATGGATTCCCT
ATGTGATATGGATTTGTAAATTGTGTATAAATAGGAGTAGGATTCCAAAACCTTTTCTAACTTGTTTTCTCTCTT
CTTTTCGAATTGTCTATATATACTTGTGTATTTTGATTGTTTCTCTCATTCTTTTGTCTTCTAATTTCGTGTGAC
ATGTATGTTTTCTCTCTAATTCTTTCTTTGTTGTTTCTCTAATTTTCGTGTAAACTTGTAAATGTTCGTATAATT
TTCGTAACTTGTATATTGTTTTATTAATTTGTTTCTCACATGTTAGCACCCTCAATCCCCGGTTTAGAACGATCC
CTGCTTATCCTATACTAACGCTCGACATTTTCAGGGTTTAAATTGGTGATGCTTTTGCACGTATCAATTGGAGCA
TAAAATGCGACGTTTATAATGTTTAAACCCTATATTTTGCTAAGTTATTTCCTTTATCTTGATCAATTTAACTTA
GTTAGTGTTTTGCAGGTAAAATGGAGTCTCAAAGTCCAAAAATGGCTAAGGAAGGCACAAGTGGCGCAGAAATGA
AAAGAAATGAAGAAATGGAAGTTCTCCCAGTTTGACTAGGAAAAGGAATCCTAGTTGGAGTAGGAATCAGAAGCT
GACTTGGATTCAAACTCTTAAGTCGCGGAAATTAGAAGTTCTACTT
