# Supplementary material for: Bimodal centromeres in pentaploid dogroses shed light on their unique meiosis
Source: Nature. 2025 Jun 18;643(8070):148–57. doi: 10.1038/s41586-025-09171-z (PMC12222009; doi:10.1038/s41586-025-09171-z)
Supplement: Supplementary file 3 — Supplementary Data 1–17. [file 41586_2025_9171_MOESM3_ESM.zip › Suppl_Dataset_3_Chr_SCOs_FINAL.pdf]

Supplementary Dataset 3. Maximum Likelihood phylogenies per synteny group. Maximum Likelihood phylogenies of the genus *Rosa* retrieved from our chromosome assemblies and the DToL *R. canina* and DToL *R. agrestis*, sect. *Caninae* pollen samples and diploid rose species based on single copy orthologous (SCO) gene set calculated for synteny group 1-7 separartely (a-g) separately. All nodes which were noit supported by 100% bootstrap are indicated as dashes lines. The *Synstylae* clade and *Rosa* clade are indicated in violet and orange branches.

a

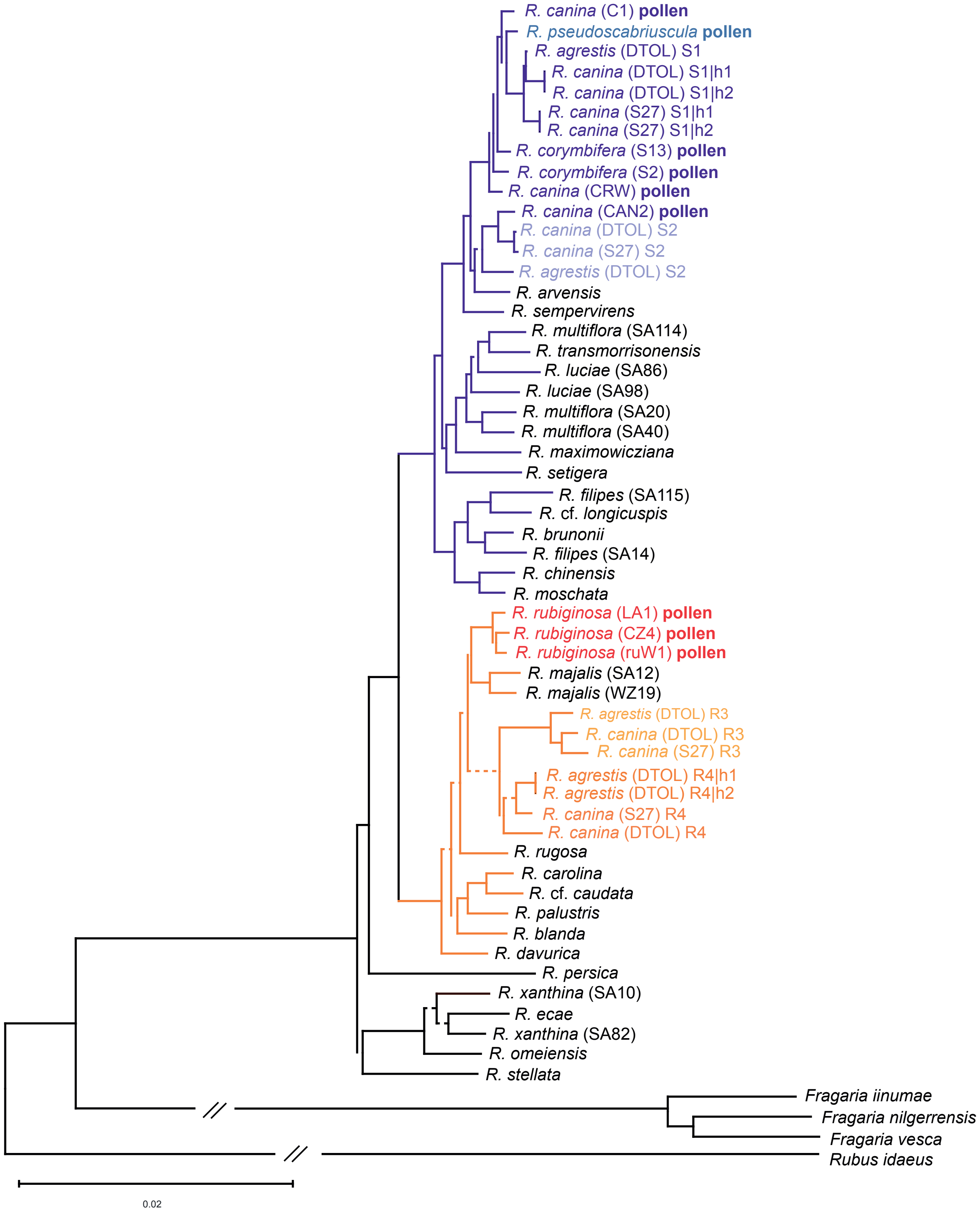

synteny group 1

b

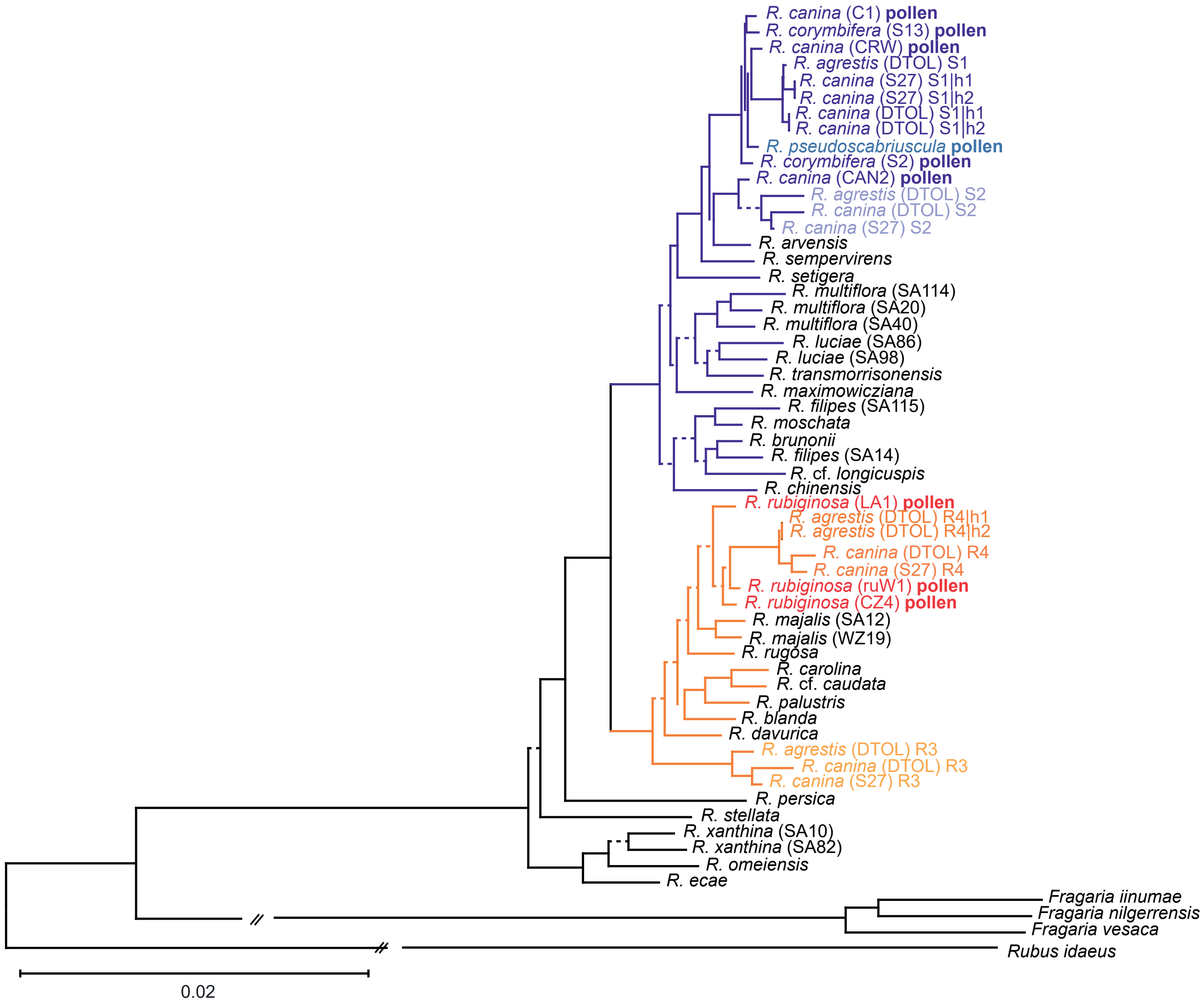

synteny group 2

C

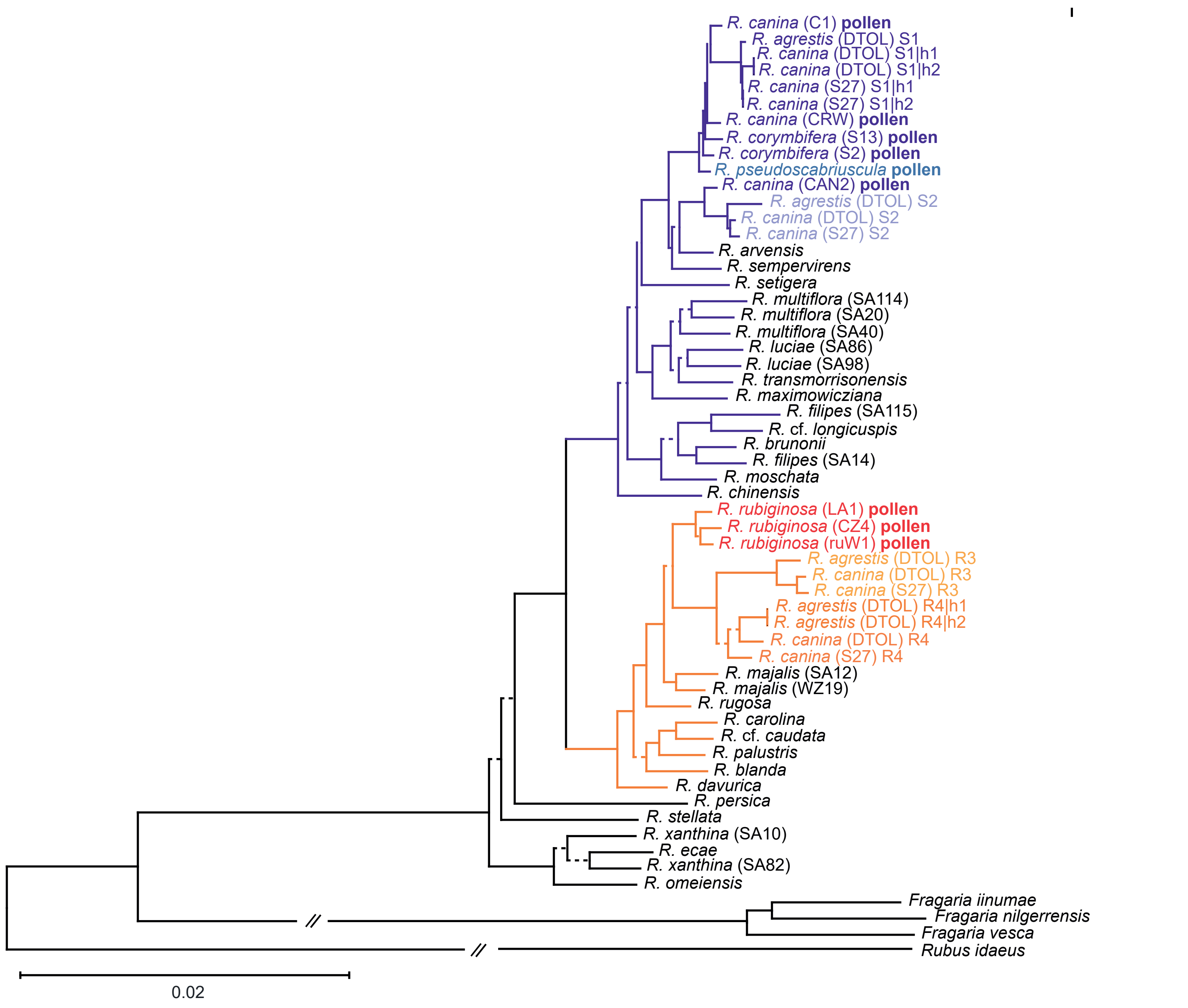

synteny group 3

d

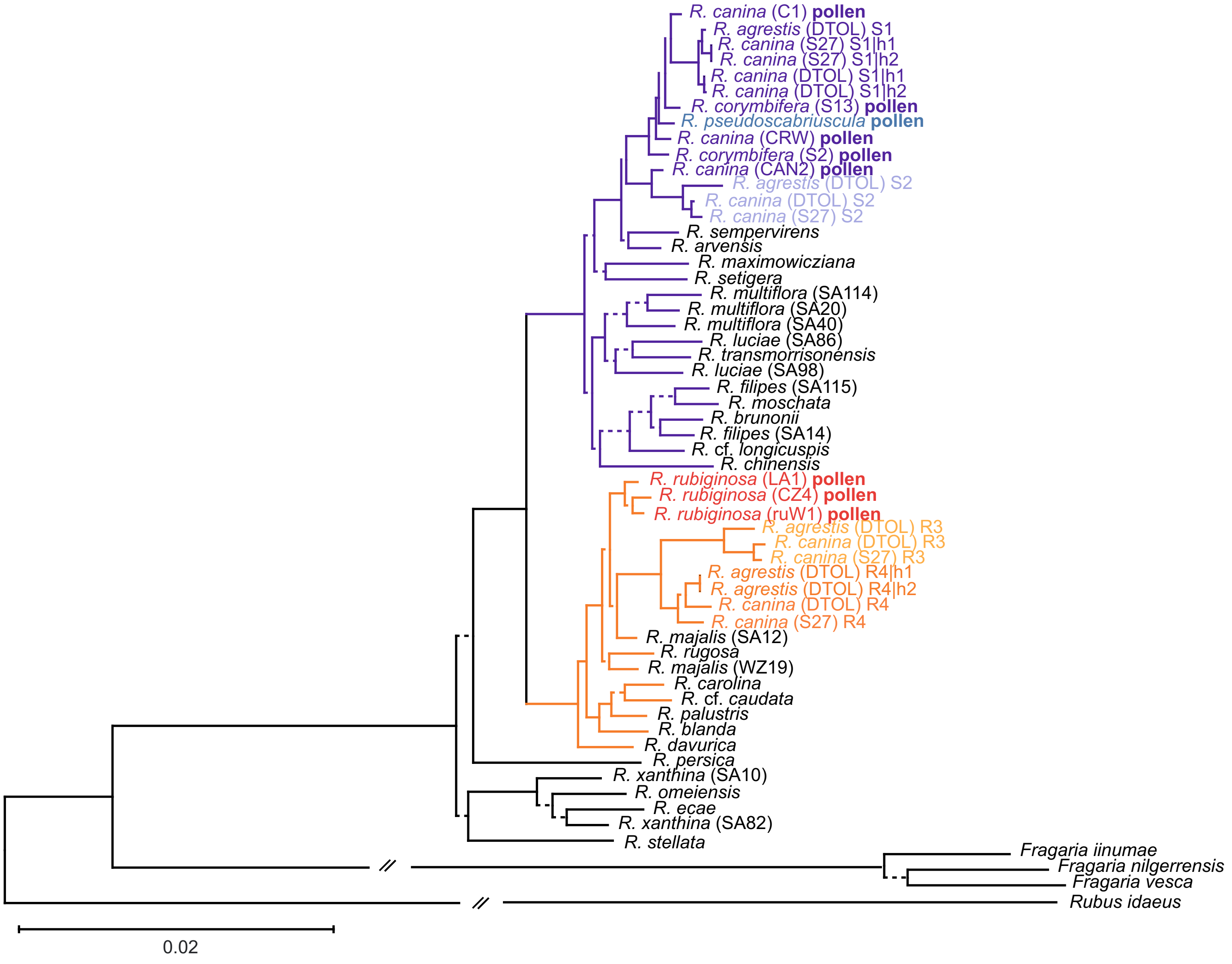

synteny group 4

e

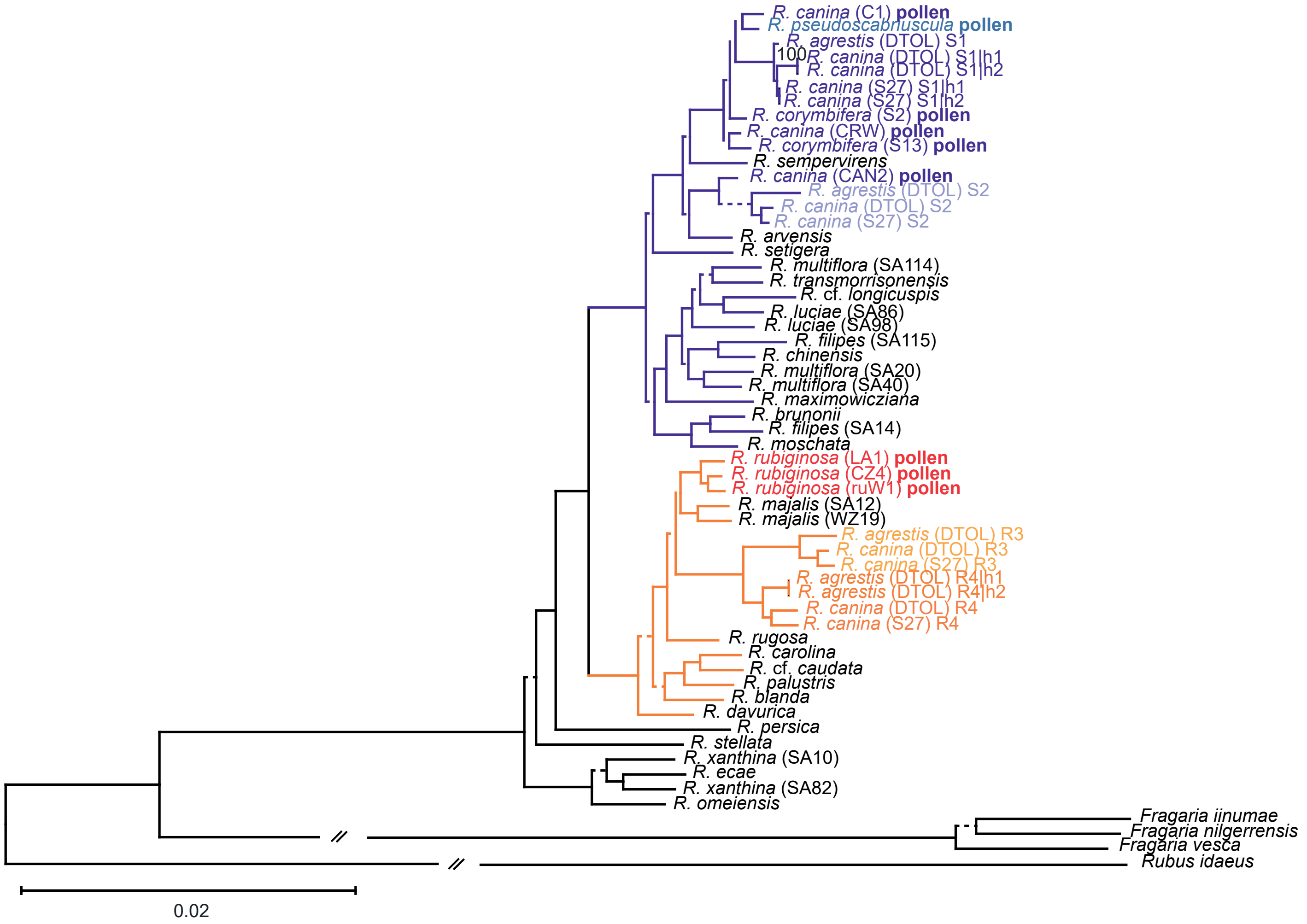

synteny group 5

f

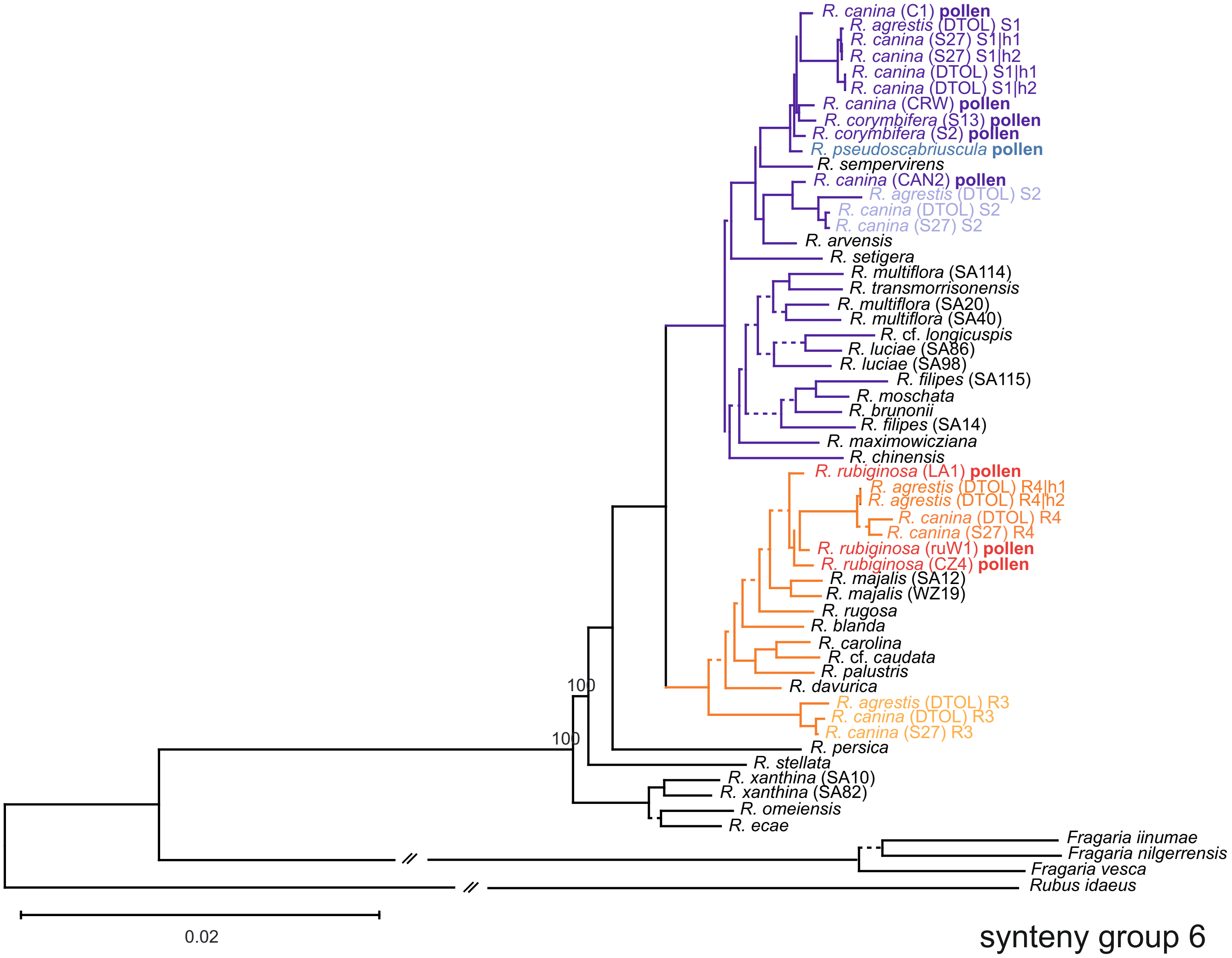
R. canina (C1) pollen
R. agrestis (DTOL) S1
R. canina (S27) S1|h1
R. canina (S27) S1|h2
R. canina (DTOL) S1|h1
R. canina (DTOL) S1|h2
R. canina (CRW) pollen
R. corymbifera (S13) pollen
R. corymbifera (S2) pollen
R. pseudoscabriuscula pollen
R. sempervirens
R. canina (CAN2) pollen
R. agrestis (DTOL) S2
R. canina (DTOL) S2
R. canina (S27) S2
R. arvensis
R. setigera
R. multiflora (SA114)
R. transmorrisonensis
R. multiflora (SA20)
R. multiflora (SA40)
R. cf. longicuspis
R. luciae (SA86)
R. luciae (SA98)
R. filipes (SA115)
R. moschata
R. brunonii
R. filipes (SA14)
R. maximowicziana
R. chinensis
R. rubiginosa (LA1) pollen
R. agrestis (DTOL) R4|h1
R. agrestis (DTOL) R4|h2
R. canina (DTOL) R4
R. canina (S27) R4
R. rubiginosa (ruW1) pollen
R. rubiginosa (CZ4) pollen
R. majalis (SA12)
R. majalis (WZ19)
R. rugosa
R. blanda
R. carolina
R. cf. caudata
R. palustris
R. davurica
R. agrestis (DTOL) R3
R. canina (DTOL) R3
R. canina (S27) R3
R. persica
R. stellata
R. xanthina (SA10)
R. xanthina (SA82)
R. omeiensis
R. ecae
Fragaria iinumae
Fragaria nilgerrensis
Fragaria vesca
Rubus idaeus
100
100
0.02
synteny group 6

g

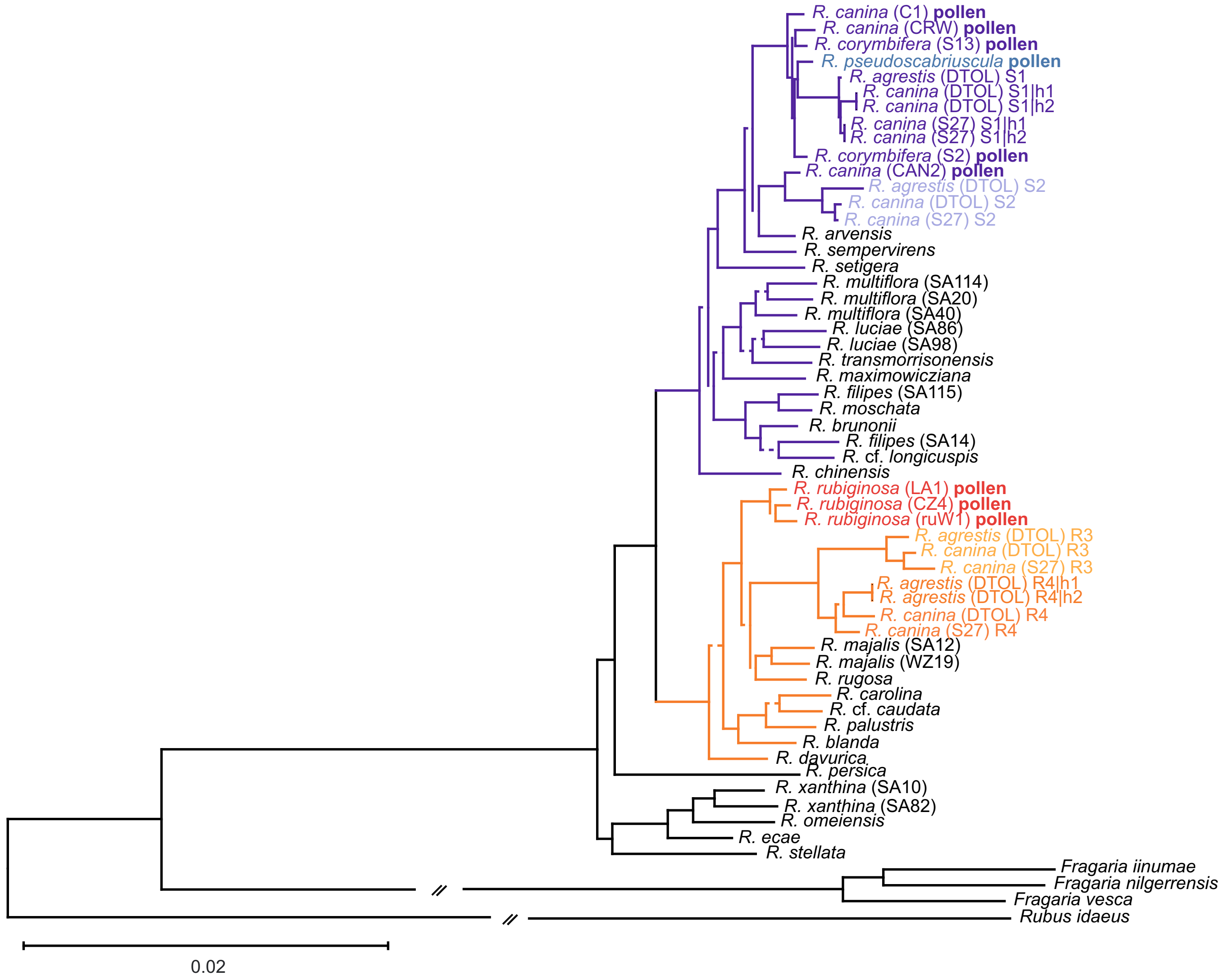

synteny group 7
