# Supplementary material for: Bimodal centromeres in pentaploid dogroses shed light on their unique meiosis
Source: Nature. 2025 Jun 18;643(8070):148–57. doi: 10.1038/s41586-025-09171-z (PMC12222009; doi:10.1038/s41586-025-09171-z)
Supplement: Supplementary file 3 — Supplementary Data 1–17. [file 41586_2025_9171_MOESM3_ESM.zip › Suppl_Dataset_4_DANTE-LTR.pdf]

LTR characterization of the *Rosa canina* pentaploid genome

DANTE LTR Summary.

Lineage	Number of elements	Element mean length [bp]	LTR mean length [bp]	Number of elements with PBS & TSD	Number of elements with PBS only	Number of elements with TSD only
Ty1/copia|Ale	5647	5127	183	1039	984	215
Ty1/copia|Alesia	15	4867	358	0	0	0
Ty1/copia|Angela	476	9816	1848	299	75	5
Ty1/copia|Bianca	16708	6834	218	12056	2269	396
Ty1/copia|Ikeros	1707	7048	491	1133	259	32
Ty1/copia|Ivana	803	4685	282	530	172	23
Ty1/copia|SIRE	40	9331	874	24	11	0
Ty1/copia|TAR	356	5744	592	21	29	181
Ty1/copia|Tork	820	5170	511	373	247	31
Ty3/gypsy|chromovirus|CRM	496	5622	474	315	86	18
Ty3/gypsy|chromovirus|Galadriel	288	5831	527	183	39	1
Ty3/gypsy|chromovirus|Reina	581	5482	278	317	87	63
Ty3/gypsy|chromovirus|Tekay	433	9563	2368	153	140	11
Ty3/gypsy|non-chromovirus|OTA|Athila	2795	12333	1845	743	447	715
Ty3/gypsy|non-chromovirus|OTA|Tat|Ogre	1600	14478	814	1009	259	101
Ty3/gypsy|non-chromovirus|OTA|Tat|Retand	3528	11884	507	2482	424	158

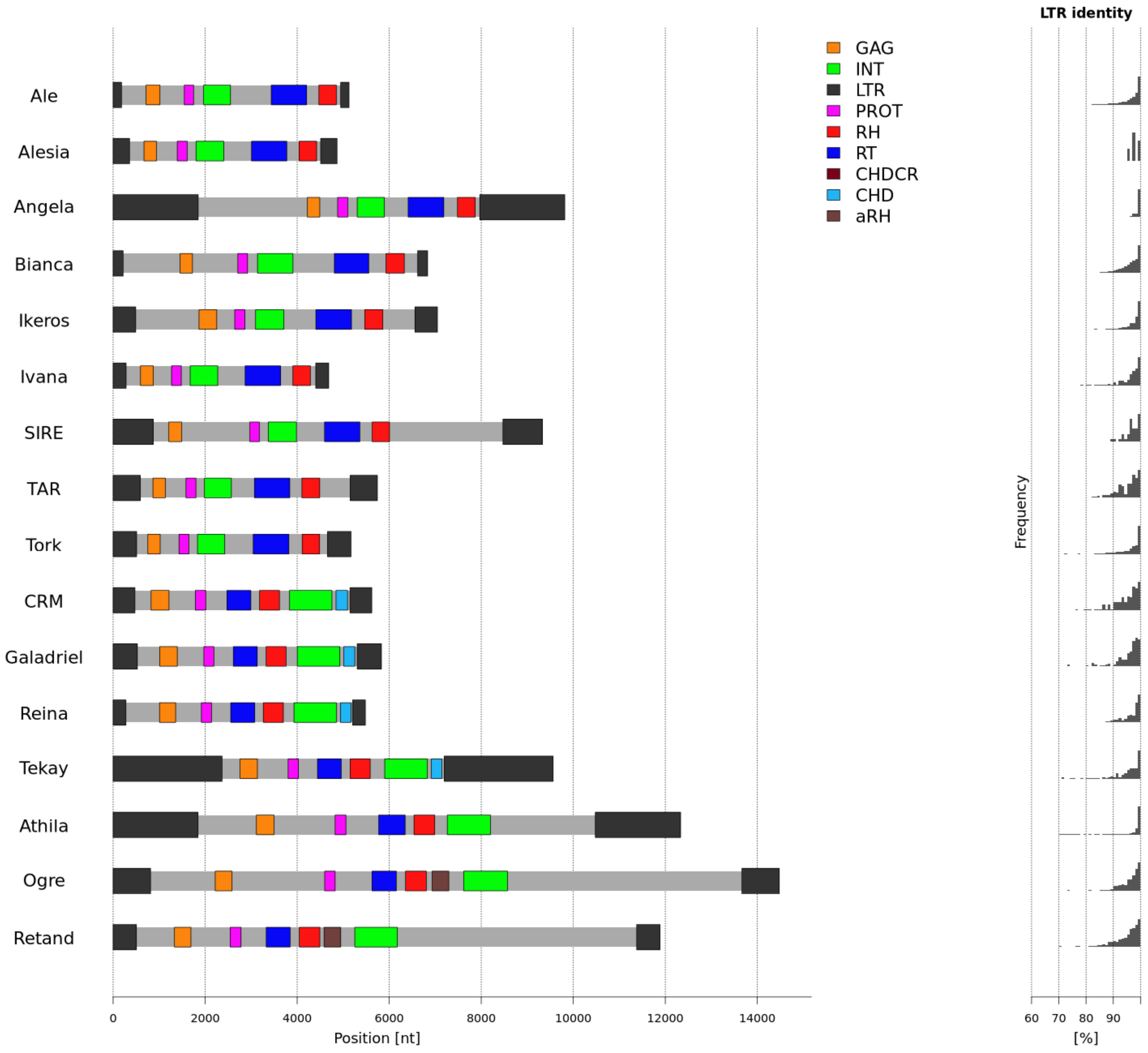

LTR elements composition and identity found in the *R. canina* genome.

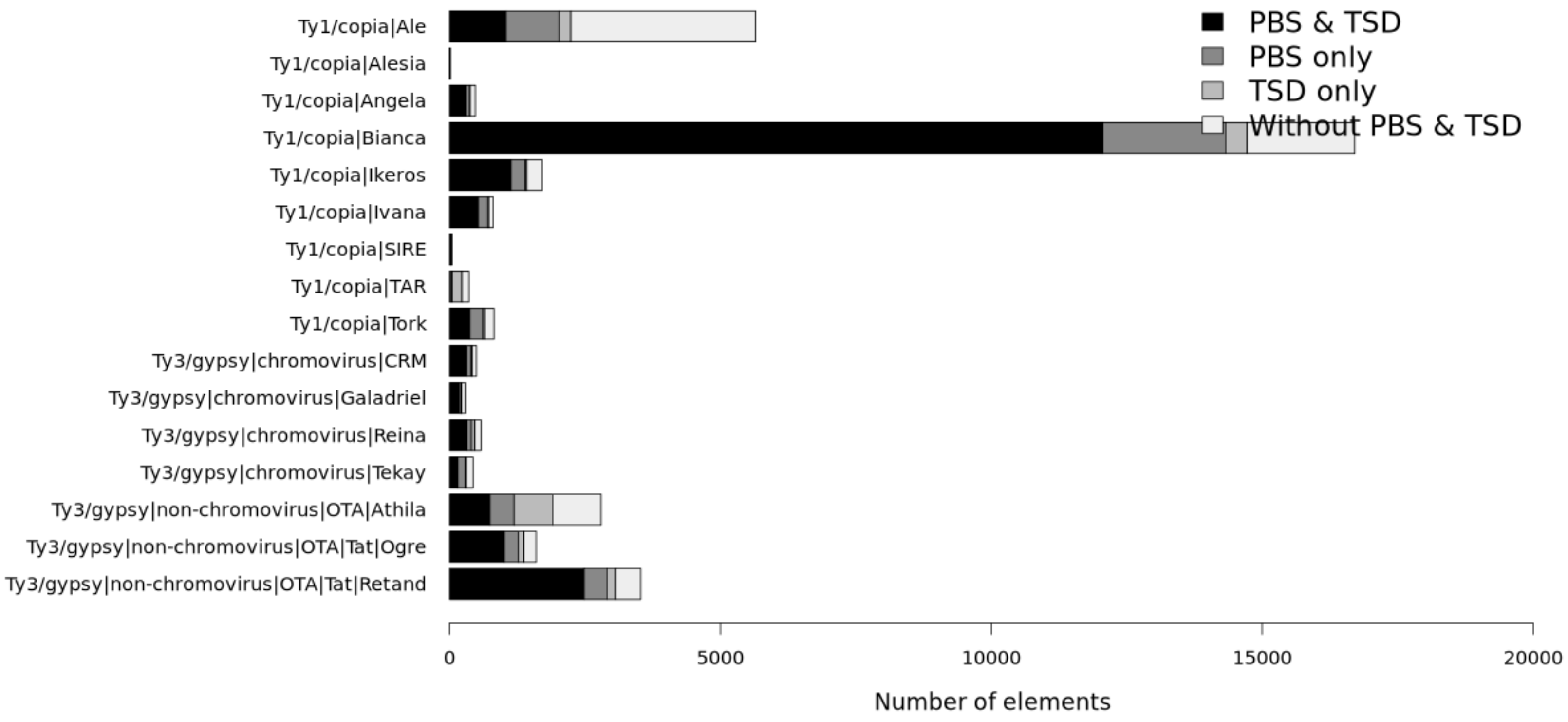

Number of LTR elements found in the *R. canina* genome.

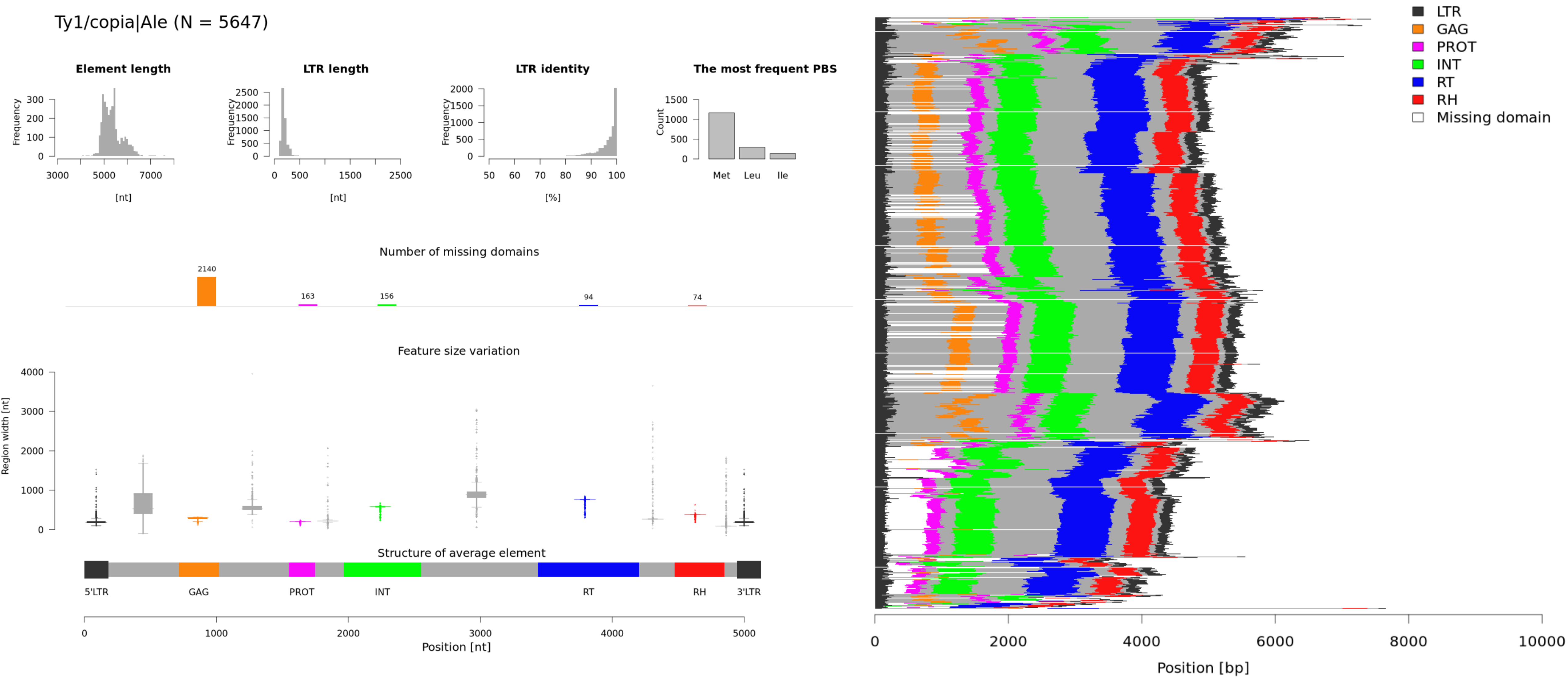

Composition and identity of LTR *Ty1/Copia ALE* elements found in the *R. canina* genome.

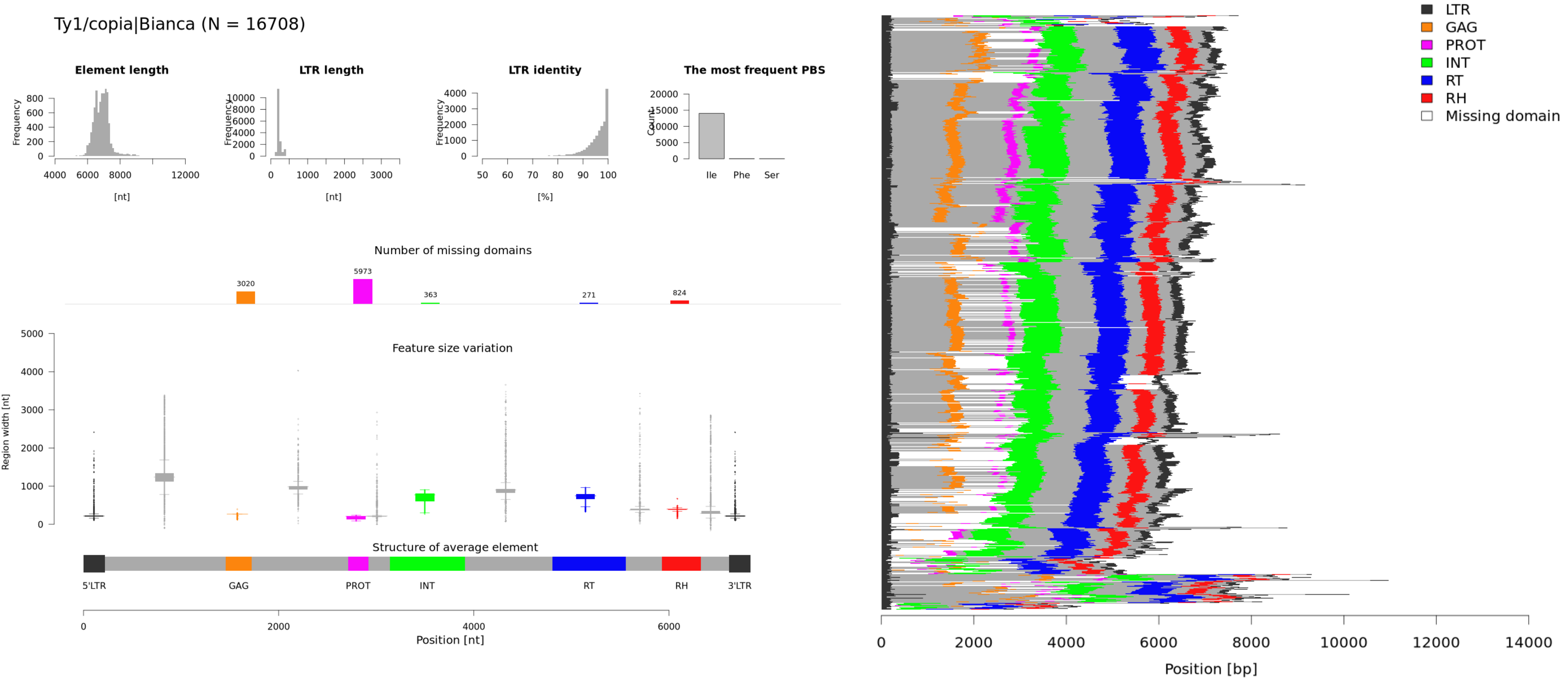

Composition and identity of LTR *Ty1/Copia BIANCA* elements found in the *R. canina* genome.

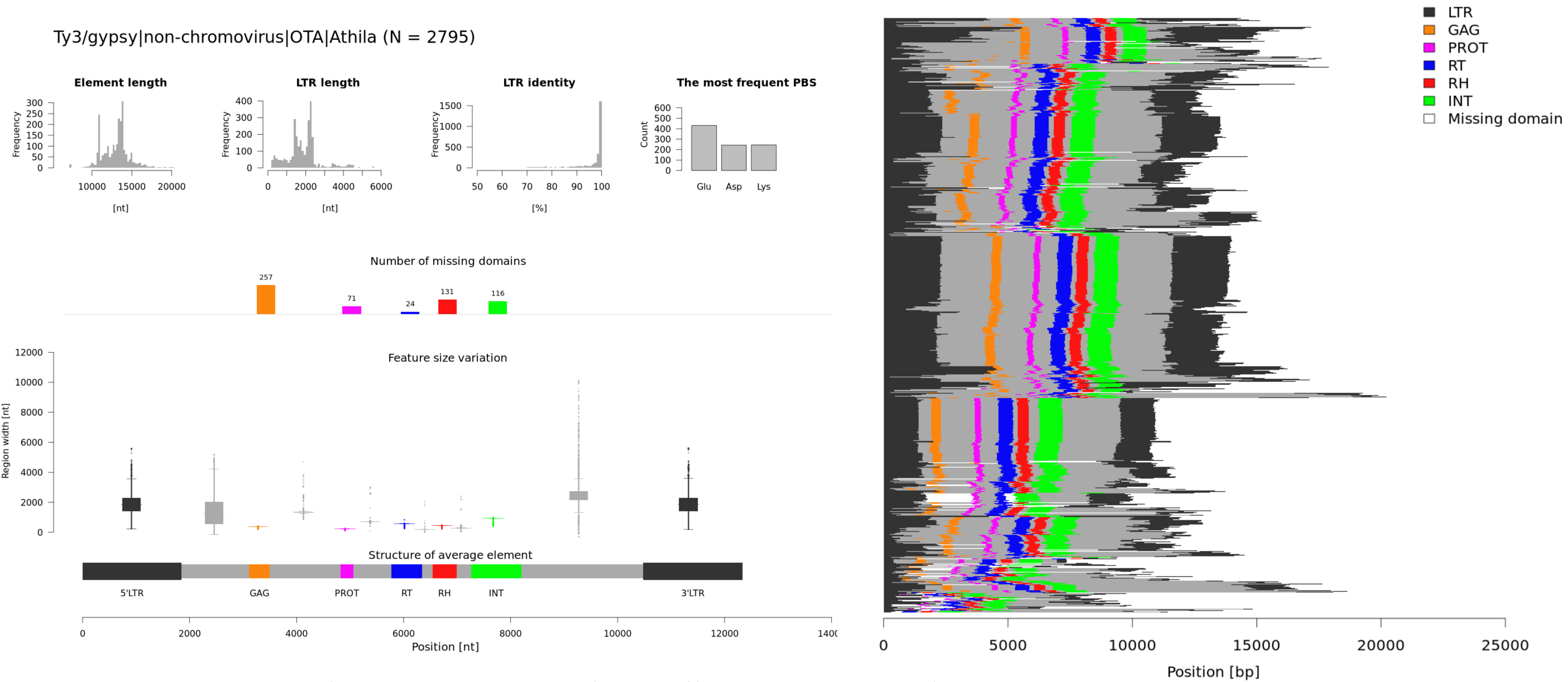

Composition and identity of LTR *Ty3/Gypsy ATHILA* elements found in the *R. canina* genome.

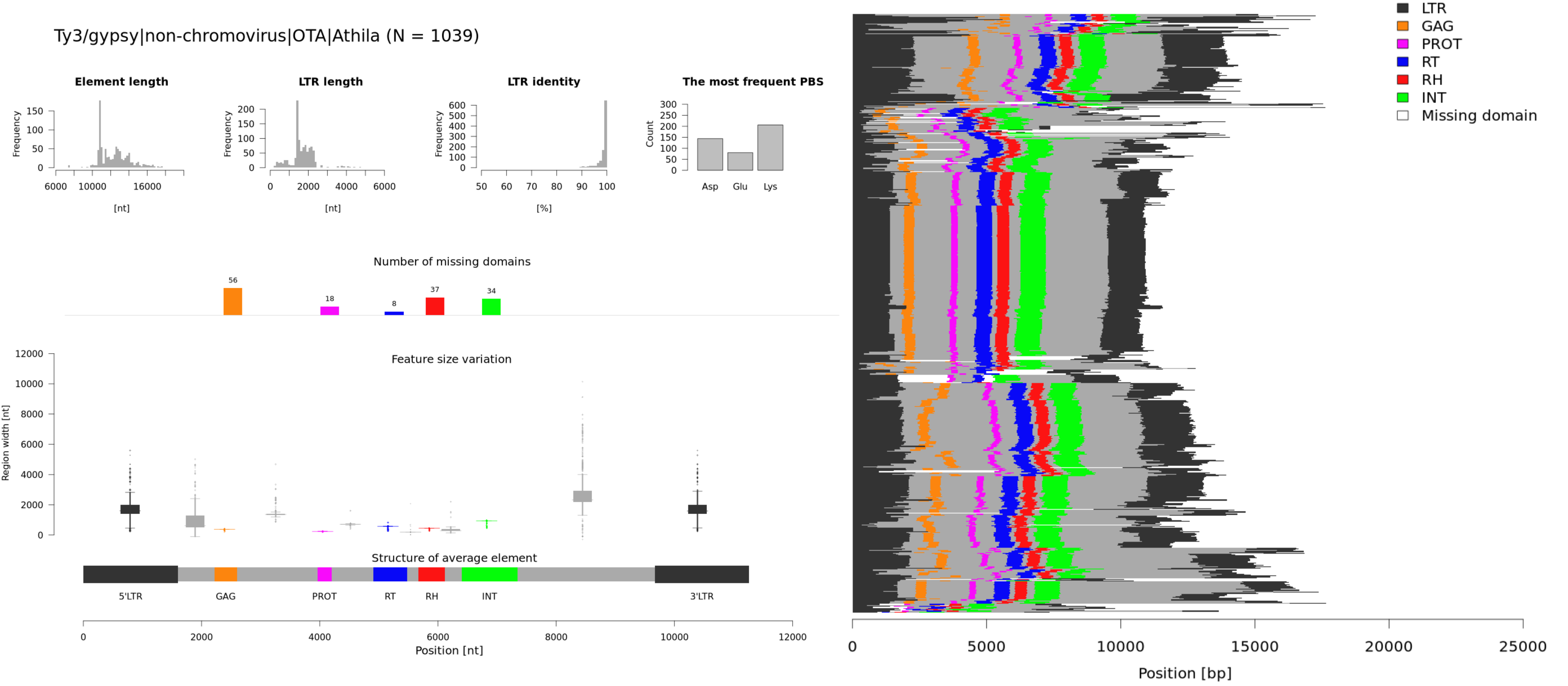

Composition and identity of NON-CENTROMERIC LTR *Ty3/Gypsy ATHILA* elements found in the *R. canina* genome.

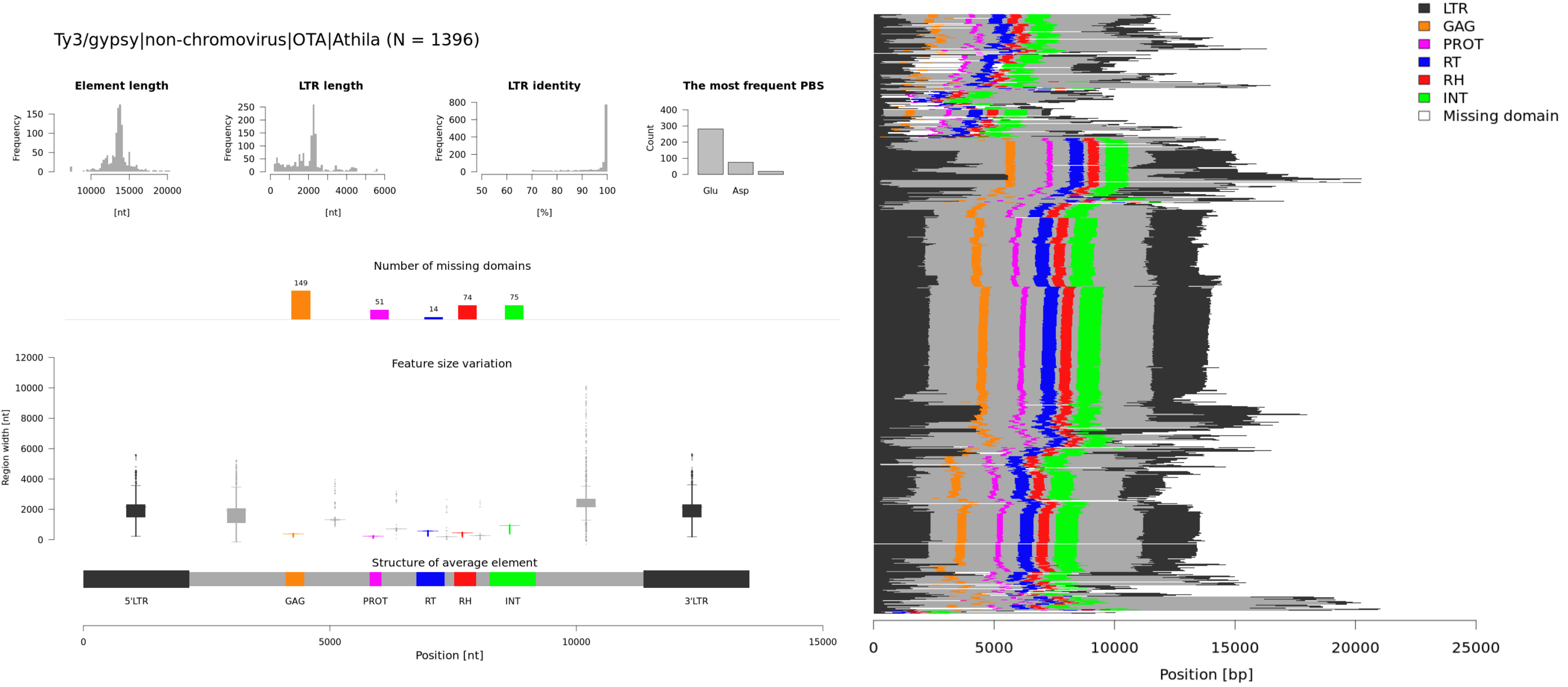

Composition and identity of CENTROMERIC LTR *Ty3/Gypsy ATHILA* elements found in the *R. canina* genome.

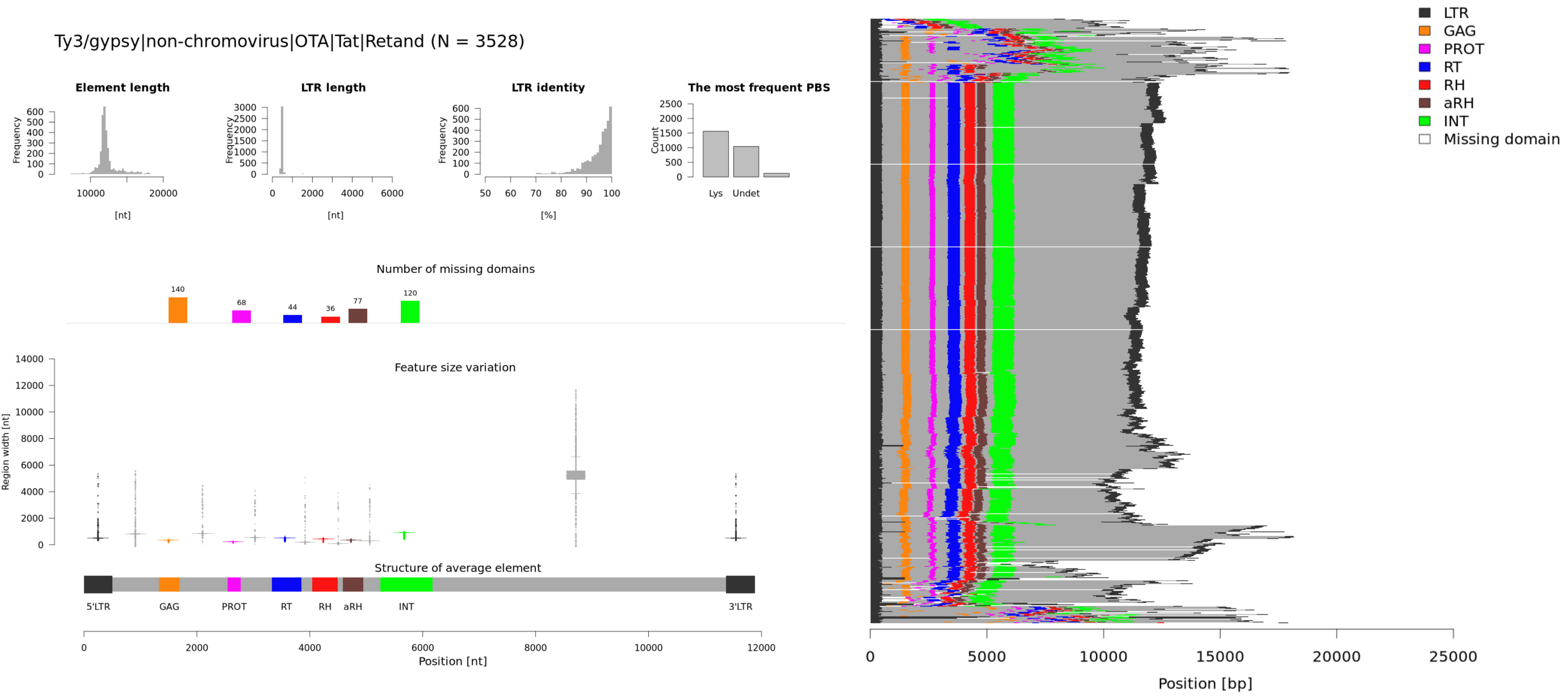

Composition and identity of LTR *Ty3/Gypsy RETAND* elements found in the *R. canina* genome.

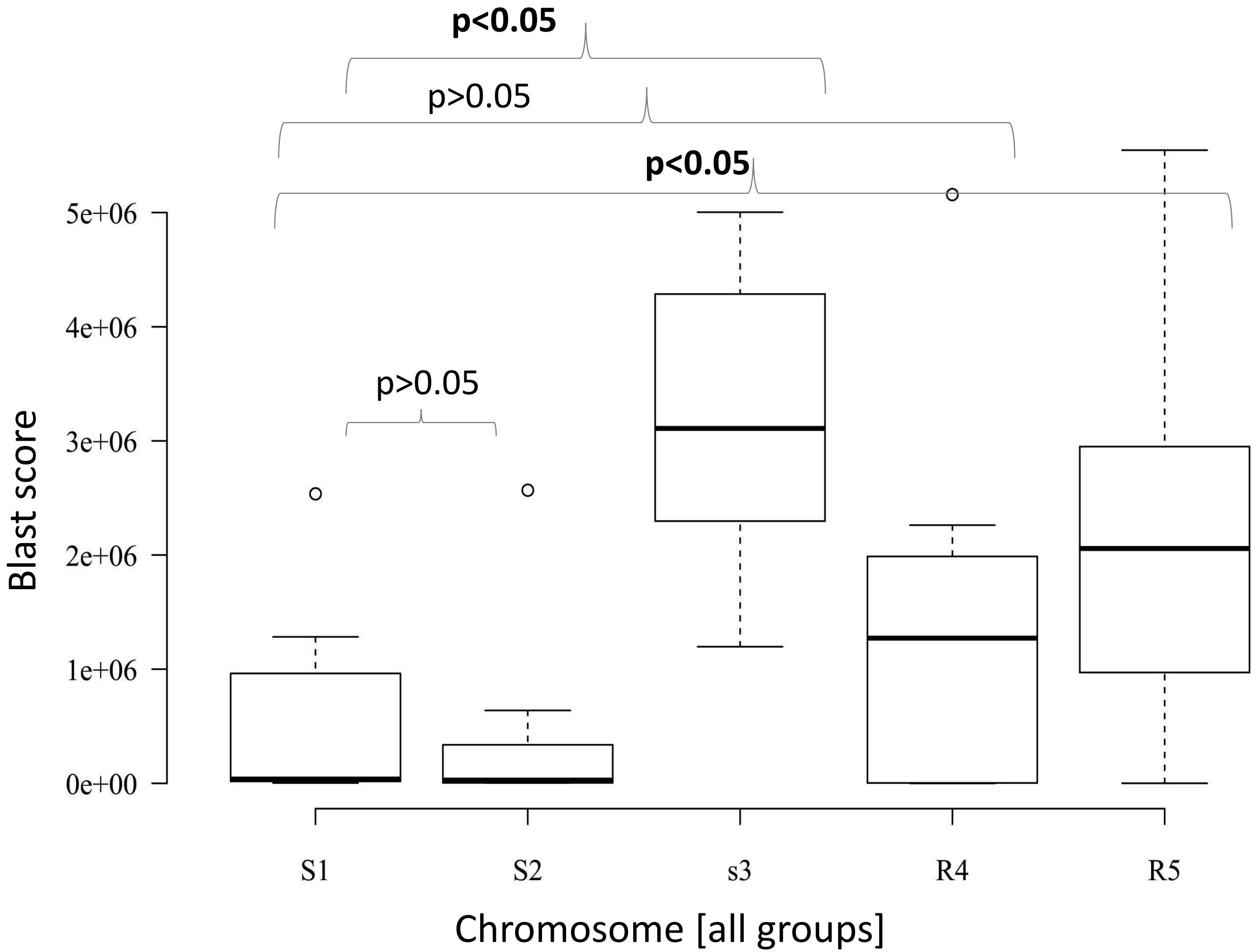

Figure SX. Comparison of the CANR4 satellite abundance between the *R.canina* chromosomes. (a) Distribution of CANR4 abundance in five subgenomes. The data were obtained from outputs of the BLAST analysis of all 35 chromosomes (seven in each subgenome) (BLAST parameters were e-value <0.001, gapscore_The box is drawn from the first and third quartiles, line in the middle represents the median. Statistics-Mann-Whitneyu-test (2 tailed).
