# Supplementary material for: Bimodal centromeres in pentaploid dogroses shed light on their unique meiosis
Source: Nature. 2025 Jun 18;643(8070):148–57. doi: 10.1038/s41586-025-09171-z (PMC12222009; doi:10.1038/s41586-025-09171-z)
Supplement: Supplementary file 3 — Supplementary Data 1–17. [file 41586_2025_9171_MOESM3_ESM.zip › Suppl_Dataset_8_rosAgr_DTOL_ModDotPlot.pdf]

Rosa agrestis DTOL

PRJEB79879

Centromeres

ModDotPlot

Supplementary Dataset 7. Structural analysis of whole chromosome and centromeres of bivalent- and univalent-forming chromosomes of *R. agrestis*. One to two prominent higher order arrays were visualised as negatively stained diagonal strips. The colour intensity histograms in top left plot the number of alignments versus pairwise sequence identity. Repeat profile densities are plotted below ModDotPlot showing the main classes of repeats identified. Window-size of 100 kbp and 10 kbp are shown for chromosome-wise and centromere plots, respectively. Please note the frequent association of *CANR4*-enriched centromeres for chromosomes of univalent-forming subgenomes *S2* and *R3*.

Rag1_S1

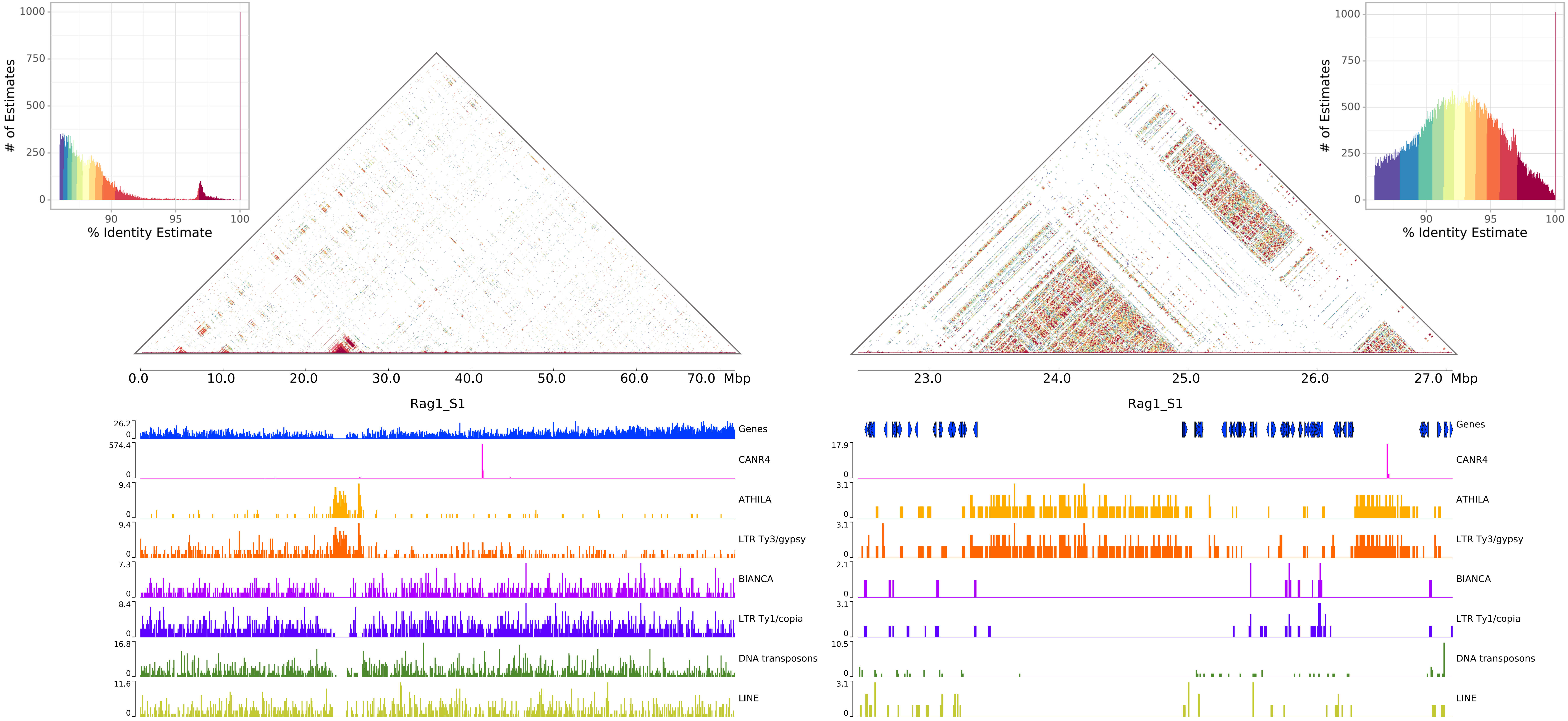

# of Estimates
1000
750
500
250
0
90
95
100
% Identity Estimate
0.0
10.0
20.0
30.0
40.0
50.0
60.0
70.0
Mbp
Rag1_S1
23.0
24.0
25.0
26.0
27.0
Mbp
Rag1_S1
Genes
CANR4
ATHILA
LTR Ty3/gypsy
BIANCA
LTR Ty1/copia
DNA transposons
LINE
26.2
574.4
9.4
9.4
7.3
8.4
16.8
11.6
17.9
3.1
3.1
2.1
3.1
10.5
3.1

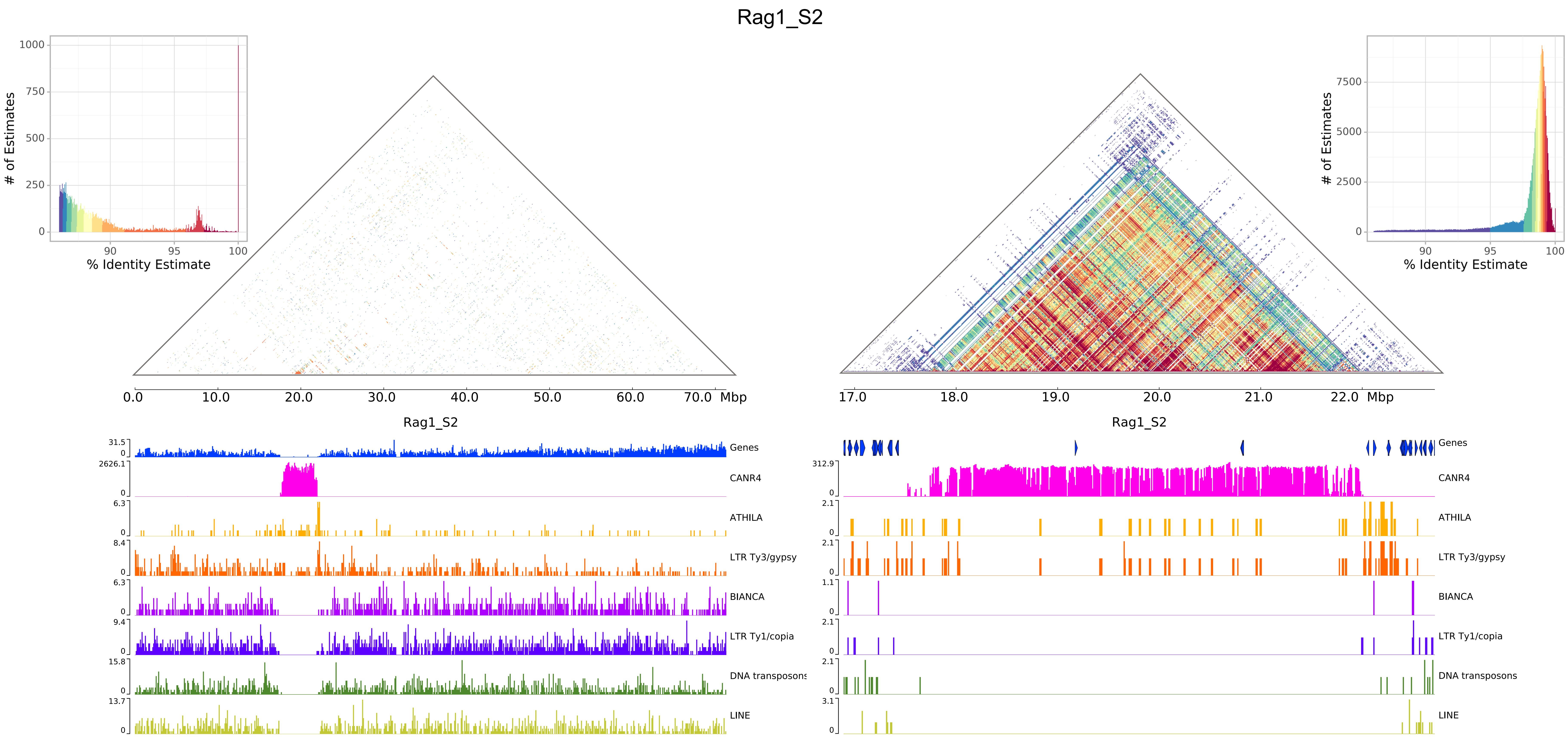

Rag1_S2
# of Estimates
1000
750
500
250
0
90
95
100
% Identity Estimate
7500
5000
2500
0.0
10.0
20.0
30.0
40.0
50.0
60.0
70.0
Mbp
Rag1_S2
17.0
18.0
19.0
20.0
21.0
22.0
Genes
CANR4
ATHILA
LTR Ty3/gypsy
BIANCA
LTR Ty1/copia
DNA transposons
LINE
31.5
2626.1
6.3
8.4
9.4
15.8
13.7
312.9
2.1
1.1
3.1

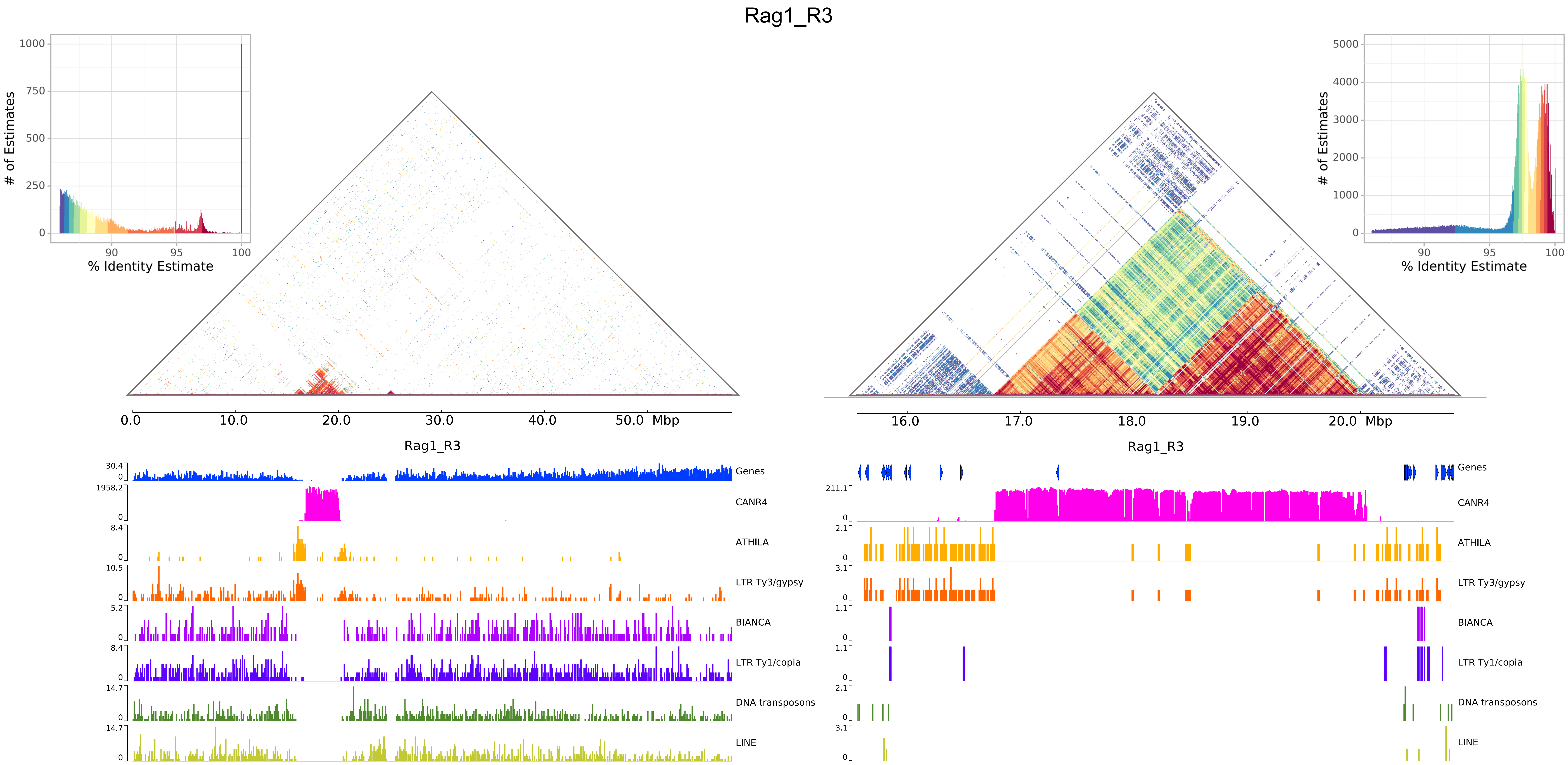

Rag1_R3
1000
750
500
250
0
# of Estimates
90
95
100
% Identity Estimate
5000
4000
3000
2000
1000
0
# of Estimates
90
95
100
% Identity Estimate
0.0
10.0
20.0
30.0
40.0
50.0
Mbp
Rag1_R3
16.0
17.0
18.0
19.0
20.0
Mbp
Rag1_R3
Genes
CANR4
ATHILA
LTR Ty3/gypsy
BIANCA
LTR Ty1/copia
DNA transposons
LINE
30.4
1958.2
8.4
10.5
5.2
8.4
14.7
14.7
211.1
2.1
3.1
1.1
1.1
2.1
3.1

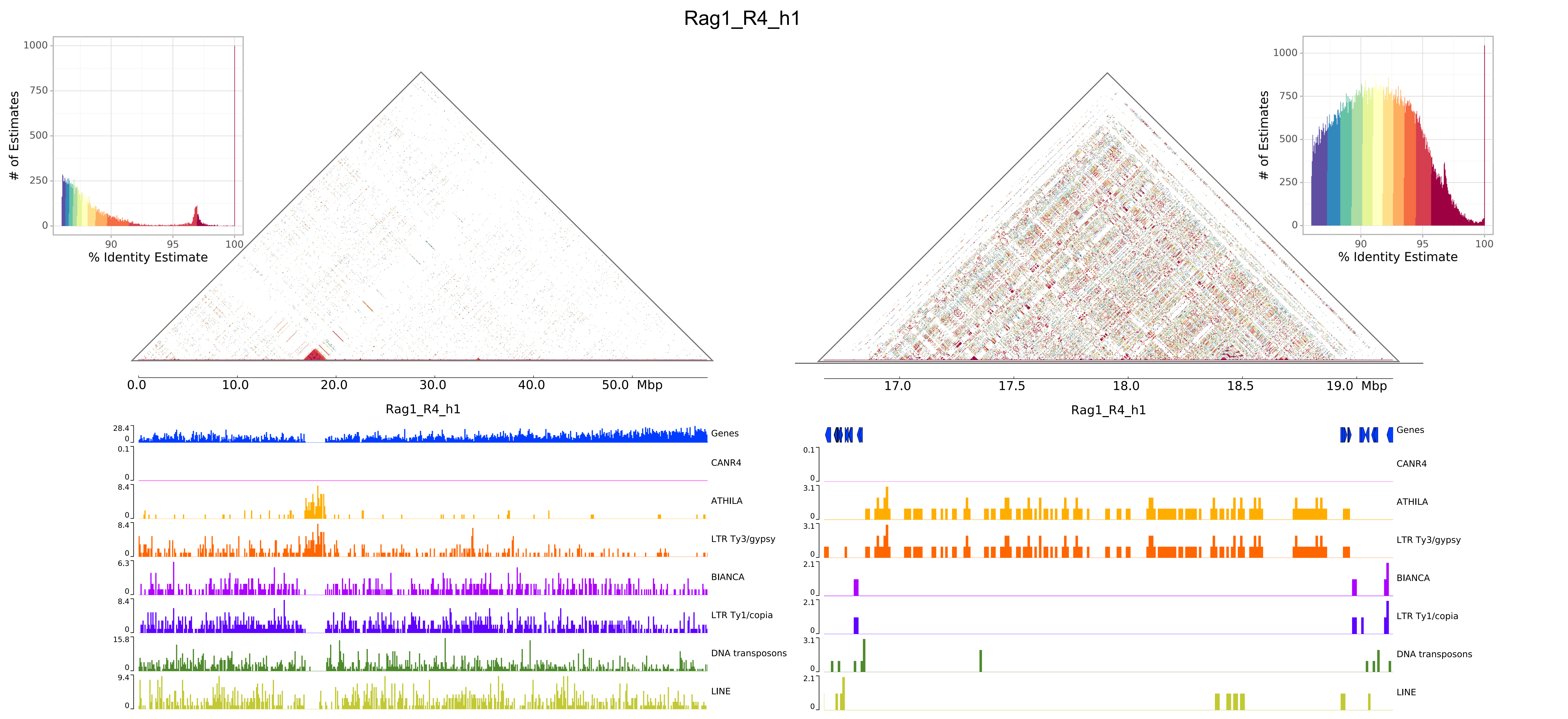

Rag1_R4_h1
# of Estimates
% Identity Estimate
1000
750
500
250
0
90
95
100
0.0
10.0
20.0
30.0
40.0
50.0 Mbp
17.0
17.5
18.0
18.5
19.0 Mbp
Rag1_R4_h1
Rag1_R4_h1
Genes
CANR4
ATHILA
LTR Ty3/gypsy
BIANCA
LTR Ty1/copia
DNA transposons
LINE
28.4
0.1
8.4
8.4
6.3
8.4
15.8
9.4
3.1
3.1
2.1
2.1
3.1
2.1

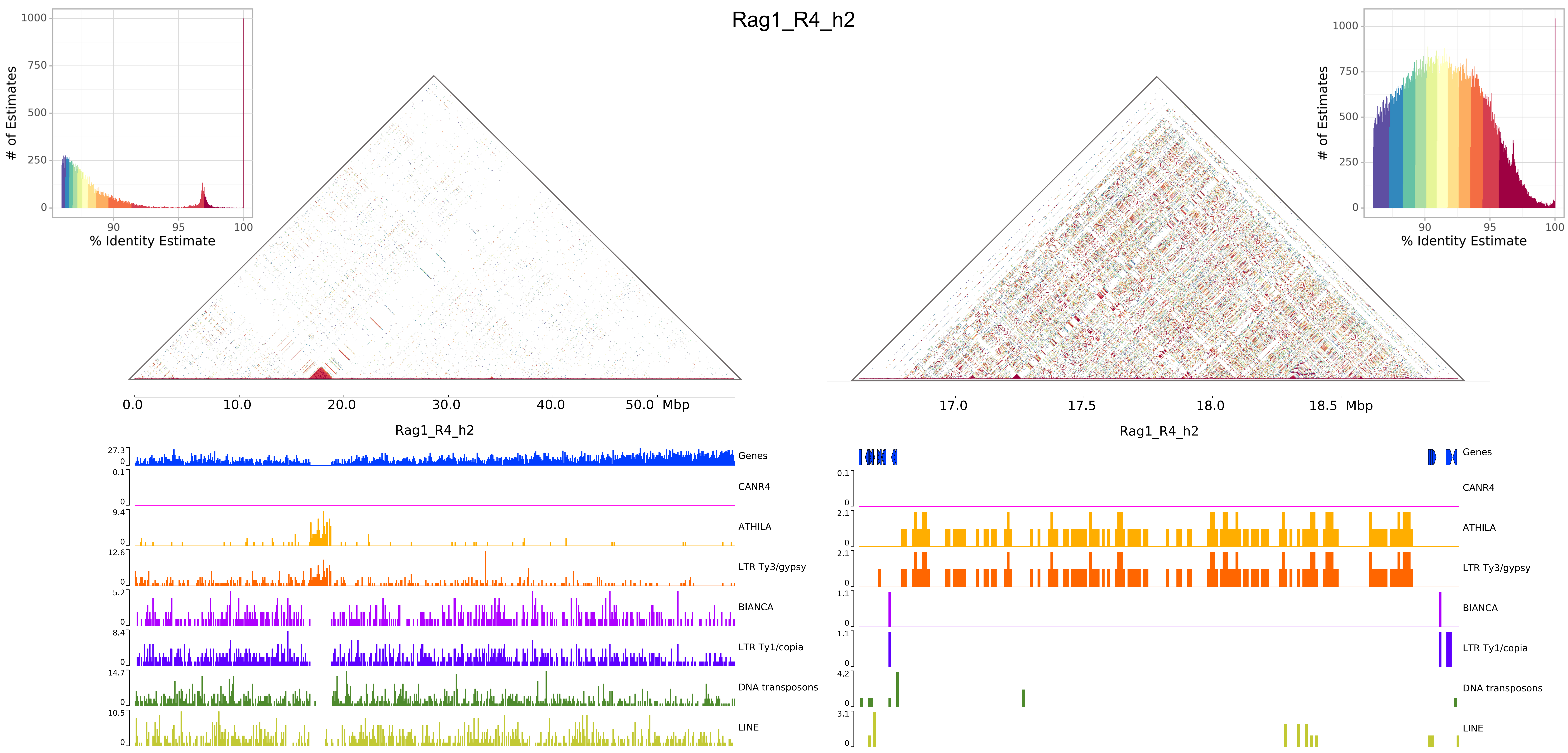

Rag1_R4_h2
# of Estimates
% Identity Estimate
0.0
10.0
20.0
30.0
40.0
50.0 Mbp
17.0
17.5
18.0
18.5 Mbp
Rag1_R4_h2
Genes
CANR4
ATHILA
LTR Ty3/gypsy
BIANCA
LTR Ty1/copia
DNA transposons
LINE

Rag2_S1

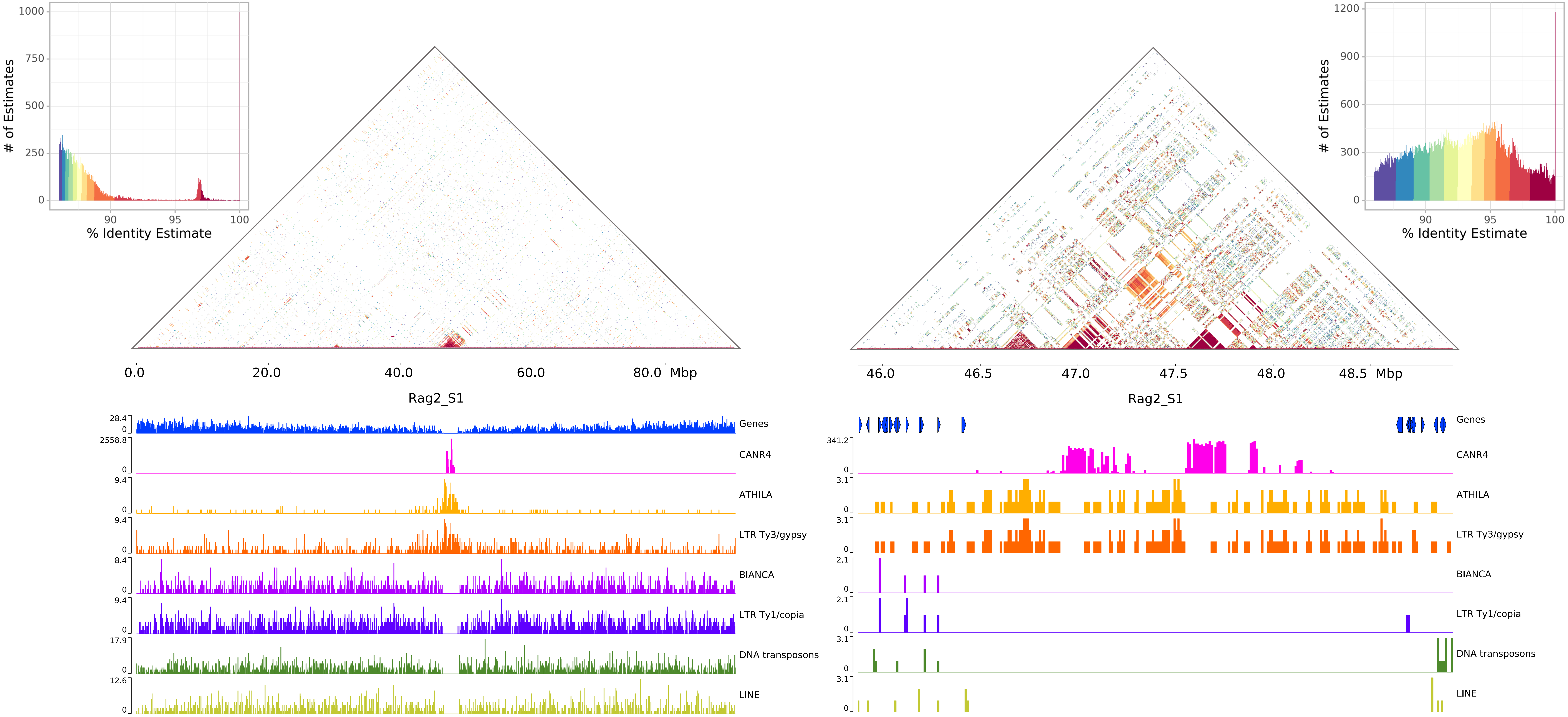

# of Estimates
% Identity Estimate
1000
750
500
250
0
90
95
100
1200
900
600
300
0.0
20.0
40.0
60.0
80.0 Mbp
46.0
46.5
47.0
47.5
48.0
48.5 Mbp
Rag2_S1
Genes
CANR4
ATHILA
LTR Ty3/gypsy
BIANCA
LTR Ty1/copia
DNA transposons
LINE
28.4
2558.8
9.4
8.4
17.9
12.6
341.2
3.1
2.1

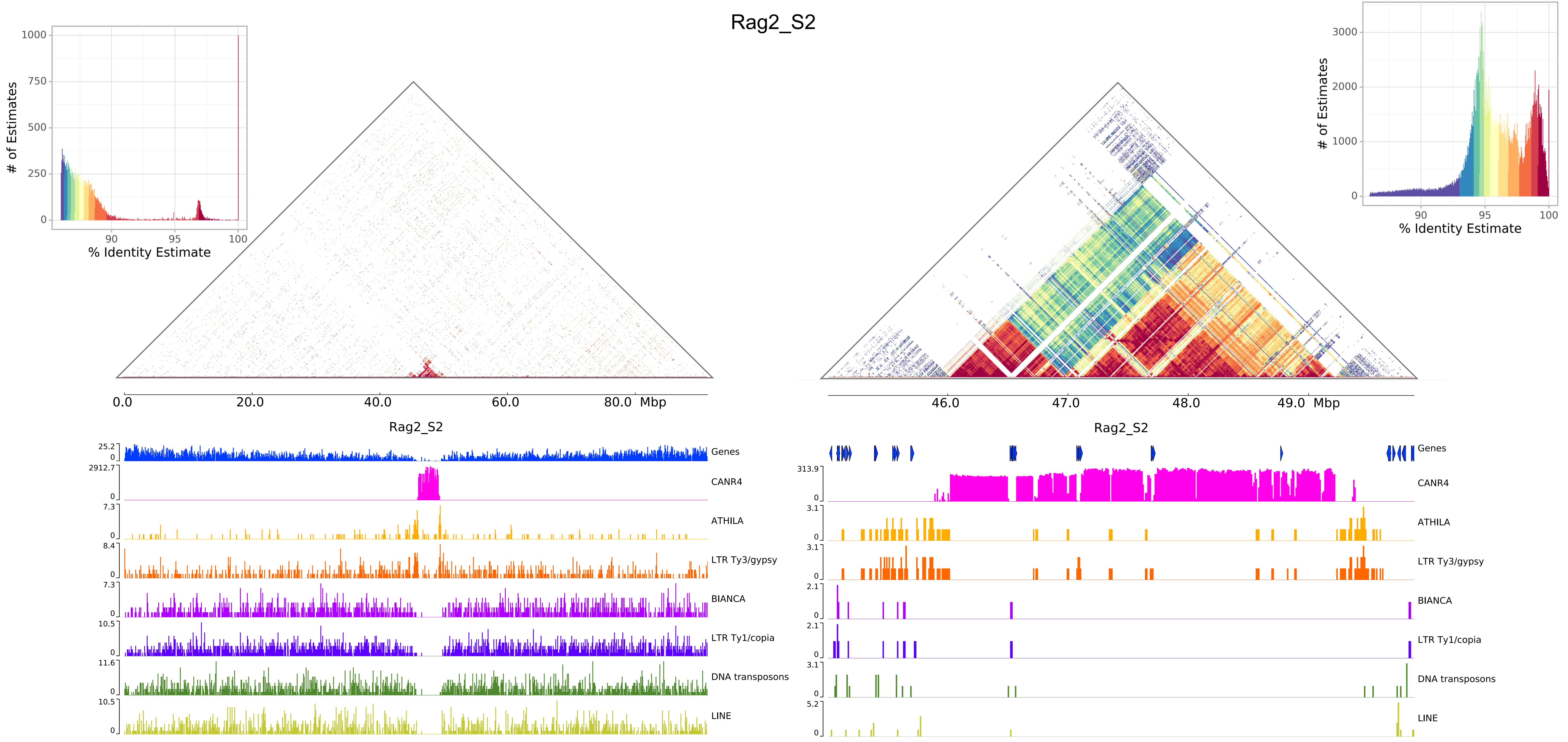

Rag2_S2
# of Estimates
% Identity Estimate
1000
750
500
250
0
90
95
100
3000
2000
1000
0.0
20.0
40.0
60.0
80.0 Mbp
46.0
47.0
48.0
49.0 Mbp
Rag2_S2
Genes
CANR4
ATHILA
LTR Ty3/gypsy
BIANCA
LTR Ty1/copia
DNA transposons
LINE
25.2
2912.7
7.3
8.4
10.5
11.6
313.9
3.1
2.1
5.2

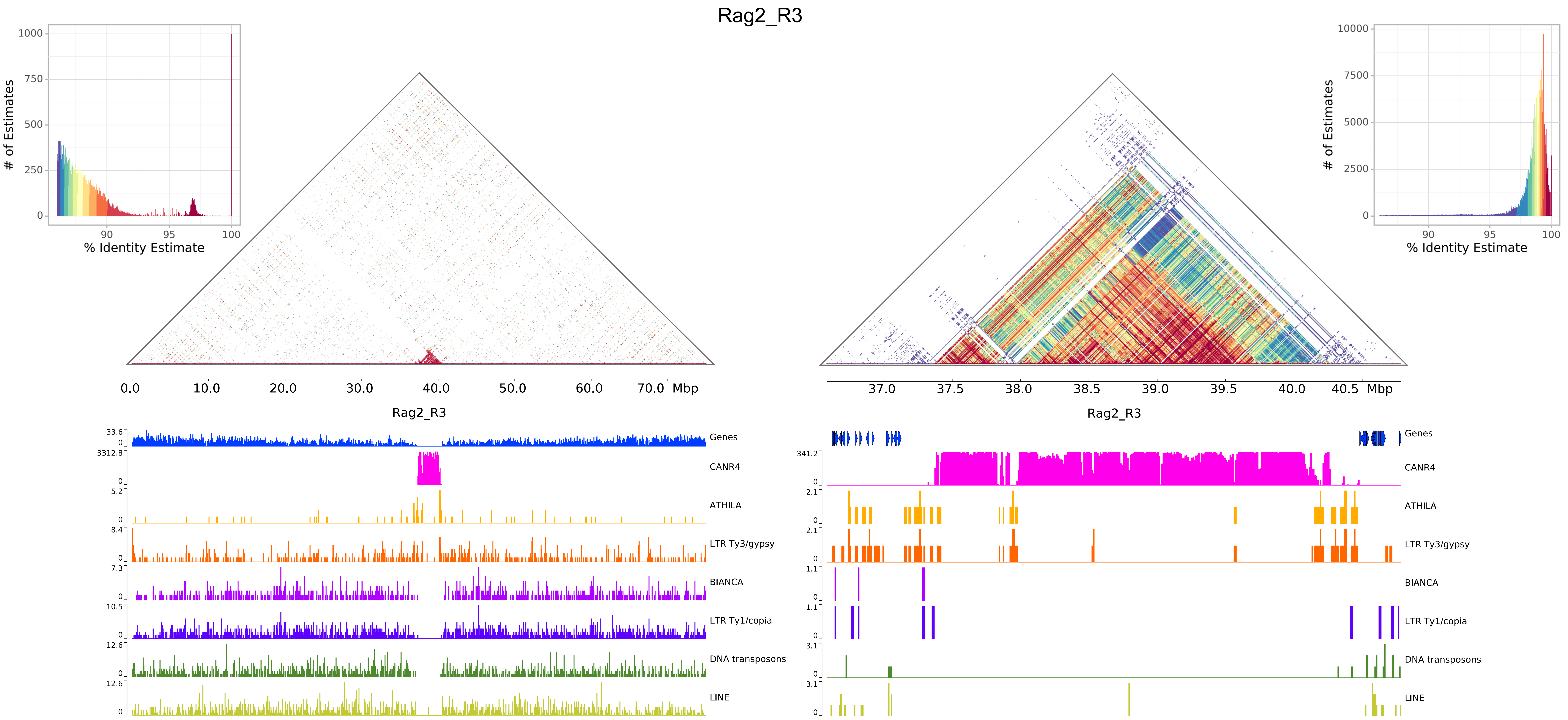

Rag2_R3
# of Estimates
% Identity Estimate
1000
750
500
250
0
90
95
100
10000
7500
5000
2500
0
0.0
10.0
20.0
30.0
40.0
50.0
60.0
70.0
Mbp
37.0
37.5
38.0
38.5
39.0
39.5
40.0
40.5
Genes
CANR4
ATHILA
LTR Ty3/gypsy
BIANCA
LTR Ty1/copia
DNA transposons
LINE
33.6
3312.8
5.2
8.4
7.3
10.5
12.6
341.2
2.1
1.1
3.1

Rag2_R4_h1

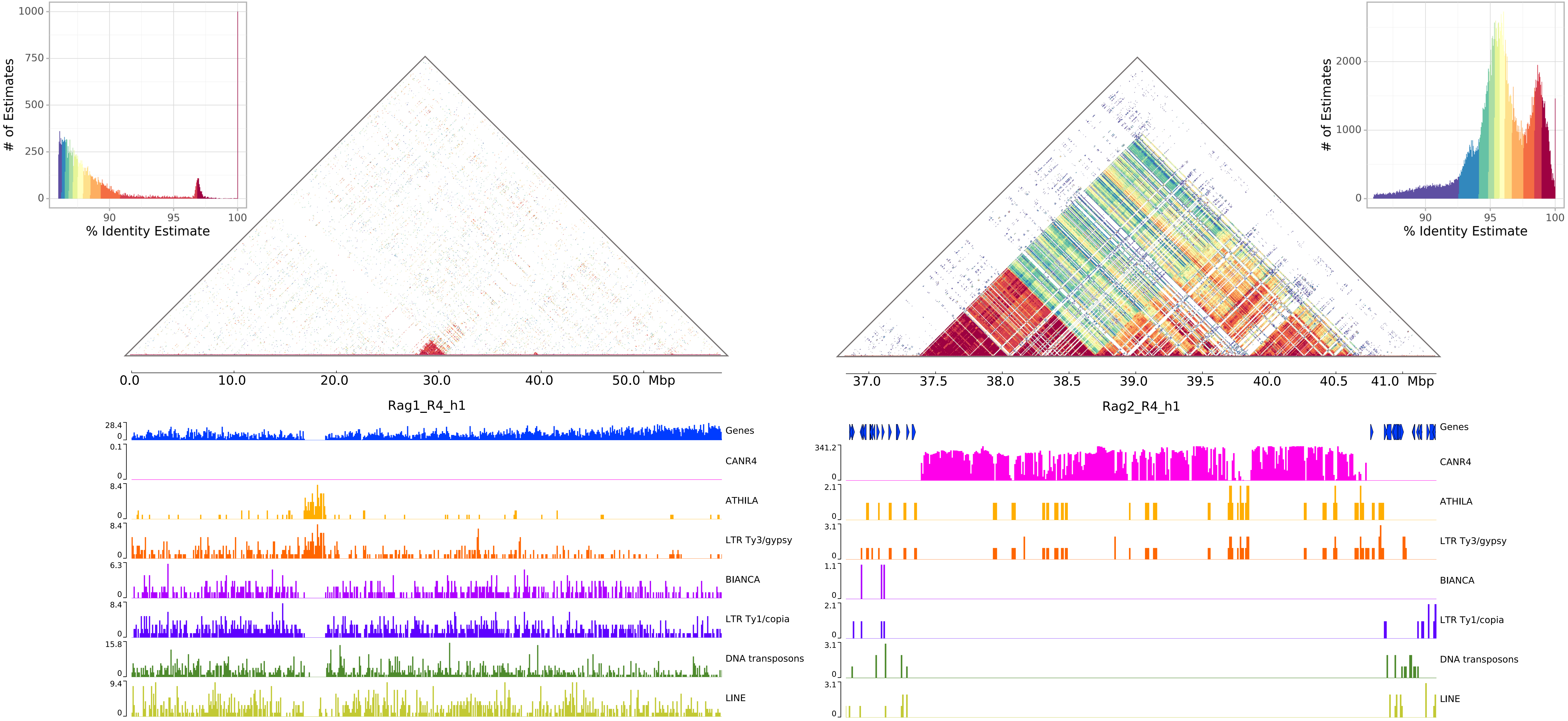

Rag2_R4_h1
# of Estimates
% Identity Estimate
Rag1_R4_h1
Rag2_R4_h1
Genes
CANR4
ATHILA
LTR Ty3/gypsy
BIANCA
LTR Ty1/copia
DNA transposons
LINE
Mbp

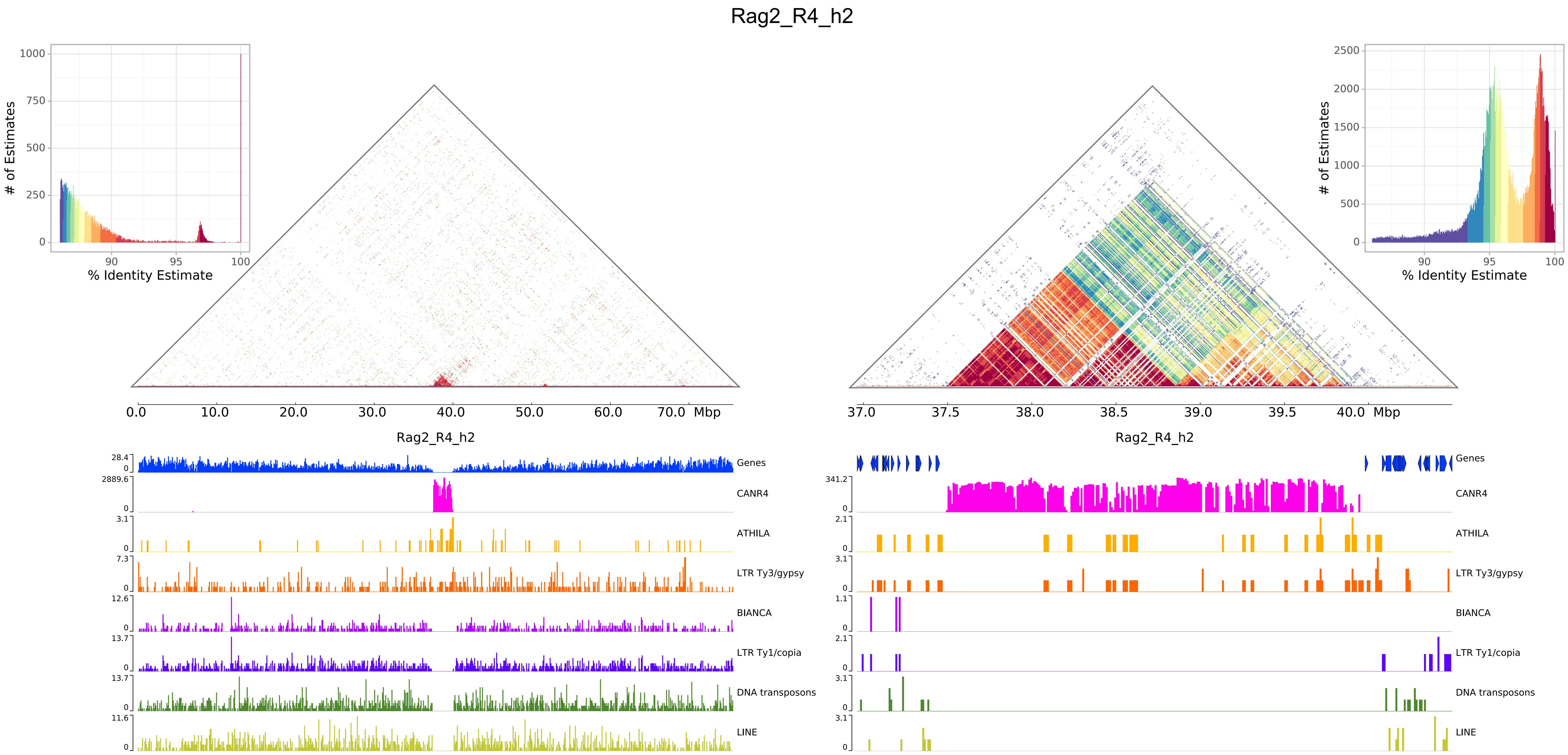

Rag2_R4_h2
# of Estimates
% Identity Estimate
0.0
10.0
20.0
30.0
40.0
50.0
60.0
70.0
Mbp
37.0
37.5
38.0
38.5
39.0
39.5
40.0
Rag2_R4_h2
Genes
CANR4
ATHILA
LTR Ty3/gypsy
BIANCA
LTR Ty1/copia
DNA transposons
LINE

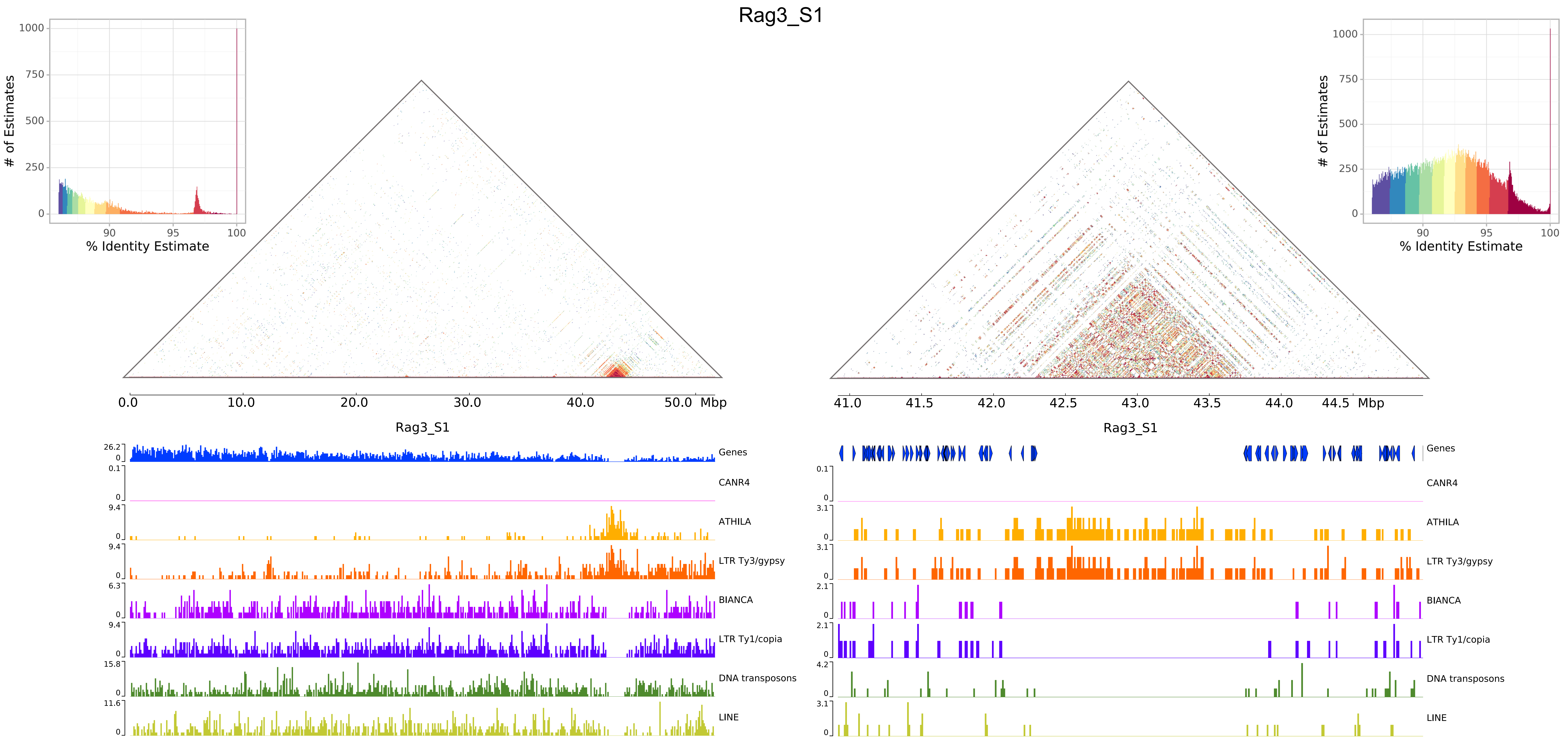

Rag3_S1
# of Estimates
1000
750
500
250
0
90
95
100
% Identity Estimate
0.0
10.0
20.0
30.0
40.0
50.0
Mbp
41.0
41.5
42.0
42.5
43.0
43.5
44.0
44.5
Rag3_S1
Genes
CANR4
ATHILA
LTR Ty3/gypsy
BIANCA
LTR Ty1/copia
DNA transposons
LINE
26.2
0.1
9.4
6.3
15.8
11.6
3.1
2.1
4.2

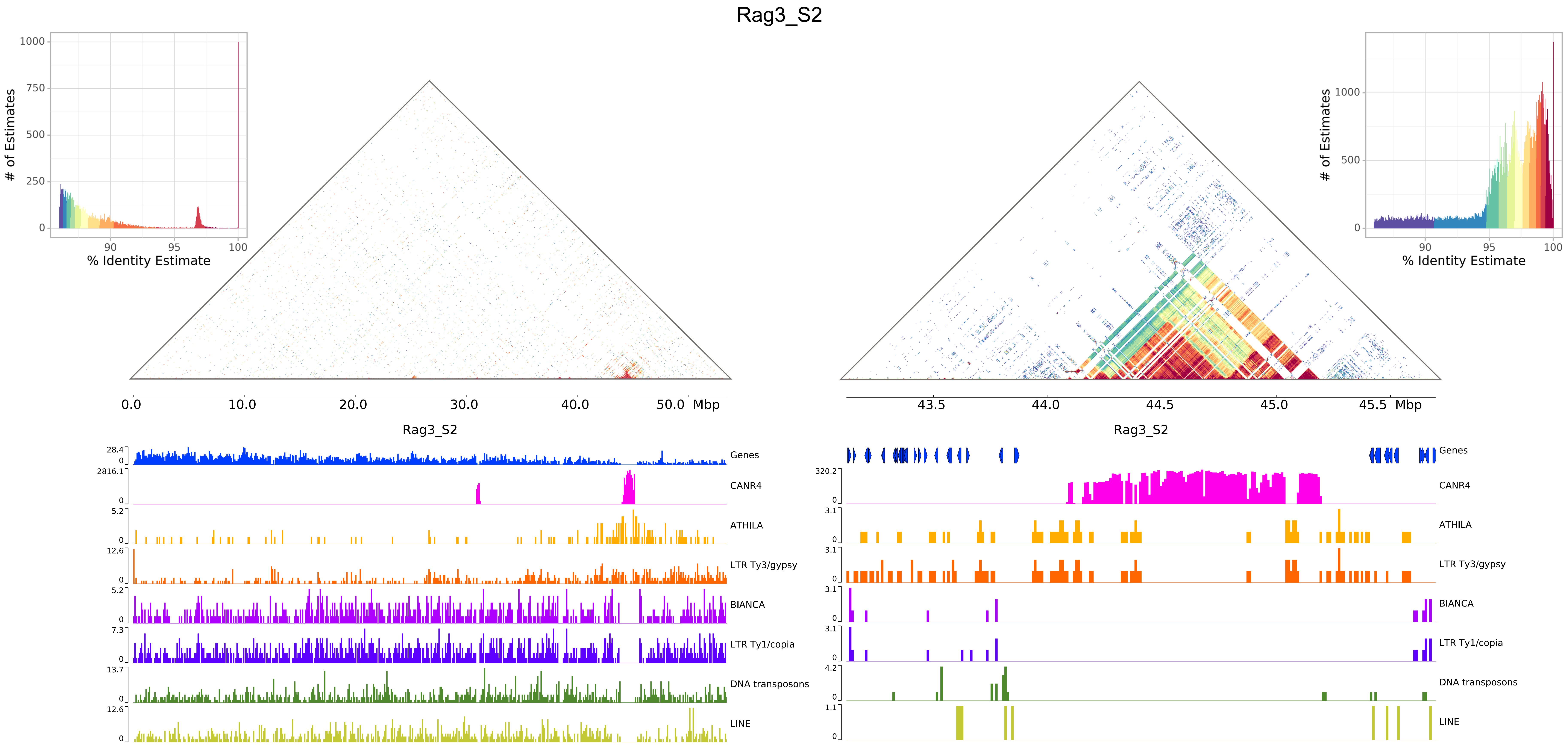

Rag3_S2
# of Estimates
% Identity Estimate
1000
750
500
250
0
90
95
100
0.0
10.0
20.0
30.0
40.0
50.0 Mbp
43.5
44.0
44.5
45.0
45.5 Mbp
Rag3_S2
Genes
CANR4
ATHILA
LTR Ty3/gypsy
BIANCA
LTR Ty1/copia
DNA transposons
LINE
28.4
2816.1
5.2
12.6
5.2
7.3
13.7
12.6
320.2
3.1
3.1
3.1
3.1
4.2
1.1

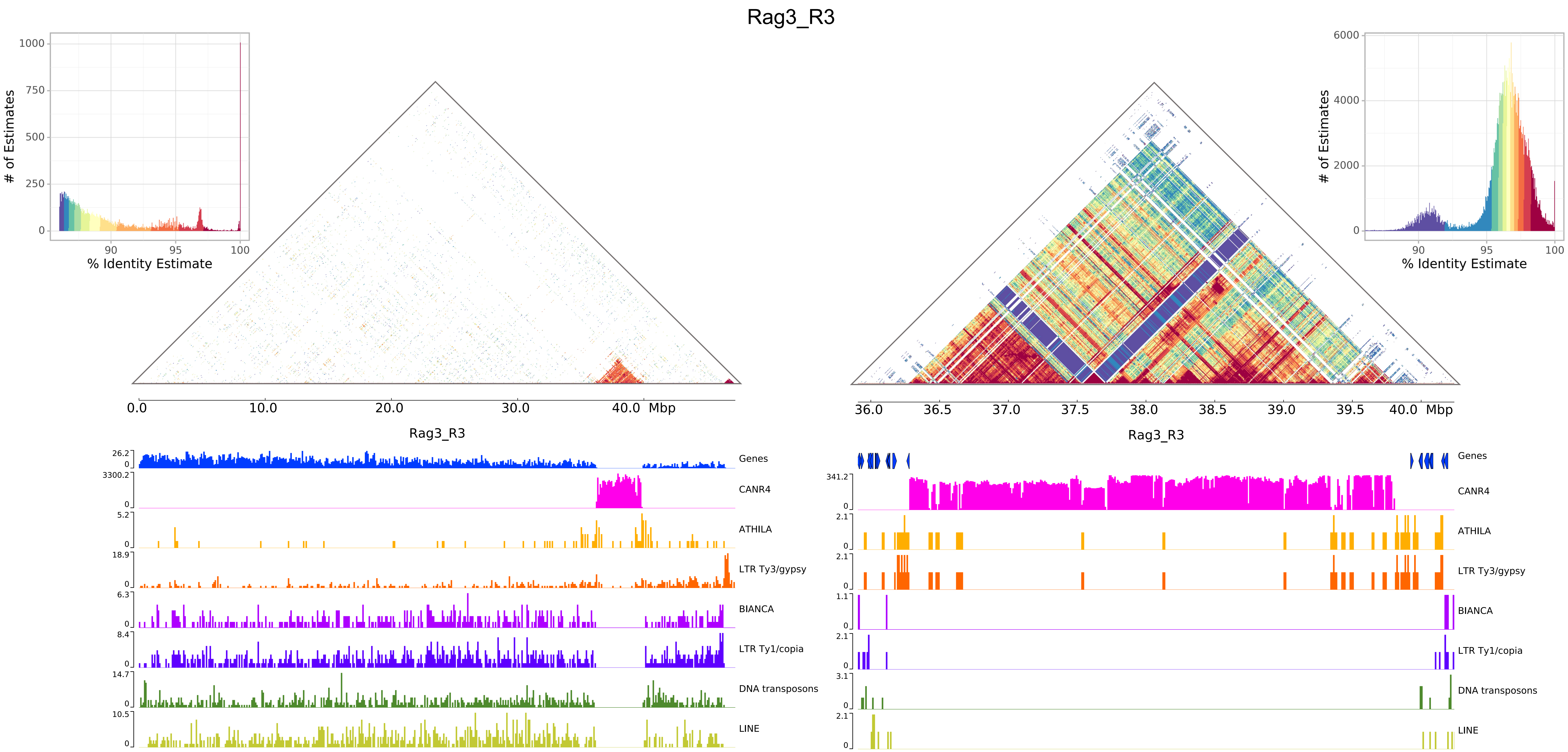

Rag3_R3
# of Estimates
% Identity Estimate
0.0
10.0
20.0
30.0
40.0 Mbp
Rag3_R3
36.0
36.5
37.0
37.5
38.0
38.5
39.0
39.5
40.0 Mbp
Rag3_R3
Genes
CANR4
ATHILA
LTR Ty3/gypsy
BIANCA
LTR Ty1/copia
DNA transposons
LINE

Rag3_R4_h1

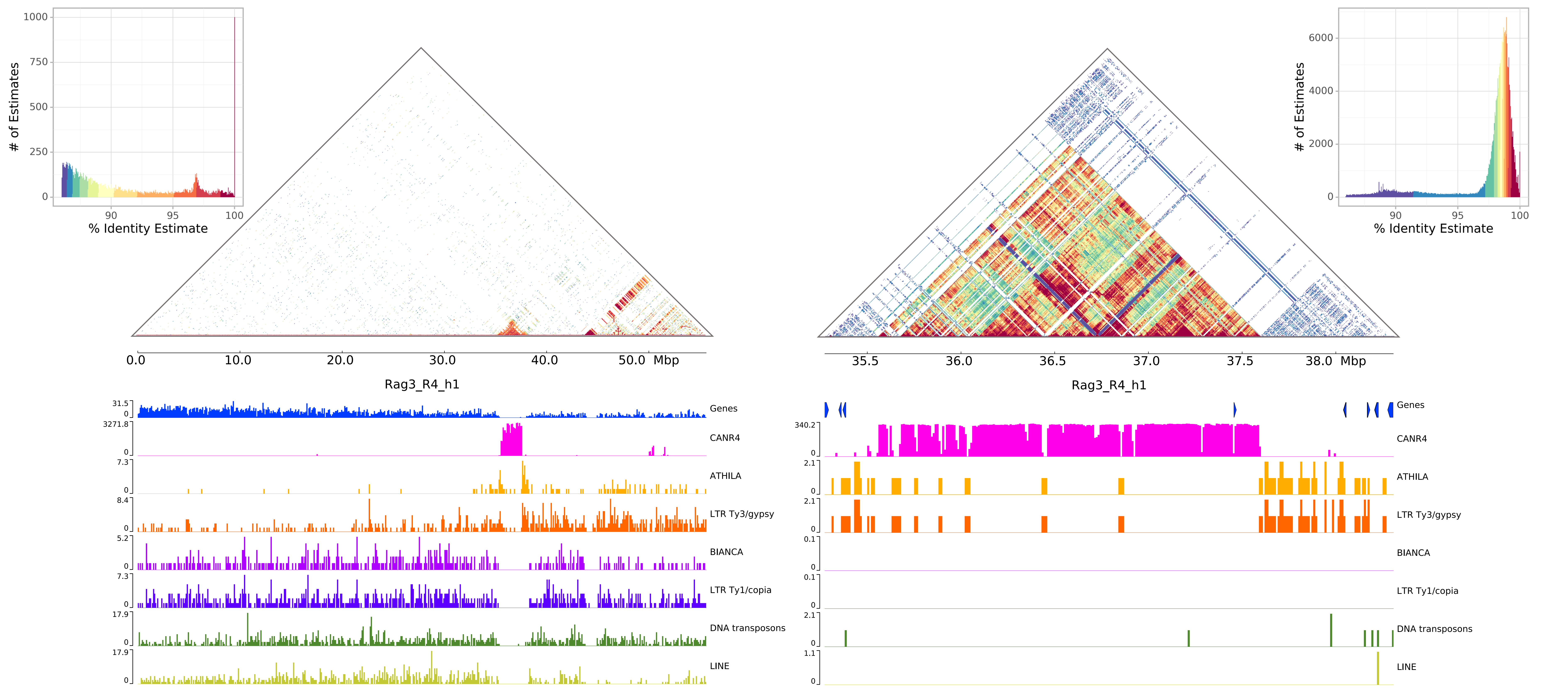

# of Estimates
% Identity Estimate
1000
750
500
250
0
90
95
100
6000
4000
2000
0.0
10.0
20.0
30.0
40.0
50.0 Mbp
Rag3_R4_h1
35.5
36.0
36.5
37.0
37.5
38.0 Mbp
Rag3_R4_h1
31.5
3271.8
7.3
8.4
5.2
17.9
340.2
2.1
0.1
1.1
Genes
CANR4
ATHILA
LTR Ty3/gypsy
BIANCA
LTR Ty1/copia
DNA transposons
LINE

Rag3_R4_h2

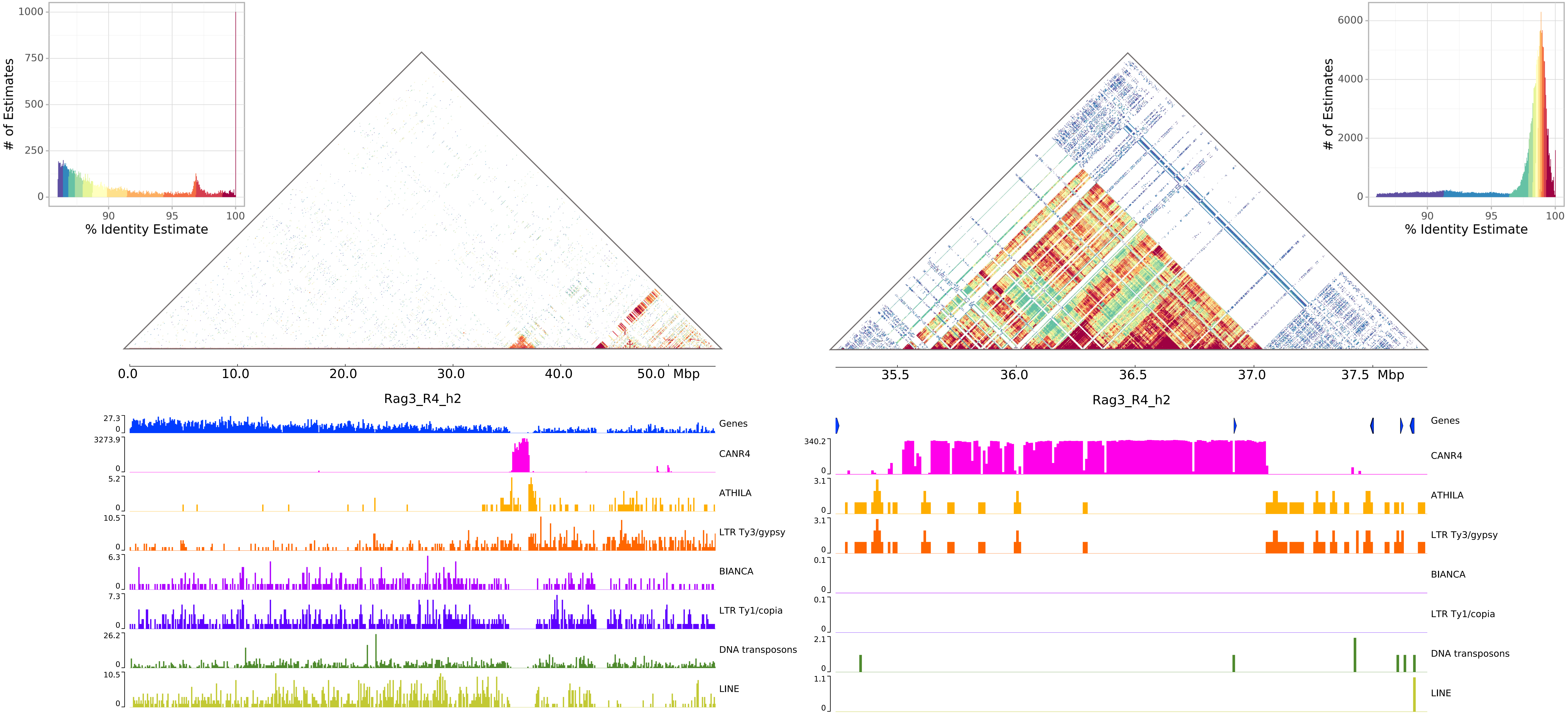

Rag3_R4_h2
# of Estimates
% Identity Estimate
1000
750
500
250
0
90
95
100
6000
4000
2000
0.0
10.0
20.0
30.0
40.0
50.0 Mbp
35.5
36.0
36.5
37.0
37.5 Mbp
Rag3_R4_h2
Genes
CANR4
ATHILA
LTR Ty3/gypsy
BIANCA
LTR Ty1/copia
DNA transposons
LINE
27.3
3273.9
5.2
10.5
6.3
7.3
26.2
10.5
340.2
3.1
3.1
0.1
0.1
2.1
1.1

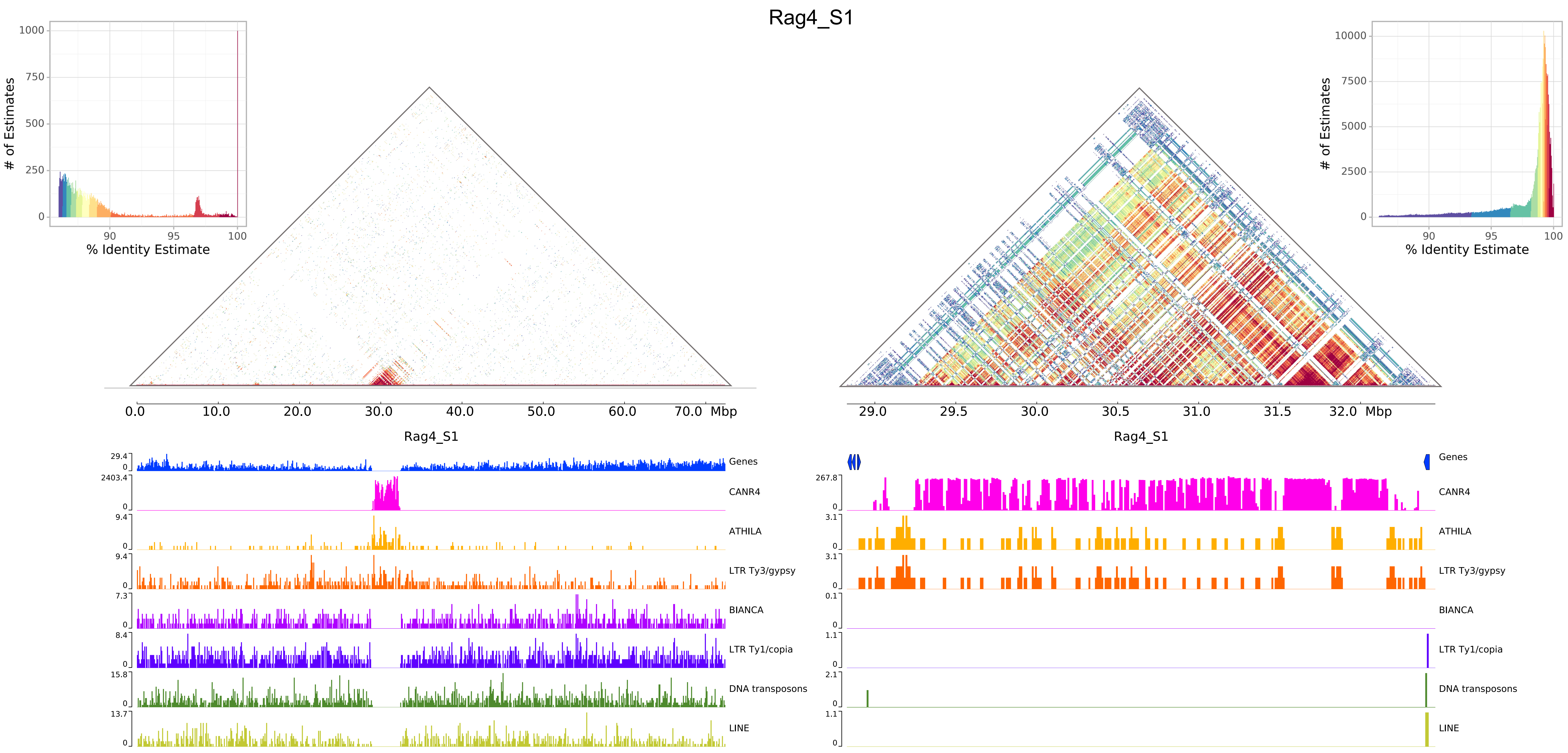

Rag4_S1
# of Estimates
1000
750
500
250
0
90
95
100
% Identity Estimate
10000
7500
5000
2500
0.0
10.0
20.0
30.0
40.0
50.0
60.0
70.0
Mbp
Rag4_S1
29.0
29.5
30.0
30.5
31.0
31.5
32.0
Genes
CANR4
ATHILA
LTR Ty3/gypsy
BIANCA
LTR Ty1/copia
DNA transposons
LINE
29.4
2403.4
9.4
7.3
8.4
15.8
13.7
267.8
3.1
0.1
1.1
2.1

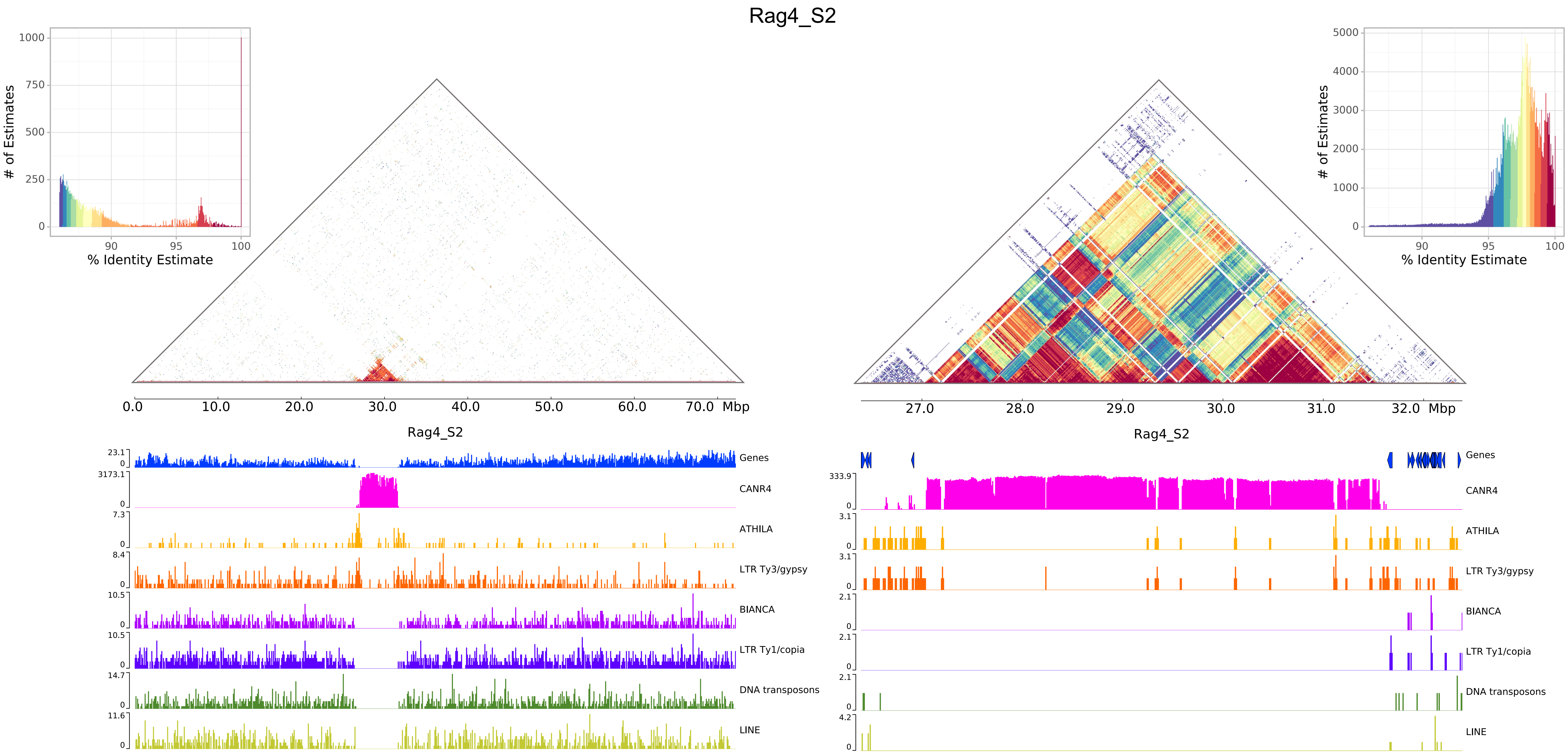

Rag4_S2
# of Estimates
% Identity Estimate
0.0
10.0
20.0
30.0
40.0
50.0
60.0
70.0 Mbp
Rag4_S2
27.0
28.0
29.0
30.0
31.0
32.0 Mbp
Rag4_S2
Genes
CANR4
ATHILA
LTR Ty3/gypsy
BIANCA
LTR Ty1/copia
DNA transposons
LINE

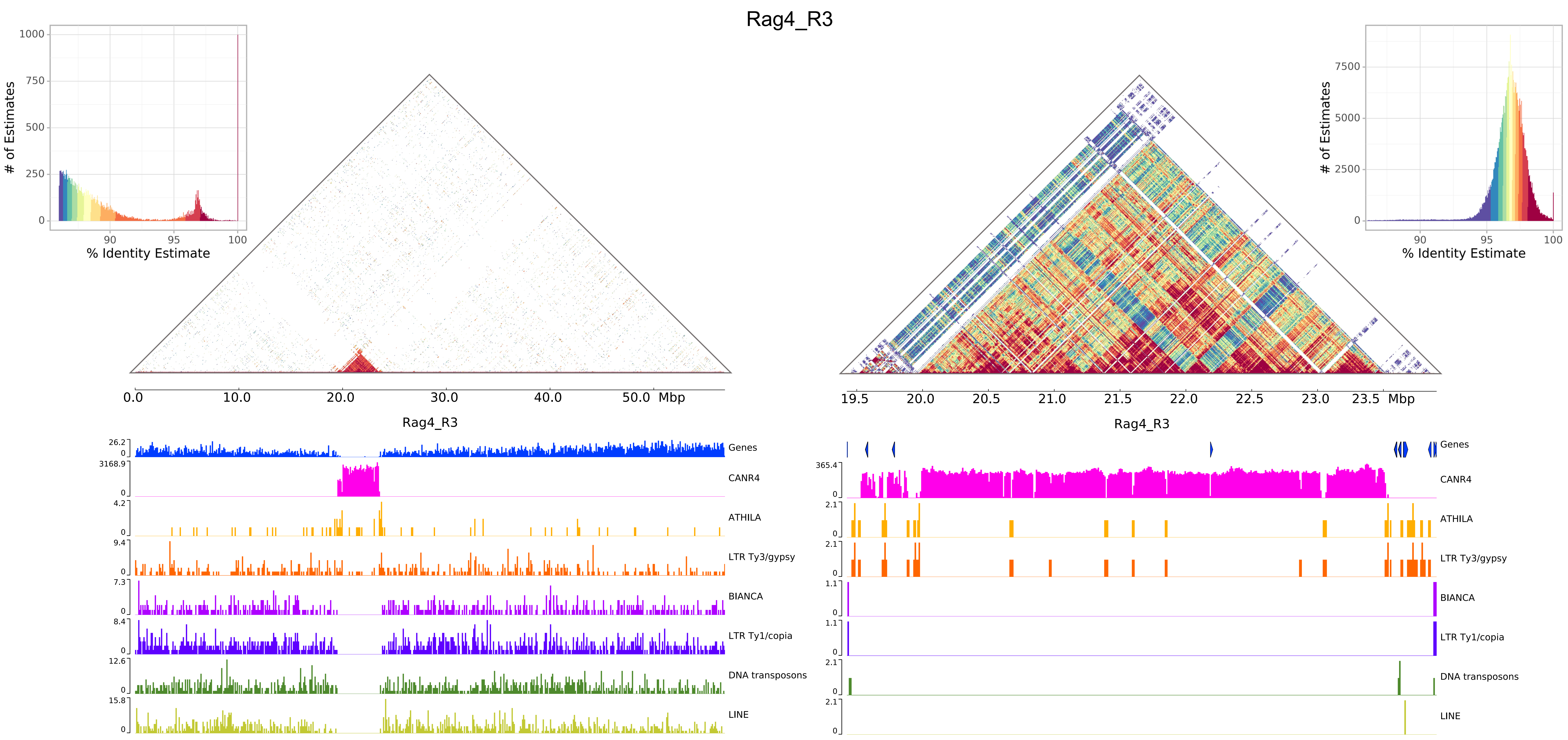
Rag4_R3
1000
750
500
250
0
# of Estimates
90
95
100
% Identity Estimate
7500
5000
2500
0
# of Estimates
90
95
100
% Identity Estimate
0.0
10.0
20.0
30.0
40.0
50.0 Mbp
Rag4_R3
19.5
20.0
20.5
21.0
21.5
22.0
22.5
23.0
23.5 Mbp
Rag4_R3
26.2
3168.9
4.2
9.4
7.3
8.4
12.6
15.8
Genes
CANR4
ATHILA
LTR Ty3/gypsy
BIANCA
LTR Ty1/copia
DNA transposons
LINE
365.4
2.1
2.1
1.1
1.1
2.1
2.1
Genes
CANR4
ATHILA
LTR Ty3/gypsy
BIANCA
LTR Ty1/copia
DNA transposons
LINE

Rag4_R4_h1

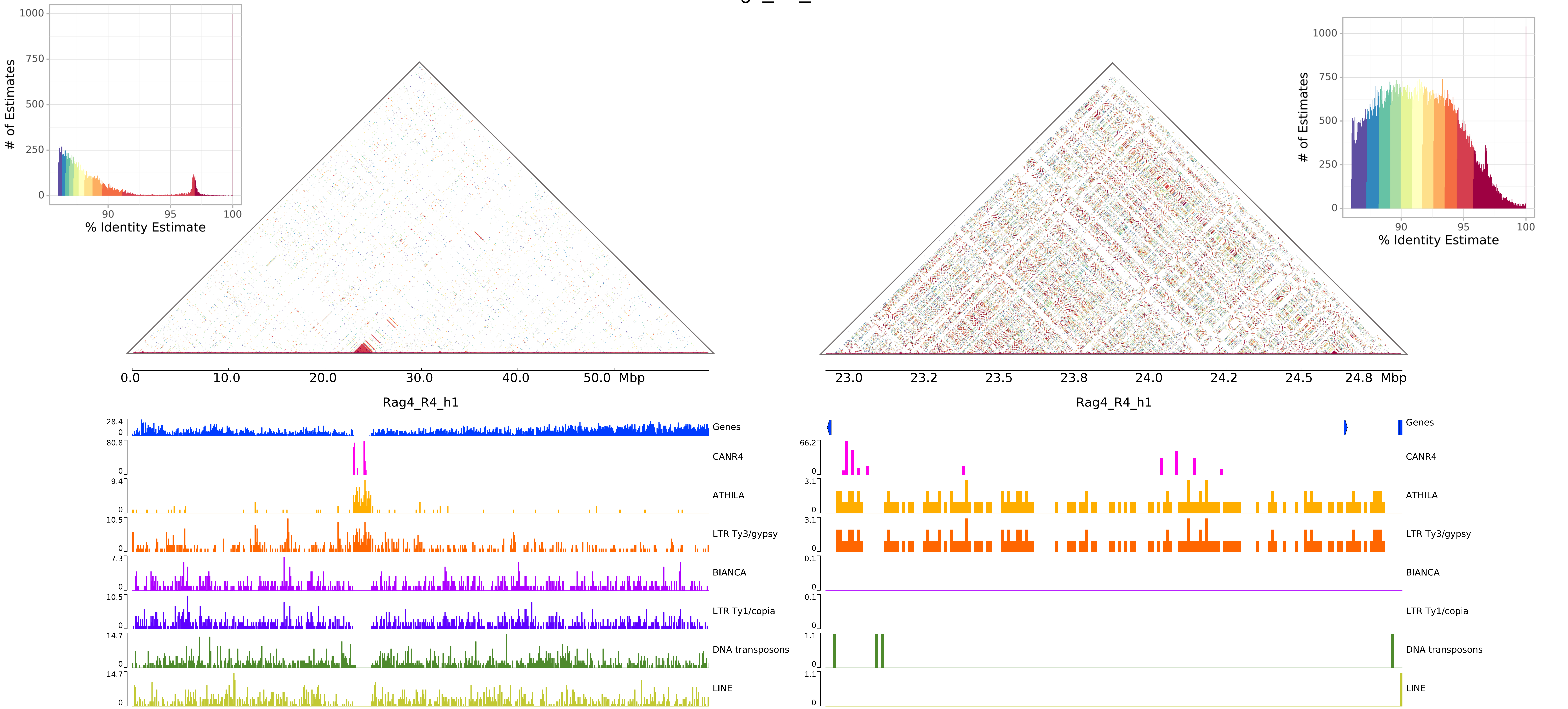

Rag4_R4_h1
# of Estimates
% Identity Estimate
0.0
10.0
20.0
30.0
40.0
50.0
Mbp
23.0
23.2
23.5
23.8
24.0
24.2
24.5
24.8
Genes
CANR4
ATHILA
LTR Ty3/gypsy
BIANCA
LTR Ty1/copia
DNA transposons
LINE

Rag4_R4_h2

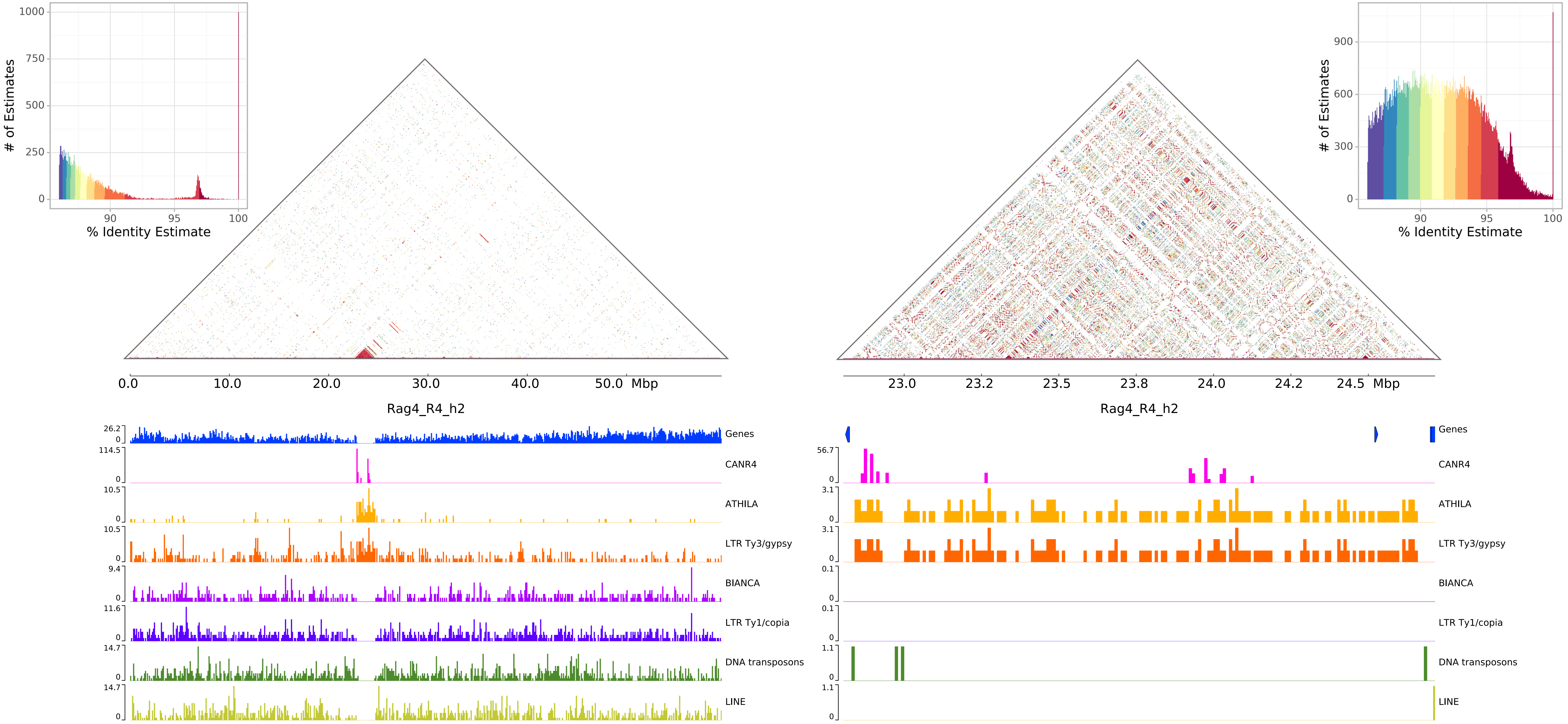

# of Estimates
1000
750
500
250
0
90
95
100
% Identity Estimate
900
600
300
0
90
95
100
% Identity Estimate
0.0
10.0
20.0
30.0
40.0
50.0 Mbp
Rag4_R4_h2
23.0
23.2
23.5
23.8
24.0
24.2
24.5 Mbp
Rag4_R4_h2
Genes
CANR4
ATHILA
LTR Ty3/gypsy
BIANCA
LTR Ty1/copia
DNA transposons
LINE
26.2
114.5
10.5
10.5
9.4
11.6
14.7
14.7
56.7
3.1
3.1
0.1
0.1
1.1
1.1

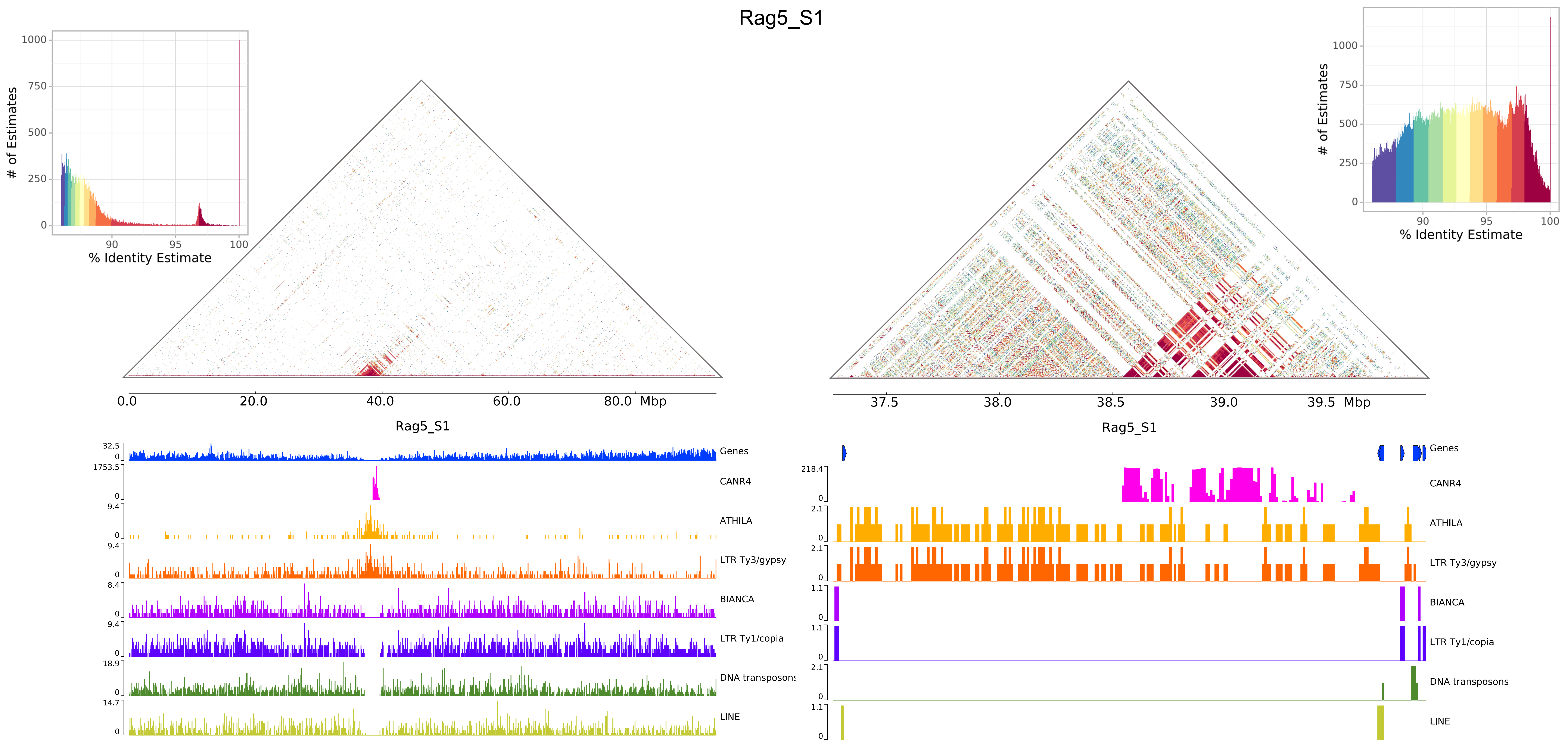

Rag5_S1
1000
750
500
250
0
# of Estimates
90
95
100
% Identity Estimate
0.0
20.0
40.0
60.0
80.0 Mbp
Rag5_S1
37.5
38.0
38.5
39.0
39.5 Mbp
Rag5_S1
Genes
CANR4
ATHILA
LTR Ty3/gypsy
BIANCA
LTR Ty1/copia
DNA transposons
LINE
32.5
1753.5
9.4
8.4
18.9
14.7
218.4
2.1
1.1

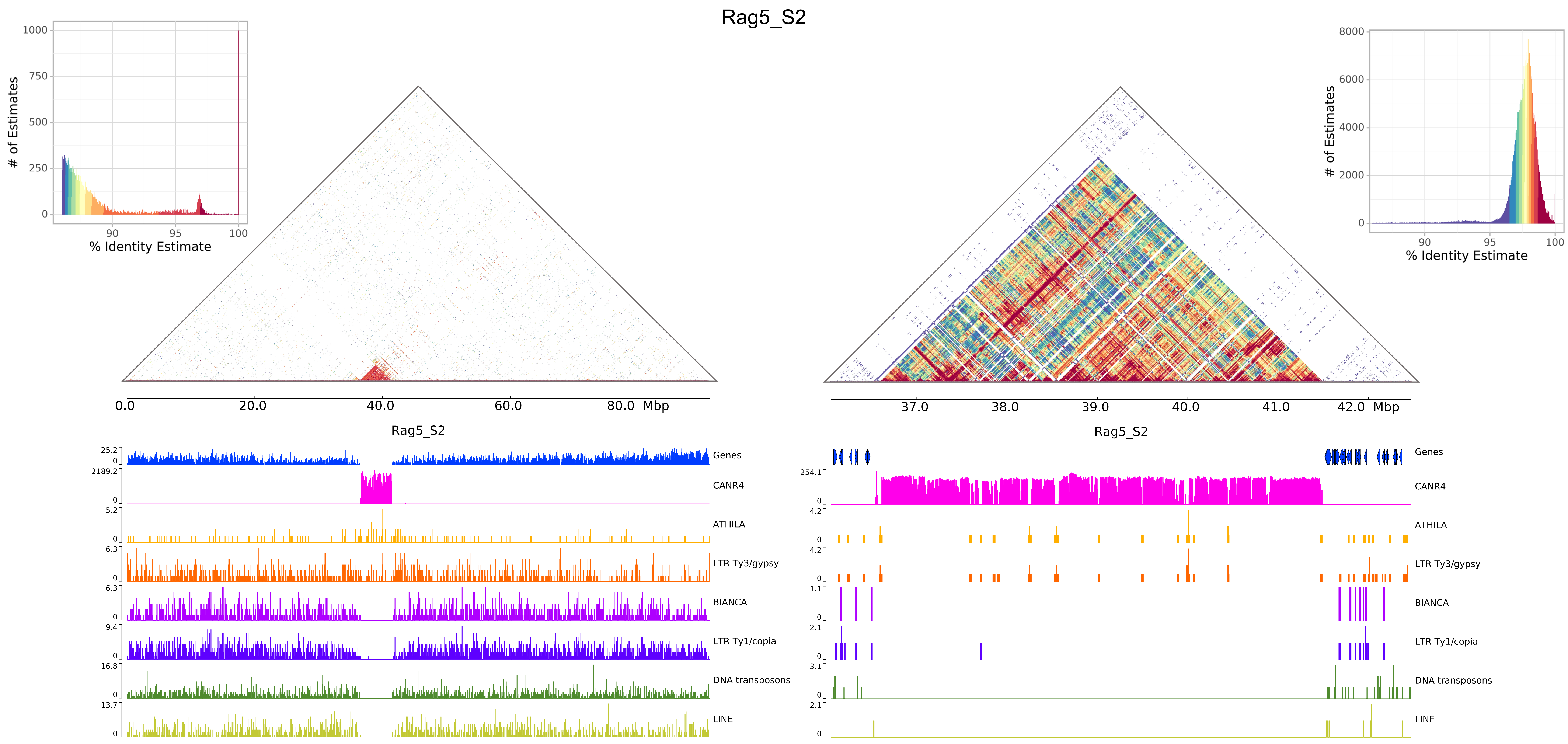

Rag5_S2
# of Estimates
% Identity Estimate
1000
750
500
250
0
90
95
100
8000
6000
4000
2000
0.0
20.0
40.0
60.0
80.0 Mbp
37.0
38.0
39.0
40.0
41.0
42.0 Mbp
Genes
CANR4
ATHILA
LTR Ty3/gypsy
BIANCA
LTR Ty1/copia
DNA transposons
LINE
25.2
2189.2
5.2
6.3
9.4
16.8
13.7
254.1
4.2
1.1
2.1
3.1

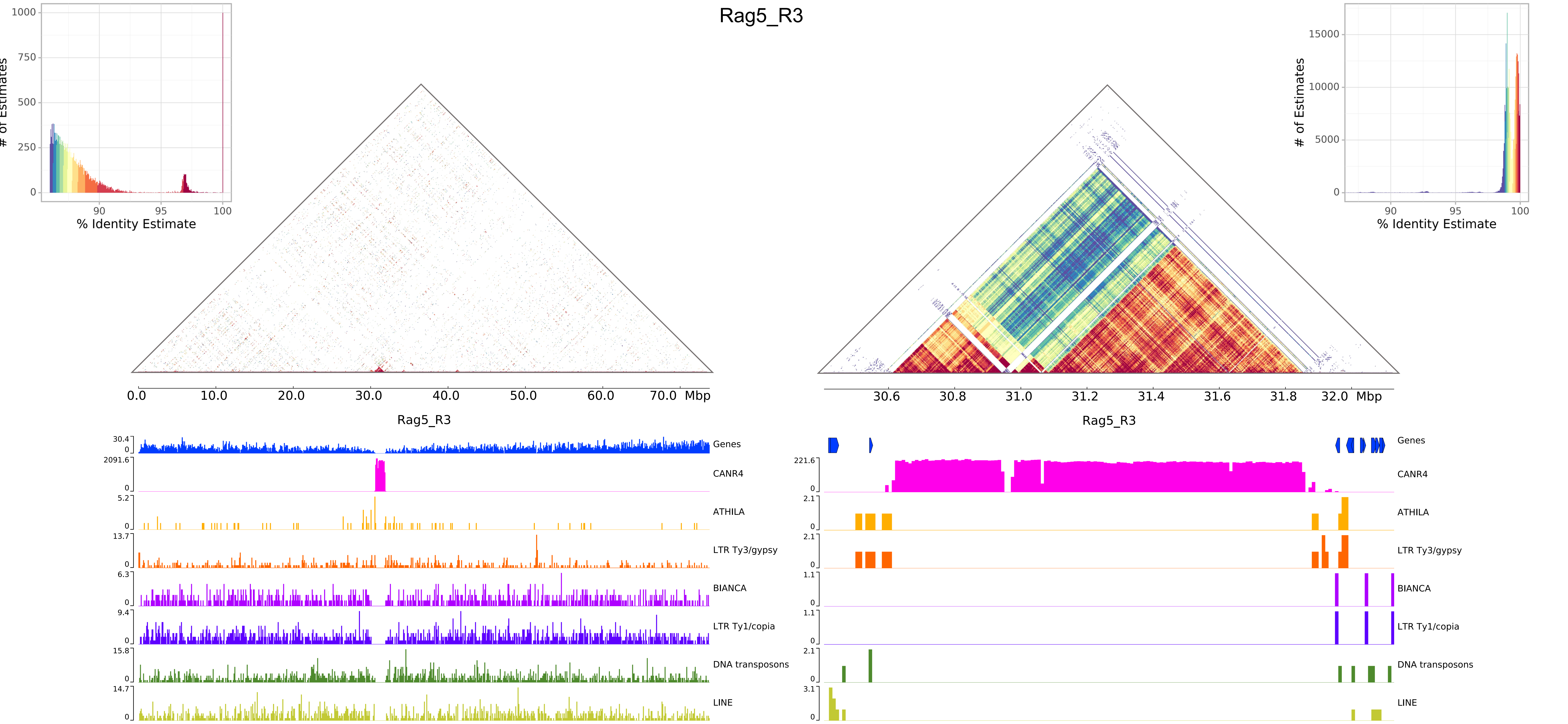
Rag5_R3
# of Estimates
% Identity Estimate
Mbp
Rag5_R3
Genes
CANR4
ATHILA
LTR Ty3/gypsy
BIANCA
LTR Ty1/copia
DNA transposons
LINE

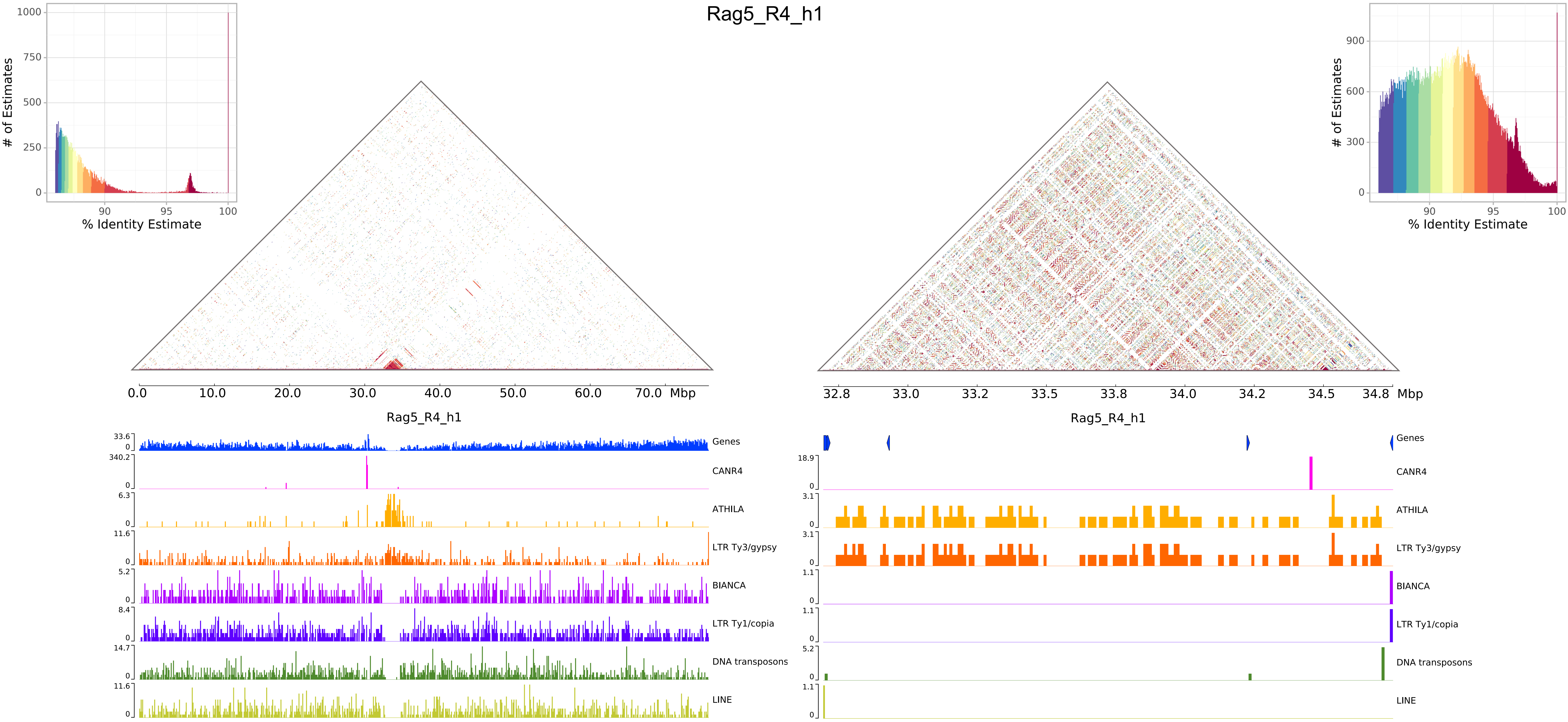
Rag5_R4_h1
# of Estimates
% Identity Estimate
1000
750
500
250
0
90
95
100
900
600
300
0
0.0
10.0
20.0
30.0
40.0
50.0
60.0
70.0
Mbp
32.8
33.0
33.2
33.5
33.8
34.0
34.2
34.5
34.8
Mbp
Rag5_R4_h1
Rag5_R4_h1
Genes
CANR4
ATHILA
LTR Ty3/gypsy
BIANCA
LTR Ty1/copia
DNA transposons
LINE
33.6
340.2
6.3
11.6
5.2
8.4
14.7
11.6
18.9
3.1
3.1
1.1
1.1
5.2
1.1

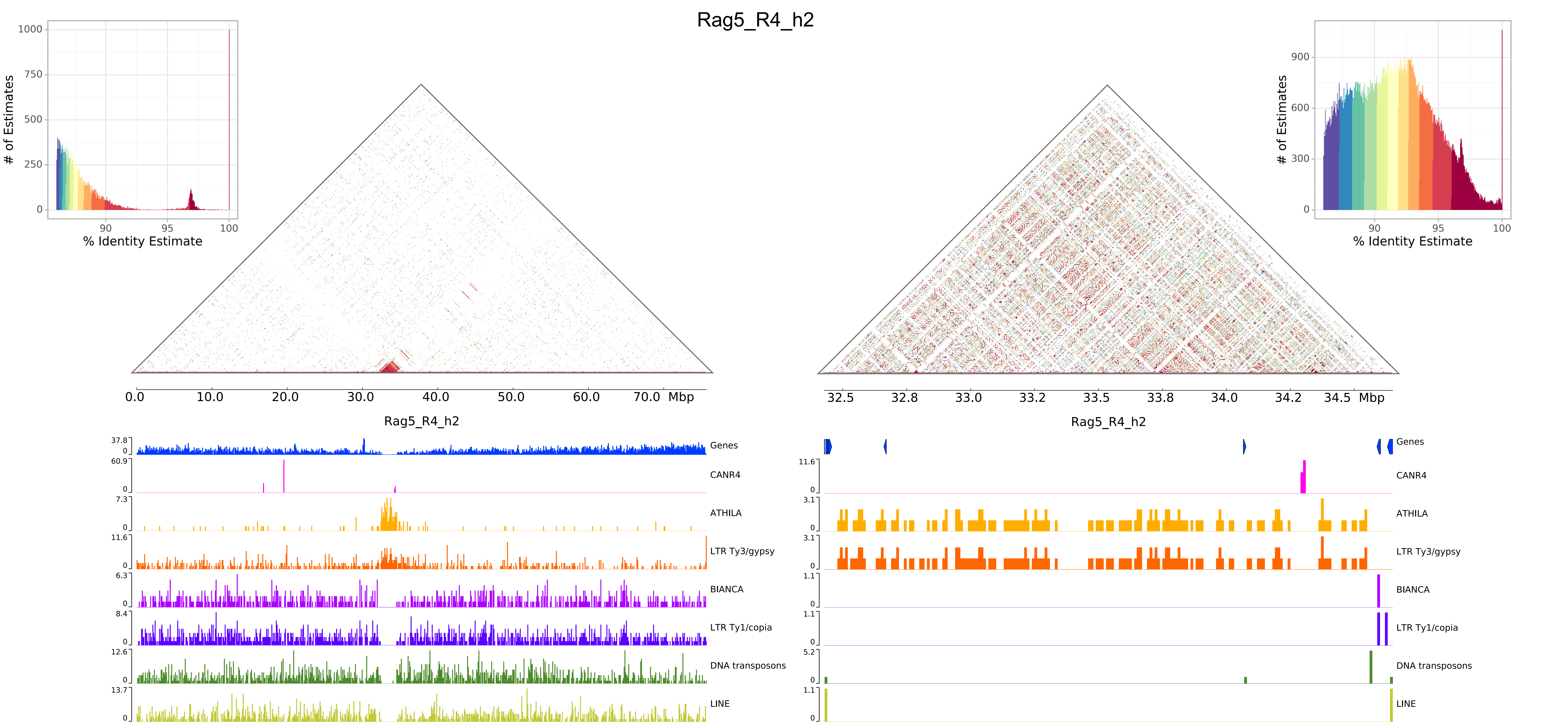
Rag5_R4_h2
# of Estimates
% Identity Estimate
0.0
10.0
20.0
30.0
40.0
50.0
60.0
70.0 Mbp
32.5
32.8
33.0
33.2
33.5
33.8
34.0
34.2
34.5 Mbp
Rag5_R4_h2
Rag5_R4_h2
Genes
CANR4
ATHILA
LTR Ty3/gypsy
BIANCA
LTR Ty1/copia
DNA transposons
LINE

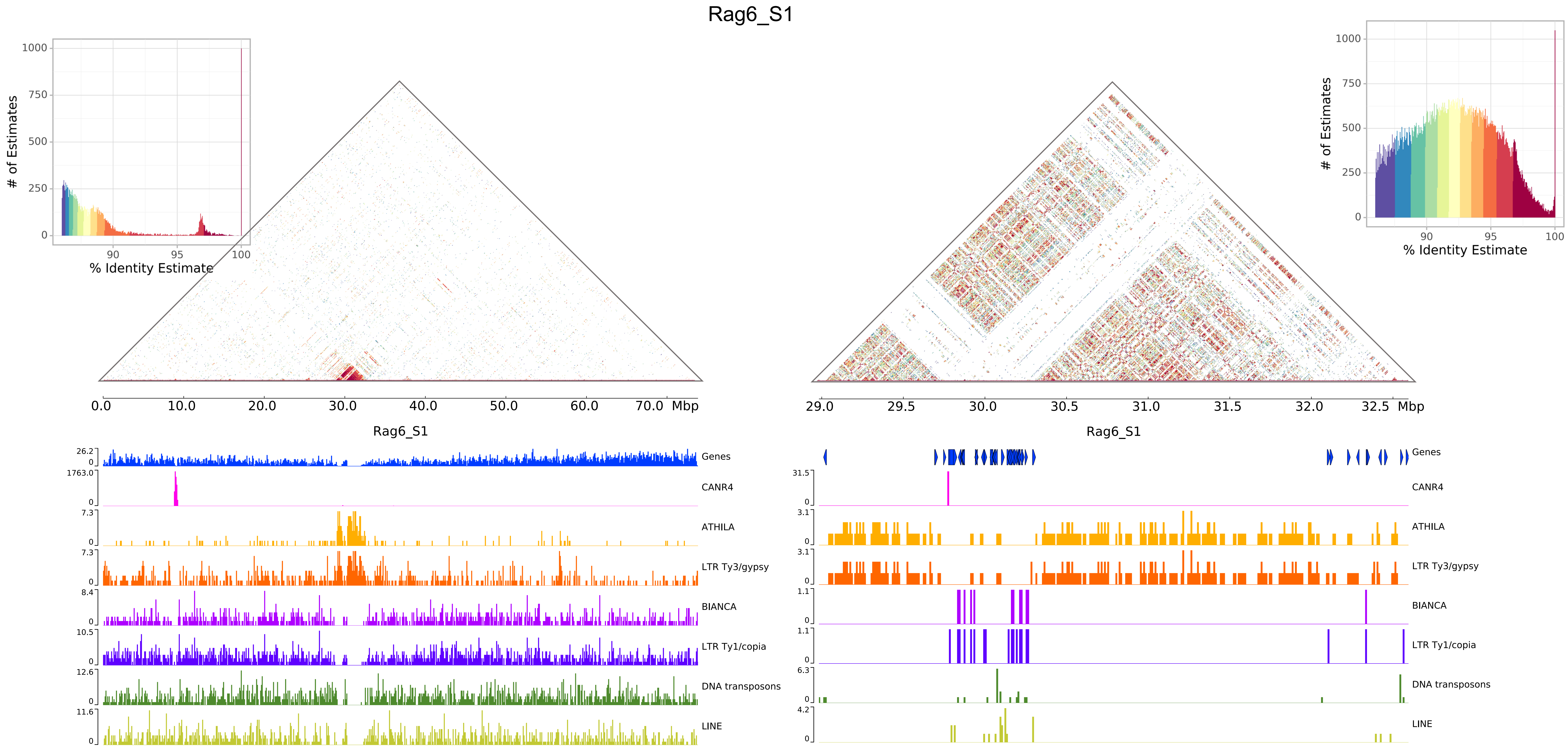
Rag6_S1
1000
750
500
250
0
# of Estimates
90
95
100
% Identity Estimate
0.0
10.0
20.0
30.0
40.0
50.0
60.0
70.0
Mbp
Rag6_S1
29.0
29.5
30.0
30.5
31.0
31.5
32.0
32.5
Mbp
Rag6_S1
26.2
1763.0
7.3
7.3
8.4
10.5
12.6
11.6
Genes
CANR4
ATHILA
LTR Ty3/gypsy
BIANCA
LTR Ty1/copia
DNA transposons
LINE
31.5
3.1
3.1
1.1
1.1
6.3
4.2

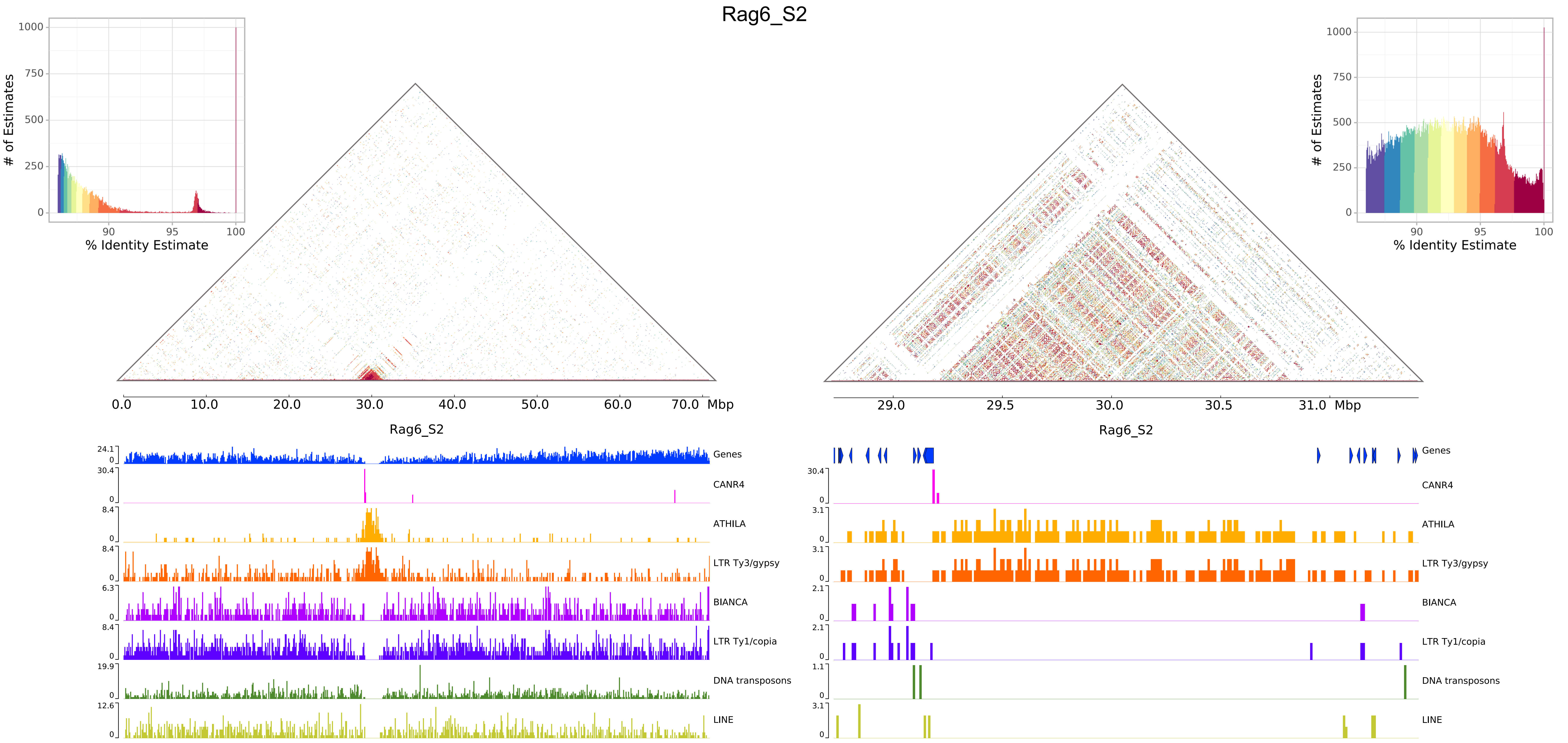

Rag6_S2
# of Estimates
% Identity Estimate
1000
750
500
250
0
90
95
100
0.0
10.0
20.0
30.0
40.0
50.0
60.0
70.0
Mbp
29.0
29.5
30.0
30.5
31.0
Rag6_S2
Genes
CANR4
ATHILA
LTR Ty3/gypsy
BIANCA
LTR Ty1/copia
DNA transposons
LINE
24.1
30.4
8.4
6.3
19.9
12.6
3.1
2.1
1.1

Rag6_R3

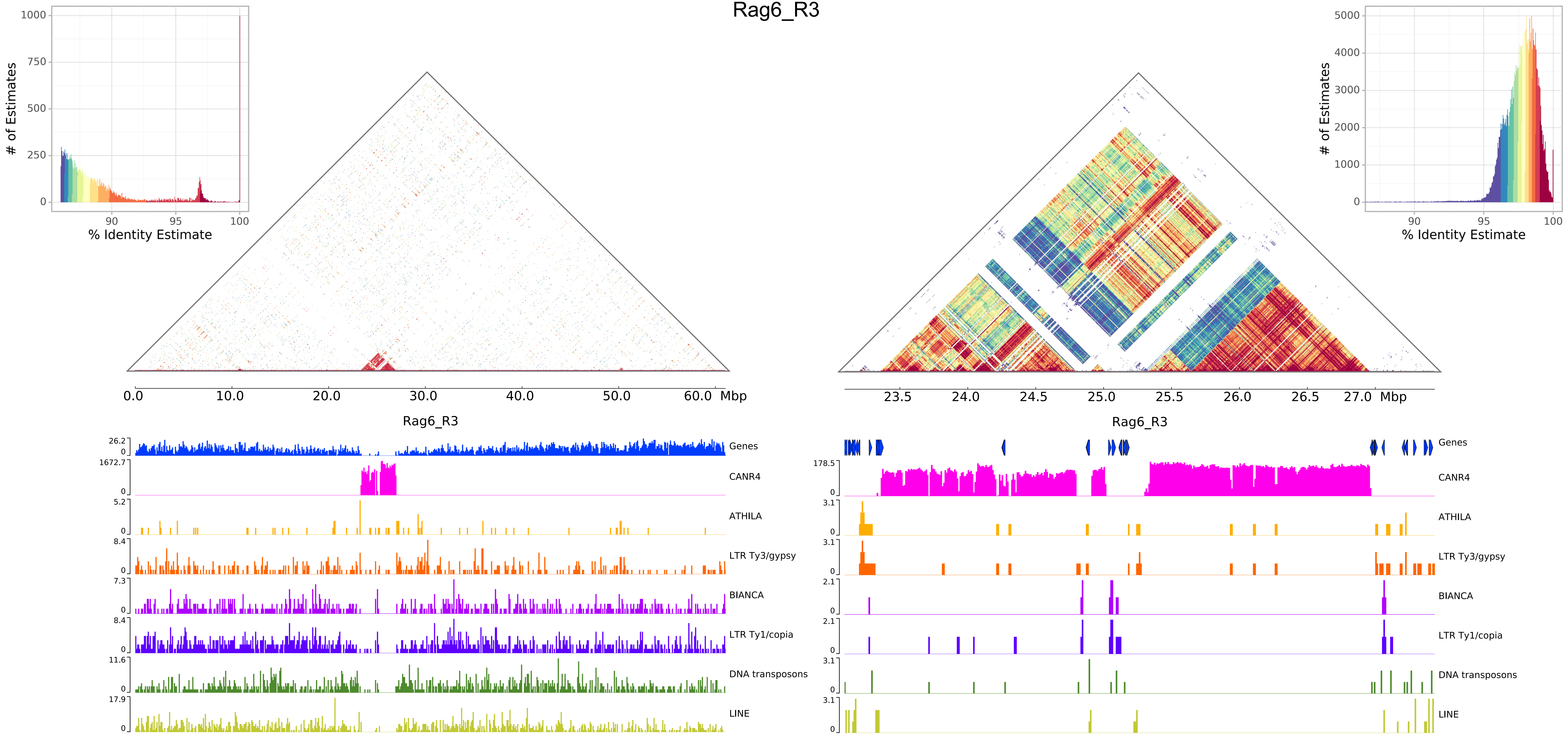

1000
750
500
250
0
# of Estimates
90
95
100
% Identity Estimate
0.0
10.0
20.0
30.0
40.0
50.0
60.0
Mbp
Rag6_R3
26.2
0
Genes
1672.7
CANR4
5.2
ATHILA
8.4
LTR Ty3/gypsy
7.3
BIANCA
8.4
LTR Ty1/copia
11.6
DNA transposons
17.9
LINE
5000
4000
3000
2000
1000
23.5
24.0
24.5
25.0
25.5
26.0
26.5
27.0
178.5
3.1
2.1

Rag6_R4_h1

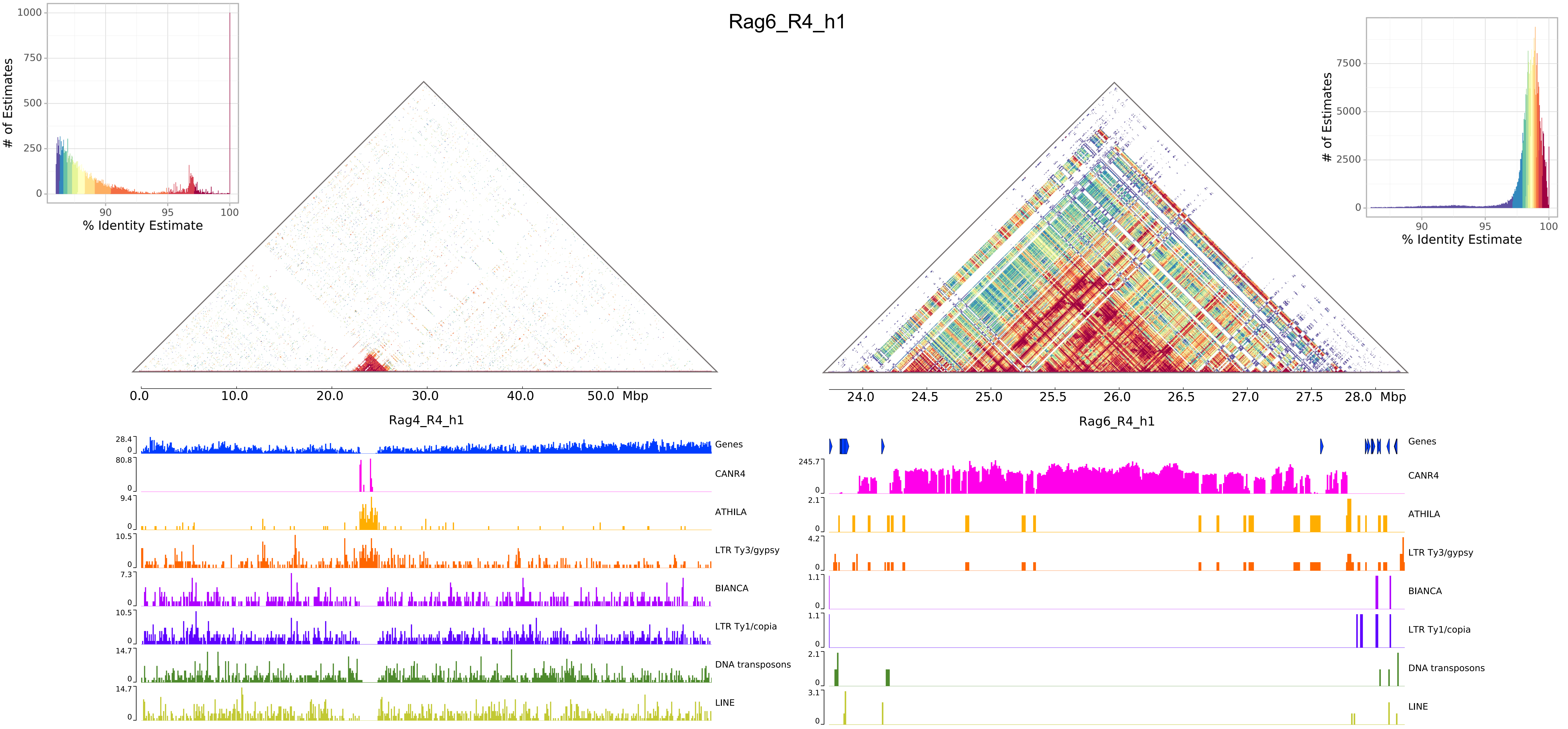

# of Estimates
% Identity Estimate
0.0 10.0 20.0 30.0 40.0 50.0 Mbp
Rag4_R4_h1
24.0 24.5 25.0 25.5 26.0 26.5 27.0 27.5 28.0 Mbp
Rag6_R4_h1
Genes
CANR4
ATHILA
LTR Ty3/gypsy
BIANCA
LTR Ty1/copia
DNA transposons
LINE

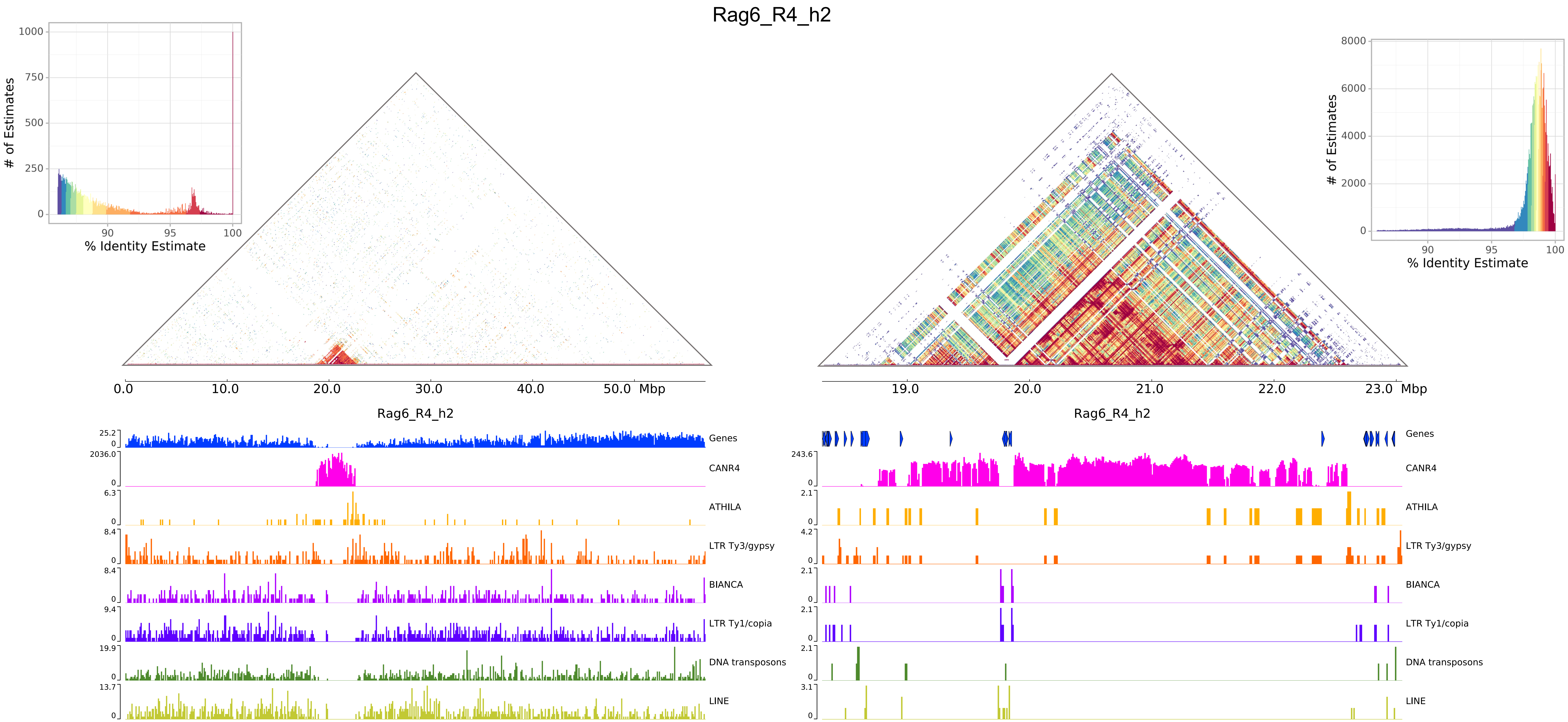

Rag6_R4_h2
# of Estimates
% Identity Estimate
1000
750
500
250
0
90
95
100
8000
6000
4000
2000
0.0
10.0
20.0
30.0
40.0
50.0 Mbp
19.0
20.0
21.0
22.0
23.0 Mbp
Rag6_R4_h2
Genes
CANR4
ATHILA
LTR Ty3/gypsy
BIANCA
LTR Ty1/copia
DNA transposons
LINE
25.2
2036.0
6.3
8.4
8.4
9.4
19.9
13.7
243.6
2.1
4.2
2.1
2.1
2.1
3.1

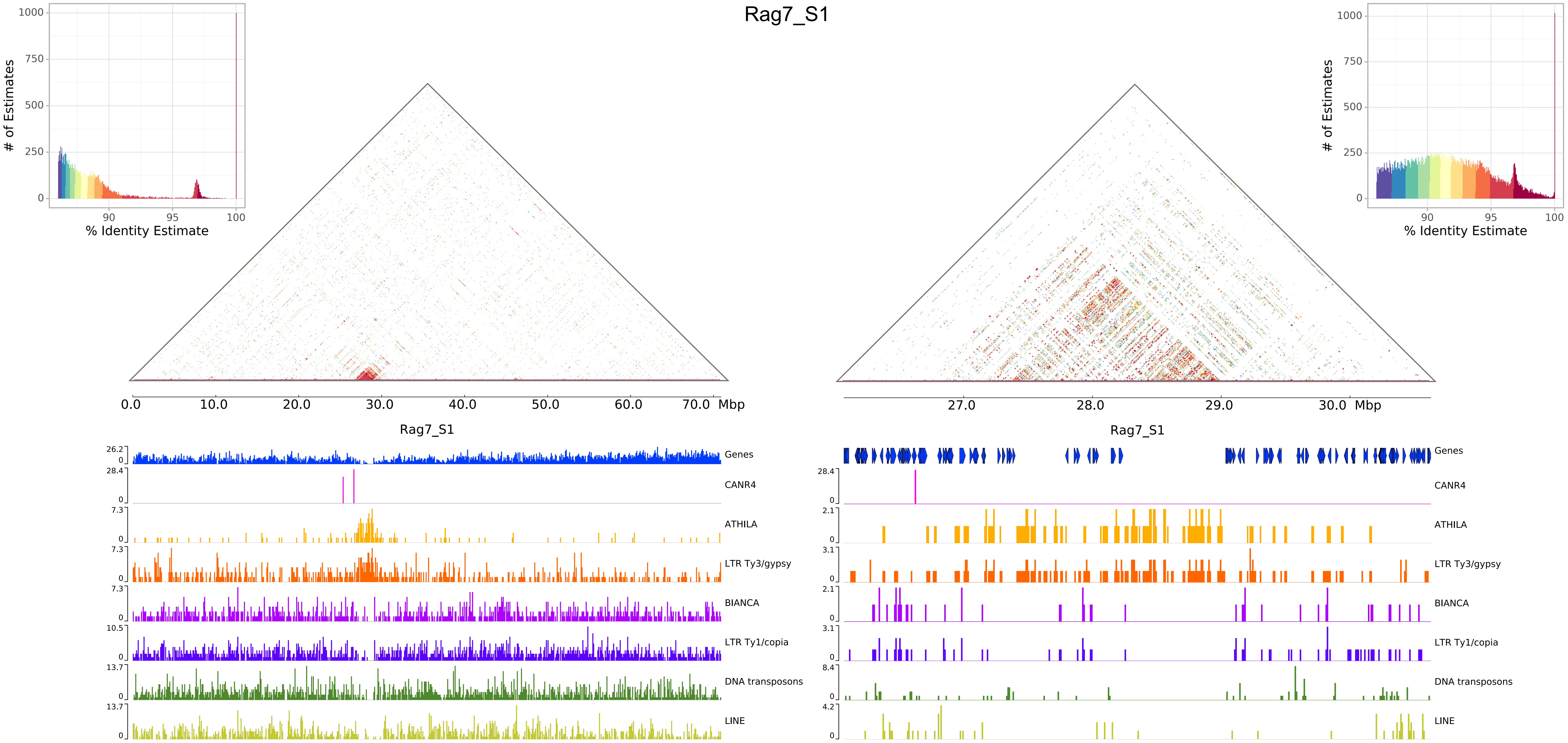

Rag7_S1
# of Estimates
% Identity Estimate
1000
750
500
250
0
90
95
100
0.0
10.0
20.0
30.0
40.0
50.0
60.0
70.0
Mbp
27.0
28.0
29.0
30.0
Rag7_S1
Genes
CANR4
ATHILA
LTR Ty3/gypsy
BIANCA
LTR Ty1/copia
DNA transposons
LINE
26.2
28.4
7.3
10.5
13.7
2.1
3.1
8.4
4.2

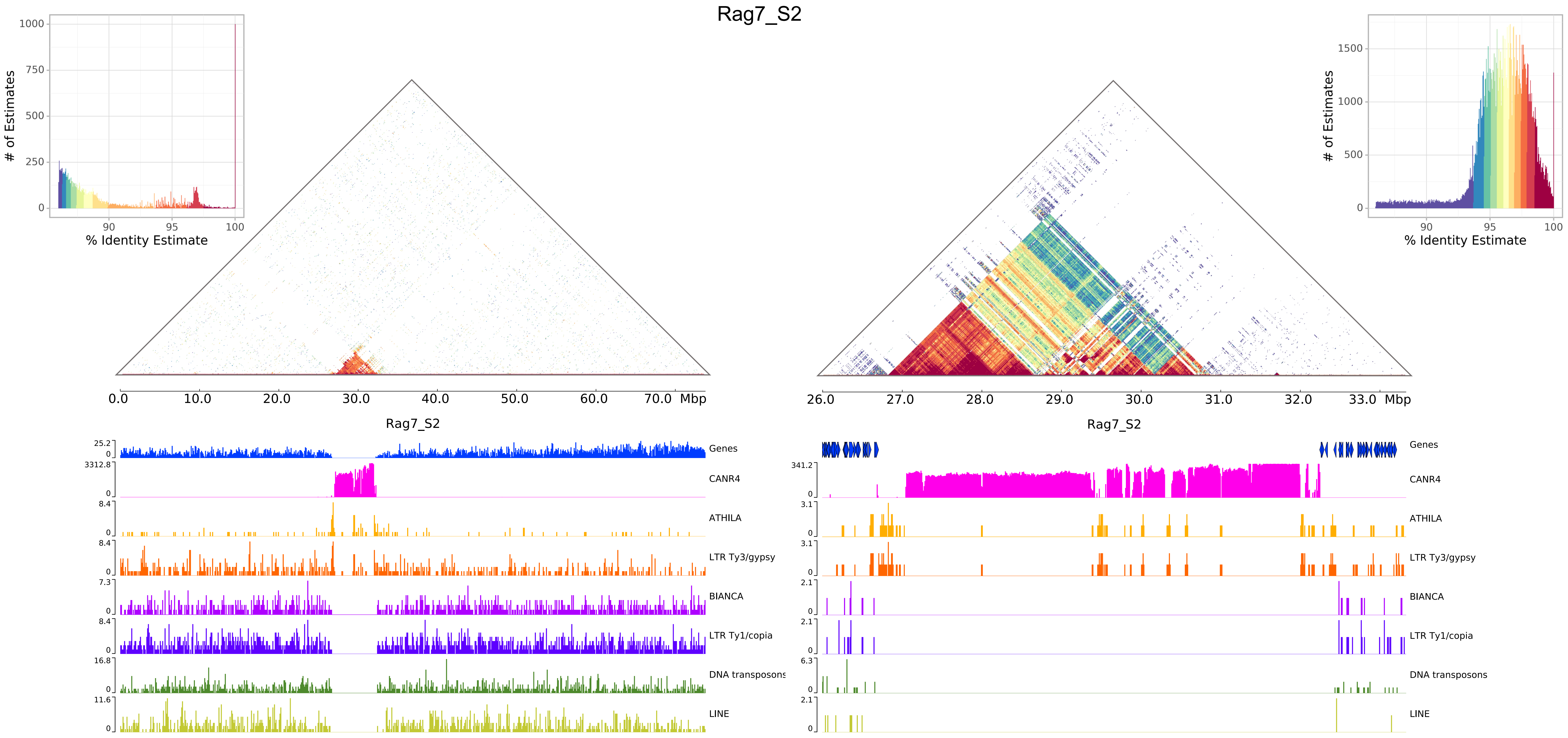
Rag7_S2
# of Estimates
% Identity Estimate
1000
750
500
250
0
90
95
100
1500
0.0
10.0
20.0
30.0
40.0
50.0
60.0
70.0
Mbp
26.0
27.0
28.0
29.0
30.0
31.0
32.0
33.0
Genes
CANR4
ATHILA
LTR Ty3/gypsy
BIANCA
LTR Ty1/copia
DNA transposons
LINE
25.2
3312.8
8.4
7.3
16.8
11.6
341.2
3.1
2.1
6.3

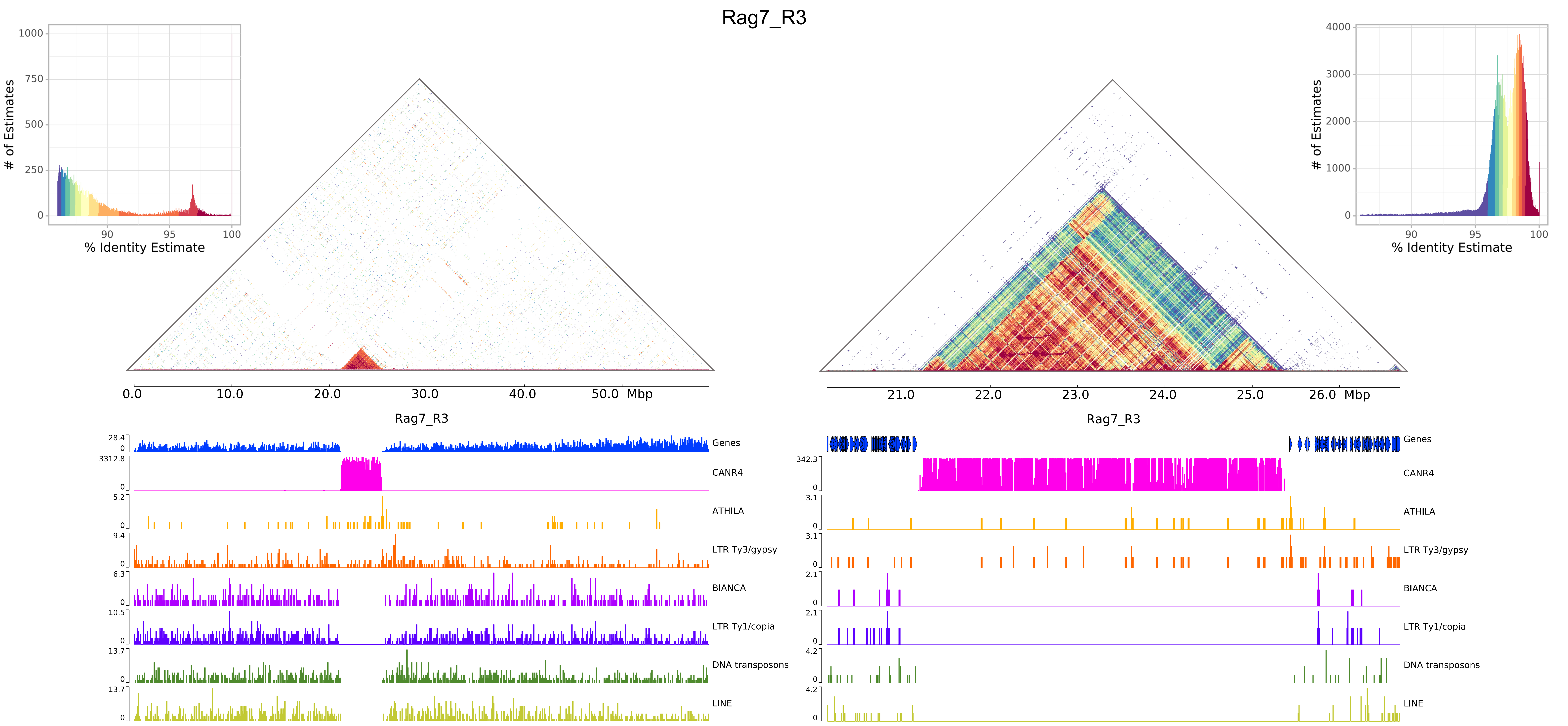

Rag7_R3
# of Estimates
% Identity Estimate
1000
750
500
250
0
90
95
100
4000
3000
2000
1000
0.0
10.0
20.0
30.0
40.0
50.0 Mbp
21.0
22.0
23.0
24.0
25.0
26.0 Mbp
Rag7_R3
Genes
CANR4
ATHILA
LTR Ty3/gypsy
BIANCA
LTR Ty1/copia
DNA transposons
LINE
28.4
3312.8
5.2
9.4
6.3
10.5
13.7
342.3
3.1
2.1
4.2

Rag7_R4_h1

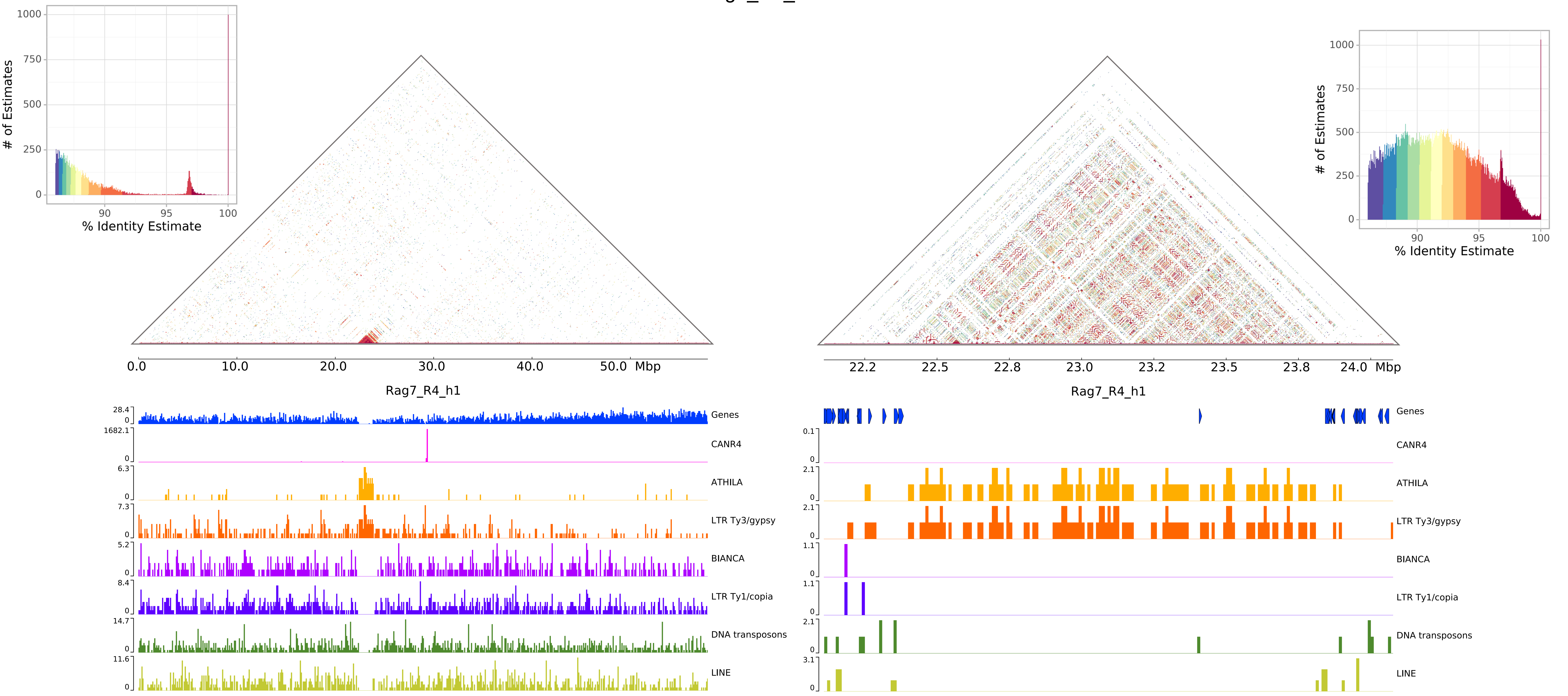

1000
750
500
250
0
# of Estimates
90
95
100
% Identity Estimate
0.0
10.0
20.0
30.0
40.0
50.0
Mbp
Rag7_R4_h1
22.2
22.5
22.8
23.0
23.2
23.5
23.8
24.0
Mbp
Rag7_R4_h1
28.4
1682.1
6.3
7.3
5.2
8.4
14.7
11.6
0.1
2.1
2.1
1.1
1.1
2.1
3.1
Genes
CANR4
ATHILA
LTR Ty3/gypsy
BIANCA
LTR Ty1/copia
DNA transposons
LINE

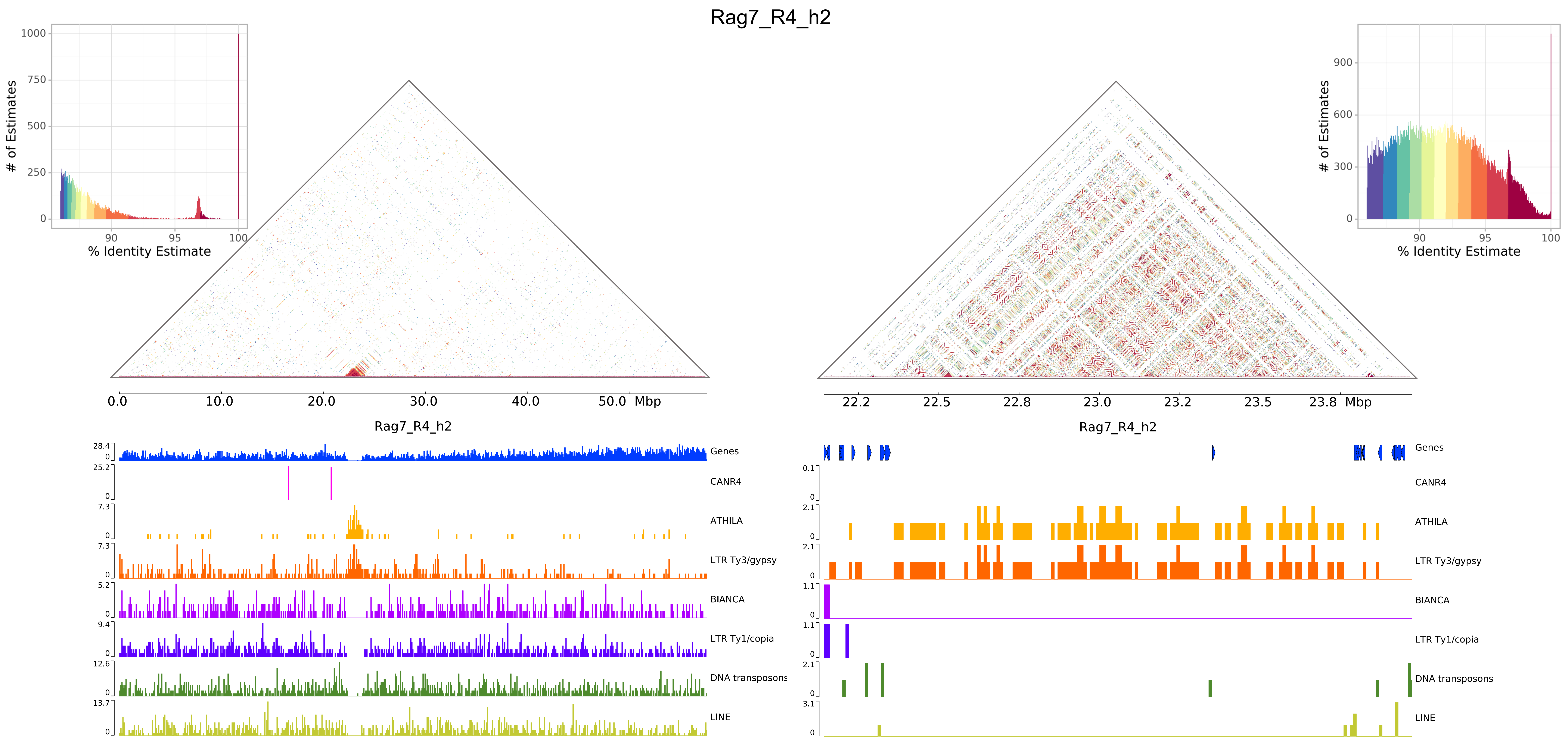
Rag7_R4_h2
# of Estimates
% Identity Estimate
1000
750
500
250
0
90
95
100
0.0
10.0
20.0
30.0
40.0
50.0
Mbp
Rag7_R4_h2
900
600
300
22.2
22.5
22.8
23.0
23.2
23.5
23.8
Genes
CANR4
ATHILA
LTR Ty3/gypsy
BIANCA
LTR Ty1/copia
DNA transposons
LINE
28.4
25.2
7.3
5.2
9.4
12.6
13.7
0.1
2.1
1.1
3.1
